# Extraction of gravitational waves in numerical relativity

**DOI:** 10.1007/s41114-016-0001-9

**Published:** 2016-10-04

**Authors:** Nigel T. Bishop, Luciano Rezzolla

**Affiliations:** 1 Department of Mathematics, Rhodes University, Grahamstown, 6140 South Africa; 2 Institute for Theoretical Physics, 60438 Frankfurt am Main, Germany; 3 Frankfurt Institute for Advanced Studies, 60438 Frankfurt am Main, Germany

**Keywords:** Gravitational wave extraction, Numerical relativity, Binary mergers, Black holes, Neutron stars

## Abstract

A numerical-relativity calculation yields in general a solution of the Einstein equations including also a radiative part, which is in practice computed in a region of finite extent. Since gravitational radiation is properly defined only at null infinity and in an appropriate coordinate system, the accurate estimation of the emitted gravitational waves represents an old and non-trivial problem in numerical relativity. A number of methods have been developed over the years to “extract” the radiative part of the solution from a numerical simulation and these include: quadrupole formulas, gauge-invariant metric perturbations, Weyl scalars, and characteristic extraction. We review and discuss each method, in terms of both its theoretical background as well as its implementation. Finally, we provide a brief comparison of the various methods in terms of their inherent advantages and disadvantages.

**Electronic supplementary material:**

The online version of this article (doi:10.1007/s41114-016-0001-9) contains supplementary material, which is available to authorized users.

## Introduction

With the commissioning of the second generation of laser interferometric gravitational-wave detectors, and the recent detection of gravitational waves (Abbott 2016), there is considerable interest in gravitational-wave astronomy. This is a huge field, covering the diverse topics of: detector hardware construction and design; data analysis; astrophysical source modeling; approximate methods for gravitational-wave calculation; and, when the weak field approach is not valid, numerical relativity.

Numerical relativity is concerned with the construction of a numerical solution to the Einstein equations, so obtaining an approximate description of a spacetime, and is reviewed, for example, in the textbooks by Alcubierre (2008), Bona et al. (2009), Baumgarte and Shapiro (2010), Gourgoulhon (2012) and Rezzolla and Zanotti (2013). The physics in the simulation may be only gravity, as is the case of a binary black hole scenario, but it may also include matter fields and/or electromagnetic fields. Thus numerical relativity may be included in the modeling of a wide range of astrophysical processes. Often (but not always), an important desired outcome of the modeling process will be a prediction of the emitted gravitational waves. However, obtaining an accurate estimate of gravitational waves from the variables evolved in the simulation is normally a rather complicated process. The key difficulty is that gravitational waves are unambiguously defined only at future null infinity ($$\mathcal
                     {J}^+$$), whereas in practice the domain of numerical simulations is a region of finite extent using a “3+1” foliation of the spacetime. This is true for most of the numerical codes, but there are also notable exceptions. Indeed, there have been attempts towards the construction of codes that include both null infinity and the central dynamic region in the domain, but they have not been successful in the general case. These attempts include the hyperboloidal method (Frauendiener 2004), Cauchy characteristic matching (Winicour 2005), and a characteristic code (Bishop et al. 1997b). The only successful application to an astrophysical problem has been to axisymmetric core collapse using a characteristic code (Siebel et al. 2003).

In the linearized approximation, where gravitational fields are weak and velocities are small, it is straightforward to derive a relationship between the matter dynamics and the emission of gravitational waves, the well-known quadrupole formula. This can be traced back to work by Einstein (1916, 1918) shortly after the publication of general relativity. The method is widely used to estimate gravitational-wave production in many astrophysical processes. However, the strongest gravitational-wave signals come from highly compact systems with large velocities, that is from processes where the linearized assumptions do not apply. And of course, it is an event producing a powerful signal that is most likely to be found in gravitational-wave detector data. Thus it is important to be able to calculate gravitational-wave emission accurately for processes such as black hole or neutron star inspiral and merger, stellar core collapse, etc. Such problems cannot be solved analytically and instead are modeled by numerical relativity, as described in the previous paragraph, to compute the gravitational field near the source. The procedure of using this data to measure the gravitational radiation far from the source is called “extraction” of gravitational waves from the numerical solution.

In addition to the quadrupole formula and full numerical relativity, there are a number of other approaches to calculating gravitational-wave emission from astrophysical sources. These techniques are not discussed here and are reviewed elsewhere. They include post-Newtonian methods (Blanchet 2014), effective one-body methods (Damour and Nagar 2016), and self-force methods (Poisson et al. 2011). Another approach, now no-longer pursued, is the so-called “Lazarus approach”, that combined analytical and numerical techniques (Baker et al. 2000b, 2002a, b).

In this article we will review a number of different extraction methods: (a) Quadrupole formula and its variations (Sect. 2.3); (b) methods using the Newman–Penrose scalar $$\psi
                     _4$$ evaluated on a worldtube ($$\varGamma
                     $$) (Sect. 3.3); (c) Cauchy Perturbative methods, using data on $$\varGamma
                     $$ to construct an approximation to a perturbative solution on a known curved background (Sects. 4, 5; Abrahams and Evans 1988, 1990); and (d) Characteristic extraction, using data on $$\varGamma
                     $$ as inner boundary data for a characteristic code to find the waveform at $$\mathcal
                     {J}^+$$ (Sects. 6, 7). The description of the methods is fairly complete, with derivations given from first principles and in some detail. In cases (c) and (d), the theory involved is quite lengthy, so we also provide implementation summaries for the reader who is more interested in applying, rather than fully understanding, a particular method, see Sects. 5.6 and 7.8.

In addition, this review provides background material on gravitational waves (Sect. 2), on the “3+1” formalism for evolving the Einstein equations (Sect. 3), and on the characteristic formalism with particular reference to its use in estimating gravitational radiation (Sect. 6). The review concludes with a comparison of the various methods for extracting gravitational waves (Sect. 8). This review uses many different symbols, and their use and meaning is summarized in “Appendix 1”. Spin-weighted, and other, spherical harmonics are discussed in “Appendix 2”, and various computer algebra scripts and numerical codes are given in “Appendix 3”.

Throughout, we will use a spacelike signature $$(-,+,+,+)$$ and a system of geometrised units in which $$G = c =
                     1$$, although when needed we will also indicate the speed of light, *c*, explicitly. We will indicate with a boldface any tensor, e.g., $$\varvec{V}$$ and with the standard arrow any three-dimensional vector or operator, e.g., $$\mathbf
                     {\varvec{v}}$$ and $$\mathbf {\nabla
                     }$$. Four-dimensional covariant and partial derivatives will be indicated in general with $$\nabla _{\mu
                     }$$ and $$\partial _{\mu
                     }$$, but other symbols may be introduced for less common definitions, or when we want to aid the comparison with classical Newtonian expressions. Within the standard convention of a summation of repeated indices, Greek letters will be taken to run from 0 to 3, while Latin indices run from 1 to 3.

We note that some of the material in this review has already appeared in books or other review articles. In particular, we have abundantly used parts of the text from the book “Relativistic Hydrodynamics”, by Rezzolla and Zanotti (2013), from the review article “Gauge-invariant non-spherical metric perturbations of Schwarzschild black-hole spacetimes”, by Nagar and Rezzolla (2006), as well as adaptations of the text from the article “Cauchy-characteristic matching”, by Bishop et al. (1999a).

## A quick review of gravitational waves

### Linearized Einstein equations

When considering the Einstein equations

1 $$\begin{aligned} G_{\mu
                        \nu } = R_{\mu \nu }-\frac{1}{2}R\, g_{\mu \nu } = 8\pi T_{\mu \nu },
                        \end{aligned}$$

as a set of second-order partial differential equations it is not easy to predict that there exist solutions behaving as waves. Indeed, the concept of gravitational waves as solutions of the Einstein equations written as linear and homogeneous wave equations is valid only under some rather idealised assumptions, such as a vacuum and asymptotically flat spacetime, a linearised regime for the gravitational fields and suitable gauges. If these assumptions are removed, the definition of gravitational waves becomes much more difficult, although still possible. It should be noted, however, that in this respect gravitational waves are not peculiar. Any wave-like phenomenon, in fact, can be described in terms of homogeneous wave equations only under simplified assumptions, such as those requiring a uniform “background” for the fields propagating as waves.

These considerations suggest that the search for wave-like solutions to the Einstein equations should be made in a spacetime with very modest curvature and with a line element which is that of flat spacetime but for small deviations of nonzero curvature, i.e., 

2 $$\begin{aligned} g_{\mu
                        \nu } = \eta _{\mu \nu } + h_{\mu \nu } + \mathcal {O}\left( (h_{\mu \nu
                        })^2\right) , \end{aligned}$$

where the linearised regime is guaranteed by the fact that $$|h_{\mu \nu }| \ll
                        1$$. Before writing the linearised version of the Einstein equations (1) it is necessary to derive the linearised expression for the Christoffel symbols. In a Cartesian coordinate basis (such as the one we will assume hereafter), we recall that the general expression for the affine connection is given by

3 $$\begin{aligned}
                        \varGamma _{\beta \gamma }^{\alpha }=\frac{1}{2}g^{\alpha \delta } \left(
                        \partial _{\gamma } g_{\delta \beta } + \partial _{\beta } g_{\delta \gamma
                        } - \partial _{\delta } g_{\beta \gamma } \right) .
                        \end{aligned}$$

where the partial derivatives are readily calculated as

4 $$\begin{aligned} \partial
                        _{\beta }g_{\nu \alpha } = \partial _{\beta }\eta _{\nu \alpha } + \partial
                        _{\beta }h_{\nu \alpha } = \partial _{\beta }h_{\nu \alpha } .
                        \end{aligned}$$

As a result, the linearised Christoffel symbols become

5 $$\begin{aligned}
                        \varGamma ^{\mu }_{\ \alpha \beta } = \frac{1}{2}\eta ^{\mu \nu }( \partial
                        _{\beta }h_{\nu \alpha } + \partial _{\alpha }h_{\nu \beta } - \partial
                        _{\nu }h_{\alpha \beta }) = \frac{1}{2}(\partial _{\beta }h^{\mu }_{\ \
                        \alpha } + \partial _{\alpha }h^{\mu }_{\ \ \beta } - \partial ^{\mu
                        }h_{\alpha \beta }) . \end{aligned}$$

Note that the operation of lowering and raising the indices in expression (5) is not made through the metric tensors $$g_{\mu \nu
                        }$$ and $$g^{\mu \nu
                        }$$ but, rather, through the spacetime metric tensors $$\eta _{\mu \nu
                        }$$ and $$\eta ^{\mu \nu
                        }$$. This is just the consequence of the linearised approximation and, despite this, the spacetime is really curved!

Once the linearised Christoffel symbols have been computed, it is possible to derive the linearised expression for the Ricci tensor which takes the form

6 $$\begin{aligned} R_{\mu
                        \nu } =\partial _{\alpha }\varGamma ^{\alpha }_{\ \mu \nu } - \partial _{\nu
                        }\varGamma ^{\alpha }_{\ \mu \alpha } = \frac{1}{2} (\partial _{\alpha
                        }\partial _{\nu } h^{\ \;\alpha }_{\mu } + \partial _{\alpha }\partial _{\mu
                        } h^{\ \;\alpha }_{\nu } - \partial _{\alpha }\partial ^{\alpha } h_{\mu \nu
                        } - \partial _{\mu }\partial _{\nu } h),
                        \end{aligned}$$

where

7 $$\begin{aligned} h
                        :=h^{\alpha }_{\ \; \alpha } = \eta ^{\mu \alpha } h_{\mu \alpha }
                        \end{aligned}$$

is the trace of the metric perturbations. The resulting Ricci scalar is then given by

8 $$\begin{aligned} R
                        :=g^{\mu \nu } R_{\mu \nu } \simeq \eta ^{\mu \nu } R_{\mu \nu } .
                        \end{aligned}$$

Making use of (6) and (8), it is possible to rewrite the Einstein equations (1) in a linearised form as

9 $$\begin{aligned} \partial
                        ^{\alpha }\partial _{\nu } h_{\mu \alpha } + \partial ^{\alpha }\partial
                        _{\mu } h_{\nu \alpha } - \partial _{\alpha }\partial ^{\alpha } h_{\mu \nu
                        } - \partial _{\mu }\partial _{\nu }h - \eta _{\mu \nu }( \partial ^{\alpha
                        }\partial ^{\beta }h_{\alpha \beta } - \partial ^{\alpha }\partial _{\alpha
                        }h) = 16 \pi T_{\mu \nu } . \end{aligned}$$

Although linearised, the Einstein equations (9) do not yet seem to suggest a wave-like behaviour. A good step in the direction of unveiling this behaviour can be made if we introduce a more compact notation, which makes use of “trace-free” tensors defined as

10 $$\begin{aligned}
                        {\bar{h}}_{\mu \nu } :=h_{\mu \nu } - \frac{1}{2} \eta _{\mu \nu } h,
                        \end{aligned}$$

where the “bar-operator” in (10) can be applied to any symmetric tensor so that, for instance, $${\bar{R}}_{\mu \nu } =
                        G_{\mu \nu }$$, and also iteratively, i.e., $${\bar{\bar{h}}}_{\mu \nu
                        } = h_{\mu \nu }$$.^1^ Using this notation, the linearised Einstein equations (9) take the more compact form

11 $$\begin{aligned}
                        -\partial ^{\alpha }\partial _{\alpha }{\bar{h}}_{\mu \nu } - \eta _{\mu \nu
                        }\, \partial ^{\alpha }\partial ^{\beta }{\bar{h}}_{\alpha \beta } +
                        \partial ^{\alpha }\partial _{\mu }{\bar{h}}_{\nu \alpha } = 16 \pi T_{\mu
                        \nu }, \end{aligned}$$

where the first term on the left-hand side of (11) can be easily recognised as the *Dalambertian* (or wave) operator, i.e., $$\partial _{\alpha
                        }\partial ^{\alpha }{\bar{h}}_{\mu \nu } = \square {\bar{h}}_{\mu \nu
                        }$$. At this stage, we can exploit the gauge freedom inherent in general relativity (see also below for an extended discussion) to recast Eq. (11) in a more convenient form. More specifically, we exploit this gauge freedom by choosing the metric perturbations $$h_{\mu \nu
                        }$$ so as to eliminate the terms in (11) that spoil the wave-like structure. Most notably, the coordinates can be selected so that the metric perturbations satisfy

12 $$\begin{aligned} \partial
                        _{\alpha } {\bar{h}}^{\mu \alpha } = 0 .
                        \end{aligned}$$

Making use of the gauge (12), which is also known as the *Lorenz* (or Hilbert) *gauge*, the linearised field equations take the form

13 $$\begin{aligned} \square
                        \,{\bar{h}}_{\mu \nu } = - 16 \pi T_{\mu \nu },
                        \end{aligned}$$

that, in vacuum reduce to the desired result

14 $$\begin{aligned} \square
                        \,{\bar{h}}_{\mu \nu } = 0 . \end{aligned}$$

Equations (14) show that, in the Lorenz gauge and in vacuum, the metric perturbations propagate as waves distorting flat spacetime.

The simplest solution to the linearised Einstein equations (14) is that of a plane wave of the type

15 $$\begin{aligned}
                        {\bar{h}}_{\mu \nu } = A_{\mu \nu } \exp (i \kappa _{\alpha }x^{\alpha }),
                        \end{aligned}$$

where of course we are interested only in the real part of (15), with $$\varvec{A}$$ being the *amplitude tensor*. Substitution of the ansatz (15) into Eq. (14) implies that $$\kappa ^{\alpha }\kappa
                        _{\alpha }=0$$ so that $$\varvec{\kappa
                        }$$ is a null four-vector. In such a solution, the plane wave (15) travels in the spatial direction $${\varvec{k}} = (\kappa
                        _x,\kappa _y,\kappa _z)/\kappa ^0$$ with frequency $$\omega :=\kappa ^0 =
                        (\kappa ^j\kappa _j)^{1/2}$$. The next step is to substitute the ansatz (15) into the Lorenz gauge condition Eq. (12), yielding $$A_{\mu \nu }\kappa ^{\mu
                        }=0$$ so that $$\varvec{A}$$ and $$\varvec{\kappa
                        }$$ are orthogonal. Consequently, the amplitude tensor $$\varvec{A}$$, which in principle has $$16-6=10$$ independent components, satisfies four conditions. Thus the imposition of the Lorenz gauge reduces the independent components of $$\varvec{A}$$ to six. We now investigate how to reduce the number of independent components to match the number of dynamical degrees of freedom of general relativity, i.e., two.

While a Lorenz gauge has been imposed [cf. Eq. (12)], this does not completely fix the coordinate system of a linearised theory. A residual ambiguity, in fact, is preserved through arbitrary *gauge changes*, i.e., through infinitesimal coordinate transformations that are consistent with the gauge that has been selected. The freedom to make such a transformation follows from a foundation of general relativity, the principle of general covariance. To better appreciate this matter, consider an infinitesimal coordinate transformation in terms of a small but otherwise arbitrary displacement four-vector $$\varvec{\xi
                        }$$

16 $$\begin{aligned}
                        x^{\alpha '} = x^{\alpha } + \xi ^{\alpha } .
                        \end{aligned}$$

Applying this transformation to the linearised metric (2) generates a “new” metric tensor that, to the lowest order, is

17 $$\begin{aligned}
                        g^\mathrm{new}_{\mu '\nu '} = \eta _{\mu \nu } + h^\mathrm{old}_{\mu \nu } -
                        \partial _{\nu }\xi _{\mu } - \partial _{\mu }\xi _{\nu },
                        \end{aligned}$$

so that the “new” and “old” perturbations are related by the following expression

18 $$\begin{aligned}
                        h^\mathrm{new}_{\mu '\nu '} = h^\mathrm{old}_{\mu \nu } - \partial _{\nu
                        }\xi _{\mu } - \partial _{\mu }\xi _{\nu },
                        \end{aligned}$$

or, alternatively, by

19 $$\begin{aligned}
                        {\bar{h}}^\mathrm{new}_{\mu '\nu '} = {\bar{h}}^\mathrm{old}_{\mu \nu } -
                        \partial _{\nu }\xi _{\mu } - \partial _{\mu }\xi _{\nu } + \eta _{\mu \nu
                        }\, \partial _{\alpha }\xi ^{\alpha }.
                        \end{aligned}$$

Requiring now that the new coordinates satisfy the condition (12) of the Lorenz gauge $$\partial ^{\alpha }
                        {\bar{h}}^\mathrm{new}_{\mu \alpha } = 0$$, forces the displacement vector to be solution of the homogeneous wave equation

20 $$\begin{aligned} \partial
                        _{\beta }\partial ^{\beta }\xi ^{\alpha } = 0 .
                        \end{aligned}$$

As a result, the plane-wave vector with components

21 $$\begin{aligned} \xi
                        ^{\alpha } :=-i C^{\alpha } \mathrm{exp}(i \kappa _{\beta }x ^{\beta })
                        \end{aligned}$$

generates, through the *four* arbitrary constants $$C^{\alpha
                        }$$, a gauge transformation that changes arbitrarily *four* components of $$\varvec{A}$$ in addition to those coming from the condition $$\varvec{A} \cdot
                        \varvec{\kappa }=0$$. Effectively, therefore, $$A_{\mu \nu
                        }$$ has only $$10-4-4=2$$ linearly independent components, corresponding to the number of degrees of freedom in general relativity (Misner et al. 1973).

Note that these considerations are not unique to general relativity and similar arguments can also be made in classical electrodynamics, where the Maxwell equations are invariant under transformations of the vector potentials of the type $$A_{\mu } \rightarrow
                        A_{\mu '} = A_{\mu } + \partial _{\mu }\varPsi $$, where $$\varPsi
                        $$ is an arbitrary scalar function, so that the corresponding electromagnetic tensor is $$F^\mathrm{new}_{\mu '
                        \nu '} = \partial _{\nu '} A_{\mu '} - \partial _{\mu '} A_{\nu '} =
                        F^\mathrm{old}_{\mu ' \nu '}$$. Similarly, in a linearised theory of general relativity, the gauge transformation (18) will preserve the components of the Riemann tensor, i.e., $$R^\mathrm{new}_{\alpha
                        \beta \mu \nu } = R^\mathrm{old}_{\alpha \beta \mu \nu } + \mathcal
                        {O}(R^2)$$.

To summarise, it is convenient to constrain the components of the amplitude tensor through the following conditions:

- *orthogonality condition*: four components of the amplitude tensor can be specified since the Lorenz gauge implies that $$\varvec{A}$$ and $$\varvec{\kappa
                                 }$$ are orthogonal, i.e., $$A_{\mu \nu }
                                 \kappa ^{\nu } = 0$$.
- *choice of observer*: three components of the amplitude tensor can be eliminated after selecting the infinitesimal displacement vector $$\xi ^{\mu } =
                                 iC^{\mu } \exp (i\kappa _{\alpha }x^{\alpha
                                 })$$ so that $$A^{\mu \nu
                                 }u_{\mu } =0$$ for some chosen four-velocity vector $$\varvec{u}$$. This means that the coordinates are chosen so that for an observer with four-velocity $$u^{\mu
                                 }$$ the gravitational wave has an effect only in spatial directions.^2^
- *traceless condition*: one final component of the amplitude tensor can be eliminated after selecting the infinitesimal displacement vector $$\xi ^{\mu } =
                                 iC^{\mu } \exp (i\kappa _{\alpha }x^{\alpha
                                 })$$ so that $$A^{\mu }_{\
                                 \,\mu } = 0$$.

Conditions *(a), (b)* and *(c)* define the so-called *transverse–traceless* (TT) *gauge*, which represents a most convenient gauge for the analysis of gravitational waves. To appreciate the significance of these conditions, consider them implemented in a reference frame which is globally at rest, i.e., with four-velocity $$u^{\alpha } =
                        (1,0,0,0)$$, where the amplitude tensor must satisfy:

- 22 $$\begin{aligned}
                                 A_{\mu \nu } \kappa ^{\nu } = 0 \qquad \Longleftrightarrow \qquad
                                 \partial ^j h_{ij} = 0, \end{aligned}$$
  i.e., the spatial components of $$h_{\mu \nu
                                 }$$ are *divergence-free*.
- 23 $$\begin{aligned}
                                 A_{\mu \nu } u^{\nu } = 0 \qquad \Longleftrightarrow \qquad h_{\mu
                                 t} = 0 , \end{aligned}$$
  i.e., only the spatial components of $$h_{\mu \nu
                                 }$$ are *nonzero*, hence the *transverse* character of the TT gauge.
- 24 $$\begin{aligned}
                                 A^{\mu }_{\ \, \mu } = 0 \qquad \Longleftrightarrow \qquad
                                 h=h^{j}_{\ j} = 0 , \end{aligned}$$
  i.e., the spatial components of $$h_{\mu \nu
                                 }$$ are *trace free* hence the *trace-free* character of the TT gauge. Because of this, and only in this gauge, $${\bar{h}}_{\mu
                                 \nu } = h_{\mu \nu }$$.

### Making sense of the TT gauge

As introduced so far, the TT gauge might appear rather abstract and not particularly interesting. Quite the opposite, the TT gauge introduces a number of important advantages and simplifications in the study of gravitational waves. The most important of these is that, in this gauge, the only nonzero components of the Riemann tensor are

25 $$\begin{aligned}
                        R_{j0k0}=R_{0j0k} = -R_{j00k} = -R_{0jk0}.
                        \end{aligned}$$

However, since

26 $$\begin{aligned}
                        R_{j0k0}=-\frac{1}{2} \partial ^2_t h^{^\mathrm{TT}}_{jk} ,
                        \end{aligned}$$

the use of the TT gauge indicates that a travelling gravitational wave with periodic time behaviour $$h^{^\mathrm{TT}}_{jk}
                        \propto \exp (i \omega t)$$ can be associated to a local oscillation of the spacetime, i.e., 

27 $$\begin{aligned} \partial
                        ^2_t h^{^\mathrm{TT}}_{jk} \sim -\omega ^2 \exp (i \omega t) \sim R_{j0k0},
                        \qquad \mathrm{and} \qquad \ R_{j0k0} = \frac{1}{2} \omega ^{2}
                        h^{^\mathrm{TT}}_{jk} . \end{aligned}$$

**Fig. 1:**
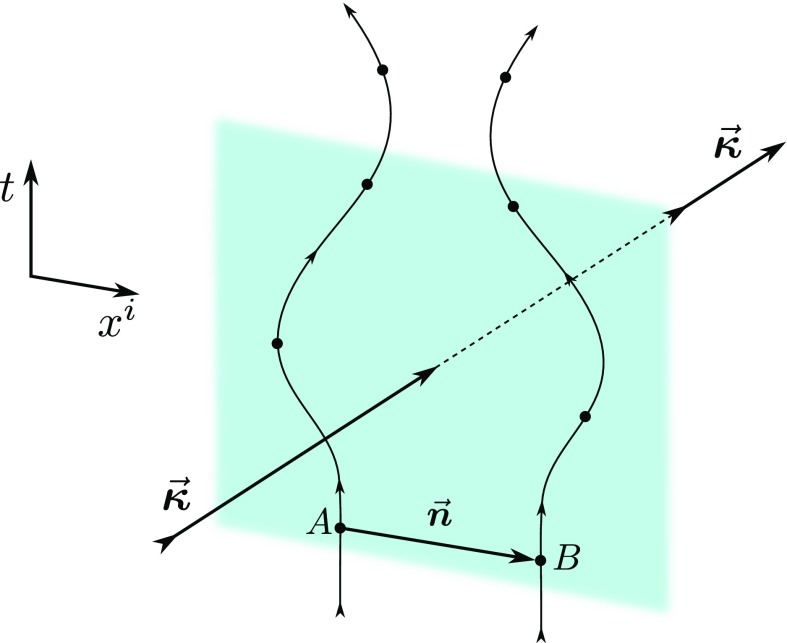
Schematic diagram for the changes in the separation vector $${\varvec{n}}$$ between two particles *A* and *B* moving along geodesic trajectories produced by the interaction with a gravitational wave propagating along the direction $${\varvec{\kappa
                                 }}$$

To better appreciate the effects of the propagation of a gravitational wave, it is useful to consider the separation between two neighbouring particles *A* and *B* on a geodesic motion and how this separation changes in the presence of an incident gravitational wave (see Fig. 1). For this purpose, let us introduce a coordinate system $$x^{\hat{\alpha
                        }}$$ in the neighbourhood of particle *A* so that along the worldline of the particle *A* the line element will have the form

28 $$\begin{aligned} ds^2 =
                        -d\tau ^2 + \delta _{\hat{i} \hat{j}} \, dx^{\hat{i}} \, dx^{\hat{j}} +
                        \mathcal {O}(|x^{\hat{j}}|^2) \, dx^{\hat{\alpha }} \, dx^{\hat{\beta }} .
                        \end{aligned}$$

The arrival of a gravitational wave will perturb the geodesic motion of the two particles and produce a nonzero contribution to the geodesic-deviation equation. We recall that the changes in the separation four-vector $$\varvec{{
                        n}}$$ between two geodesic trajectories with tangent four-vector $$\varvec{{
                        u}}$$ are expressed through the geodesic-deviation equation (see Fig. 1)

29 $$\begin{aligned}
                        \frac{D^2 n^{\alpha }}{D \tau ^2} = u^{\gamma }\nabla _{\gamma }\,\left(
                        u^{\beta }\nabla _{\beta } n^{\alpha }\right) = -R^{\alpha }_{\ \beta \delta
                        \gamma } u^{\beta } u^{\delta } n^{\gamma },
                        \end{aligned}$$

where the operator

30 $$\begin{aligned}
                        \frac{D}{D \tau }:=u^{\alpha } \nabla _{\alpha },
                        \end{aligned}$$

is the covariant time derivative along the worldline (in this case a geodesic) of a particle.

Indicating now with $$n^{\hat{j}}_{_\mathrm{B}} :=x^{\hat{j}}_{_\mathrm{B}} -
                        x^{\hat{j}}_{_\mathrm{A}} =
                        x^{\hat{j}}_{_\mathrm{B}}$$ the components of the separation three-vector in the positions of the two particles, the geodesic-deviation equation (29) can be written as

31 $$\begin{aligned}
                        \frac{D^2 x^{\hat{j}}_{_\mathrm{B}}}{D \tau ^2} = -R^{\hat{j}}_{\ 0 \hat{k}
                        0} x^{\hat{k}}_{_\mathrm{B}} . \end{aligned}$$

A first simplification to these equations comes from the fact that around the particle *A*, the affine connections vanish (i.e., $$\varGamma
                        ^{\hat{j}}_{{\hat{\alpha }} {\hat{\beta }}}=0$$) and the covariant derivative in (31) can be replaced by an ordinary total derivative. Furthermore, because in the TT gauge the coordinate system $$x^{\hat{\alpha
                        }}$$ moves together with the particle *A*, the proper and the coordinate time coincide at first order in the metric perturbation [i.e., $$\tau =t + \mathcal
                        {O}((h^{^\mathrm{TT}}_{\mu \nu })^2)$$]. As a result, equation (31) effectively becomes

32 $$\begin{aligned}
                        \frac{d^2 x^{\hat{j}}_{_\mathrm{B}}}{d t^2} = \frac{1}{2}\left(
                        \frac{\partial ^2 h^{^\mathrm{TT}}_{{\hat{j}} {\hat{k}}}}{\partial t^2}
                        \right) x^{\hat{k}}_{_\mathrm{B}} , \end{aligned}$$

and has solution

33 $$\begin{aligned}
                        x^{\hat{j}}_{_\mathrm{B}}(t) = x^{\hat{k}}_{_\mathrm{B}}(0) \left[ \delta
                        _{{\hat{j}} {\hat{k}}} + \frac{1}{2} h^{^\mathrm{TT}}_{{\hat{j}}
                        {\hat{k}}}(t)\right] . \end{aligned}$$

Equation (33) has a straightforward interpretation and indicates that, in the reference frame comoving with *A*, the particle *B* is seen oscillating with an amplitude proportional to $$h^{^\mathrm{TT}}_{{\hat{j}}
                        {\hat{k}}}$$.

Note that because these are transverse waves, they will produce a local deformation of the spacetime only in the plane orthogonal to their direction of propagation. As a result, if the two particles lay along the direction of propagation (i.e., if $${\varvec{n}} \parallel
                        {\varvec{\kappa }}$$), then $$h^{^\mathrm{TT}}_{{\hat{j}} {\hat{k}}}
                        x^{\hat{j}}_{_\mathrm{B}}(0) \propto h^{^\mathrm{TT}}_{{\hat{j}} {\hat{k}}}
                        \kappa ^{\hat{j}}_{_\mathrm{B}}(0) = 0$$ and no oscillation will be recorded by *A* [cf. Eq. (22)].

Let us now consider a concrete example and in particular a planar gravitational wave propagating in the positive *z*-direction. In this case

34 $$\begin{aligned}
                        h^{^\mathrm{TT}}_{xx}= & {} - h^{^\mathrm{TT}}_{yy} = {\mathfrak
                        {R}\left\{ A_{+} \exp [-i\omega (t-z)]\right\} } ,
                        \end{aligned}$$

35 $$\begin{aligned}
                        h^{^\mathrm{TT}}_{xy}= & {} h^{^\mathrm{TT}}_{yx} = {\mathfrak
                        {R}\left\{ A_{\times } \exp [-i\omega (t-z)]\right\} },
                        \end{aligned}$$

where $$A_{+}$$, $$A_{\times
                        }$$ represent the two independent modes of polarization, and the symbol $$\mathfrak
                        {R}$$ refers to the real part. As in classical electromagnetism, in fact, it is possible to decompose a gravitational wave in two *linearly* polarized plane waves or in two *circularly* polarized ones. In the first case, and for a gravitational wave propagating in the *z*-direction, the polarization *tensors* $$+$$ (“plus”) and $$\times
                        $$ (“cross”) are defined as

36 $$\begin{aligned}
                        \varvec{e}_{+}:= & {} {\varvec{e}}_{x} \otimes {\varvec{e}}_{x} -
                        {\varvec{e}}_{y} \otimes {\varvec{e}}_{y} ,
                        \end{aligned}$$

37 $$\begin{aligned}
                        \varvec{e}_{\times }:= & {} {\varvec{e}}_{x} \otimes
                        {\varvec{e}}_{x} + {\varvec{e}}_{y} \otimes {\varvec{e}}_{y} .
                        \end{aligned}$$

**Fig. 2:**
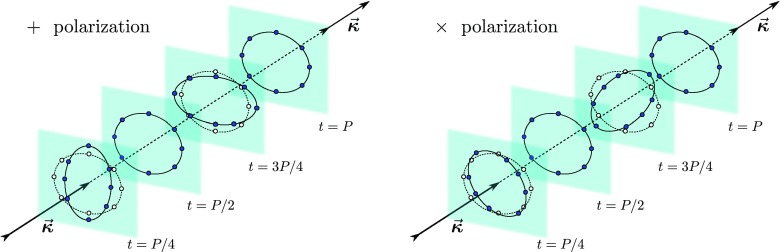
Schematic deformations produced on a ring of freely-falling particles by gravitational waves that are linear polarized in the “$$+$$” (“plus”) and “$$\times
                                 $$” (“cross”) modes. The *continuous lines* and the *dark filled dots* show the positions of the particles at different times, while the *dashed lines* and the *open dots* show the unperturbed positions

The deformations that are associated with these two modes of linear polarization are shown in Fig. 2 where the positions of a ring of freely-falling particles are schematically represented at different fractions of an oscillation period. Note that the two linear polarization modes are simply rotated of $$\pi
                        /4$$.

**Fig. 3:**
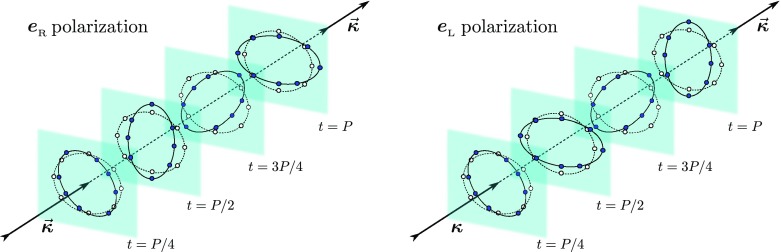
Schematic deformations produced on a ring of freely-falling particles by gravitational waves that are circularly polarized in the *R* (clockwise) and *L* (counter-clockwise) modes. The *continuous lines* and the *dark filled dots* show the positions of the particles at different times, while the *dashed lines* and the *open dots* show the unperturbed positions

In a similar way, it is possible to define two *tensors* describing the two states of circular polarization and indicate with $\varvec{e}_{_\mathrm{R}}$ the circular polarization that rotates clockwise (see Fig. 3)

38 $$\begin{aligned}
                        \varvec{e}_{_\mathrm{R}} :=\frac{\varvec{e}_{+} + i \varvec{e}_{\times
                        }}{\sqrt{2}} , \end{aligned}$$

and with $\varvec{e}_{_\mathrm{L}}$ the circular polarization that rotates counter-clockwise (see Fig. 3)

39 $$\begin{aligned}
                        \varvec{e}_{_\mathrm{L}} :=\frac{\varvec{e}_{+} - i \varvec{e}_{\times
                        }}{\sqrt{2}} . \end{aligned}$$

The deformations that are associated to these two modes of circular polarization are shown in Fig. 3.

### The quadrupole formula

The quadrupole formula and its domain of applicability were mentioned in Sect. 1, and some examples of its use in a numerical simulation are presented in Sect. 8. In practice, the quadrupole formula represents a low-velocity, weak-field approximation to measure the gravitational-wave emission within a purely Newtonian description of gravity.^3^ In practice, the formula is employed in those numerical simulations that either treat gravity in an approximate manner (e.g., via a post-Newtonian approximation or a conformally flat metric) or that, although in full general relativity, have computational domains that are too small for an accurate calculation of the radiative emission.

In what follows we briefly discuss the amounts of energy carried by gravitational waves and provide simple expressions to estimate the gravitational radiation luminosity of potential sources. Although the estimates made here come from analogies with electromagnetism, they provide a reasonable approximation to more accurate expressions from which they differ for factors of a few. Note also that while obtaining such a level of accuracy requires only a small effort, reaching the accuracy required of a template to be used in the realistic detection of gravitational waves is far more difficult and often imposes the use of numerical relativity calculations on modern supercomputers.

In classical electrodynamics, the energy emitted per unit time by an oscillating electric dipole $$d =
                        qx$$, with *q* the electrical charges and *x* their separation, is easily estimated to be

40 $$\begin{aligned}
                        L_\mathrm{electric\ dip.} :=\frac{(\mathrm{energy\,
                        emitted})}{(\mathrm{unit\, time})} =\frac{2}{3}q^2 (\ddot{x}\,)^2
                        =\frac{2}{3}(\ddot{d}\,)^2 , \end{aligned}$$

where the number of “dots” counts the order of the total time derivative. Equally simple is to calculate the corresponding luminosity in gravitational waves produced by an oscillating mass dipole. In the case of a system of *N* point-like particles of mass $$m_{_{A}}$$ ($$A=1,2,\ldots
                        ,N$$), in fact, the total mass dipole and its first time derivative are

41 $$\begin{aligned}
                        {\varvec{d}} :=\sum ^N_{A=1} m_{_{A}} {\varvec{x}}_{_{A}} ,
                        \end{aligned}$$

and

42 $$\begin{aligned}
                        \dot{{\varvec{d}}} :=\sum ^N_{A=1} m_{_{A}} \dot{{\varvec{x}}}_{_{A}} =
                        {\varvec{p}}, \end{aligned}$$

respectively. However, the requirement that the system conserves its total linear momentum

43 $$\begin{aligned}
                        \ddot{{\varvec{d}}} :=\dot{{\varvec{p}}}=0,
                        \end{aligned}$$

forces to conclude that $$L_\mathrm{mass\
                        dip.}=0$$, i.e., that there is no mass-dipole radiation in general relativity (This is equivalent to the impossibility of having electromagnetic radiation from an electric monopole oscillating in time.). Next, consider the electromagnetic energy emission produced by an oscillating electric quadrupole. In classical electrodynamics, this energy loss is given by

44 $$\begin{aligned}
                        L_\mathrm{electric\ quad.} :=\frac{1}{20}({\dddot{Q}})^2 =\frac{1}{20}
                        ({\dddot{Q}_{jk}}{\dddot{Q}_{jk}}) ,
                        \end{aligned}$$

where

45 $$\begin{aligned} Q_{jk}
                        :=\sum ^N_{A=1} q_{_{A}} \left[ (x_{_{A}})_j (x_{_{A}})_k -
                        \frac{1}{3}\delta _{jk} (x_{_{A}})_i (x_{_{A}})^i\right] ,
                        \end{aligned}$$

is the electric quadrupole for a distribution of *N* charges $$(q_1, q_2, \ldots ,
                        q_N)$$.

In close analogy with expression (44), the energy loss per unit time due to an oscillating mass quadrupole is calculated to be46
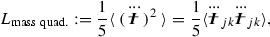
where 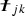 is the trace-less mass quadrupole (or “reduced” mass quadrupole), defined as47
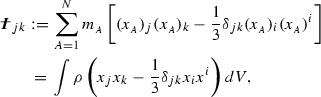
and the brackets $$\langle ~ \rangle
                        $$ indicate a time average [Clearly, the second expression in (47) refers to a continuous distribution of particles with rest-mass density $$\rho
                        $$.].

A crude estimate of the third time derivative of the mass quadrupole of the system is given by48
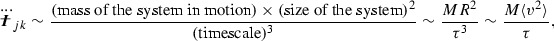
where $$\langle v \rangle
                        $$ is the mean internal velocity. Stated differently,49
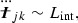
where $$L_\mathrm{int}$$ is the power of the system flowing from one part of the system to the other.

As a result, the gravitational-wave luminosity in the quadrupole approximation can be calculated to be (we here restore the explicit use of the gravitational constant and of the speed of light)

50 $$\begin{aligned}
                        L_\mathrm{mass-quad} \sim \left( \frac{G}{c^5}\right) \left( \frac{M \langle
                        v^2 \rangle }{\tau } \right) ^2\ \sim \left( \frac{G^4}{c^5}\right) \left(
                        \frac{M}{R} \right) ^5 \sim \left( \frac{c^5}{G}\right) \left(
                        \frac{R_{_\mathrm{S}}}{R}\right) ^2 \left( \frac{\langle v^2\rangle }{c^2}
                        \right) ^3 . \end{aligned}$$

The second equality has been derived using the virial theorem for which the kinetic energy is of the same order of the potential one, i.e., $$M \langle v^2 \rangle
                        \sim G M^2/R$$, and assuming that the oscillation timescale is inversely proportional to the mean stellar density, i.e., $$\tau \sim (1/G\langle
                        \rho \rangle )^{1/2} \sim (R^3/GM)^{1/2}$$. Similarly, the third equality expresses the luminosity in terms of dimensionless quantities such as the size of the source relative to the Schwarzschild radius $$R_{_\mathrm{S}} =
                        2GM/c^2$$, and the source speed in units of the speed of light. Note that the quantity $${c^5}{G}$$ has indeed the units of a luminosity, i.e., $$\mathrm{erg\ s^{-1}\ =\
                        cm^2\ g\ s^{-3}}$$ in cgs units.

Although extremely simplified, expressions (48) and (50) contain the two most important pieces of information about the generation of gravitational waves. The first one is that the conversion of any type of energy into gravitational waves is, in general, not efficient. To see this it is necessary to bear in mind that expression (46) is in geometrized units and that the conversion to conventional units, say cgs units, requires dividing (46) by the very large factor $$c^5/G \simeq 3.63 \times
                        10^{59}\ \mathrm{erg\ s}^{-1}$$. The second one is contained in the last expression in Eq. (50) and that highlights how the gravitational-wave luminosity can also be extremely large. There are in fact astrophysical situations, such as those right before the merger of a binary system of compact objects, in which $$\sqrt{\langle v^2\rangle
                        } \sim 0.1\,c$$ and $$R \sim
                        10\,R_{_\mathrm{S}}$$, so that $$L_\mathrm{mass-quad}
                        \sim 10^{51}\ \mathrm{erg\ s}^{-1} \sim 10^{18}\, L_{\odot
                        }$$, that is, $$10^{18}$$ times the luminosity of the Sun; this is surely an impressive release of energy.

#### Extensions of the quadrupole formula

Although valid only in the low-velocity, weak-field limit, the quadrupole-formula approximation has been used extensively in the past and still finds use in several simulations, ranging from stellar-core collapse (see, e.g., Zwerger and Müller 1997 for some initial application) to binary neutron-star mergers (see, e.g., Oechslin et al. 2002 for some initial application). In many of the simulations carried out to study stellar collapse, one makes the additional assumption that the system remains axisymmetric and the presence of an azimuthal Killing vector has two important consequences. Firstly, the gravitational waves produced in this case will carry away energy but not angular momentum, which is a conserved quantity in this spacetime. Secondly, the gravitational waves produced will have a single polarization state, so that the transverse traceless gravitational field is completely determined in terms of its only nonzero transverse and traceless (TT) independent component. Following Zwerger and Müller (1997) and considering for simplicity an axisymmetric system, it is useful to express the gravitational strain $h^{^\mathrm{TT}}(t)$ observed at a distance *R* from the source in terms of the quadrupole wave amplitude $A_{20}$ (Zanotti et al. 2003)

51 $$\begin{aligned}
                           h^{^\mathrm{TT}}(t) = F_+ \left( \frac{1}{8} \sqrt{\frac{15}{\pi
                           }}\right) \frac{A_{20}(t-R)}{R} ,
                           \end{aligned}$$

where $$F_+=F_+(R,\theta
                           ,\phi )$$ is the detector’s beam pattern function and depends on the orientation of the source with respect to the observer. As customary in these calculations, we will assume it to be optimal, i.e., $$F_+=1$$. The $$\ell =2,
                           m=0$$ wave amplitude $$A_{20}$$ in Eq. (51) is simply the second time derivative of the reduced mass quadrupole moment in axisymmetry and can effectively be calculated without taking time derivatives numerically, which are instead replaced by spatial derivatives of evolved quantities after exploiting the continuity and the Euler equations (Finn and Evans 1990; Blanchet et al. 1990; Rezzolla et al. 1999b). The result in a spherical coordinate system is

52 $$\begin{aligned}
                           A_{20} :=\frac{d^2 I_{[ax]}}{dt^2} =&\, k \int \rho \biggl [v_r
                           v^r (3 z^2 - 1) + v_\theta v^\theta (2 - 3 z^2) - v_\phi v^\phi \nonumber
                           \\&-6 z \sqrt{(v^r v_r)(v_\theta v^\theta ) (1 - z^2)} \biggl . -
                           r \frac{\partial \Phi }{\partial r} (3 z^2 - 1) \nonumber \\&+ 3
                           z\frac{\partial \Phi }{\partial \theta } \sqrt{1 - z^2}\biggr ] r^2 \, dr
                           \, dz , \end{aligned}$$

where $$z:=\cos \theta
                           $$, $$k = 16 \pi
                           ^{3/2}/\sqrt{15} $$, $$\Phi
                           $$ is the Newtonian gravitational potential, and $$I_{[ax]}$$ is the appropriate component of the Newtonian reduced mass-quadrupole moment in axisymmetry

53 $$\begin{aligned}
                           I_{[ax]}:=\int \rho \left( \frac{3}{2}z^2 - \frac{1}{2}\right) r^4 \, dr
                           \, dz . \end{aligned}$$

Of course it is possible to consider more generic conditions and derive expressions for the strain coming from more realistic sources, such as a an astrophysical system with equatorial symmetry. In this case, focussing on the lowest $$\ell
                           =2$$ moments, the relevant multipolar components for the strain are Baiotti et al. (2009)

54 $$\begin{aligned}
                           h^{20}&=\dfrac{1}{r}\sqrt{\dfrac{24\pi }{5}}\left( \ddot{\mathcal
                           {I}}_{zz} - \dfrac{1}{3}\mathrm{Tr}{(\ddot{\mathcal {I}})}\right) ,
                           \end{aligned}$$

55 $$\begin{aligned}
                           h^{21}&=-\dfrac{\i }{r}\sqrt{\dfrac{128\pi }{45}}\left(
                           \ddot{\mathcal {J}}_{xz} - i \ddot{\mathcal {J}}_{yz}\right) ,
                           \end{aligned}$$

56 $$\begin{aligned}
                           h^{22}&=\dfrac{1}{r}\sqrt{\dfrac{4\pi }{5}}\left( \ddot{\mathcal
                           {I}}_{xx} - 2 i\ddot{\mathcal {I}}_{xy}-\ddot{\mathcal {I}}_{yy}\right) ,
                           \end{aligned}$$

where

57 $$\begin{aligned}
                           \mathcal {I}_{ij}&= \int d^3 x \,\rho \, x_i x_j,
                           \end{aligned}$$

58 $$\begin{aligned}
                           \mathcal {J}_{ij}&= \int d^3x \,\rho \,\epsilon _{abi}\,x_j x_a\,
                           v^b, \end{aligned}$$

are the more general Newtonian mass and mass-current quadrupoles.

Expressions (52)–(58) are strictly Newtonian. Yet, these expression are often implemented in numerical codes that are either fully general relativistic or exploit some level of general-relativistic approximation. More seriously, these expressions completely ignore considerations that emerge in a relativistic context, such as the significance of the coordinate chosen for their calculation. As a way to resolve these inconsistencies, improvements to these expressions have been made to increase the accuracy of the computed gravitational-wave emission. For instance, for calculations on known spacetime metrics, the gravitational potential in expression (52) is often approximated with expressions derived from the metric, e.g., as $$\Phi = (1 -
                           g_{rr})/2$$ (Zanotti et al. 2003), which is correct to the first Post-Newtonian (PN) order. Improvements to the mass quadrupole (53) inspired by a similar spirit have been computed in Blanchet et al. (1990), and further refined and tested in Shibata and Sekiguchi (2004), Nagar et al. (2005), Cerdá-Durán et al. (2005), Pazos et al. (2007), Baiotti et al. (2007), Dimmelmeier et al. (2007), Corvino et al. (2010).

A systematic comparison among the different expressions of the quadrupole formulas developed over the years was carried out in Baiotti et al. (2009), where a generalization of the mass-quadrupole formula (57) was introduced. In essence, following previous work in Nagar et al. (2005), Baiotti et al. (2009) introduced a “generalized” mass-quadrupole moment of the form

59 $$\begin{aligned}
                           \mathcal {I}_{ij}[\varrho ] :=\int d^3 x \varrho x_i x_j,
                           \end{aligned}$$

where the generalized rest-mass density $$\varrho
                           $$ was assumed to take a number of possible expressions, namely,

60 $$\begin{aligned}&\varrho :=\rho ,
                           \end{aligned}$$

61 $$\begin{aligned}&\varrho :=\alpha ^2\sqrt{\gamma
                           }T^{00}, \end{aligned}$$

62 $$\begin{aligned}&\varrho :=\sqrt{\gamma }W\rho ,
                           \end{aligned}$$

63 $$\begin{aligned}&\varrho :=u^0\rho
                           =\frac{W}{\alpha }\rho , \end{aligned}$$

Clearly, the first option corresponds to the “standard” quadrupole formula, but, as remarked in Baiotti et al. (2009) none of the alternative quadrupole formulas obtained using these generalized quadrupole moments should be considered better than the others, at least mathematically. None of them is gauge invariant and indeed they yield different results depending on the underlining choice made for the coordinates. Yet, the comparison is meaningful in that these expressions were and still are in use in many numerical codes, and it is therefore useful to determine which expression is effectively closer to the fully general-relativistic one.

Making use of a fully general-relativistic measurement of the gravitational-wave emission from a neutron star oscillating nonradially as a result of an initial pressure perturbation, Baiotti et al. (2009) concluded that the various quadrupole formulas are comparable and give a very good approximation to the phasing of the gravitational-wave signals. At the same time, they also suffer from systematic over-estimate [expression (61)] or under-estimates of the gravitational-wave amplitude [expressions (60), and (62)–(63)]. In all cases, however, the relative difference in amplitude was of 50 % at most, which is probably acceptable given that these formulas are usually employed in complex astrophysical calculations in which the systematic errors coming from the microphysical modelling are often much larger.

## Basic numerical approaches

### The 3+1 decomposition of spacetime

At the heart of Einstein’s theory of general relativity is the equivalence among all coordinates, so that the distinction of spatial and time coordinates is more an organisational matter than a requirement of the theory. Despite this “covariant view”, however, our experience, and the laws of physics on sufficiently large scales, do suggest that a distinction of the time coordinate from the spatial ones is the most natural one in describing physical processes. Furthermore, while not strictly necessary, such a distinction of time and space is the simplest way to exploit a large literature on the numerical solution of hyperbolic partial differential equations as those of relativistic hydrodynamics. In a generic spacetime, analytic solutions to the Einstein equations are not known, and a numerical approach is often the only way to obtain an estimate of the solution.

Following this principle, a decomposition of spacetime into “space” and “time” was already proposed in the 1960s within a Hamiltonian formulation of general relativity and later as an aid to the numerical solution of the Einstein equations in vacuum. The basic idea is rather simple and consists in “foliating” spacetime in terms of a set of non-intersecting spacelike hypersurfaces $$\varSigma :=\varSigma
                        (t)$$, each of which is parameterised by a constant value of the coordinate *t*. In this way, the three spatial coordinates are split from the one temporal coordinate and the resulting construction is called the 3+1 *decomposition* of spacetime (Misner et al. 1973).

**Fig. 4:**
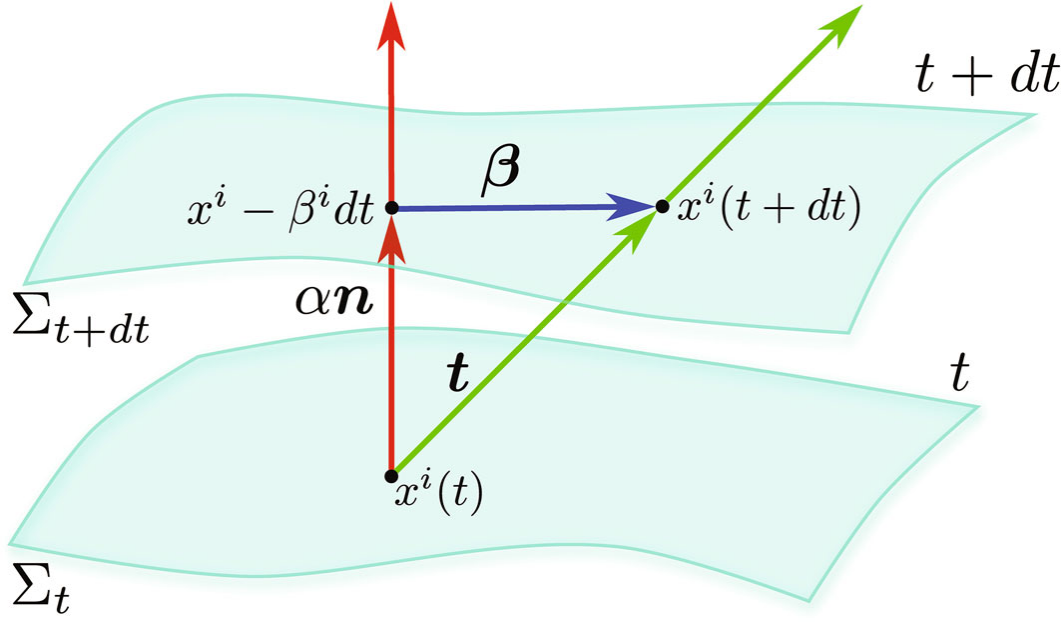
Schematic representation of the 3+1 decomposition of spacetime with hypersurfaces of constant time coordinate $$\varSigma
                                 _t$$ and $$\varSigma
                                 _{t+dt}$$ foliating the spacetime. The four-vector $$\varvec{t}$$ represents the direction of evolution of the time coordinate *t* and can be split into a timelike component $$\alpha
                                 \varvec{n}$$, where $$\varvec{n}$$ is a timelike unit normal to the hypersurface, and into a spacelike component, represented by the spacelike four-vector $$\varvec{\beta
                                 }$$. The function $$\alpha
                                 $$ is the “lapse” and measures the proper time between adjacent hypersurfaces, while the components of the “shift” vector $$\beta
                                 ^{i}$$ measure the change of coordinates from one hypersurface to the subsequent one

Given one such constant-time hypersurface, $$\varSigma
                        _t$$, belonging to the *foliation* $$\varSigma
                        $$, we can introduce a timelike four-vector $$\varvec{n}$$ normal to the hypersurface at each event in the spacetime and such that its dual one-form $$\varvec{\varOmega }
                        :=\varvec{\nabla } t$$ is parallel to the gradient of the coordinate *t*, i.e., 

64 $$\begin{aligned} n_\mu =A
                        \varOmega _{\mu } = A \nabla _\mu t ,
                        \end{aligned}$$

with $$n_{\mu } = \{A,0,0,0
                        \}$$ and *A* a constant to be determined. If we now require that the four-vector $$\varvec{n}$$ defines an observer and thus that it measures the corresponding four-velocity, then from the normalisation condition on timelike four-vectors, $$n^\mu n_\mu
                        =-1$$, we find that

65 $$\begin{aligned} n^\mu
                        n_\mu =g^{\mu \nu }n_\mu n_\nu = g^{tt}A^2=-\frac{1}{\alpha ^2}A^2=-1 ,
                        \end{aligned}$$

where we have defined $$\alpha ^2 :=-
                        1/g^{tt}$$. From the last equality in expression (65) it follows that $$A=\pm \alpha
                        $$ and we will select $$A=-\alpha
                        $$, such that the associated vector field $$n^\mu
                        $$ is future directed. The quantity $$\alpha
                        $$ is commonly referred to as the *lapse* function, it measures the rate of change of the coordinate time along the vector $$n^\mu
                        $$ (see Fig. 4), and will be a building block of the metric in a 3+1 decomposition [cf. Eq. (72)].

The specification of the normal vector $\varvec{n}$ allows us to define the metric associated to each hypersurface, i.e., 

66 $$\begin{aligned} \gamma
                        _{\mu \nu } :=g_{\mu \nu } + n_{\mu } n_{\nu },\quad \gamma ^{\mu \nu }
                        :=g^{\mu \nu } + n^{\mu } n^{\nu }, \end{aligned}$$

where $$\gamma ^{0\mu
                        }=0$$, $$\gamma _{ij} =
                        g_{ij}$$, but in general $$\gamma ^{ij} \ne
                        g^{ij}$$. Also note that $$\gamma ^{ik} \gamma
                        _{kj}= \delta ^i_j$$, that is, $$\gamma
                        ^{ij}$$ and $$\gamma
                        _{ij}$$ are the inverse of each other, and so can be used for raising and lowering the indices of purely spatial vectors and tensors (that is, defined on the hypersurface $$\varSigma
                        _t$$).

The tensors $$\varvec{n}$$ and $$\varvec{\gamma
                        }$$ provide us with two useful tools to decompose any four-dimensional tensor into a purely spatial part (hence contained in the hypersurface $$\varSigma
                        _t$$) and a purely timelike part (hence orthogonal to $$\varSigma
                        _t$$ and aligned with $$\varvec{n}$$). Not surprisingly, the spatial part is readily obtained after contracting with the *spatial projection operator* (or *spatial projection tensor*)

67 $$\begin{aligned} \gamma
                        ^{\mu }_{\ \,\nu } :=g^{\mu \alpha } \gamma _{\alpha \nu } = g^{\mu }_{\
                        \,\nu } + n^{\mu } n_{\nu } = \delta ^{\mu }_{\ \,\nu } + n^{\mu } n_{\nu },
                        \end{aligned}$$

while the timelike part is obtained after contracting with the *time projection operator* (or *time projection tensor*)

68 $$\begin{aligned} N^{\mu
                        }_{\ \,\nu } :=- n^{\mu } n_{\nu }, \end{aligned}$$

and where the two projectors are obviously orthogonal, i.e., 

69 $$\begin{aligned} \gamma
                        ^{\alpha }_{\ \,\mu } N^{\mu }_{\ \nu } = 0.
                        \end{aligned}$$

We can now introduce a new vector, $$\varvec{t}$$, along which to carry out the time evolutions and that is dual to the surface one-form $$\varvec{\varOmega
                        }$$. Such a vector is just the time-coordinate basis vector and is defined as the linear superposition of a purely temporal part (parallel to $$\varvec{n}$$) and of a purely spatial one (orthogonal to $$\varvec{n}$$), namely

70 $$\begin{aligned}
                        \varvec{t} = \varvec{e}_t = \partial _t :=\alpha \varvec{n} + \varvec{\beta
                        }. \end{aligned}$$

The purely spatial vector $$\varvec{\beta
                        }$$ [i.e., $$\beta ^{\mu } = (0,\beta
                        ^i)$$] is usually referred to as the *shift vector* and will be another building block of the metric in a 3+1 decomposition [cf. Eq. (72)]. The decomposition of the vector $$\varvec{t}$$ into a timelike component $$n\varvec{\alpha
                        }$$ and a spatial component $$\varvec{\beta
                        }$$ is shown in Fig. 4.

Because $$\varvec{t}$$ is a coordinate basis vector, the integral curves of $$t^{\mu
                        }$$ are naturally parameterised by the time coordinate. As a result, all infinitesimal vectors $$t^{\mu
                        }$$ originating at a given point $$x_0^i$$ on one hypersurface $$\varSigma
                        _t$$ would end up on the hypersurface $$\varSigma
                        _{t+dt}$$ at a point whose coordinates are also $$x_0^i$$. This condition is not guaranteed for translations along $$\varOmega _{\mu
                        }$$ unless $$\beta ^\mu
                        =0$$ since $$t^{\mu }t_{\mu } =
                        g_{tt}= -\alpha ^2 + \beta ^{\mu }\beta _{\mu }$$, and as illustrated in Fig. 4.

In summary, the components of $\varvec{n}$ are given by

71 $$\begin{aligned} n_\mu =
                        \left( -\alpha ,0,0,0 \right) , \quad n^\mu =\frac{1}{\alpha }\left(
                        1,-\beta ^i \right) , \end{aligned}$$

and we are now ready to deduce that the lapse function and the shift vector can be employed to express the generic *line element* in a 3+1 decomposition as

72 $$\begin{aligned} ds^{2} =
                        -(\alpha ^{2}-\beta _{i}\beta ^{i}) \, dt^{2}+ 2 \beta _{i} \, dx^{i} \, dt
                        + \gamma _{ij} \, dx^{i} \, dx^{j} .
                        \end{aligned}$$

Expression (72) clearly emphasises that when $$\beta
                        ^i=0=dx^i$$, the lapse measures the proper time, $$d\tau ^2 =
                        -ds^2$$, between two adjacent hypersurfaces, i.e., 

73 $$\begin{aligned} d\tau
                        ^{2} = \alpha ^{2}(t,x^j) \, dt^{2} ,
                        \end{aligned}$$

while the shift vector measures the change of coordinates of a point that is moved along $$\varvec{n}$$ from the hypersurface $$\varSigma
                        _t$$ to the hypersurface $$\varSigma
                        _{t+dt}$$, i.e., 

74 $$\begin{aligned}
                        x^i_{t+dt}=x^i_{t}-\beta ^i (t,x^j) \, dt .
                        \end{aligned}$$

Similarly, the covariant and contravariant components of the metric (72) can be written explicitly as

75 $$\begin{aligned}&g_{\mu \nu } = \left(
                        \begin{array}{cc} -\alpha ^2 + \beta _i \beta ^i ~~&{}~~ \beta _i \\
                        ~~&{}~~\\ \beta _i ~~&{}~~ \gamma _{ij} \\ \end{array}
                        \right) ,&g^{\mu \nu } = \left( \begin{array}{cc} -1/\alpha ^2
                        &{} \beta ^i/\alpha ^2 \\ &{}\\ \beta ^i/\alpha ^2
                        &{} \gamma ^{ij} - \beta ^i \beta ^j/ \alpha ^2 \\ \end{array}
                        \right) , \end{aligned}$$

from which it is easy to obtain an important identity which will be used extensively hereafter, i.e., 

76 $$\begin{aligned}
                        \sqrt{-g} = \alpha \sqrt{\gamma }, \end{aligned}$$

where $$g :=\det (g_{\mu \nu
                        })$$ and $$\gamma :=\det (\gamma
                        _{ij})$$.

When defining the unit timelike normal $$\varvec{n}$$ in Eq. (65), we have mentioned that it can be associated to the four-velocity of a special class of observers, which are referred to as *normal* or *Eulerian observers*. Although this denomination is somewhat confusing, since such observers are not at rest with respect to infinity but have a coordinate velocity $$dx^i/dt = n^i =-\beta
                        ^i/\alpha $$, we will adopt this traditional nomenclature also in the following and thus take an “Eulerian observer” as one with four-velocity given by (71).

When considering a fluid with four-velocity $$\varvec{u}$$, the spatial four-velocity $$\varvec{v}$$ measured by an Eulerian observer will be given by the ratio between the projection of $$\varvec{u}$$ in the space orthogonal to $$\varvec{n}$$, i.e., $$\gamma ^i_{\ \,\mu }
                        u^{\mu } = u^i$$, and the *Lorentz factor* *W* of $$\varvec{u}$$ as measured by $$\varvec{n}$$ (de Felice and Clarke 1990)

77 $$\begin{aligned} -n_{\mu
                        } u^{\mu } = \alpha u^t = W. \end{aligned}$$

As a result, the spatial four-velocity of a fluid as measured by an Eulerian observer will be given by

78 $$\begin{aligned}
                        \varvec{v} :=\frac{\varvec{\gamma } \cdot \varvec{u}}{-\varvec{n}\cdot
                        \varvec{u}}. \end{aligned}$$

Using now the normalisation condition $$u^{\mu }u_{\mu
                        }=-1$$, we obtain

79 $$\begin{aligned} \alpha
                        u^t = -\varvec{n}\cdot \varvec{u} = \frac{1}{\sqrt{1 - v^iv_i}} = W,\quad
                        u_t = W(-\alpha +\beta _i v^i) , \end{aligned}$$

so that the components of $\varvec{v}$ can be written as

80 $$\begin{aligned} v^i =
                        \frac{u^i}{W} + \frac{\beta ^i}{\alpha } = \frac{1}{\alpha }\left(
                        \frac{u^i}{u^t} + \beta ^i\right) , \quad v_i = \frac{u_i}{W} =
                        \frac{u_i}{\alpha u^t} , \end{aligned}$$

where in the last equality we have exploited the fact that $$\gamma _{ij}u^j = u_i -
                        \beta _i W/\alpha $$.

### The ADM formalism: 3+1 decomposition of the Einstein equations

The 3+1 decomposition introduced in Sect. 3.1 can be used not only to decompose tensors, but also equations and, in particular, the Einstein equations, which are then cast into an initial-value form suitable to be solved numerically. A 3+1 decomposition of the Einstein equations was presented by Arnowitt et al. (2008), but it is really the reformulation suggested by York (1979) that represents what is now widely known as the *ADM formulation* (see, e.g., Alcubierre 2008; Gourgoulhon 2012 for a detailed and historical discussion). As we will see in detail later on, in this formulation the Einstein equations are written in terms of purely spatial tensors that can be integrated forward in time once some constraints are satisfied initially.

Here, we only outline the ADM formalism, and refer to the literature for the derivation and justification. Further, it is important to note that the ADM formulation is, nowadays, not used in practice because it is only weakly hyperbolic. However, the variables used in the ADM method, in particular the three-metric and the extrinsic curvature, are what will be needed later for gravitational-wave extraction, and are easily obtained from the output of other evolution methods (see discussion in Sects. 5, 7).

Instead of the ADM formalism, modern simulations mainly formulate the Einstein equations using: the BSSNOK method (Nakamura et al. 1987; Shibata and Nakamura 1995; Baumgarte and Shapiro 1999); the CCZ4 formulation (Alic et al. 2012), which was developed from the Z4 method (Bona et al. 2003, 2004; Bona and Palenzuela-Luque 2009) (see also Bernuzzi and Hilditch 2010 for the so-called Z4c formulation and Alic et al. 2013 for some comparisons); or the generalized harmonic method (Pretorius 2005) (see also Baumgarte and Shapiro 2010; Rezzolla and Zanotti 2013 for more details).

We start by noting that once a 3+1 decomposition is introduced as discussed in Sect. 3.1, it is then possible to define the *three-dimensional covariant derivative* $$D_i$$. Formally, this is done by projecting the standard covariant derivative onto the space orthogonal to $$n^\mu
                        $$, and the result is a covariant derivative defined with respect to the connection coefficients

81 $$\begin{aligned}
                        {^{{(3)}}}\!\varGamma _{j k }^{i }=\frac{1}{2} \gamma ^{i \ell }\left(
                        \partial _{j} \gamma _{k \ell }+ \partial _{k} \gamma _{\ell j}- \partial
                        _{\ell } \gamma _{j k}\right) , \end{aligned}$$

where we will use the upper left index $${^{{(3)}}}\!$$ to mark a purely spatial quantity that needs to be distinguished from its spacetime counterpart.^4^ Similarly, the three-dimensional Riemann tensor $${^{{(3)}}}\!R^{i}_{\ \,j
                        k\ell }$$ associated with $$\varvec{\gamma
                        }$$ has an explicit expression given by

82 $$\begin{aligned}
                        {^{{(3)}}}\!R^i_{\ \,jk\ell } = \partial _{k} {^{{(3)}}}\!\varGamma
                        ^i_{j\ell }- \partial _{\ell } {^{{(3)}}}\!\varGamma ^i_{jk}+
                        {^{{(3)}}}\!\varGamma ^i_{m k} {^{{(3)}}}\!\varGamma ^m_{j\ell }-
                        {^{{(3)}}}\!\varGamma ^i_{m\ell } {^{{(3)}}}\!\varGamma ^m_{jk}.
                        \end{aligned}$$

In a similar manner, the three-dimensional contractions of the three-dimensional Riemann tensor, i.e., the three-dimensional Ricci tensor and the three-dimensional Ricci scalar, are defined respectively as their four-dimensional counterparts, i.e., 

83 $$\begin{aligned}
                        {^{{(3)}}}\!R_{ij} :={^{{(3)}}}\!R^k_{\ \,ik j} , \quad {^{{(3)}}}\!R
                        :={^{{(3)}}}\!R^k_{\ \,k}. \end{aligned}$$

The information present in $$R^\mu _{\ \,\nu \kappa
                        \sigma }$$ and missing in $${^{{(3)}}}\!R^i_{\
                        \,jk\ell }$$ can be found in another symmetric tensor, the *extrinsic curvature* $$K_{ij}$$, which is purely spatial. Loosely speaking, the extrinsic curvature provides a measure of how the three-dimensional hypersurface $$\varSigma
                        _t$$ is curved with respect to the four-dimensional spacetime. For our purposes, it is convenient to define the extrinsic curvature as (but note that other definitions, which can be shown to be equivalent, are common)

84 $$\begin{aligned}
                        K_{ij}=-\frac{1}{2}\mathscr {L}_{\varvec{n}}\gamma _{ij},
                        \end{aligned}$$

where $$\mathscr
                        {L}_{\varvec{n}}$$ is the Lie derivative relative to the normal vector field $$\varvec{n}$$. Expression (84) provides a simple interpretation of the extrinsic curvature $$K_{ij}$$ as the rate of change of the three-metric $$\gamma
                        _{ij}$$ as measured by an Eulerian observer. Using properties of the Lie derivative, it follows that

85 $$\begin{aligned} \partial
                        _t \gamma _{ij} = -2\alpha K_{ij} + D_i\beta _j + D_j\beta _i .
                        \end{aligned}$$

Note that Eq. (85) is a geometrical result and is independent of the Einstein equations.

The next step is to note further purely geometric relations, how the spacetime curvature is related to the intrinsic and extrinsic curvatures of the hypersurface $$\varSigma
                        _t$$. These formulas are known as the Gauss–Codazzi equations and the Codazzi–Mainardi equations. They are

86 $$\begin{aligned} \gamma
                        ^{\mu }_{\ \,i}\, \gamma ^{\nu }_{\ \,j}\, \gamma ^{\rho }_{\ \,k}\, \gamma
                        ^{\sigma }_{\ \,\ell }\, R_{\mu \nu \rho \sigma }&=
                        {^{{(3)}}}\!R_{ijk\ell } + K_{ik}K_{j\ell } - K_{i\ell }K_{jk},
                        \end{aligned}$$

87 $$\begin{aligned} \gamma
                        ^{\rho }_{\ \,j} \gamma ^{\mu }_{\ \,i} \gamma ^{\nu }_{\ \,\ell } n^{\sigma
                        } R_{\rho \mu \nu \sigma }&= D_{i} K_{j\ell } - D_{j} K_{i\ell },
                        \end{aligned}$$

88 $$\begin{aligned} \gamma
                        ^{\alpha }_{\ \,i} \gamma ^{\beta }_{\ \,j} n^{\delta } n^{\lambda }
                        R_{\alpha \delta \beta \lambda }&= \mathscr {L}_{\varvec{n}} K_{ij}
                        - \frac{1}{\alpha }D_{i} D_{j} \alpha + K^{k}_{\ \,j} \,K_{ik}.
                        \end{aligned}$$

We now have enough identities to rewrite the Einstein equations in a 3+1 decomposition. After contraction, we can use the Einstein equations to replace the spacetime Ricci tensor with terms involving the stress-energy tensor, and then after further manipulation the final result is:

89 $$\begin{aligned} \partial
                        _t K_{ij}= & {} -D_i D_j \alpha + \beta ^k\partial _k K_{ij} +
                        K_{ik}\partial _j \beta ^k + K_{kj}\partial _i\beta ^k\nonumber \\&+
                        \alpha \left( {^{{(3)}}}\!R_{ij}+K K_{ij} - 2 K_{ik}K^{k}_{\ \,j}\right) +
                        4\pi \alpha \left[ \gamma _{ij}\left( S-E)-2S_{ij}\right) \right] ,
                        \end{aligned}$$

90 $$\begin{aligned}&{^{{(3)}}}\!R + K^2 -
                        K_{ij}K^{ij}=16\pi E, \end{aligned}$$

91 $$\begin{aligned}&D_j(K^{ij}-\gamma ^{ij}K) = 8\pi
                        S^i . \end{aligned}$$

The following definitions have been made for the “matter” quantities

92 $$\begin{aligned} S_{\mu
                        \nu } :=\gamma ^{\alpha }_{\ \,\mu } \, \gamma ^{\beta }_{\ \,\nu }
                        T_{\alpha \beta }, \qquad S_{\mu } :=-\gamma ^{\alpha }_{\ \,\mu }\, n^\beta
                        T_{\alpha \beta },\qquad S :=S^\mu _{\ \,\mu } , \;\; E :=n^\alpha \,
                        n^\beta T_{\alpha \beta } , \end{aligned}$$

that is, for contractions of the energy–momentum tensor that would obviously be zero in vacuum spacetimes.

Overall, the six equations (89), together with the six equations (85) represent the time-evolving part of the ADM equations and prescribe how the three-metric and the extrinsic curvature change from one hypersurface to the following one. In contrast, Eqs. (90) and (91) are constraints that need to be satisfied on each hypersurface. This distinction into evolution equations and constraint equations is not unique to the ADM formulation and is indeed present also in classical electromagnetism. Just as in electrodynamics the divergence of the magnetic field remains zero if the field is divergence-free at the initial time, so the constraint equations (90) and (91), by virtue of the Bianchi identities (Alcubierre 2008; Bona et al. 2009; Baumgarte and Shapiro 2010; Gourgoulhon 2012; Rezzolla and Zanotti 2013), will remain satisfied during the evolution if they are satisfied initially (Frittelli 1997). Of course, this concept is strictly true in the continuum limit, while numerically the situation is rather different. However, that issue is not pursued here.

Two remarks should be made before concluding this section. The first one is about the gauge quantities, namely, the lapse function $$\alpha
                        $$ and the shift vector $$\beta
                        ^i$$. Since they represent the four degrees of freedom of general relativity, they are not specified by the equations discussed above and indeed they can be prescribed arbitrarily, although in practice great care must be taken in deciding which prescription is the most useful. The second comment is about the mathematical properties of the time-evolution ADM equations (89) and (85). The analysis of these properties can be found, for instance, in Reula (1998) or in Frittelli and Gómez (2000), and reveals that such a system is only weakly hyperbolic with zero eigenvalues and, as such, not necessarily well-posed. The weak-hyperbolicity of the ADM equations explains why, while an historical cornerstone in the 3+1 formulation of the Einstein equations, they are rarely used in practice and have met only limited successes in multidimensional calculations (Cook et al. 1998; Abrahams et al. 1998). At the same time, the weak hyperbolicity of the ADM equations and the difficulty in obtaining stable evolutions, has motivated, and still motivates, the search for alternative formulations.

### Gravitational waves from $$\psi
                        _4$$ on a finite worldtube(s)

The Newman–Penrose scalars are scalar quantities defined as contractions between the Weyl, or conformal, tensor

93 $$\begin{aligned}
                        C_{\alpha \beta \mu \nu }=R_{\alpha \beta \mu \nu }-g_{\alpha [\mu }R_{\nu
                        ]\beta }+g_{\beta [\mu }R_{\nu ]\alpha } +\frac{g_{\alpha [\mu }g_{\nu
                        ]\beta }R}{3}, \end{aligned}$$

(in four dimensions), and an orthonormal null tetrad $$\ell ^\alpha
                        ,n_{_{[NP]}}^\alpha ,m^\alpha ,\bar{m}^\alpha $$ (Newman and Penrose 1963). The null tetrad is constructed from an orthonormal tetrad, and we use the notation $$n_{_{[NP]}}^\alpha
                        $$, rather than the usual $$n^\alpha
                        $$, because $$n_{_{[NP]}}^\alpha
                        $$ is obtained in terms of the hypersurface normal $$n^\alpha
                        $$. Supposing that the spatial coordinates (*x*, *y*, *z*) are approximately Cartesian, then spherical polar coordinates are defined using Eqs. (399) and (400). However, in general these coordinates are not exactly spherical polar, and in particular the radial coordinate is not a surface area coordinate (for which the 2-surface $$r=t=$$ constant must have area $$4\pi
                        r^2$$). We reserve the notation *r* for a surface area radial coordinate, so the radial coordinate just constructed will be denoted by *s*. Then the outward-pointing radial unit normal $$\varvec{e}_{s}$$ is

94 $$\begin{aligned} \left(
                        \varvec{e}_{s}\right) ^i=\frac{\gamma ^{ij} s_j}{\sqrt{\gamma ^{ij} s_i
                        s_j}} \qquad \hbox {where} \quad s_j=\nabla _j \sqrt{x^2+y^2+z^2}.
                        \end{aligned}$$

**Fig. 5:**
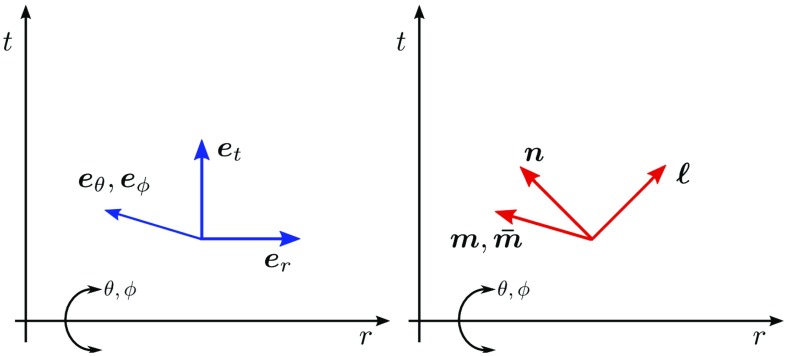
Schematic representation of an orthonormal tetrad and a null tetrad in Minkowski spacetime in spherical polar $$(t,r,\theta ,\phi )$$ coordinates. The *left panel* shows the orthonormal tetrad $$(\varvec{e}_t,\varvec{e}_r,\varvec{e}_{\theta
                                    }, \varvec{e}_{\phi })$$, and the *right panel* illustrates the null tetrad $$(\varvec{\ell },\varvec{n},
                                    \varvec{m},\bar{\varvec{m}})$$. Both $$(\varvec{e}_{\theta },\varvec{e}_{\phi
                                    })$$ and $$(\varvec{m},\bar{\varvec{m}})$$ constitute a basis of the $$(\theta ,\phi )$$ subspace; and both $$(\varvec{e}_t,\varvec{e}_r)$$ and $$(\varvec{\ell
                                    },\varvec{n})$$ constitute a basis of the (*t*, *r*) subspace

An orthonormal basis $$(\varvec{e}_{s},\varvec{e}_{\theta },\varvec{e}_{\phi
                        })$$ of $$\varSigma
                        _t$$ is obtained by Gram–Schmidt orthogonalization, and is extended to be an orthonormal tetrad of the spacetime by incuding the hypersurface normal $$\varvec{n}$$. Then the orthonormal null tetrad is

95 $$\begin{aligned}
                        \varvec{\ell }=\frac{1}{\sqrt{2}}\left( \varvec{n}+\varvec{e}_{s}\right)
                        ,\qquad \varvec{n}_{_{[NP]}}=\frac{1}{\sqrt{2}}\left(
                        \varvec{n}-\varvec{e}_{s}\right) ,\qquad \varvec{m}=\frac{1}{\sqrt{2}}\left(
                        \varvec{e}_{\theta }+i\varvec{e}_{\phi }\right) .
                        \end{aligned}$$

(The reader should be aware that some authors use different conventions, e.g., without a factor of $$\sqrt{2}$$, leading to different forms for various equations). For example, in Minkowski spacetime there is no distinction between *s* and *r*, and in spherical polar coordinates $$(t,r,\theta ,\phi
                        )$$ (Fig. 5)

96 $$\begin{aligned} \ell
                        ^\alpha =\left( \frac{1}{\sqrt{2}},\frac{1}{\sqrt{2}},0,0\right) ,\qquad
                        n_{_{[NP]}}^\alpha =\left( \frac{1}{\sqrt{2}},-\frac{1}{\sqrt{2}},0,0\right)
                        ,\qquad m^\alpha =\left( 0,0,\frac{1}{r\sqrt{2}},\frac{i}{r\sqrt{2}\sin
                        \theta }\right) . \end{aligned}$$

The null tetrad satisfies the orthonormality conditions

97 $$\begin{aligned} 0=\ell
                        ^\alpha \ell _\alpha =n_{_{[NP]}}^\alpha n_{_{[NP]}\alpha }=m^\alpha
                        m_\alpha =m^\alpha n_\alpha =m^\alpha \ell _\alpha ,\; \ell ^\alpha
                        n_{_{[NP]}\alpha } =-1,\; m^\alpha \bar{m}_\alpha =1.
                        \end{aligned}$$

The Newman–Penrose, or Weyl, scalars (Newman and Penrose 1963) are defined as

98 $$\begin{aligned} \psi
                        _0=&\,-C_{\alpha \beta \mu \nu }\ell ^\alpha m^\beta \ell ^\mu m^\nu
                        , \end{aligned}$$

99 $$\begin{aligned} \psi
                        _1=&\,-C_{\alpha \beta \mu \nu }\ell ^\alpha n_{_{[NP]}}^\beta \ell
                        ^\mu m^\nu , \end{aligned}$$

100 $$\begin{aligned} \psi
                        _2=&\,-C_{\alpha \beta \mu \nu }\ell ^\alpha m^\beta \bar{m}^\mu
                        n_{_{[NP]}}^\nu , \end{aligned}$$

101 $$\begin{aligned} \psi
                        _3=&\,-C_{\alpha \beta \mu \nu }\ell ^\alpha n_{_{[NP]}}^\beta
                        \bar{m}^\mu n_{_{[NP]}}^\nu , \end{aligned}$$

102 $$\begin{aligned} \psi
                        _4=&\,-C_{\alpha \beta \mu \nu }n_{_{[NP]}}^\alpha \bar{m}^\beta
                        n_{_{[NP]}}^\mu \bar{m}^\nu . \end{aligned}$$

For our purposes, the most important of these quantities is $$\psi
                        _4$$ since in the asymptotic limit it completely describes the outgoing gravitational radiation field: far from a source, a gravitational wave is locally plane and $$\psi
                        _4$$ is directly related to the metric perturbation in the TT gauge

103 $$\begin{aligned} \psi
                        _4={\partial ^2_t}\left( h_+ -ih_\times \right) .
                        \end{aligned}$$

In an asymptotically flat spacetime using appropriate coordinates (these issues are discussed more formally in Sect. 6), the peeling theorem (Penrose 1965a; Geroch 1977; Hinder et al. 2011) shows that $$\psi
                        _4$$ falls off as $$r^{-1}$$, and more generally that $$\psi
                        _n$$ falls off as $$r^{n-5}$$. Thus, gravitational waves are normally described not by $$\psi
                        _4$$ but by $$r\psi
                        _4$$ which should be evaluated in the limit as $$r\rightarrow \infty
                        $$ (which in practice may mean evaluated at as large a value of *r* as is feasible). Often $$\lim _{r\rightarrow
                        \infty }r\psi _4$$ is denoted by $$\psi _4^0(t,\theta ,\phi
                        )$$, but that notation will not be used in this section. These issues are discussed further in Sect. 6, but for now we will regard gravitational waves, and specifically $$r\psi
                        _4$$, as properly defined only in a spacetime whose metric can be written in a form that tends to the Minkowski metric, and for which the appropriate definition of the null tetrad is one that tends to the form Eq. (96), as $$r\rightarrow \infty
                        $$.

Equation (102) for $$\psi
                        _4$$ involves spacetime, rather than hypersurface, quantities, and this is not convenient in a “3+1” simulation. However, the expression for $$\psi
                        _4$$ can be manipulated into a form involving hypersurface quantities only (Gunnarsen et al. 1995) (there is also a derivation in the textbook (Alcubierre 2008), but note the sign difference in the definition of $$\psi
                        _4$$ used there):

104 $$\begin{aligned} \psi
                        _4=(-R_{ij}-KK_{ij}+K_{ik}K^k_j+i\epsilon _i^{k\ell }\nabla _k K_{\ell j})
                        \bar{m}^i\bar{m}^j. \end{aligned}$$

The proof is not given here, but in summary is based on using an arbitrary timelike vector, in this case the hypersurface normal $\varvec{n}$, to decompose the Weyl tensor into its “electric” and “magnetic” parts.

The above procedures lead to an estimate $$\psi
                        _4$$, but results are rarely reported in this form. Instead, $$\psi
                        _4$$ is decomposed into spin-weighted spherical harmonics (see section “The spin-weighted spherical harmonics $${}_s Y^{\ell
                        \,m}$$” in “Appendix 2”),

105 $$\begin{aligned} \psi
                        _4=\sum _{\ell \ge 2,|m|\le \ell }\psi _4^{\ell \,m}\,{}_{-2}Y^{\ell \,m}\,
                        \qquad \hbox {where}\qquad \psi _4^{\ell \, m}=\int _{S^2} \psi _4\,
                        \;{}_{-2}\bar{Y}^{\ell \,m}\, d\varOmega ,
                        \end{aligned}$$

and the $$r\psi _4^{\ell \,
                        m}$$ are evaluated and reported. Although, normally, the dominant part of a gravitational-wave signal is in the lowest modes with $$\ell =
                        2$$, the other modes are important to gravitational-wave data analysis, recoil calculations, etc.

#### Extracting gravitational waves using $$\psi
                           _4$$ on a finite worldtube

“3+1” numerical simulations are restricted to a finite domain, so it is not normally possible to calculate exactly a quantity given by an asymptotic formula (but see Sects. 6, 7). A simple estimate of $$r\psi
                           _4$$ can be obtained by constructing coordinates $$(s,\theta ,\phi
                           )$$ and an angular null tetrad vector $$\varvec{m}$$ as discussed at the beginning of Sect. 3.3. Then $$r\psi
                           _4$$ can be evaluated using Eq. (104) on a worldtube $$s=$$ constant, and the estimate is $$r\psi _4=s\psi
                           _4$$ or alternatively $$r\psi _4=\psi _4
                           \sqrt{A/4\pi }$$, where *A* is the area of the worldtube at time *t*. This approach was first used in Smarr (1977), and subsequently in, for example, Pollney et al. (2007), Pfeiffer (2007) and Scheel et al. (2009). This method does not give a unique answer, and there are many variations in the details of its implementation. However, the various estimates obtained for $$r\psi
                           _4$$ should differ by no more than $${{\mathcal
                           {O}}}(r^{-1})$$.

The quantity $$\psi
                           _4$$ has no free indices and so tensorially is a scalar, but its value does depend on the choice of tetrad. *However*, it may be shown that $$\psi
                           _4$$ is first-order tetrad-invariant if the tetrad is a small perturbation about a natural tetrad of the Kerr spacetime. This result was shown by Teukolsky (1972, 1973); see also Chandrasekhar (1978), and Campanelli et al. (2000). Briefly, the reasoning is as follows. The Kinnersley null tetrad is an exact null tetrad field in the Kerr geometry (Kinnersley 1969). It has the required asymptotic limit, and the vectors $$\ell ^\alpha
                           $$, $$n_{_{[NP]}}^\alpha
                           $$ are generators of outgoing and ingoing radial null geodesics respectively. In the Kerr geometry $$C^\mathrm{[Kerr]}_{\alpha \beta \mu \nu }\ne
                           0$$, but using the Kinnersley tetrad all $$\psi
                           _n$$ are zero except $$\psi
                           _2$$. Thus, to first-order, $$\psi
                           _4$$ is evaluated using the perturbed Weyl tensor and the background tetrad; provided terms of the form $$C^\mathrm{[Kerr]}_{\alpha \beta \mu \nu
                           }n_{_{[NP]}}^\alpha \bar{m}^\beta n_{_{[NP]}}^\mu \bar{m}^\nu
                           $$, where three of the tetrad vectors take background values and only one is perturbed, are ignorable. Allowing for those $$\psi
                           _n$$ that are zero, and using the symmetry properties of the Weyl tensor, all such terms vanish. This implies that the ambiguity in the choice of tetrad is of limited importance because it is a second-order effect; see also Campanelli and Lousto (1998); Campanelli et al. (1998). These ideas have been used to develop analytic methods for estimating $$\psi
                           _4$$ (Campanelli et al. 2000; Baker et al. 2000a; Baker and Campanelli 2000; Baker et al. 2001). Further, the Kinnersley tetrad is the staring point for a numerical extraction procedure.

In practice the spacetime being evolved is not Kerr, but in many cases at least far from the source it should be Kerr plus a small perturbation, and in the far future it should tend to Kerr. Thus an idea for an appropriate tetrad for use on a finite worldtube is to construct an approximation to the Kinnersley form, now known as the quasi-Kinnersley null tetrad (Beetle et al. 2005; Nerozzi et al. 2005). The quasi-Kinnersley tetrad has the property that as the spacetime tends to Kerr, then the quasi-Kinnersley tetrad tends to the Kinnersley tetrad. The method was used in a number of applications in the mid-2000s (Nerozzi et al. 2006; Campanelli et al. 2006; Fiske et al. 2005; Nerozzi 2007).

Despite the mathematical attraction of the quasi-Kinnersley approach, nowadays the extrapolation method which assumes the simpler Schwarzschild background (see next section) is preferred since, at a practical level, and as discussed in Sect. 8, extrapolation can give highly accurate results. Modern simulations typically extract on a worldtube at between 100 and 1000 M where the correction due to the background being Kerr rather than Schwarzschild is negligible. More precisely, an invariant measure of curvature is the square root of the Kretschmann scalar, which for the Kerr geometry (Henry 2000) takes the asymptotic form

106 $$\begin{aligned}
                           \sqrt{R^\mathrm{[Kerr]}_{\alpha \beta \mu \nu
                           }\,R_\mathrm{[Kerr]}^{\alpha \beta \mu \nu
                           }}=4\sqrt{3}\frac{M}{r^3}\left( 1-\frac{21a^2\cos ^2\theta
                           }{2r^2}+{{\mathcal {O}}}\left( \frac{a^4}{r^4}\right) \right) ,
                           \end{aligned}$$

where $a:=J/M^2$. The curvature is already small in the Schwarzschild ($a=0$) case, and the effect of ignoring *a* is a small relative error of order $a^2/r^2$.

#### Extracting gravitational waves using $$\psi
                           _4$$ in practice: the extrapolation method

**Fig. 6:**
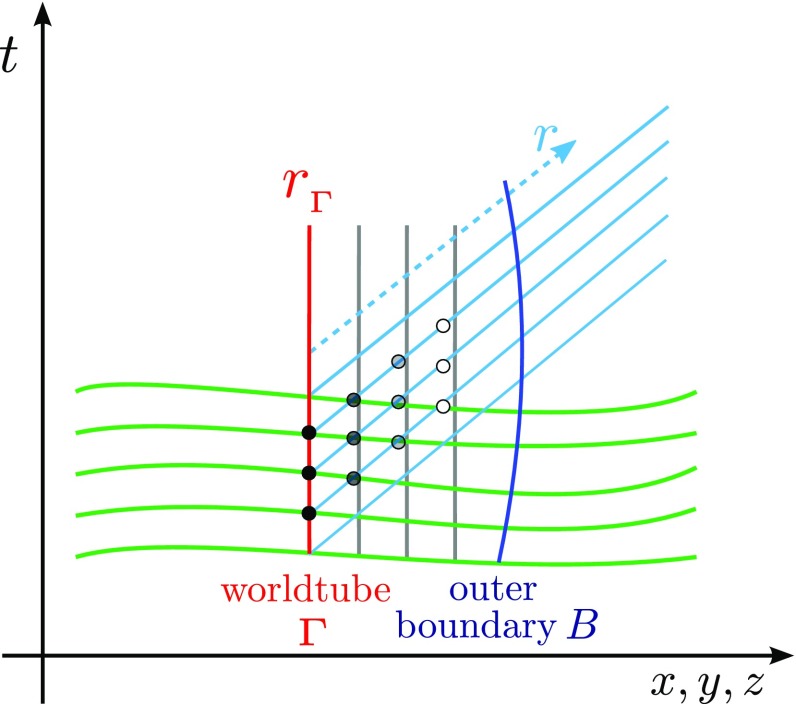
Schematic illustration of $$\psi _4$$ extrapolation. The Cauchy evolution is shown with *green slices*, with an outer boundary in *blue* subject to a boundary condition that excludes incoming gravitational waves. The *light blue lines* are approximations to outgoing null slices. $$\psi _4$$ is evaluated where the Cauchy slices meet the innermost worldtube $$r_\varGamma $$; and also at fixed values of $$r> r_\varGamma
                                    $$ on each Cauchy slice, and then interpolated onto the *black dots* shown on the null slices. Values of $$\psi _4$$ at the *black dots* on a given null slice are then extrapolated to $$r\rightarrow \infty
                                    $$

The method most commonly used at present is an adaptation of a simple estimate on a finite worldtube, and has become known as the extrapolation method. A schematic illustration of the method is given in Fig. 6. A preliminary version of extrapolation was used in 2005 (Baker et al. 2006). However, the method, as used at present, was developed in 2009 by two different groups (Pollney et al. 2009; Boyle and Mroué 2009), and a recent description is given by Taylor et al. (2013); see also Pollney et al. (2011). The essential idea is that $$\psi
                           _4$$ is estimated on worldtubes at a number of different radii, and then the data is fitted to a polynomial of form $$\psi _4=\sum _{n=1}^N
                           A_n/r^n$$ so that $$\lim _{r\rightarrow
                           \infty }r\psi _4$$ is approximated by $$A_1$$. However, there are some subtleties that complicate the procedure a little. The expected polynomial form of $$\psi
                           _4$$ is applicable only on an outgoing null cone; and further *r* should be a surface area coordinate (although often requiring this property is not important). We assume that the data available is $$\psi _4^{\ell
                           m}(t,s)$$ obtained by decomposing $$\psi
                           _4$$ into spherical harmonic components on a spherical surface of fixed coordinate radius *s* at a given coordinate time *t*. Then the first step in extrapolation is to obtain $$\psi _4^{\ell
                           m}(t_{*},r)$$, where $$t_*$$ is a retarted time coordinate specified in Eq. (108) below, and where

107 $$\begin{aligned}
                           r=r(t,s)=\sqrt{\frac{A}{4\pi }}, \end{aligned}$$

with *A* the area of the coordinate 2-surface $t=~$constant, $s=~$constant. Because the spacetime is dynamic, it would be a complicated process to construct $t_*$ exactly. Instead, it is assumed that extraction is performed in a region of spacetime in which the geometry is approximately Schwarzschild with (*t*, *s*) approximately standard Schwarzschild coordinates. Then

108 $$\begin{aligned}
                           t_{*}(t,s)=\int ^{t}_0 \frac{\sqrt{-g^{ss}(t^\prime ,s)/g^{tt}(t^\prime
                           ,s)}}{1-2 M/r(t^\prime ,s)} dt^\prime -r(t,s)-2 M \ln \left(
                           \frac{r(t,s)}{2M}-1\right) . \end{aligned}$$

In Eq. (108), *M* is an estimate of the initial mass of the system, usually the ADM mass, and $$g^{tt},g^{ss}$$ are averaged over the 2-sphere $$t^\prime
                           =~$$constant, $$s_j=~$$constant. It is straightforward to check that if (*t*, *s*) are exactly Schwarzschild coordinates, then $$t_*$$ is null.

In this way we obtain, for fixed $$t_*$$, $$\psi _4^{\ell
                           m}$$ at a number of different extraction radii; that is, we have data of the form $$\psi _4^{\ell
                           m}(t_*,r_k)$$, $$k=1,\ldots
                           ,K$$.^5^ In practice, the real and imaginary parts of $$\psi _4^{\ell
                           m}$$ may vary rapidly, and it has been found to be smoother to fit the data to the amplitude and phase.^6^ For each spherical harmonic component two data-fitting problems are solved

109 $$\begin{aligned}&|\psi _4^{\ell m}(t_*,r_k)|
                           \hbox {to} \sum _{n=1}^N \frac{A_n(t_*)}{r^n}, \nonumber \\&\arg
                           (\psi _4^{\ell m}(t_*,r_k)) \hbox {to} \sum _{n=0}^N \frac{\phi
                           _n(t_*)}{r^n}, \end{aligned}$$

using the least-squares method, and where the $$A_n, \phi
                           _n$$ are all real. Note that $$\phi
                           (t_*,r)$$ must be continuous in *r*, and so for certain values of $$r_k$$ it may be necessary to add $$\pm 2\pi
                           $$ to $$\arg (\psi _4^{\ell
                           m}(t_*,r_k))$$. Then the estimate is

110 $$\begin{aligned} \lim
                           _{r\rightarrow \infty }r\psi _4^{\ell m}= A_1 \exp (i\phi _0).
                           \end{aligned}$$

The remaining issue is the specification of *N*, and of the extraction spheres $$r_k$$ (or more precisely, since extraction is performed on spheres of specified coordinate radius, of the $$s_k$$). The key factors are the innermost and outermost extraction spheres, i.e., the values of $$r_1$$ and $$r_{_K}$$, and of course the requirement that $$K>N+1$$. Essentially, the extrapolation process uses data over the interval $$1/r\in
                           [1/r_{_K},1/r_1]$$ to construct an estimate at $$1/r=0$$. Polynomial extrapolation can be unreliable, or even divergent, as *N* is increased; it can also be unreliable when the distance from the closest data point is larger than the size of the interval over which the data is fitted. As a result of this latter condition, it is normal to require $$r_{_K}>2
                           r_1$$. On the other hand, increasing $$r_{_K}$$ increases the computational cost of a simulation, and decreasing $$r_1$$ could mean that a higher order polynomial is needed for accurate modelling of the data at that point. A compromise is needed between these conflicting factors. Values commonly used are that *N* is between 3 and 5, $$r_1$$ is normally of order 100 M, and $$r_{_K}$$ is 300 M with values as large as 1000 M reported. Typically, *K* is about 8, with the $$1/r_k$$ evenly distributed over the interval $$[1/r_{_K},1/r_1]$$.

If the desired output of a computation is a waveform (to be used, say, in the analysis of LIGO detector data), then $$\psi
                           _4$$ needs to be translated into its wave strain components $$(h_+,h_\times
                           )$$. From Eq. (103),

111 $$\begin{aligned}
                           h_+^{\ell m}(t) - i h_\times ^{\ell m}(t) = \int ^t \left( \int
                           ^{t^\prime } \psi _4^{\ell m}(t^{\prime \prime }) \, dt^{\prime \prime
                           }\right) dt^\prime + A^{\ell m} t + B^{\ell m},
                           \end{aligned}$$

where the constants of integration $$A^{\ell m}, B^{\ell
                           m}$$ need to be fixed by the imposition of some physical condition, for example that the strain should tend to zero towards the end of the computation. While this procedure is simple and straightforward, in practice it has been observed that the double time integration may lead to a reduction in accuracy, and in particular may introduce nonlinear drifts into the waveform. (The presence of a linear drift is easily corrected by means of an adjustment to the integration constants $$A^{\ell m}, B^{\ell
                           m}$$). It was shown in Reisswig and Pollney (2011) that the cause of the problem is that $$\psi _4^{\ell
                           m}$$ includes random noise, and this can lead to noticeable drifts after a double integration. The usual procedure to control the effect is via a transform to the Fourier domain. The process to construct the wave strain from $$\psi _4^{\ell
                           m}$$, without any correction for drift, is

112 $$\begin{aligned}
                           \tilde{\psi }_4^{\ell m}(\omega )&={{\mathcal {F}}}[\psi _4^{\ell
                           m}(t)] , \end{aligned}$$

113 $$\begin{aligned}
                           \tilde{h}^{\ell m}_+(\omega ) - i \tilde{h}^{\ell m}_\times (\omega
                           )&=-\frac{\tilde{\psi }_4^{\ell m}(\omega )}{\omega ^2} ,
                           \end{aligned}$$

114 $$\begin{aligned}
                           h_+^{\ell m}(t) - i h_\times ^{\ell m}(t)&= {{\mathcal
                           {F}}}^{-1}[\tilde{h}^{\ell m}_+(\omega ) - i \tilde{h}^{\ell m}_\times
                           (\omega )], \end{aligned}$$

where $${{\mathcal
                           {F}}}$$ is the Fourier transform operator, $$\omega
                           $$ denotes frequency in the Fourier domain, and $$\tilde{
                           }$$  denotes a Fourier transformed function. The division by $$\omega
                           ^2$$ in the second line of Eq. (114) is clearly potentially problematic for small $$\omega
                           $$, and an obvious strategy is to apply a filter to modify this equation. A number of such filters have been proposed, based on reducing those frequency components that are lower than $$\omega
                           _0$$—the lowest frequency expected, on physical grounds, in the waveform. The simplest choice is a step function (Campanelli et al. 2009; Aylott 2009), but it has the drawback that it leads to Gibbs phenomena. To suppress this effect, a smooth transition is needed near $$\omega
                           _0$$ and various filters have been investigated (Santamaría et al. 2010; McKechan et al. 2010; Reisswig and Pollney 2011). A particularly simple choice of filter, yet effective in many cases (Reisswig and Pollney 2011), is

115 $$\begin{aligned}
                           \tilde{h}^{\ell m}_+(\omega ) - i \tilde{h}^{\ell m}_\times (\omega )
                           =&-\frac{\tilde{\psi }_4^{\ell m}(\omega )}{\omega ^2} \quad
                           (\omega \ge \omega _0), \end{aligned}$$

116 $$\begin{aligned}
                           =&-\frac{\tilde{\psi }_4^{\ell m}(\omega )}{\omega _0^2} \quad
                           (\omega <\omega _0). \end{aligned}$$

#### Energy, momentum and angular momentum in the waves

Starting from the mass loss result of Bondi et al. (1962), the theory of energy and momentum radiated as gravitational waves was further developed in the 1960s (Penrose 1963, 1965a; Tamburino and Winicour 1966; Winicour 1968; Isaacson 1968) and subsequently (Geroch 1977; Thorne 1980a; Geroch and Winicour 1981). Formulas for the radiated angular momentum were presented in Campanelli and Lousto (1999), Lousto and Zlochower (2007) based on earlier work by Winicour (1980); formulas were also obtained in Ruiz et al. (2007), Ruiz et al. (2008) using the Isaacson effective stress-energy tensor of gravitational waves (Isaacson 1968).

The result is formulas that express the energy, momentum and angular momentum content of the gravitational radiation in terms of $$\psi
                           _4$$. Strictly, all the quantities should be evaluated in the limit as $$r\rightarrow \infty
                           $$ and using an appropriate null tetrad. The energy equation is

117 $$\begin{aligned}
                           \frac{d E}{d t}=\frac{1}{16\pi }\oint \left| \int _{-\infty }^t r\psi _4
                           \, dt^\prime \right| ^2 d\varOmega .
                           \end{aligned}$$

The linear momentum equations are

118 $$\begin{aligned}
                           \frac{d P_i}{d t}=\frac{1}{16\pi }\oint \hat{r}_i\left| \int _{-\infty
                           }^t r\psi _4 \, dt^\prime \right| ^2 d\varOmega ,
                           \end{aligned}$$

where $$\hat{r}_i$$ is a unit radial vector. If the angular coordinate system being used is spherical polars, then from Eq. (399) $$\hat{r}_i=(\sin
                           \theta \cos \phi ,\sin \theta \sin \phi ,\cos \theta
                           )$$, whereas if the coordinates are stereographic $$\hat{r}_i$$ would by given by Eqs. (405) and (406). The angular momentum equations are

119 $$\begin{aligned}
                           \frac{d J_i}{d t}=-\frac{1}{16\pi }\mathfrak {R}\left[ \oint \left( \int
                           _{-\infty }^t r\bar{\psi }_4 \, dt^\prime \right) \hat{J}_i\left( \int
                           _{-\infty }^t \int _{-\infty }^{t^\prime }r\psi _4 \, dt^\prime \,
                           dt^{\prime \prime } \right) d\varOmega \right] ,
                           \end{aligned}$$

where the $\hat{J}_i$ are operators given, in spherical polar coordinates, by

120 $$\begin{aligned}&\displaystyle \hat{J}_x = -\sin
                           \phi \partial _\theta -\cos \phi (\cot \theta \partial _\phi -is\csc
                           \theta ), \end{aligned}$$

121 $$\begin{aligned}&\displaystyle \hat{J}_y = \cos
                           \phi \partial _\theta -\sin \phi (\cot \theta \partial _\phi -is\csc
                           \theta ), \end{aligned}$$

122 $$\begin{aligned}&\displaystyle \hat{J}_z =
                           \partial _\phi . \end{aligned}$$

In practice, the above formulas are rarely used directly, and instead $$\psi
                           _4$$ is first decomposed into spin-weighted spherical harmonics using Eq. (105). Then the energy equation is

123 $$\begin{aligned}
                           \frac{d E}{d t}=\frac{1}{16\pi }\sum _{\ell \ge 2,|m|\le \ell } \left|
                           \int _{-\infty }^t r\psi _4^{\ell \,m} \, dt^\prime \right| ^2.
                           \end{aligned}$$

The momentum flux leaving the system is

124 $$\begin{aligned}
                           \frac{d P_x+iP_y}{d t}=&\,\frac{r^2}{8\pi }\sum _{\ell \ge
                           2,|m|\le \ell } \int _{-\infty }^t \psi _4^{\ell \,m} \, dt^\prime \int
                           _{-\infty }^t\nonumber \\&\times \left( a_{\ell \,m}\bar{\psi
                           }_4^{\ell ,m+1} +b_{\ell ,-m}\bar{\psi }_4^{\ell -1,m+1} -b_{\ell
                           +1,m+1}\bar{\psi }_4^{\ell +1,m+1} \right) dt^\prime ,
                           \end{aligned}$$

125 $$\begin{aligned}
                           \frac{d P_z}{d t}=&\,\frac{r^2}{16\pi }\sum _{\ell \ge 2,|m|\le
                           \ell } \int _{-\infty }^t \psi _4^{\ell \,m} \, dt^\prime \int _{-\infty
                           }^t \left( c_{\ell \,m}\bar{\psi }_4^{\ell \,m} +d_{\ell \,m}\bar{\psi
                           }_4^{\ell -1,m} +d_{\ell +1,m}\bar{\psi }_4^{\ell +1,m} \right) dt^\prime
                           , \end{aligned}$$

where

126 $$\begin{aligned}
                           a_{\ell \,m}=&\,\frac{\sqrt{(\ell -m)(\ell +m+1)}}{\ell (\ell
                           +1)},\quad b_{\ell \,m}= \frac{1}{2\ell }\sqrt{\frac{(\ell -2)(\ell
                           +2)(\ell +m)(\ell +m-1)}{(2\ell -1)(2\ell +1)}}, \nonumber \\ c_{\ell
                           \,m}=&\,\frac{2m}{\ell (\ell +1)}, \quad d_{\ell \,m}=
                           \frac{1}{\ell }\sqrt{\frac{(\ell -2)(\ell +2)(\ell -m)(\ell +m)}{(2\ell
                           -1)(2\ell +1)}}. \end{aligned}$$

The angular momentum equations become

127 $$\begin{aligned}
                           \frac{d J_x}{d t}=&\,-\frac{ir^2}{32\pi }\mathfrak {I}\left( \sum
                           _{\ell \ge 2,|m|\le \ell } \int _{-\infty }^t \int _{-\infty }^{t^\prime
                           } \psi _4^{\ell \,m} \, dt^{\prime \prime } \, dt^\prime \int _{-\infty
                           }^t \left( f_{\ell \,m}\bar{\psi }_4^{\ell ,m+1} +f_{\ell ,-m}\bar{\psi
                           }_4^{\ell ,m-1} \right) dt^\prime \right) ,
                           \end{aligned}$$

128 $$\begin{aligned}
                           \frac{d J_y}{d t}=&\,-\frac{r^2}{32\pi }\mathfrak {R}\left( \sum
                           _{\ell \ge 2,|m|\le \ell } \int _{-\infty }^t \int _{-\infty }^{t^\prime
                           } \psi _4^{\ell \,m} \, dt^{\prime \prime } \, dt^\prime \int _{-\infty
                           }^t \left( f_{\ell \,m}\bar{\psi }_4^{\ell ,m+1} -f_{\ell ,-m}\bar{\psi
                           }_4^{\ell ,m-1} \right) dt^\prime \right) ,
                           \end{aligned}$$

129 $$\begin{aligned}
                           \frac{d J_z}{d t}=&\,-\frac{ir^2}{16\pi }\mathfrak {I}\left( \sum
                           _{\ell \ge 2,|m|\le \ell } m\int _{-\infty }^t \int _{-\infty }^{t^\prime
                           } \psi _4^{\ell \,m} \, dt^{\prime \prime } \, dt^\prime \int _{-\infty
                           }^t \bar{\psi }_4^{\ell \,m} dt^\prime \right) ,
                           \end{aligned}$$

where the symbol $$\mathfrak
                           {I}$$ refers to the imaginary part and

130 $$\begin{aligned}
                           f_{\ell \,m} :=\sqrt{\ell (\ell +1)-m(m+1)}.
                           \end{aligned}$$

## Gravitational waves in the Cauchy-perturbative approach

Black-hole perturbation theory has been fundamental not only for understanding the stability and oscillations properties of black hole spacetimes (Regge and Wheeler 1957), but also as an essential tool for clarifying the dynamics that accompanies the process of black hole formation as a result of gravitational collapse (Price 1972a, b). As one example among the many possible, the use of perturbation theory has led to the discovery that Schwarzschild black holes are characterised by decaying modes of oscillation that depend on the black hole mass only, i.e., the black hole quasi-normal modes (Vishveshwara 1970b, a; Press 1971; Chandrasekhar and Detweiler 1975). Similarly, black-hole perturbation theory and the identification of a power-law decay in the late-time dynamics of generic black-hole perturbations has led to important theorems, such as the “no hair” theorem, underlining the basic black-hole property of removing all perturbations so that “all that can be radiated away is radiated away” (Price 1972a, b; Misner et al. 1973).

The foundations of non-spherical metric perturbations of Schwarzschild black holes date back to the work of Regge and Wheeler (1957), who first addressed the linear stability of the Schwarzschild solution. A number of investigations, both gauge-invariant and not, then followed in the 1970s, when many different approaches were proposed and some of the most important results about the physics of perturbed spherical and rotating black holes established (Price 1972a, b; Vishveshwara 1970b, a; Chandrasekhar and Detweiler 1975; Zerilli 1970a, b; Moncrief 1974; Cunningham et al. 1978, 1979; Teukolsky 1972, 1973). Building on these studies, which defined most of the mathematical apparatus behind generic perturbations of black holes, a number of applications have been performed to study, for instance, the evolutions of perturbations on a collapsing background spacetime (Gerlach and Sengupta 1979b, a, 1980; Karlovini 2002; Seidel et al. 1987, 1988; Seidel 1990, 1991). Furthermore, the gauge-invariant and coordinate independent formalism for perturbations of spherically symmetric spectimes developed in the 1970s by Gerlach and Sengupta (1979b, 1979a, 1980), has been recently extended to higher-dimensional spacetimes with a maximally symmetric subspace in Kodama et al. (2000), Kodama and Ishibashi (2003), Ishibashi and Kodama (2003), Kodama and Ishibashi (2004), for the study of perturbations in brane-world models.

Also nowadays, when numerical relativity calculations allow to evolve the Einstein equations in the absence of symmetries and in fully nonlinear regimes, black hole perturbative techniques represent important tools.^7^ Schwarzschild perturbation theory, for instance, has been useful in studying the late-time behaviour of the coalescence of compact binaries in a numerical simulation after the apparent horizon has formed (Price and Pullin 1994; Abrahams and Cook 1994; Abrahams et al. 1995). In addition, methods have been developed that match a fully numerical and three-dimensional Cauchy solution of Einstein’s equations on spacelike hypersurfaces with a perturbative solution in a region where the components of three-metric (or of the extrinsic curvature) can be treated as linear perturbations of a Schwarzschild black hole [this is usually referred to as the “Cauchy-Perturbative Matching”] (Abrahams et al. 1998; Rupright et al. 1998; Camarda and Seidel 1999; Allen et al. 1998; Rezzolla et al. 1999a; Lousto et al. 2010; Nakano et al. 2015). This method, in turn, allows to “extract” the gravitational waves generated by the simulation, evolve them out to the wave-zone where they assume their asymptotic form, and ultimately provide outer boundary conditions for the numerical evolution.

This section intends to review the mathematical aspects of the metric perturbations of a Schwarzschild black hole, especially in its gauge-invariant formulations. Special care is paid to “filter” those technical details that may obscure the important results and provide the reader with a set of expressions that can be readily used for the calculation of the odd and even-parity perturbations of a Schwarzschild spacetime in the presence of generic matter-sources. Also, an effort is made to “steer” the reader through the numerous conventions and notations that have accompanied the development of the formalism over the years. Finally, as mentioned in the Introduction, a lot of the material presented here has already appeared in the Topical Review by Nagar and Rezzolla (2006).

### Gauge-invariant metric perturbations

It is useful to recall that even if the coordinate system of the background spacetime has been fixed, the coordinate freedom of general relativity introduces a problem when linear perturbations are added. In particular, it is not possible to distinguish an infinitesimal “physical” perturbation from one produced as a result of an infinitesimal coordinate transformation (or gauge-transformation). This difficulty, however, can be removed either by explicitly fixing a gauge (see, e.g., Regge and Wheeler 1957; Price 1972a, b; Vishveshwara 1970b, a; Zerilli 1970a, b), or by introducing linearly gauge–invariant perturbations (as initially suggested by Moncrief 1974 and subsequently adopted in several applications Cunningham et al. 1978, 1979; Seidel et al. 1987, 1988; Seidel 1990, 1991).

More specifically, given a tensor field $$\varvec{X}$$ and its infinitesimal perturbation $$\delta
                        \varvec{X}$$, an infinitesimal coordinate transformation $$x^{\mu }\rightarrow
                        x^{\mu '} :=x^{\mu }+\xi ^{\mu }$$ with $$\xi ^{\mu }\ll
                        1$$ will yield a new tensor field

131 $$\begin{aligned} \delta
                        \varvec{X}\rightarrow \delta \varvec{X}'=\delta \varvec{X}+ \mathcal
                        {L}_{\varvec{\xi }} \varvec{X}, \end{aligned}$$

where $$\mathcal
                        {L}_{\varvec{\xi }}$$ is the Lie derivative along $$\varvec{\xi
                        }$$. We will then consider $$\delta
                        \varvec{X}$$ to be gauge-invariant if and only if $$\mathcal
                        {L}_{\varvec{\xi }}\varvec{X}=0$$, i.e., if $$\delta
                        \varvec{X}'=\delta \varvec{X}$$. In particular, since gravitational waves are metric perturbations, we will consider the case that $$\varvec{X}$$ is the background metric 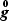, and then metric perturbations are gauge invariant if and only if 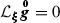.

Stated differently, the possibility of building gauge–invariant metric perturbations relies on the existence of symmetries of the background metric. In the case of a general spherically symmetric background spacetime (i.e., one allowing for a time dependence) and which has been decomposed in multipoles (see Sect. 4.2), the construction of gauge-invariant quantities is possible for multipoles of order $$\ell \ge
                        2$$ only (Gerlach and Sengupta 1979b, a; Martín-García and Gundlach 1999; Gundlach and Martín-García 2000). In practice, the advantage in the use of gauge-invariant quantities is that they are naturally related to scalar observables and, for what is relevant here, to the energy and momentum of gravitational waves. At the same time, this choice guarantees that possible gauge-dependent contributions are excluded by construction.

Of course, this procedure is possible if and only if the background metric has the proper symmetries under infinitesimal coordinates transformation; in turn, a gauge-invariant formulation of the Einstein equations for the perturbations of a general spacetime is not possible. Nevertheless, since any asymptotically flat spacetime can in general be matched to a Schwarzschild one at sufficiently large distances, a gauge-invariant formulation can be an effective tool to extract physical information about the gravitational waves generated in a numerically evolved, asymptotically flat spacetime (Abrahams et al. 1998; Rupright et al. 1998; Camarda and Seidel 1999; Allen et al. 1998; Rezzolla et al. 1999a) (see also Sect. 5.6 for additional implementational details). The following section is dedicated to a review of the mathematical techniques to obtain gauge-invariant perturbations of a the Schwarzschild metric.

### Multipolar expansion of metric perturbations

Given a spherically symmetric Schwarzschild solution with metric 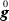 and line element132
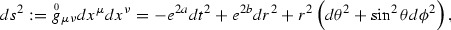
where $$e^{2a}=e^{-2b}=\left(
                        1-2M/r\right) $$, we generically consider small non-spherical perturbations $$h_{\mu \nu
                        }$$ such that the new perturbed metric is133
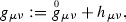
where 
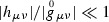
. Although we have chosen to employ Schwarzschild coordinates to facilitate the comparison with much of the previous literature, this is not the only possible choice, nor the best one. Indeed, it is possible to formulate the perturbations equations independently of the choice of coordinates as discussed in Martel and Poisson (2005), or in horizon-penetrating coordinates when the perturbations are in vacuum (Sarbach and Tiglio 2001). Mostly to remain with the spirit of a review and because most of the results have historically been derived in these coordinates, we will hereafter continue to use Schwarzschild coordinates although the reader should bear in mind that this is not the optimal choice.

Because the background manifold $$\mathcal
                        {M}$$ is spatially spherically symmetric, it can be written as the product $$\mathcal
                        {M}=\mathsf{M}^2\times \mathsf{S}^2$$, where $$\mathsf{M}^2$$ is a Lorentzian 2-dimensional manifold of coordinates (*t*, *r*) and $$\mathsf{S}^2$$ is the 2-sphere of unit radius and coordinates $$(\theta , \phi
                        )$$. As a result, the perturbations can be split “ab initio” in a part confined to $$\mathsf{M}^2$$ and in a part confined on the 2-sphere $$\mathsf{S}^2$$ of metric $$\varvec{\gamma
                        }$$. Exploiting this, we can expand the metric perturbations $$\varvec{h}$$ in multipoles referred to as “odd” or “even-parity” according to their transformation properties under parity. In particular, are *odd* (or *axial*) multipoles those that transform as $$(-1)^{\ell
                        +1}$$, under a parity transformation $$(\theta , \phi )
                        \rightarrow (\pi -\theta , \pi +\phi )$$, while are *even* (or *polar*) those multipoles that transform as $$(-1)^{\ell
                        }$$. As a result, the metric perturbations can be written as

134 $$\begin{aligned} h_{\mu
                        \nu }= \sum _{\ell ,m}\left[ \left( h_{\mu \nu }^{{\ell m}}\right)
                        ^{(\mathrm{o})} +\left( h_{\mu \nu }^{\ell m}\right) ^{(\mathrm{e})}\right]
                        , \end{aligned}$$

where $$\sum _{\ell ,m} :=\sum
                        _{\ell =2}^{\infty } \; \sum _{m=-\ell }^{\ell }$$, and the upper indices $$^{(\mathrm{o})}$$ and $$^{(\mathrm{e})}$$ distinguish odd and even-parity objects, respectively. Adapting now a notation inspired by that of Gerlach and Sengupta (Gerlach and Sengupta 1979b, a, 1980) and recently revived by Gundlach and Martín-García (Martín-García and Gundlach 1999; Gundlach and Martín-García 2000, 2001), we use lower-case indices $$a,\,b\, \ldots
                        =0,1$$ to label the coordinates of $$\mathsf{M}^2$$ and upper-case indices $$C,\,D\ldots
                        =2,3$$ to label the coordinates of $$\mathsf{S}^2$$. Using this notation, the scalar spherical harmonics $$Y^{{\ell
                        m}}$$ are then simply defined as

135 $$\begin{aligned} \gamma
                        ^{_{CD}}\nabla _{_D}\nabla _{_C}Y^{{\ell m}}= -\varLambda Y^{{\ell m}},
                        \end{aligned}$$

where $$\nabla
                        _{_C}$$ indicates the covariant derivative with respect to the metric $$\varvec{\gamma
                        }:=\mathrm {diag}(1,\sin ^2\theta )$$ of $$\mathsf{S}^2$$, and where

136 $$\begin{aligned}
                        \varLambda :=\ell (\ell +1). \end{aligned}$$

It is now convenient to express the odd and even-parity metric functions in (133) in terms of tensor spherical harmonics. To do this we introduce the axial vector $$S^{{\ell
                        m}}_{_C}$$ defined as

137 $$\begin{aligned}
                        S_{_C}^{{\ell m}} :=\epsilon _{_{CD}}\gamma ^{_{DE}}\nabla _{_E} Y^{{\ell
                        m}}, \end{aligned}$$

where $$\epsilon
                        _{_{CD}}$$ is the volume form on $$\mathsf{S}^2$$ as defined by the condition $$\epsilon
                        _{_{CD}}\epsilon ^{_{CE}}=\gamma _{_D}^{_{\;\;E}}$$ and such that $$\nabla _{_C}\epsilon
                        _{_{AB}}=0$$. In this way, each odd-parity metric function in (133) can be written as

138 $$\begin{aligned} \left(
                        h_{\mu \nu }^{{\ell m}}\right) ^{(\mathrm{o})}= \left( \begin{array}{c|c} 0
                        &{} h_{a}^{(\mathrm{o})}S_{_C}^{{\ell m}} \\ \hline \\
                        h_{a}^{(\mathrm{o})}S_{_C}^{{\ell m}} &{} h \nabla _{_{(D}}
                        S_{_C)}^{{\ell m}}\\ \end{array} \right) ,\\ \nonumber
                        \end{aligned}$$

where $$h,h_{a}^{(\mathrm{o})}$$ are functions of (*t*, *r*) only, and where we have omitted the indices $$\ell ,
                        m$$ on $$h,h_{a}^{(\mathrm{o})}$$for clarity.

Proceeding in a similar manner, each even-parity metric function can be decomposed in tensor spherical harmonics as

139 $$\begin{aligned} \left(
                        h_{\mu \nu }^{{\ell m}}\right) ^{(\mathrm {e})} =\left( \begin{array}{cc|cc}
                        e^{2a} H_0Y^{{\ell m}} &{} H_1Y^{{\ell m}} &{} \qquad
                        h_{a}^{(\mathrm{e})}\nabla _{_C}Y^{{\ell m}} \\ H_1Y^{{\ell m}} &{}
                        e^{2b} H_2Y^{{\ell m}} &{} \\ \hline &{} &{}\\
                        h_{a}^{(\mathrm{e})}\nabla _{_C} Y^{{\ell m}} &{} &{}
                        r^2\left( KY^{{\ell m}}\gamma _{_{CD}}+ G \nabla _{_D}\nabla _{_C} Y^{{\ell
                        m}}\right) \\ \end{array}\right) , \end{aligned}$$

where $$H_0,\,H_1,\,H_2,\,
                        h_0^{(\mathrm{e})},\,h_1^{(\mathrm{e})}\, K$$, and *G* (with the indices $$\ell ,
                        m$$ omitted for clarity) are the coefficients of the even-parity expansion, are also functions of (*t*, *r*) only.

Note that we have used the Regge–Wheeler set of tensor harmonics to decompose the even-parity part of the metric in multipoles (Regge and Wheeler 1957). Despite this being a popular choice in the literature, it is not the most convenient one since the tensor harmonics in this set are not linearly independent. An orthonormal set is instead given by the Zerilli–Mathews tensor harmonics (Zerilli 1970; Mathews 1962) and the transformation from one basis to the other is given by defining the tensor $$Z_{_{CD}}^{{\ell
                        m}}$$ confined on the 2-sphere $$\mathsf{S}^2$$ (see also section “Vector and tensor spherical harmonics” in “Appendix 2”)

140 $$\begin{aligned}
                        Z_{_{CD}}^{{\ell m}} :=\nabla _{_C} \nabla _{_D} Y^{{\ell m}}+
                        \frac{\varLambda }{2}\gamma _{_{CD}}Y^{{\ell m}},
                        \end{aligned}$$

and then replacing in Eq. (139) the second covariant derivative of the spherical harmonics $$\nabla _{_C}\nabla
                        _{_D}Y^{{\ell m}}$$ with $$Z^{{\ell
                        m}}_{_{CD}}$$. This transformation has to be taken into account, for instance, when developing gauge-invariant procedures for extracting the gravitational-wave content from numerically generated spacetimes which are “almost” Schwarzschild spacetimes (Abrahams et al. 1992; Anninos et al. 1993, 1995a, b; Abrahams and Price 1996b, a).

Besides vacuum tensor perturbations, the background Schwarzschild spacetime can be modified if non-vacuum tensor perturbations are present and have a nonzero mass-energy, but much smaller than that of the black hole. In this case, the generic stress-energy tensor $$t_{\mu \nu
                        }$$ describing the matter-sources can be similarly decomposed in odd and even-parity parts

141 $$\begin{aligned} t_{\mu
                        \nu }=\sum _{\ell ,m}\left[ \left( t^{{\ell m}}_{\mu \nu }\right)
                        ^{(\mathrm{o})}+\left( t^{{\ell m}}_{\mu \nu }\right) ^{(\mathrm{e})}\right]
                        , \end{aligned}$$

that are naturally gauge-invariant since the background is the vacuum Schwarzschild spacetime and are given explicitely by

142 $$\begin{aligned} \left(
                        t^{{\ell m}}_{\mu \nu }\right) ^{(\mathrm{o})}= \left( \begin{array}{cc} 0
                        &{}\quad L_{a}^{{\ell m}}S_{_C}^{{\ell m}} \\ \\ L_{a}^{{\ell
                        m}}S_{_C}^{{\ell m}} &{}\quad L^{{\ell m}} \nabla
                        _{_{(D}}S_{_{C)}}^{{\ell m}}\\ \end{array} \right) ,
                        \end{aligned}$$

for the odd-parity part and by

143 $$\begin{aligned} \left(
                        t^{{\ell m}}_{\mu \nu }\right) ^{(\mathrm{e})}= \left( \begin{array}{c|c}
                        T_{ab}^{{\ell m}}Y^{{\ell m}} &{} T_{a}^{{\ell m}}\nabla _{_C}
                        Y^{{\ell m}}\\ &{} \\ \hline &{} \\ T_{a}^{{\ell m}}\nabla
                        _{_C} Y^{{\ell m}} &{} r^2T_3^{{\ell m}}Y^{{\ell m}}\gamma _{_{CD}}+
                        T_2^{{\ell m}}Z_{_{CD}}^{{\ell m}}\\ \end{array} \right) ,
                        \end{aligned}$$

for the even-parity one. Note that we have now used the Zerilli-Matthews set of harmonics for the expansion, that the ten coefficients $$L_{a}^{{\ell m}},\,
                        L^{{\ell m}},\, T_{ab}^{{\ell m}},\, T_{a}^{{\ell m}},\, T_2^{{\ell m}},\,
                        T_3^{{\ell m}}$$ are gauge-invariant, and that explicit expressions for them will be presented in the following sections.

Let us now consider the Einstein field equations that, in the static vacuum background, take the simple form144
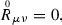
where 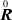 is the Ricci tensor built from the background metric 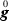. At first order in the perturbations, the field equations reduce to145
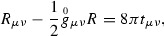
where $\varvec{R}$ is now the Ricci tensor built from the metric perturbations $\varvec{h}$.

Note that while a generic perturbation will be a mixture of odd and even-parity contributions, we will exploit the linearity of the approach to handle them separately and simplify the treatment. In the following two sections we will discuss the form the Einstein equations (145) assume in response to purely odd and even-parity perturbations over a Schwarzschild background. In particular, we will show how the three odd-parity coefficients of the expansion in harmonics of the metric, i.e., $$h_{a}^{(\mathrm{o})},\,h$$, and the seven even-parity ones, i.e., $$H_0,\,H_1,\,H_2,\,
                        h_0^{(\mathrm{e})},\,h_1^{(\mathrm{e})}\, K,\, G$$, can be combined to give two gauge-invariant master equations, named respectively after Regge and Wheeler (1957) and Zerilli (1970), each of which is a wave-like equation in a scattering potential.^8^

Although our attention is here focussed on the *radiative* degrees of freedom of the perturbations (i.e., those with $$\ell \ge
                        2$$) because of their obvious application to the modelling of sources of gravitational waves, a comment should be made also on lower-order multipoles. In particular, it is worth remarking that the monopole component of the metric for a vacuum perturbation (i.e., with $$\ell =
                        0$$) is only of even-parity type and represents a variation in the mass-parameter of the Schwarzschild solution. On the other hand, the dipole component of the even-parity metric for a vacuum perturbation (i.e., with $$\ell
                        =1$$) is of pure-gauge type and it can be removed by means of a suitable gauge transformation (Zerilli 1970b). This is not the case for a dipolar odd-parity metric perturbation, which can instead be associated to the introduction of angular momentum onto the background metric.

### Gauge-invariant odd-parity perturbations

Before discussing the derivation of the odd-parity equation, we should make a choice for the odd-parity master function. Unfortunately, this choice has not been unique over the years and different authors have made different choices, making comparisons between different approaches less straightforward. Here, we will make a choice which highlights the relation with the gravitational-wave amplitude measured by a distant observer and, in particular, we construct the gauge-invariant combination of multipoles (Gerlach and Sengupta 1979b; Martín-García and Gundlach 1999; Harada et al. 2003)

146 $$\begin{aligned} k_{a}
                        :=h_{a}-\nabla _{\!a} h +2h\frac{\nabla _{\!a}r}{r},
                        \end{aligned}$$

where, we recall, $$\nabla
                        _{\!a}$$ represents the covariant derivative with respect to the connection of the submanifold $$\mathsf{M}^2$$. If $$\epsilon
                        _{ab}$$ is the antisymmetric volume form on $$\mathsf{M}^2$$, then the function

147 $$\begin{aligned} \Phi
                        ^{(\mathrm{o})}(t,r) :=r^3\epsilon ^{ab}\nabla _{\!b}\left(
                        \frac{k_{a}}{r^2}\right) = r\left[ \partial _{t}h_{1}^{(\mathrm{o})}-
                        r^2\partial _r\left( \frac{h_0^{(\mathrm{o})}}{r^2}\right) \right] ,
                        \end{aligned}$$

is gauge-invariant and will be our choice for the Regge–Wheeler master function (Gerlach and Sengupta 1979b; Martín-García and Gundlach 1999; Gundlach and Martín-García 2000; Harada et al. 2003).

A slight variation of the master function (147) has been introduced by Cunningham et al. (1978) in terms of the function $$\widetilde{\psi }
                        :=\varLambda \Phi ^{(\mathrm{o})}$$ and this has been used so extensively in the literature (Seidel et al. 1987, 1988; Seidel 1991) that it is now commonly referred to as the Cunningham-Price-Moncrief (CPM) convention. We partly follow this suggestion and introduce a new master function for the odd-parity perturbations defined as

148 $$\begin{aligned} \varPsi
                        ^{(\mathrm{o})} :=\frac{1}{\varLambda -2} \,\Phi ^{(\mathrm{o})}.
                        \end{aligned}$$

With the choice (148), the Einstein field equations (145) with odd-parity perturbations lead to the inhomogeneous “Regge–Wheeler” equation

149 $$\begin{aligned} \partial
                        ^2_{t} \varPsi ^{(\mathrm{o})} - \partial ^2_{r_*} \varPsi ^{(\mathrm{o})} +
                        V_{\ell }^{(\mathrm{o})}\varPsi ^{(\mathrm{o})}=S^{(\mathrm{o})},
                        \end{aligned}$$

where

150 $$\begin{aligned}
                        r_*:=r+2M\ln \left( \frac{r}{2M}-1\right) ,
                        \end{aligned}$$

is the “tortoise coordinate” (Misner et al. 1973) and $$V_{\ell
                        }^{(\mathrm{o})}$$ is the odd-parity potential, defined as

151 $$\begin{aligned} V_{\ell
                        }^{(\mathrm{o})} :=\left( 1-\frac{2M}{r}\right) \left( \frac{\varLambda
                        }{r^2}- \frac{6M}{r^3} \right) . \end{aligned}$$

The right-hand side of Eq. (149) represents the generic odd-parity “matter-source” and is given by

152 $$\begin{aligned}
                        S^{(\mathrm{o})} :=\frac{16\pi r}{\varLambda -2} \, e^{2a}\epsilon
                        ^{bc}\nabla _{\!c}L_{b} = \frac{16\pi r}{\varLambda -2} \left[ \left(
                        1-\frac{2M}{r}\right) \partial _{t}L_{1}^{{\ell m}} - \partial _{r_*}
                        L_{0}^{{\ell m}}\right] , \end{aligned}$$

with the components of the odd-parity matter-source vector defined as

153 $$\begin{aligned}
                        L_{a}^{{\ell m}} :=\frac{1}{\varLambda }\int \frac{d\varOmega }{\sin \theta
                        }\left( {i}\,m\, t_{{a2}}Y^{*}_{{\ell m}}+ t_{{a3}}\,\partial _{\theta
                        }Y_{\ell m}^{*}\right) , \qquad a=0,1,
                        \end{aligned}$$

and where $$d\varOmega =\sin \theta
                        d\phi d\theta $$ is the surface element on the 2-sphere $$\mathsf{S}^2$$.

Another choice for the gauge-invariant odd-parity master variable is possible and indeed was originally proposed by Moncrief (1974). This function, which hereafter we will refer to as the odd-parity Moncrief function, is defined as154
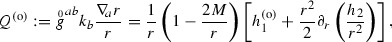
where the first expression is coordinate independent (Martel and Poisson 2005), while the second one is specialized to Schwarzschild coordinates with $$h_2 = - 2
                        h$$ (Moncrief 1974). In the Regge–Wheeler gauge, i.e., for $$h_2=h=0$$, the definition (154) coincides with the variable used by Regge and Wheeler (1957). Historically, the choice of (154) as master variable has been the most common in the literature to describe odd-parity perturbations of a Schwarzschild spacetime and we will refer to it as “Regge–Wheeler” (RW) convention. It should be noted that while (154) is a solution of the Regge–Wheeler equation, the corresponding source term differs from expression (152). A general expression of the source in the RW convention can be found in Martel and Poisson (2005), Martel (2004) together with its specification for a point-particle (see also Andrade and Price 1999; Tominaga et al. 1999; Ferrari and Kokkotas 2000).

The two master functions $$Q^{(\mathrm
                        o)}$$ and $$\varPsi ^{(\mathrm
                        o)}$$ are intimately related through the variational formalism employed by Moncrief (1974), and through the explicit expression (Martel and Poisson 2005)

155 $$\begin{aligned} \partial
                        _t\varPsi ^{(\mathrm o)}=-Q^{(\mathrm o)} + \frac{16\pi }{\varLambda
                        -2}\frac{r}{e^{2b}} L_1^{\ell m} . \end{aligned}$$

Note that Eq. (155) highlights an important difference between the two master functions which is not just a dimensional one (i.e., $$\varPsi
                        ^{(\mathrm{o})}$$ has the dimensions of a length, while $$Q^{(\mathrm{o})}$$ is dimensionless) and this will have consequences on the asymptotic expressions for the gravitational waveforms when these are expressed in one or in the other convention. A detailed discussion of this will be made in Sects. 5.1, 5.3 and 5.4.

### Gauge-invariant even-parity perturbations

Also in the case of even-parity perturbations, it is possible to express the evolution of the even-parity perturbations in terms of a wave-like equation in a scattering potential [cf. the Regge–Wheeler equation (149)]. In particular, following Moncrief (1974), we define the gauge-invariant functions

156 $$\begin{aligned} \kappa
                        _1:= & {} K+\frac{1}{e^{2b}}\left( r\partial _r G-
                        \frac{2}{r}h_1^{(\mathrm{e})}\right) ,
                        \end{aligned}$$

157 $$\begin{aligned} \kappa
                        _2:= & {} \frac{1}{2}\left[ e^{2b}H_2- e^b \partial _r \left( r e^b
                        K\right) \right] , \end{aligned}$$

and where their linear combination

158 $$\begin{aligned} q_1
                        :=r\varLambda \kappa _1 + \frac{4r}{e^{4b}}\kappa _2 .
                        \end{aligned}$$

is also a gauge-invariant function. Strictly related to expression (158) is the gauge-invariant function most frequently used in the literature (Seidel 1990; Gundlach and Martín-García 2001; Martel 2004; Lousto and Price 1997; Ruoff 2001; Ruoff et al. 2001; Martel and Poisson 2002; Nagar et al. 2004; Poisson 2004)

159 $$\begin{aligned} {\varPsi
                        }^{(\mathrm{e})} :=\frac{r q_1}{\varLambda \left[ r\left( \varLambda
                        -2\right) +6M\right] } , \end{aligned}$$

which is also the solution of the inhomogeneous even-parity master equation or “Zerilli” equation

160 $$\begin{aligned} \partial
                        ^2_{t}{\varPsi }^{(\mathrm{e})} - \partial ^2_{r_*}{\varPsi
                        }^{(\mathrm{e})}+ V_{\ell }^{(\mathrm{e})}{\varPsi
                        }^{(\mathrm{e})}=S^{(\mathrm{e})} , \end{aligned}$$

and, again, is a wave-like equation in the scattering Zerilli potential (Zerilli 1970a)

161 $$\begin{aligned} V_{\ell
                        }^{(\mathrm{e})} :=\left( 1-\frac{2M}{r}\right) \frac{\varLambda (\varLambda
                        -2)^2r^3 +6(\varLambda -2)^2Mr^2+36(\varLambda -2)M^2r+72M^3}{r^3\left[
                        (\varLambda -2)r+6M\right] ^2}. \end{aligned}$$

The even-parity matter-source has a rather extended expression given by Martel (2004), Nagar et al. (2004, 2005)

162 $$\begin{aligned}
                        S^{(\mathrm{e})}\!= & {} \!-\frac{8\pi }{\varLambda \left[
                        (\varLambda \!-\!2)r\!+\!6M\right] } \Bigg \{ \frac{\varLambda \Big
                        (6r^3-16Mr^2\Big )-r^3\varLambda ^2-8r^3\!+\!68Mr^2-108M^2r}{(\varLambda
                        -2)r\!+\!6M} T_{00}^{{\ell m}} \nonumber \\&+\frac{1}{e^{4b}}\Big
                        [2Mr+r^2(\varLambda -4)\Big ]T_{11}^{{\ell m}}+ 2r^3 \partial _{r_*}
                        T_{00}^{{\ell m}} -\frac{2r^3}{e^{4b}} \partial _{r_*} T_{11}^{{\ell m}}
                        \nonumber \\&+\frac{4\varLambda r}{e^{4b}}T_{1}^{{\ell m}}
                        +\frac{1}{e^{2b}}\left[ 2\varLambda \left( 1-\frac{3M}{r}\right) -
                        \varLambda ^2\right] T_{2}^{{\ell m}}+ \frac{4r^2}{e^{4b}}T_3^{{\ell
                        m}}\Bigg \}. \end{aligned}$$

Note that the expressions of the even-parity vector and tensor spherical-harmonics for the matter-source needed in (162) can be obtained from the orthogonality properties of the harmonics and are

163 $$\begin{aligned}&\displaystyle T_{a}^{{\ell
                        m}}=\frac{1}{\varLambda }\int d\varOmega \left[ t_{a 2} \partial _{\theta
                        }(\bar{Y}^{\ell m}) - t_{a 3} \frac{ i m \bar{Y}^{\ell m}}{\sin ^2\theta }
                        \right] , \quad a = 0,1, \end{aligned}$$

164 $$\begin{aligned}&\displaystyle T_{2}^{{\ell
                        m}}\!=\!\frac{2}{\varLambda (\varLambda -2)} \int d\varOmega \bigg [t_{22}
                        \frac{\bar{W}^{\ell m}}{2} + t_{23} \frac{2\bar{X}^{{\ell m}}}{\sin \theta }
                        +t_{33} \bigg (\frac{\varLambda \bar{Y}^{{\ell m}}}{2} -\frac{m^2
                        \bar{Y}^{{\ell m}}}{\sin ^2\theta } + \cot \theta \partial _{\theta
                        }\bar{Y}^{\ell m}\bigg ) \bigg ],\qquad \quad
                        \end{aligned}$$

165 $$\begin{aligned}&\displaystyle T_{3}^{{\ell m}}
                        =\frac{1}{2r^2}\int d\varOmega \left( t_{22}+t_{33}\frac{1}{\sin ^2\theta
                        }\right) \bar{Y}^{\ell m}, \end{aligned}$$

166 $$\begin{aligned}&\displaystyle T_{ab}^{{\ell
                        m}}=\int d\varOmega \,t_{ab}\, \bar{Y}^{\ell m},\; \qquad a,b = 0,1 ,
                        \end{aligned}$$

where the angular functions $$W^{\ell m}(\theta , \phi
                        )$$ and $$X^{\ell m}(\theta , \phi
                        )$$ are defined as Regge and Wheeler (1957)

167 $$\begin{aligned}&\displaystyle W^{{\ell m}}
                        :=\frac{\nabla _{(\phi }S_{\theta )}^{\ell m}}{\sin \theta }= \partial
                        ^2_{\theta }Y^{{\ell m}}-\cot \theta \,\partial _{\theta } Y^{{\ell
                        m}}-\frac{1}{\sin ^2\theta }\partial ^2_{\phi } Y^{{\ell m}},
                        \end{aligned}$$

168 $$\begin{aligned}&\displaystyle X^{{\ell m}} :=-\sin
                        \theta \left( \nabla _{\theta }S_{\theta }^{\ell m}- \frac{\nabla _\phi
                        S_{\phi }^{\ell m}}{\sin ^2\theta }\right) = 2\left( \partial ^2_{\theta
                        \phi } Y^{{\ell m}}-\cot \theta \partial _{\phi }Y^{{\ell m}}\right) ,
                        \end{aligned}$$

and where the overbar stands for complex conjugation.

We should note that even-parity functions can be found in the literature under different notations. A particularly common choice is that proposed in Moncrief (1974) for the even parity gauge-invariant master function $$Q^{(\mathrm{e})}$$ which is related to Zerilli function (159) simply as $$Q^{(\mathrm{e})} =
                        \varLambda \varPsi ^{(\mathrm{e})}$$, while other authors use instead a master function defined as $$Z:=2{\varPsi
                        }^{(\mathrm{e})}$$ (Martel 2004; Lousto and Price 1997). Another even-parity function can be introduced in terms of two new gauge-invariant metric functions *k* and $$\chi
                        $$ are defined as Gundlach and Martín-García (2000, 2001)

169 $$\begin{aligned}&\displaystyle k = \kappa _1 =
                        K+\frac{1}{e^{2b}}\left[ r \partial _r G-
                        \frac{2}{r}h_1^{(\mathrm{e})}\right] ,
                        \end{aligned}$$

170 $$\begin{aligned}&\displaystyle \chi + k = H_2
                        -\frac{2}{e^{2b}}\partial _rh_1^{(\mathrm e)}- \frac{2M}{r^2}h_1^{(\mathrm
                        e)} + \frac{1}{e^{2b}}\partial _r \left( r^2\partial _rG\right) +M\partial
                        _r G,&\end{aligned}$$

and such that

171 $$\begin{aligned} \kappa
                        _2 = \frac{1}{2}e^{2b}\left( \chi -r\partial _r k+\frac{M}{r}e^{2b} k\right)
                        . \end{aligned}$$

In this case, the Zerilli function (159) can be equivalently defined as Gundlach and Martín-García (2001)

172 $$\begin{aligned} {\varPsi
                        }^{(\mathrm{e})}:=\frac{2r^2}{\varLambda \left[ (\varLambda -2)r+6M\right]
                        e^{2b}} \left[ \chi +\left( \frac{\varLambda }{2}+\frac{M}{r}\right) e^{2b}
                        k -r \partial _{r} k\right] . \end{aligned}$$

Finally, the homogeneous odd and even-parity master equations (149) and (160) can be transformed into each other by means of differential operations (Chandrasekhar 1983), and that they are connected to the master equation that Bardeen and Press have derived via the Newman–Penrose formalism (Bardeen and Press 1973).

## Numerical implementations of the Cauchy-perturbative approach

In the previous section we have reviewed the derivation of the equations describing the evolution of perturbations of nonrotating black holes induced, for instance, by a nonzero stress-energy tensor. These perturbations have been assumed to be generic in nature, needing to satisfy only the condition of having a mass-energy much smaller than that of the black hole. The solution of these equations with suitable initial conditions completely specifies the reaction of the black hole to the perturbations and this is essentially represented by the emission of gravitational waves.

As mentioned in Sect. 4.1, the importance of the gauge-invariant variables used so far is that they are directly related to the amplitude and energy of the gravitational-wave signal measured at large distances. The purpose of this Chapter is to review the steps necessary to obtain the relations between the master functions for the odd and even-parity perturbations and the “plus” and “cross” polarisation amplitudes $$h_+, h_\times
                     $$ of a gravitational wave in the TT gauge. In practice, and following the guidelines tracked in Cunningham et al. (1978, 1979), we will derive an expression for the perturbation metric $$\varvec{h}$$ equivalent to that obtained in the standard TT gauge on a Minkowski spacetime and relate it to the odd and even-parity master functions $$\varPsi
                     ^{(\mathrm{o})}$$ and $$\varPsi
                     ^{(\mathrm{e})}$$.

To obtain this result a number of conditions need to be met. First, we need to evaluate each multipole of the decomposed metric perturbations in the tetrad $$\varvec{e}_{\hat{\nu
                     }}$$ of stationary observers in the background Schwarzschild spacetime, i.e., $$h_{\hat{\mu }\hat{\nu }} =
                     \varvec{e}^{\mu }_{\hat{\mu }} \varvec{e}^{\nu }_{\hat{\nu }} h_{\mu \nu
                     }$$, where $$\varvec{e}$$ is diagonal with components

173 $$\begin{aligned}
                     \varvec{e}_{\hat{\mu }}^{\mu }:=\left( e^b, e^{-b}, r^{-1}, (r \sin \theta
                     )^{-1}\right) , \end{aligned}$$

and where the indices $${\hat{\mu
                     }}$$ refer to the locally “flat” coordinates. Second, all of the quantities need to be evaluated far away from the source (i.e., in the “wave-zone”) and in the so-called *radiation gauge*. In practice, this amounts to requiring that that components $$h_{\hat{\theta }\hat{\theta
                     }}$$, $$h_{\hat{\phi }\hat{\phi
                     }}$$ and $$h_{\hat{\theta }\hat{\phi
                     }}$$ are functions of the type $$f(t-r)/r$$ (i.e., they are outgoing spherical waves), while all the other components have a more rapid decay of $$\mathcal
                     {O}(1/r^2)$$. Finally, we need to impose the condition that the metric is traceless modulo higher order terms, i.e., $$h_{{\hat{\theta
                     }}{\hat{\theta }}} + h_{\hat{\phi }\hat{\phi }}= 0 + \mathcal
                     {O}(1/r^2)$$.

In the following sections we will discuss the asymptotic expressions from odd- and even-parity perturbations, and how to implement the Cauchy-perturbative approach to extract gravitational-wave information within a standard numerical-relativity code.

### Asymptotic expressions from odd-parity perturbations

We first consider odd-parity perturbations and recall that from the radiation-gauge conditions and since for large *r* the metric asymptotes that of a flat spacetime, i.e., $$e^{b}\sim e^{-b}\sim
                        1$$, we have

174 $$\begin{aligned}
                        h_{\hat{\theta }\hat{t}}^{(\mathrm{o})}&= \displaystyle
                        \frac{h_0^{(\mathrm{o})}}{r}e^{b}S_{\theta }\sim
                        \frac{h_0^{(\mathrm{o})}}{r}\sim \mathcal {O}\left( \frac{1}{r^2}\right)
                        \;\longrightarrow h_0^{(\mathrm{o})}\sim \mathcal {O}\left(
                        \frac{1}{r}\right) , \end{aligned}$$

175 $$\begin{aligned}
                        h_{\hat{\theta }\hat{r}}^{(\mathrm{o})}&= \displaystyle
                        \frac{h_1^{(\mathrm{o})}}{r}e^{b}S_{\theta }\sim
                        \frac{h_1^{(\mathrm{o})}}{r}\sim \mathcal {O}\left( \frac{1}{r^2}\right)
                        \;\longrightarrow h_1^{(\mathrm{o})}\sim \mathcal {O}\left(
                        \frac{1}{r}\right) , \end{aligned}$$

where the $$\ell ,
                        m$$ indices have been omitted for clarity. Similarly, since $$h_{\hat{\theta
                        }\hat{\theta }}^{(\mathrm{o})} = 2h r^{-2} \nabla _{\theta } S_{\theta }\sim
                        \mathcal {O}(1/r)$$, we can deduce that $$h\sim \mathcal
                        {O}(r)$$, so that the only components of the metric having wave-like properties at large *r* are

176 $$\begin{aligned}&\displaystyle h_{+}^{(\mathrm{o})}
                        :=\frac{1}{2}\left( h_{\hat{\theta }\hat{\theta
                        }}^{(\mathrm{o})}-h_{\hat{\phi }\hat{\phi }}^{(\mathrm{o})}\right)
                        =\frac{h}{r^2}\left( \nabla _{\theta }S_{\theta }- \frac{\nabla _{\phi
                        }S_{\phi }}{\sin ^2\theta }\right) + \mathcal {O}\left( \frac{1}{r^2}\right)
                        , \end{aligned}$$

177 $$\begin{aligned}&\displaystyle h_{\times
                        }^{(\mathrm{o})}:=h_{\hat{\theta } \hat{\phi }}^{(\mathrm{o})}=\frac{h}{r^2}
                        \frac{\nabla _{(\phi }S_{\theta )}}{\sin \theta }+ \mathcal {O}\left(
                        \frac{1}{r^2}\right) . \end{aligned}$$

Note that since *h* has the dimensions of a length squared, both $$h_{+}$$ and $$h_{\times
                        }$$ are dimensionless. Next, we need to relate the perturbation *h* to the odd-parity master function $$\varPsi
                        ^{(\mathrm{o})}$$. To do so, we follow the procedure outlined in Cunningham et al. (1978), and note that (cf. Eq. (III-20)^9^)

178 $$\begin{aligned} \partial
                        _t h =\left( 1-\frac{2M}{r}\right) \partial _r\left( r\varPsi ^{(\mathrm
                        o)}\right) +h_0^{(\mathrm o)}. \end{aligned}$$

Equation (178) represents one of the Hamilton equations as derived by Moncrief in a Hamiltonian formulation of perturbation equations (Moncrief 1974). The radiation-gauge conditions on *h* and $$h_0^{(\mathrm
                        o)}$$ imply that $$\varPsi
                        ^{(\mathrm{o})}\sim \mathcal {O}(1)$$, i.e., in the wave-zone $$\varPsi
                        ^{(\mathrm{o})}$$ has the dimensions of a length, behaves as an outgoing-wave, but it does not depend explicitly on *r*.

Exploiting now the outgoing-wave behaviour of *h* at large distances we can write

179 $$\begin{aligned} \partial
                        _t h=-\partial _r h+\mathcal {O}\left( \frac{1}{r}\right) ,
                        \end{aligned}$$

so that asymptotically Eq. (178) simply becomes

180 $$\begin{aligned} \partial
                        _r h=-\partial _r\left( r\varPsi ^{(\mathrm o)}\right) +\mathcal {O} \left(
                        \frac{1}{r}\right) , \end{aligned}$$

and its integration yields

181 $$\begin{aligned}
                        \frac{h}{r}\sim -\varPsi ^{(\mathrm{o})}+\mathcal {O}\left(
                        \frac{1}{r}\right) . \end{aligned}$$

As a result, the “$$+$$” and “$$\times
                        $$” polarisation amplitudes of the gravitational wave can be calculated from Eqs. (177)–(176) as

182 $$\begin{aligned}
                        h_{+}^{(\mathrm{o})}&=-\frac{1}{r}\varPsi ^{(\mathrm{o})}\; \left(
                        \nabla _\theta S_{\theta }- \frac{\nabla _\phi S_{\phi }}{\sin ^2\theta
                        }\right) + \mathcal {O}\left( \frac{1}{r^2}\right) ,
                        \end{aligned}$$

183 $$\begin{aligned}
                        h_{\times }^{(\mathrm{o})}&=-\frac{1}{r} \varPsi ^{(\mathrm{o})}\;
                        \frac{\nabla _{(\phi }S_{\theta )}}{\sin \theta }+\mathcal {O}\left(
                        \frac{1}{r^2}\right) . \end{aligned}$$

Expressions (182) and (183) can be written in a compact form using the $s=-2$ spin-weighted spherical harmonics (see also section “Vector and tensor spherical harmonics” in “Appendix 2”)

184 $$\begin{aligned}
                        _{_{-2}}Y^{{\ell m}}(\theta ,\phi ):=\sqrt{\frac{(\ell -2)!}{(\ell +2)!}}
                        \left( W^{{\ell m}}-{i}\; \frac{X^{{\ell m}}}{\sin \theta }\right) ,
                        \end{aligned}$$

so that expressions (182) and (183) can be combined into a single complex expression given by

185 $$\begin{aligned} \left(
                        h^{(\mathrm{o})}_+ - {i}h^{(\mathrm{o})}_{\times }\right) _{{\ell m}}=
                        \frac{{i}}{r}\;\sqrt{\frac{(\ell +2)!}{(\ell -2)!}}\; \varPsi
                        ^{(\mathrm{o})}_{{\ell m}}\;_{_{-2}}Y^{{\ell m}}(\theta ,\phi ) + \mathcal
                        {O}\left( \frac{1}{r^2}\right) , \end{aligned}$$

where, for clarity, we have explicitly restored the multipole indices $$\ell ,
                        m$$.

#### The master function $Q^{(\mathrm{o})}$

As discussed in Sect. 4.3, the odd-parity metric perturbations are sometimes expressed in terms of the odd-parity Moncrief function $Q^{(\mathrm{o})}$ [cf. Eq. (154)]; indeed it is not unusual to find in the literature the gravitational-wave amplitudes expressed in terms of this quantity. However, great care must be paid to the asymptotic relation between the master function $Q^{(\mathrm{o})}$ and the gravitational-wave amplitudes and, indeed, this is sometimes a source of confusion (Kawamura and Oohara 2004; Kawamura et al. 2003). To clarify this point, we recall that the derivation of the asymptotic relation between $Q^{(\mathrm{o})}$ and *h* proceeds in a way similar to the one discussed above. In the radiation gauge and at large distances from the black hole, we can use relation (179) in the definition (154) with $h_2=-2h$, so that

186 $$\begin{aligned}
                           Q^{(\mathrm{o})}\sim \frac{1}{r} \partial _t h + \mathcal {O}\left(
                           \frac{1}{r}\right) , \end{aligned}$$

which is also a dimensionless quantity. Since $$h \sim \mathcal
                           {O}(r)$$, the function $$Q^{(\mathrm{o})}$$ does not depend on *r* at leading order and Eq. (186) can be integrated to give

187 $$\begin{aligned}
                           \frac{h(t)}{r}\sim \int _{-\infty }^{t} Q^{(\mathrm{o})}(t') \, dt' +
                           \mathcal {O}\left( \frac{1}{r}\right) + \mathrm {const.},
                           \end{aligned}$$

where the integration constant can be defined as

188 $$\begin{aligned}
                           \mathrm {const.}:=\lim _{t\rightarrow - \infty } \frac{h(t,r)}{r}\sim
                           \mathcal {O}(1), \end{aligned}$$

and it can be set to zero in the case of asymptotically flat metric perturbations ($h=0$) at earlier times. Combining now expressions (167), (168) and (187), the gravitational-wave amplitudes in the two polarisations and with the new master function read

189 $$\begin{aligned}
                           \left( h^{(\mathrm{o})}_+ -{i}h^{(\mathrm{o})}_{\times }\right) _{{\ell
                           m}}= -\frac{{i}}{r}\sqrt{\frac{(\ell +2)!}{(\ell -2)!}} \left( \int
                           _{-\infty }^{t} Q^{(\mathrm{o})}_{{\ell m}}(t')dt' \right)
                           \;_{_{-2}}Y^{{\ell m}}(\theta ,\phi )+ \mathcal {O}\left(
                           \frac{1}{r^2}\right) . \end{aligned}$$

Note that in expressions (185) and (189) the quantities $$\varPsi
                           ^{(\mathrm{o})}$$ and $$Q^{(\mathrm{o})}$$ are both solutions of the Regge–Wheeler equation (149), but they yield two different asymptotic expressions for the gravitational-wave amplitudes. This difference, which is consistent with Eq. (155) when evaluated in a an asymptotic region of the spacetime where $$L_1^{\ell
                           m}=0$$, is subtle but important and, as mentioned above, it has led to some inconsistencies in the literature both for the determination of the asymptotic gravitational-wave amplitudes and for the energy losses. This will be further discussed in Sects. 5.3 and 5.4.

### Asymptotic expressions from even-parity perturbations

A calculation conceptually analogous to the one carried out in Sect. 5.1 leads to the relation between the gravitational-wave amplitude and the even-parity master function. In particular, after projecting Eq. (139) along the stationary tetrad, the asymptotic wave amplitudes in the two polarisation states are

190 $$\begin{aligned}&\displaystyle
                        h_{+}^{(\mathrm{e})}=\frac{1}{2} \left( h_{\hat{\theta }\hat{\theta
                        }}^{(\mathrm{e})}- h_{\hat{\phi }\hat{\phi }}^{(\mathrm{e})}\right) =
                        \frac{G}{2}\left( \nabla _\theta \nabla _\theta Y^{{\ell m}}-\frac{\nabla
                        _\phi \nabla _\phi Y^{{\ell m}}}{\sin ^2\theta }\right)
                        =\frac{G}{2}\;W^{{\ell m}}, \end{aligned}$$

191 $$\begin{aligned}&\displaystyle h_{\times
                        }^{(\mathrm{e})}=h_{\hat{\theta }\hat{\phi }}^{(\mathrm{e})}=G\,
                        \frac{\nabla _\theta \nabla _\phi Y^{{\ell m}}}{\sin \theta }
                        =\frac{G}{2}\;\frac{\;\;\;X^{{\ell m}}}{\sin \theta },
                        \end{aligned}$$

so that we essentially need to relate the metric perturbation *G* with the even-parity function $${\varPsi
                        }^{(\mathrm{e})}$$. Firstly, it is easy to realize that the even-parity metric projected onto the tetrad, $$h_{\hat{\mu }\hat{\nu
                        }}^{(\mathrm{e})}$$, is such that

192 $$\begin{aligned} H_2\sim
                        \mathcal {O}\left( \frac{1}{r^2}\right) , \qquad \mathrm{and} \qquad
                        h_{1}^{(\mathrm{e})} \sim \mathcal {O}\left( \frac{1}{r}\right) ,
                        \end{aligned}$$

so that the terms proportional to these multipoles are of higher order for large *r* and can be neglected. Furthermore, from the transverse traceless condition

193 $$\begin{aligned}
                        h_{\hat{\theta }\hat{\theta }}^{(\mathrm{e})}+ h_{\hat{\phi }\hat{\phi
                        }}^{(\mathrm{e})}= 0 + \mathcal {O}\left( \frac{1}{r^2}\right) ,
                        \end{aligned}$$

we obtain an asymptotic relation between the gauge-invariant functions *K* and *G*

194 $$\begin{aligned}
                        2KY^{{\ell m}}+G\left( \nabla _\theta \nabla _\theta Y^{{\ell
                        m}}+\frac{\nabla _\phi \nabla _\phi Y^{{\ell m}}}{\sin ^2\theta }\right)
                        =\left( 2K-G\varLambda \right) Y^{{\ell m}}\sim \mathcal {O}\left(
                        \frac{1}{r^2}\right) , \end{aligned}$$

where we have used the definition (135) to derive the right-hand side of expression (194). As a result, the asymptotic relation between the two components of the even-parity part of the perturbation metric simply reads

195 $$\begin{aligned} K\sim
                        \frac{\varLambda }{2}\,G+\mathcal {O}\left( \frac{1}{r^2}\right) .
                        \end{aligned}$$

Using now the definitions (156)–(157), we have that asymptotically

196 $$\begin{aligned} \kappa
                        _1 \sim&\, \frac{\varLambda }{2}\,G + r \partial _r G+ \mathcal
                        {O}\left( \frac{1}{r^2}\right) \end{aligned}$$

197 $$\begin{aligned} \kappa
                        _2 \sim&\, -\frac{1}{2}\left( K+r \partial _r K\right) \sim
                        -\frac{\varLambda }{4}\left( G+r \partial _r G\right) + \mathcal {O}\left(
                        \frac{1}{r^2}\right) , \end{aligned}$$

and their linear combination (157) becomes

198 $$\begin{aligned} q_1\sim
                        \frac{rG}{2}\;\varLambda \left( \varLambda -2\right) + \mathcal {O}\left(
                        \frac{1}{r}\right) . \end{aligned}$$

Finally, the asymptotic gauge-invariant even-parity master function reads

199 $$\begin{aligned} {\varPsi
                        }^{(\mathrm{e})}\sim \frac{r q_1}{\varLambda \left[ r(\varLambda -2)+
                        6M\right] }\sim \frac{1}{2}\,rG +\mathcal {O}\left( \frac{1}{r}\right) ,
                        \end{aligned}$$

so that, modulo higher-order terms, the even-parity gravitational-wave amplitudes measured by a distant observer can be written in the compact form

200 $$\begin{aligned} \left(
                        h^{(\mathrm{e})}_{+}-{i}h^{(\mathrm{e})}_{\times }\right) _{{\ell m}}=
                        \frac{1}{r}\sqrt{\frac{(\ell -2)!}{(\ell +2)!}} {\varPsi
                        }^{(\mathrm{e})}_{{\ell m}}\;_{_{-2}}Y^{{\ell m}}(\theta ,\phi )+ \mathcal
                        {O}\left( \frac{1}{r^2}\right) . \end{aligned}$$

### Asymptotic general expressions

It is often convenient to combine the expressions for the asymptotic gravitational-wave amplitudes related to odd and even-parity perturbations into the single expression

201 $$\begin{aligned}
                        h_{+}-{i}h_{\times }=\frac{1}{r}\sum _{\ell ,m} \sqrt{\frac{(\ell
                        +2)!}{(\ell -2)!}} \left( {\varPsi }^{(\mathrm{e})}_{{\ell m}}+ {i}{\varPsi
                        }_{{\ell m}}^{(\mathrm{o})}\right) \;_{_{-2}}Y^{{\ell m}}(\theta ,\phi )+
                        \mathcal {O}\left( \frac{1}{r^2}\right) ,
                        \end{aligned}$$

or, equivalently

202 $$\begin{aligned}
                        h_+-{i}h_{\times }\!=\!\frac{1}{r}\sum _{\ell ,m} \sqrt{\frac{(\ell
                        \!+\!2)!}{(\ell -2)!}} \left( {\varPsi }^{(\mathrm{e})}_{{\ell m}}-{i}
                        \!\int _{-\infty }^tQ^{(\mathrm{o})}_{{\ell m}}(t')dt' \right)
                        \,_{_{-2}}Y^{{\ell m}}(\theta ,\phi ) \!+\! \mathcal {O}\left(
                        \frac{1}{r^2}\right) ,\nonumber \\ \end{aligned}$$

where we have defined $$h_{+}:=h^{(\mathrm{o})}_{+} +
                        h^{(\mathrm{e})}_{+}$$ and $$h_{\times
                        }:=h^{(\mathrm{o})}_{\times } + h^{(\mathrm{e})}_{\times
                        }$$. Note that $$X^{{\ell
                        0}}=0$$ for any value of $$\ell
                        $$, so that in the case of axisymmetry the gravitational-wave signal is proportional to $$W^{\ell
                        0}$$ only.

It is also useful to underline that while expression (201) resembles the corresponding expression (10) of Kawamura and Oohara (2004), it is indeed different. Firstly, because in Kawamura and Oohara (2004) the Moncrief function is adopted for the odd-parity part of the perturbations and hence, modulo a normalisation factor, the function $$\varPsi
                        ^{(\mathrm{o})}$$ appearing there corresponds to our function $$Q^{(\mathrm{o})}$$ [cf. expression (154)]. Secondly, because with this choice for the odd-parity perturbations a time derivative is needed in the asymptotic expression for the gravitational-wave amplitudes [cf. the discussion in the derivation of Eq. (189)]. As a result, expression (10) of Kawamura and Oohara (2004) (which is also missing the distinction between the real and imaginary parts) should really be replaced by expression (202). A similar use of the Moncrief function for the odd-parity part is present also in Shibata et al. (2003), Shibata and Sekiguchi (2003), Shibata and Sekiguchi (2005), where it is employed to calculate the gravitational-wave content of numerically simulated spacetimes.

### Energy and angular momentum losses

Using the expressions derived in the previous sections we can now estimate the energy and angular momentum losses due to gravitational waves propagating outwards to spatial infinity. More specifically, this can be done by using expression (201) and the definition of Isaacson’s stress-energy pseudo-tensor $$\tau _{\mu \nu
                        }$$ for the gravitational-wave field $${\varvec{h}}$$ propagating in the curved background field 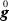 and in a Lorentz gauge (Isaacson 1968; Landau and Lifshitz 1975)

203 $$\begin{aligned} \tau
                        _{\mu \nu } :=\frac{1}{32 \pi } \left\langle \nabla _{\mu } {h}_{\alpha
                        \beta } \nabla _{ \nu } {h}^{\alpha \beta } \right\rangle ,
                        \end{aligned}$$

where the brackets $$\langle \cdots \rangle
                        $$ refer to a spatial average over a length much larger than the typical gravitational wavelength (Isaacson 1968; Misner et al. 1973). The averaged expression (203) is gauge-invariant (Isaacson 1968) and holds in the “limit of high frequency” (or *short-wave* approximation), *i.e.,* whenever the wavelength of the gravitational-wave field is small when compared to the local radius of curvature of the background geometry. In practice, gravitational radiation from isolated systems is of high frequency whenever it is far enough away from its source.

Expression (203) accounts for the amount of energy and momentum carried by the gravitational wave over a certain region of spacetime, but since we are interested in the energy flux as measured by an inertial observer, we need to project the pseudo-tensor on the observer’s locally orthonormal tetrad, where it becomes

204 $$\begin{aligned} \tau
                        _{\hat{\mu } \hat{\nu }} :=\frac{1}{32 \pi } \left\langle \partial
                        _{\hat{\mu }} {\bar{h}}_{\hat{\alpha } \hat{\beta }} \partial _{\hat{\nu }}
                        {\bar{h}}^{\hat{\alpha } \hat{\beta }} \right\rangle ,
                        \end{aligned}$$

with $$\bar{h}_{\hat{\mu
                        }\hat{\nu }} :=h_{\hat{\mu }\hat{\nu }} - \frac{1}{2}h \eta _{\hat{\mu
                        }\hat{\nu }}$$ and *h* being now the trace of $$h_{\hat{\mu }\hat{\nu
                        }}$$. As a result, the energy per unit time and angle carried by the gravitational waves and measured by a stationary observer at large distance is given by

205 $$\begin{aligned}
                        \frac{d^2E}{dtd\varOmega }\!=\!\frac{r^2}{16\pi } \left[ \left( \frac{d
                        {h}_{\hat{\theta }\hat{\phi }}}{dt}\right) ^2\!+\! \frac{1}{4}\left( \frac{d
                        h_{\hat{\theta }\hat{\theta }}}{dt}- \frac{d h_{\hat{\theta }\hat{\phi
                        }}}{dt}\right) ^2\right] \!=\! \frac{r^2}{16\pi }\left( \left| \frac{d
                        {h}_+}{dt}\right| ^2 \!+\!\left| \frac{d h_{\times }}{dt}\right| ^2\right) ,
                        \end{aligned}$$

where the total derivative is made with respect to the asymptotic observer’s time. Integrating (205) over the solid angle, the total power emitted in gravitational waves is then given by

206 $$\begin{aligned}
                        \frac{dE}{dt}= & {} \frac{1}{16\pi }\sum _{\ell ,m}\frac{(\ell
                        +2)!}{(\ell -2)!} \left( \left| \frac{d {\varPsi }^{(\mathrm{e})}_{{\ell
                        m}}}{dt}\right| ^2 +\left| \frac{d {\varPsi }^{(\mathrm{o})}_{{\ell
                        m}}}{dt}\right| ^2\right) , \end{aligned}$$

207 $$\begin{aligned}=
                        & {} \frac{1}{16\pi }\sum _{\ell ,m} \left( \varLambda (\varLambda
                        -2) \left| \frac{d {\varPsi }^{(\mathrm{e})}_{{\ell m}}}{dt}\right| ^2
                        +\frac{\varLambda }{\varLambda -2} \left| \frac{d {\Phi
                        }^{(\mathrm{o})}_{{\ell m}}}{dt} \right| ^2\right) ,
                        \end{aligned}$$

where expression (207) was first presented in Cunningham et al. (1978, 1979).

Note that as discussed at the end of Sect. 5.3, these expressions need to be suitably modified when the energy losses are expressed in terms of the odd-parity Moncrief function $Q^{(\mathrm{o})}$, in which case the energy-loss rate needs to be modified as

208 $$\begin{aligned}
                        \frac{dE}{dt} = \frac{1}{16\pi }\sum _{\ell ,m}\frac{(\ell +2)!}{(\ell -2)!}
                        \left( \left| \frac{d {\varPsi }^{(\mathrm{e})}_{{\ell m}}}{dt}\right| ^2+
                        \left| Q_{{\ell m}}^{(\mathrm{o})}\right| ^2\right) .
                        \end{aligned}$$

Similarly, the angular momentum flux carried away in the form of gravitational waves can also be calculated in terms of the energy-momentum tensor (204). In particular, using spherical coordinates and assuming that the rotation is parametrised by the angle $$\phi
                        $$, we have

209 $$\begin{aligned}
                        \frac{d^2J}{dtd\varOmega }=\frac{r^2}{32\pi } \left\langle \partial _{\phi
                        }\bar{h}_{\hat{\mu }\hat{\nu }} \partial _r\bar{h}^{\hat{\mu }\hat{\nu
                        }}\right\rangle = -\frac{r^2}{16\pi } \left\langle \partial
                        _rh_{{\hat{\theta }}{\hat{\theta }}} \partial _\phi h_{{\hat{\theta
                        }}{\hat{\theta }}} +\partial _rh_{{\hat{\theta }}\hat{\phi }}\partial _{\phi
                        } h_{{\hat{\theta }}\hat{\phi }}\right\rangle .
                        \end{aligned}$$

Since the metric components in the radiation-gauge behave like outgoing spherical waves and since $$h_{{\hat{\theta
                        }}{\hat{\theta }}}=h_+$$ and $$h_{{\hat{\theta
                        }}\hat{\phi }}=h_{\times }$$, the angular momentum carried away in the form of gravitational waves (209) is then expressed as

210 $$\begin{aligned}
                        \frac{d^2J}{dtd\varOmega }=-\frac{r^2}{16\pi }\left( \partial _th_+\partial
                        _\phi \bar{h}_+ + \partial _t h_{\times }\partial _{\phi }\bar{h}_{\times
                        }\right) , \end{aligned}$$

Proceeding in a way similar to the one followed in the calculation of the emitted power, the total angular momentum lost per unit time to gravitational wave reads (Martel and Poisson 2005)

211 $$\begin{aligned}
                        \frac{dJ}{dt}=\frac{1}{16\pi }\sum _{\ell ,m} i m \frac{(\ell +2)!}{(\ell
                        -2)!} \left[ \frac{d\varPsi ^{(\mathrm e)}_{\ell m}}{dt} \left( \bar{\varPsi
                        }_{\ell m}^{(\mathrm{e})}\right) + \frac{d\varPsi ^{(\mathrm o)}_{\ell
                        m}}{dt} \left( \bar{\varPsi }_{\ell m}^{(\mathrm{o})}\right) \right] ,
                        \end{aligned}$$

or, using the Moncrief master function (154) for the odd-parity perturbations (Poisson 2004)

212 $$\begin{aligned}
                        \frac{dJ}{dt}=\frac{1}{16\pi }\sum _{\ell , m} i m \frac{(\ell +2)!}{(\ell
                        -2)!} \left[ \frac{d\varPsi ^{(\mathrm e)}_{\ell m}}{dt} \left( \bar{\varPsi
                        }_{\ell m}^{(\mathrm{e})}\right) + Q_{\ell m}^{(\mathrm o)}\int _{-\infty
                        }^{t} \left( \bar{Q}_{\ell m}^{(\mathrm o)}\right) (t')dt'\right] .
                        \end{aligned}$$

To conclude, we report the expression for the energy spectrum $${dE}/{d\omega
                        }$$, which is readily calculated from Eq. (206) after performing the Fourier transform of the odd and even-parity master functions, i.e., 

213 $$\begin{aligned}
                        \frac{dE}{d\omega }=\frac{1}{16\pi ^2}\sum _{\ell ,m} \frac{(\ell
                        +2)!}{(\ell -2)!}\;\, \omega ^2\left( \left| \widetilde{\varPsi
                        }^{(\mathrm{e})}_{{\ell m}}\right| ^2 +\left| \widetilde{\varPsi
                        }^{(\mathrm{o})}_{{\ell m}}\right| ^2\right) ,
                        \end{aligned}$$

where we have indicated with $$\widetilde{f}(\omega
                        ,r)$$ the Fourier transform of the timeseries *f*(*t*, *r*). Similarly, when using the odd-parity Moncrief function one obtains

214 $$\begin{aligned}
                        \frac{dE}{d\omega }=\frac{1}{16\pi ^2}\sum _{\ell ,m} \frac{(\ell
                        +2)!}{(\ell -2)!}\;\, \left( \omega ^2\left| \widetilde{\varPsi
                        }^{(\mathrm{e})}_{{\ell m}}\right| ^2 +\left|
                        \widetilde{Q}^{(\mathrm{o})}_{{\ell m}} \right| ^2\right) .
                        \end{aligned}$$

### A commonly used convention

A rather popular choice for the gauge-invariant master functions has found successful application in the extraction of the gravitational-wave content of numerically simulated spacetimes (Abrahams and Price 1996b, a; Abrahams et al. 1998; Rupright et al. 1998; Rezzolla et al. 1999a). For instance, the convention discussed below has been implemented in the Cactus computational toolkit (Camarda and Seidel 1999; Allen et al. 1998), a diffused and freely available infrastructure for the numerical solution of the Einstein equations (Allen et al. 1999; Cactus 2016). Numerous tests and applications of this implementation have been performed over the years and we refer the reader to Camarda and Seidel (1999), Allen et al. (1998), Font et al. (2002), Baiotti et al. (2005) for examples both in vacuum and non-vacuum spacetimes.

The reference work for this convention is the one by Abrahams and Price (1996b, 1996a), although a similar approach for the even-parity part of the perturbations was also adopted in previous works (Abrahams et al. 1992; Anninos et al. 1995b). We first note that the coefficients $$c_0$$, $$c_1$$ and $$c_2$$ introduced in Abrahams and Price (1996b, 1996a) are related simply to the multipolar coefficients of the odd-parity part introduced in Sect. 4.2. More specifically, considering that $$c_2 = - 2h =
                        h_2$$, $$c_0 =
                        h_0^{(\mathrm{o})}$$, and $$c_1 =
                        h_1^{(\mathrm{o})}$$, it is then easy to realise that the odd and even-parity master functions $$Q^{\times }_{{\ell
                        m}}$$ and $$Q^{+}_{{\ell
                        m}}$$ defined in Abrahams and Price (1996b, 1996a) are related to the master functions discussed so far through the simple algebraic expressions

215 $$\begin{aligned}
                        Q^{\times }_{{\ell m}}:= & {} \sqrt{\frac{2(\ell +2)!}{(\ell
                        -2)!}}\, Q^{(\mathrm{o})}_{{\ell m}},
                        \end{aligned}$$

216 $$\begin{aligned}
                        Q^{+}_{{\ell m}}:= & {} \sqrt{\frac{2(\ell +2)!}{(\ell -2)!}}\,
                        {\varPsi }^{(\mathrm{e})}_{{\ell m}},
                        \end{aligned}$$

so that the asymptotic expression for the gravitational-wave amplitudes in the two polarisations are given by

217 $$\begin{aligned} h_+=
                        & {} \frac{1}{\sqrt{2}r}\sum _{\ell ,m} \sqrt{\frac{(\ell
                        -2)!}{(\ell +2)!}}\, \left[ Q^+_{{\ell m}}W^{{\ell m}}-\left( \int _{-\infty
                        }^{t} Q^{\times }_{{\ell m}}(t')dt'\right) \frac{\;\;\; X^{{\ell m}}}{\sin
                        \theta }\right] + \mathcal {O}\left( \frac{1}{r^2}\right) ,
                        \end{aligned}$$

218 $$\begin{aligned}
                        h_{\times }= & {} \frac{1}{\sqrt{2}r}\sum _{\ell ,m}
                        \sqrt{\frac{(\ell -2)!}{(\ell +2)!}}\, \left[ Q^+_{{\ell
                        m}}\frac{\;\;\;X^{{\ell m}}}{\sin \theta }+ \left( \int _{-\infty
                        }^{t}Q^{\times }_{{\ell m}}(t')dt'\right) W^{{\ell m}}\right] + \mathcal
                        {O}\left( \frac{1}{r^2}\right) .\nonumber \\
                        \end{aligned}$$

Similarly, expressions (217) and (218) can be combined into a single one

219 $$\begin{aligned}
                        h_{+}-{i}h_{\times }=\frac{1}{\sqrt{2}r}\sum _{\ell ,m}\left( Q^{+}_{{\ell
                        m}} - {i}\int _{-\infty }^{t} Q^{\times }_{{\ell m}}(t')dt'\right)
                        \;_{_{-2}}Y^{{\ell m}}(\theta ,\phi ) + \mathcal {O}\left(
                        \frac{1}{r^2}\right) , \end{aligned}$$

which closely resembles expression (202) and that in its compactness highlights the advantage of the normalisation (215)–(216). We should remark that the notation in Eq. (219) could be misleading as it seems to suggest that $$h_\times
                        $$ is always of odd-parity and $$h_+$$ is always of even-parity. Indeed this is not true in general and in the absence of axisymmetry, i.e., when $$m\ne
                        0$$, both $$h_{\times
                        }$$ and $$h_+$$ are a superposition of odd and even parity modes. It is only for axisymmetric systems, for which only $$m=0$$ modes are present, that $$Q^\times _{\ell
                        m}$$ and $$Q^+_{\ell
                        m}$$ are *real* numbers, that $$h_+$$ is *only* even-parity and $$h_\times
                        $$ is *only* odd-parity.

Also very compact is the expression for the emitted power that, with this convention, simply reads

220 $$\begin{aligned}
                        \frac{dE}{dt}=\frac{1}{32\pi }\sum _{\ell ,m}\left( \left| \frac{d
                        {Q}^{+}_{{\ell m}}}{dt}\right| ^2+ \left| Q^{\times }_{{\ell m}}\right|
                        ^2\right) . \end{aligned}$$

Finally, the flux of linear momentum emitted in gravitational waves in the *i*-direction can be computed from the Isaacson’s energy-momentum tensor and can be written in terms of the two polarization amplitudes as Favata et al. (2004)

221 $$\begin{aligned} \mathcal
                        {F}_i:=\dot{P_i}=\dfrac{r^2}{16\pi }\int d\varOmega \; n_i\left(
                        \dot{h}_+^2+\dot{h}_{\times }^2\right) ,
                        \end{aligned}$$

where $$n_i=x_i/r$$ is the unit radial vector that points from the source to the observer. The calculation of this flux in terms of $$Q^+_{\ell
                        m}$$ and $$Q^\times _{\ell
                        m}$$ can be computed after inserting Eq. (219) in Eq. (221), decomposing $$n_i$$ in spherical harmonics and performing the angular integral. This procedure goes along the lines discussed in Thorne (1980b), where all the relevant formulae are essentially available [cf. Eq. (4.20) there, but see also Sopuerta et al. (2006), Pollney et al. (2007)], so that we only need to adapt them to our notation. More specifically, in Thorne (1980b) the even-parity (or *electric*) multipoles are indicated with $$I_{\ell
                        m}$$ and the odd-parity (or *magnetic*) ones with $$S_{\ell
                        m}$$, and are related to our notation by

222 $$\begin{aligned} ^{(\ell
                        )}I_{\ell m} :=&\, Q^+_{\ell m} ,
                        \end{aligned}$$

223 $$\begin{aligned} ^{(\ell
                        +1)}S_{\ell m} :=&\, Q_{\ell m}^\times ,
                        \end{aligned}$$

where $$^{(\ell )}f_{\ell
                        m}:=d^{\ell } f_{\ell m}/dt^{\ell }$$. From the property $$(Q^{+,\times }_{\ell
                        m})^* = (-1)^mQ_{\ell \,-m}^{+,\times }$$, where the asterisk indicates complex conjugation, we rewrite Eq. (4.20) of Thorne (1980b) in a more compact form. Following Damour and Gopakumar (2006) where the lowest multipolar contribution was explicitly computed in this way, it is convenient to combine the components of the linear momentum flux in the equatorial plane in a complex number as $$\mathcal
                        {F}_x+\mathrm{i}\mathcal {F}_y$$. The multipolar expansion of the flux vector can be written as Pollney et al. (2007)

224 $$\begin{aligned} \mathcal
                        {F}_x+ i \mathcal {F}_y&= \sum _{\ell =2}^{\infty }\sum _{m=0}^{\ell
                        }\delta _m\left( \mathcal {F}_{x}^{\ell m} + i \mathcal {F}_y^{\ell
                        m}\right) , \end{aligned}$$

225 $$\begin{aligned} \mathcal
                        {F}_z&= \sum _{\ell =2}^{\infty }\sum _{m=0}^{\ell }\delta _m
                        \mathcal {F}_{z}^{\ell m} , \end{aligned}$$

where $$\delta
                        _m=1$$ if $$m\ne
                        0$$ and $$\delta
                        _m=1/2$$ if $$m=0$$. A more extended representation in terms of the various multipoles reads

226 $$\begin{aligned} \mathcal
                        {F}_x^{\ell m}+ i \mathcal {F}_y^{\ell m} :=&\,\dfrac{(-1)^m}{16\pi
                        \ell (\ell +1)}\Bigg \{-2 i \bigg [a_{\ell m}^+ \dot{Q}^+_{\ell -m}Q_{\ell
                        \,m-1}^\times +a_{\ell m}^-\dot{Q}^+_{\ell m} Q^\times _{\ell \;-(m+1)}\bigg
                        ] \nonumber \\&+ \sqrt{\dfrac{\ell ^2(\ell -1)(\ell +3)}{(2\ell
                        +1)(2\ell +3)}} \bigg [ b_{\ell m}^- \left( \dot{Q}^+_{\ell
                        \;-m}\dot{Q}^+_{\ell +1\;m-1} + Q_{\ell \;-m}^\times \dot{Q}_{\ell
                        +1\;m-1}^\times \right) \nonumber \\&+ b_{\ell m}^+\left(
                        \dot{Q}^+_{\ell m}\dot{Q}^+_{\ell +1\;-(m+1)}+Q_{\ell m}^\times
                        \dot{Q}^\times _{\ell +1\;-(m+1)}\right) \bigg ] \Bigg \} ,
                        \end{aligned}$$

227 $$\begin{aligned} \mathcal
                        {F}^{\ell m}_z :=&\, \dfrac{(-1)^m}{8\pi \ell (\ell +1)} \bigg \{
                        2m\; \mathfrak {I}\left[ \dot{Q}_{\ell \,-m}^+Q_{\ell m}^{\times }\right]
                        \nonumber \\&+ c_{\ell m}\sqrt{\dfrac{\ell ^2(\ell -1)(\ell
                        +3)}{(2\ell +1)(2\ell +3)}} \mathfrak {R}\left[ \dot{Q}_{\ell
                        \,-m}^+Q^+_{\ell +1\,m}+ Q^\times _{\ell \,-m}\dot{Q}^\times _{\ell
                        +1\,m}\right] \bigg \} , \end{aligned}$$

where

228 $$\begin{aligned} a_{\ell
                        m}^{\pm }:= & {} \sqrt{(\ell \pm m)(\ell \mp m+1)} ,
                        \end{aligned}$$

229 $$\begin{aligned} b_{\ell
                        m}^{\pm }:= & {} \sqrt{(\ell \pm m+1)(\ell \pm m+2}) ,
                        \end{aligned}$$

230 $$\begin{aligned} c_{\ell
                        m}:= & {} \sqrt{(\ell - m+1)(\ell - m+1}) .
                        \end{aligned}$$

Note that here both $$\mathcal {F}_x^{\ell
                        m}$$ and $$\mathcal {F}_y^{\ell
                        m}$$ are *real* numbers and are obtained as the real and imaginary part of the right-hand side of Eq. (226).

### Implementation summary

All of the material presented in the previous sections about the gauge-invariant description of the perturbations of a Schwarzschild black hole has laid the ground for the actual implementation of the Cauchy-perturbative extraction method in numerical-relativity calculations. We recall that the goal of the Cauchy-perturbative method is that of replacing, at least in parts of the three-dimensional numerical domain, the solution of the full nonlinear Einstein’s equations with the solution of a set of simpler linear equations that can be integrated to high accuracy with minimal computational cost. In turn, this provides an unexpensive evolution of the radiative degrees of freedom, the extraction of the gravitational-wave information, and, if needed, the imposition of boundary conditions via the reconstruction of the relevant quantities at the edge of the three-dimensional computational domain.

**Fig. 7:**
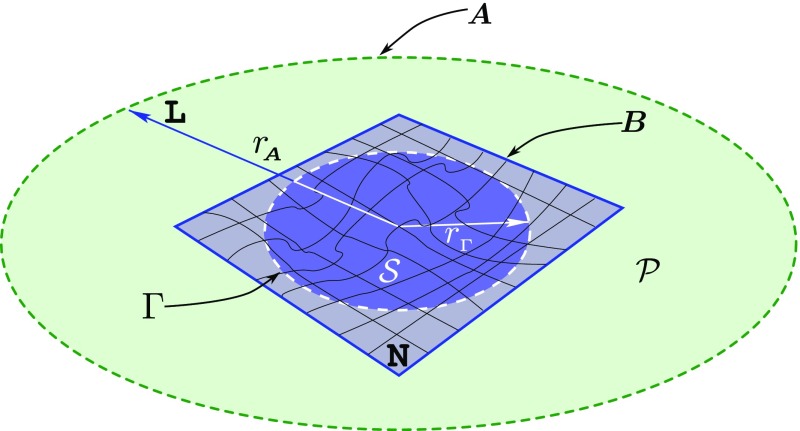
Schematic illustration of a perturbative Cauchy extraction on a single spacelike hypersurface. Schematic picture of the Cauchy-perturbative matching procedure for a spacelike slice of spacetime (one spatial dimension has been suppressed). **N** is the three-dimensional numerical grid on the spacelike hypersurface $\varSigma _t$ in which the full Einstein equations are solved, and $\varvec{B}$ its two-dimensional outer boundary. The interior (*dark shaded*) region $\mathcal {S}$ shows the strong-field highly dynamical region of spacetime fully covered by **N**. $\mathcal {P}$ is the region of spacetime where a perturbative solution can be performed and extends from the 2-sphere $\varGamma$ (of radius $r_{_\varGamma }$) to the 2-sphere $\varvec{A}$ (of radius $r_{_A}$) located in the asymptotically flat region of spacetime. $\mathcal {P}$ is covered entirely by a one-dimensional grid **L** and partially by the three-dimensional grid **N**

In order to do this, it is necessary to determine the region of spacetime where a perturbative approach can be applied. In general, the three-dimensional numerical grid (indicated as **N** in Fig. 7) will comprise an isolated region of spacetime where the gravitational fields are strong and highly dynamical. In this region, indicated as $$\mathcal
                        {S}$$ in Fig. 7, the full nonlinear Einstein equations must be solved. Outside of $$\mathcal
                        {S}$$, however, in what we will refer to as the perturbative region $$\mathcal
                        {P}$$, a perturbative approach is not only possible but highly advantageous. Anywhere in the portion of $$\mathcal
                        {P}$$ covered by **N** we can place a two-dimensional surface, indicated as $$\varGamma
                        $$ in Fig. 7, which will serve as the surface joining numerically the highly dynamical strong-field region $$\mathcal
                        {S}$$ and the perturbative one $$\mathcal
                        {P}$$. In practice, it is easier to choose this surface to be a 2-sphere of radius $$r_{_\varGamma
                        }$$, where $$r_{_\varGamma
                        }$$ can either be the local coordinate radius, the corresponding Schwarzschild radial coordinate, or some more sophisticated radial coordinate deduced from the local values of the metric (cf. discussion in Sect. 4.2).^10^ It is important to emphasize that the 2-sphere $$\varGamma
                        $$ need not be in a region of spacetime where the gravitational fields are weak or the curvature is small. In contrast to approaches which matched Einstein’s equations onto a Minkowski background (Abrahams and Evans 1988, 1990), the matching is here made on a Schwarzschild background, so that the only requirement is that the spacetime outside of $$\mathcal
                        {S}$$ approaches a Schwarzschild one. Of course, even in the case of a binary black-hole merger, it will be possible to find a region of spacetime, sufficiently distant from the black holes, where this requirement is met to the desired precision (Price and Pullin 1994; Abrahams and Cook 1994; Abrahams and Price 1996b, a; Abrahams et al. 1995).

In a practical implementation of the Cauchy-perturbative approach (Rupright et al. 1998; Rezzolla et al. 1999a), a numerical code provides the solution to the full nonlinear Einstein equations everywhere in the three-dimensional grid **N** except at its outer boundary surface $$\varvec{B}$$. At the extraction 2-sphere $$\varGamma
                        $$, a different code (i.e., the perturbative module) “extracts” the gravitational wave information and transforms it into a set of multipole amplitudes which are chosen to depend only on the radial and time coordinates of the background Schwarzschild metric (Rupright et al. 1998; Rezzolla et al. 1999a).

In this way, two of the three spatial dimensions of the problem are suppressed and the propagation of gravitational waves on a curved background is reduced to a one-dimensional problem. During each timestep, information about the gravitational field read-off at $$\varGamma
                        $$ is propagated by the perturbative module out to the 2-sphere $$\varvec{A}$$ in the asymptotic flat region of spacetime. This is done by solving a set of coupled one-dimensional linear differential equations (one for each of the multipoles extracted at $$\varGamma
                        $$) on the one-dimensional grid **L** covering the perturbative region $$\mathcal
                        {P}$$ and ranging between $$r_{_\varGamma
                        }$$ and $$r_{_A} \gg r_{_\varGamma
                        }$$. From a computational point of view, this represents an enormous advantage: with a few straightforward transformations, the computationally expensive three-dimensional evolution of the gravitational waves via the nonlinear Einstein equations is replaced with a set of one-dimensional linear equations that can be integrated to high accuracy with minimal computational cost. Although linear, these equations account for all of the effects of wave propagation in a curved spacetime and, in particular, automatically incorporate the effects of backscatter off the curvature.

**Fig. 8:**
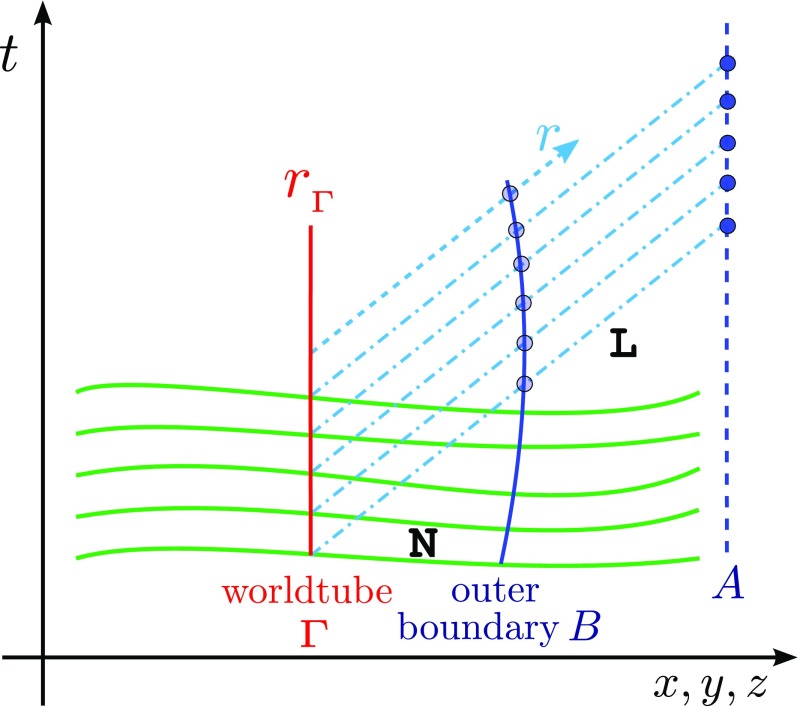
Schematic illustration of a perturbative Cauchy extraction. The Cauchy evolution is shown with *green slices*, comprising hypersurfaces $\varSigma _t$ on each of which is constructed a three-dimensional grid **N** The outer-boundary surface *B* of the three-dimensional grid is shown in *dark blue*, and is subject to a boundary condition that excludes incoming gravitational waves. Data from the Cauchy evolution on the worldtube $\varGamma$ supplies boundary data to the perturbative equations, whose solution leads to the gravitational waves on the asymptotic boundary *A*. Note the difference between the asymptotic values of the gravitational waves extracted at *A* (*filled blue circles*) with the boundary values that can instead be injected on *B*

Note that as a result of this construction, (and as shown in Fig. 7), the perturbative region $$\mathcal
                        {P}$$ is entirely covered by a one-dimensional grid L and only partially by a three-dimensional grid in the complement to $$\mathcal
                        {S}$$ in **N**. The overlap between these two grids is essential. In fact, the knowledge of the solution on $$\mathcal
                        {P}$$ allows the perturbative approach to provide boundary conditions at the outer boundary surface $$\varvec{B}$$ and, if useful, Dirichlet data on every gridpoint of **N** outside the strong region $$\mathcal
                        {S}$$. This is also illustrated in Fig. 8, which represents a one-dimensional cut of Fig. 7, and highlights the difference between the asymptotic values of the gravitational waves extracted at the boundary *A* of the one-dimensional grid (filled blue circles) with and the boundary values that can be instead specified (i.e., “injected”) on the outer boundary surface *B* of the three-dimensional grid.

The freedom to specify boundary data on a 2-surface of arbitrary shape as well as on a whole three-dimensional region of **N** represents an important advantage of the perturbative approach over similar approaches to the problem of gravitational-wave extraction and imposition of boundary conditions.

In what follows we briefly review the main steps necessary for the numerical implementation of the Cauchy-perturbative approach in a numerical-relativity code solving the Einstein equations in a 3+1 split of spacetime. This approach, which follows closely the discussion made in Rupright et al. (1998), Rezzolla et al. (1999a), basically consists of three steps: *(1) extraction* of the independent multipole amplitudes on $$\varGamma
                        $$; *(2) evolution* of the radial wave equations (247)–(249) on **L** out to the distant wave zone; * (3) reconstruction* of $$K_{ij}$$ and $$\partial _t
                        K_{ij}$$ at specified gridpoints at the outer boundary of **N**. We next discuss in detail each of these steps.

#### Perturbative expansion

The first step is to linearize the Einstein equations around a static Schwarzschild background by separating the gravitational quantities of interest into background (denoted by a tilde) and perturbed parts: the three-metric $$\gamma _{i j} =
                           \widetilde{\gamma }_{i j} + h_{i j}$$, the extrinsic curvature $$K_{i j} =
                           \widetilde{K}_{i j} + \kappa _{i j}$$, the lapse $$N = \widetilde{N} +
                           \alpha $$, and the shift vector $$\beta ^i =
                           \widetilde{\beta }^i + v^i$$. Note that the large majority of modern numerical-relativity codes implement the BSSNOK (Nakamura et al. 1987; Shibata and Nakamura 1995; Baumgarte and Shapiro 1999) or the CCZ4 (Alic et al. 2012) formulation of the Einstein equations. As mentioned in Sect. 3.2, in these formulations, the extrinsic curvature tensor is not evolved directly, but rather a traceless tensor extrinsic curvature tensor related to a conformal decomposition of the three-metric (Alcubierre 2008; Bona et al. 2009; Baumgarte and Shapiro 2010; Gourgoulhon 2012; Rezzolla and Zanotti 2013). Of course, also in these formulations it is possible to reconstruct the physically related extrinsic curvature tensor $$K_{ij}$$ and we will therefore continue to make use of $$K_{ij}$$ hereafter.

In Schwarzschild coordinates $$(t, r, \theta , \phi
                           )$$, the background quantities are given by

231 $$\begin{aligned}
                           \widetilde{N} =&\left( 1 - \frac{2M}{r}\right) ^{1/2} ,
                           \end{aligned}$$

232 $$\begin{aligned}
                           \widetilde{g}_{i j} \, dx^i \, dx^j = \,&\widetilde{N}^{-2} dr^2
                           + r^2 (d\theta ^2 + \sin ^2\theta d\phi ^2) ,
                           \end{aligned}$$

233 $$\begin{aligned}
                           \widetilde{\beta }^i = \,&0 ,
                           \end{aligned}$$

234 $$\begin{aligned}
                           \widetilde{K}_{i j} = \,&0 ,
                           \end{aligned}$$

while the perturbed quantities have arbitrary angular dependence. The background quantities satisfy the dynamical equations $$\partial _t
                           \widetilde{\gamma }_{i j} = 0$$, $$\partial _t
                           \widetilde{N} = 0$$, and thus remain constant for all time. The perturbed quantities, on the other hand, obey the following evolution equations

235 $$\begin{aligned}
                           \partial _t h_{i j} =&\, -2 \widetilde{N} \kappa _{i j} + 2
                           \widetilde{\nabla }_{(i} v_{j)} ,
                           \end{aligned}$$

236 $$\begin{aligned}
                           \partial _t \alpha =&\, v^i \widetilde{\nabla }_i \widetilde{N} -
                           \widetilde{N}^2 \kappa , \end{aligned}$$

237 $$\begin{aligned}
                           \widetilde{N}^{-1}\partial _t^2\kappa _{ij} -
                           \widetilde{N}\widetilde{\nabla }^k\widetilde{\nabla }_k\kappa _{ij}
                           =&\, - 4 \widetilde{\nabla }_{(i}\kappa ^k_{\ j)}
                           \widetilde{\nabla }_k \widetilde{N} + \widetilde{N}^{-1} \kappa _{ij}
                           \widetilde{\nabla }^k \widetilde{N} \widetilde{\nabla }_k \widetilde{N} +
                           3 \widetilde{\nabla }^k \widetilde{N} \widetilde{\nabla }_k \kappa
                           _{ij}\nonumber \\&+ \kappa _{ij} \widetilde{\nabla }^k
                           \widetilde{\nabla }_k \widetilde{N} - 2 \kappa ^k_{\;(i}
                           \widetilde{\nabla }_{j)} \widetilde{\nabla }_k \widetilde{N} - 2
                           \widetilde{N}^{-1} \kappa ^k_{\;(i} \widetilde{\nabla }_{j)}
                           \widetilde{N} \widetilde{\nabla }_k \widetilde{N} \nonumber \\&+
                           2 \kappa \widetilde{\nabla }_i \widetilde{\nabla }_j \widetilde{N}+ 4
                           \partial _{(i} \kappa \partial _{j)} \widetilde{N} + 2 \widetilde{N}^{-1}
                           \kappa \widetilde{\nabla }_i \widetilde{N} \widetilde{\nabla }_j
                           \widetilde{N}\nonumber \\&- 2 \widetilde{N} \widetilde{R}_{k(i}
                           \kappa ^k_{\ j)} - 2 \widetilde{N} \widetilde{R}_{kijm} \kappa ^{km} ,
                           \end{aligned}$$

where $$\kappa :=\kappa ^i_{\
                           i}$$ and, as mentioned above, the tilde denotes a spatial quantity defined in terms of the background metric, $$\widetilde{\gamma
                           }_{i j}$$. Note that the wave equation for $$\kappa _{i
                           j}$$ involves only the background lapse and curvature.

Next, it is possible to simplify the evolution equation (237) by separating out the angular dependence, thus reducing it to a set of one-dimensional equations. This is accomplished by expanding the extrinsic curvature in Regge–Wheeler tensor spherical harmonics (Regge and Wheeler 1957) and substituting this expansion into (237). Using the notation of Moncrief (1974) we express the expansion as

238 $$\begin{aligned}
                           \kappa _{i j} =&\, a_\times (t,r) (\hat{e}_1)_{i j} + r b_\times
                           (t,r) (\hat{e}_2)_{i j} + \widetilde{N}^{-2} a_+ (t,r) (\hat{f}_2)_{i j}
                           + r b_+ (t,r) (\hat{f}_1)_{i j} \nonumber \\&+r^2 c_+ (t,r)
                           (\hat{f}_3)_{i j} + r^2 d_+ (t,r) (\hat{f}_4)_{i j} ,
                           \end{aligned}$$

where $$(\hat{e}_1)_{i
                           j},\ldots ,(\hat{f}_4)_{i j}$$ are the Regge–Wheeler harmonics, which are functions of $$(\theta ,\phi
                           )$$ and have suppressed angular indices $$(\ell
                           ,m)$$ for each mode. Explicit expressions for these tensors are given in section “Regge–Wheeler harmonics” in “Appendix 2”.

The odd-parity multipoles ($$a_\times
                           $$ and $$b_\times
                           $$) and the even-parity multipoles ($$a_+$$, $$b_+$$, $$c_+$$, and $$d_+$$) also have suppressed indices for each angular mode and there is an implicit sum over all modes in (238). The six multipole amplitudes correspond to the six components of $$\kappa _{i
                           j}$$. However, using the linearized momentum constraints

239 $$\begin{aligned}
                           \widetilde{\nabla }_j (\kappa ^j_{\ i} - \delta ^j_{\ i} \kappa ) = 0 ,
                           \end{aligned}$$

we reduce the number of independent components of $$\kappa _{i
                           j}$$ to three. An important relation is also obtained through the wave equation for $$\kappa
                           $$, whose multipole expansion is simply given by $$\kappa = h(t,r)
                           Y_{_{\ell m}}$$. Using this expansion, in conjunction with the momentum constraints (239), we derive a set of radial constraint equations which relate the dependent amplitudes $$(b_\times )_{_{\ell
                           m}}$$, $$(b_+)_{_{\ell
                           m}}$$, $$(c_+)_{_{\ell
                           m}}$$ and $$(d_+)_{_{\ell
                           m}}$$ to the three independent amplitudes $$(a_\times )_{_{\ell
                           m}}$$, $$(a_+)_{_{\ell
                           m}}$$, $$(h)_{_{\ell
                           m}}$$

240 $$\begin{aligned}
                           (b_\times )_{_{\ell m}} =&-\frac{1}{(\ell +2)(\ell -1)}
                           [(1+3\widetilde{N}^2) + 2\widetilde{N}^2 r\;\partial _r]\; (a_\times
                           )_{_{\ell m}} , \end{aligned}$$

241 $$\begin{aligned}
                           (b_+)_{_{\ell m}} =&\frac{1}{\ell (\ell +1)} [(3+r\partial _r)\;
                           (a_+)_{_{\ell m}} - (1+r\partial _r)\; (h)_{_{\ell m}}] ,
                           \end{aligned}$$

242 $$\begin{aligned}
                           (c_+)_{_{\ell m}} =&\frac{1}{2(\ell +2)(\ell -1)} \{2(1-\ell
                           -\ell ^2)\; (a_+)_{_{\ell m}} - 2\; (h)_{_{\ell m}} \nonumber
                           \\&+ \ell (\ell +1) [(1+5\widetilde{N}^2) + 2\widetilde{N}^2 r\;
                           \partial _r]\; (b_+)_{_{\ell m}}\} ,
                           \end{aligned}$$

243 $$\begin{aligned}
                           (d_+)_{_{\ell m}} =&\frac{1}{\ell (\ell +1)}[(a_+)_{_{\ell m}} +
                           2 (c_+)_{_{\ell m}} - (h)_{_{\ell m}}] ,
                           \end{aligned}$$

for each $$(\ell
                           ,m)$$ mode.

#### Extraction

Taking the extraction 2-sphere $$\varGamma
                           $$ as the surface joining the evolution of the highly dynamical, strong field region (dark shaded area of Fig. 7) and the perturbative regions (light shaded areas), at each timestep, $$K_{i
                           j}$$ and $$\partial _t K_{i
                           j}$$ are computed on **N** as a solution to Einstein’s equations. Assuming that **N** uses topologically Cartesian coordinates,^11^ the Cartesian components of these tensors are then transformed into their equivalents in a spherical coordinate basis and their traces are computed using the inverse background metric, i.e., $$H = \widetilde{\gamma
                           }^{i j} K_{i j}$$, $$\partial _t H =
                           \widetilde{\gamma }^{i j} \partial _t K_{i j}$$. From the spherical components of $$K_{i
                           j}$$ and $$\partial _t K_{i
                           j}$$, the independent multipole amplitudes for each $$(\ell
                           ,m)$$ mode are then derived by an integration over the 2-sphere:

244 $$\begin{aligned}&\displaystyle (a_\times
                           )_{_{\ell m}} = \frac{1}{\ell (\ell +1)} \int \frac{1}{\sin \theta }
                           \left[ K_{r \phi } \, \partial _\theta - K_{r \theta } \, \partial _\phi
                           \right] \, {Y^*_{_{\ell m}}} \, d\varOmega ,
                           \end{aligned}$$

245 $$\begin{aligned}&\displaystyle (a_+)_{_{\ell m}}
                           =\int \widetilde{N}^2 \, K_{r r} \, Y^*_{\ell m} \, d\varOmega ,
                           \end{aligned}$$

246 $$\begin{aligned}&\displaystyle (h)_{_{\ell m}} =
                           \int \, H \, Y^*_{\ell m} d\varOmega .
                           \end{aligned}$$

Their time derivatives are computed similarly. Rather than performing the integrations (244)–(246) using spherical polar coordinates, it is useful to cover $$\varGamma
                           $$ with two stereographic coordinate “patches”. These are uniformly spaced two-dimensional grids onto which the values of $$K_{i
                           j}$$ and $$\partial _t K_{i
                           j}$$ are interpolated using either a three-linear or a three-cubic polynomial interpolation scheme. As a result of this transformation, the integrals over the 2-sphere in (244)–(246) are computed avoiding polar singularities (see discussion in section “Stereographic coordinates” in “Appendix 2”).

#### Perturbative evolution

Substituting (238) into (237) and using the constraint equations (240), we obtain a set of linearized radial wave equations for each independent amplitude. For each $$(\ell
                           ,m)$$ mode we have one odd-parity equation

247 $$\begin{aligned}
                           \Biggl \{ \partial ^2_t - \widetilde{N}^4 \partial ^2_r -
                           \frac{2}{r}\widetilde{N}^2 \partial _r - \frac{2 M}{r^3} \left( 1 -
                           \frac{3 M}{2 r} \right) + \widetilde{N}^2 \left[ \frac{\ell (\ell
                           +1)}{r^2} - \frac{6 M}{r^3} \right] \Biggr \} (a_\times )_{_{\ell m}} = 0
                           , \end{aligned}$$

and two coupled even-parity equations,

248 $$\begin{aligned}&\Biggl [ \partial ^2_t -
                           \widetilde{N}^4 \partial ^2_r - \frac{6}{r}\widetilde{N}^4 \partial _r
                           +\widetilde{N}^2 \frac{\ell (\ell +1)}{r^2} - \frac{6}{r^2}
                           +\frac{14M}{r^3}-\frac{3M^2}{r^4} \Biggr ] (a_+)_{_{\ell m}} \nonumber
                           \\&\quad +\Biggl [\frac{4}{r} \widetilde{N}^2 \left( 1
                           -\frac{3M}{r}\right) \partial _r + \, \frac{2}{r^2} \left( 1 -
                           \frac{M}{r} - \frac{3M^2}{r^2}\right) \Biggr ] (h)_{_{\ell m}} = 0 ,
                           \end{aligned}$$

249 $$\begin{aligned}&\Biggl [ \partial ^2_t -
                           \widetilde{N}^4 \partial ^2_r - \frac{2}{r}\widetilde{N}^2 \partial _r +
                           \widetilde{N}^2 \frac{\ell (\ell +1)}{r^2} + \frac{2 M}{r^3} - \frac{7
                           M^2}{r^4} \Biggr ] (h)_{_{\ell m}} \nonumber \\&\quad - \frac{2
                           M}{r^3} \left( 3 - \frac{7 M}{r}\right) (a_+)_{_{\ell m}} = 0 .
                           \end{aligned}$$

These equations are related to the standard Regge–Wheeler and Zerilli equations (Regge and Wheeler 1957; Zerilli 1970a).

Once the multipole amplitudes, $$(a_\times )_{_{\ell
                           m}}$$, $$(a_+)_{_{\ell
                           m}}$$, $$(h)_{_{\ell
                           m}}$$ and their time derivatives are computed on $$\varGamma
                           $$ in the timeslice $$t=t_0$$, they are imposed as inner boundary conditions on the one-dimensional grid. Using a suitably accurate integration scheme, the radial wave equations (247)–(249) can be evolved for each $$(\ell ,
                           m)$$ mode forward to the next timeslice at $$t=t_1$$. The outer boundary of the one-dimensional grid is always placed at a distance large enough that background field and near-zone effects are unimportant, and a radial Sommerfeld condition for the wave equations (247)–(249) can be imposed there. The evolution equations for $$h_{i
                           j}$$ [Eq. (235)] and $$\alpha
                           $$ [Eq. (236)] can also be integrated using the data for $$K_{i
                           j}$$ computed in this region. Note also that because $$h_{i
                           j}$$ and $$\alpha
                           $$ evolve along the coordinate time axis, these equations need only be integrated in the region in which their values are desired, not over the whole region **L**.

Of course, the initial data on **L** must be consistent with the initial data on **N**, and this can be determined by applying the aforementioned extraction procedure to the initial data set at each gridpoint of **L** in the region of overlap with **N**. In the latter case, initial data outside the overlap region can be set by considering the asymptotic fall-off of each variable.

#### Reconstruction

An important side product of the evolution step discussed above is that outer boundary values for **N** can now be computed, although, to the best of our knowledge, this procedure has not been implemented yet as a way to obtain outer boundary conditions. In particular, for codes using the BSSNOK (Nakamura et al. 1987; Shibata and Nakamura 1995; Baumgarte and Shapiro 1999) or the CCZ4 (Alic et al. 2012) formulation of the Einstein equations, it is sufficient to provide boundary data only for $$K_{i
                           j}$$, since the interior code can calculate $$\gamma _{i
                           j}$$ at the outer boundary by integrating in time the boundary values for $$K_{i
                           j}$$.

In order to compute $$K_{i
                           j}$$ at an outer boundary point of **N** (or any other point in the overlap between **N** and $$\mathcal
                           {P}$$), it is necessary to reconstruct $$K_{i
                           j}$$ from the multipole amplitudes and tensor spherical harmonics. The Schwarzschild coordinate values $$(r,\theta ,\phi
                           )$$ of the relevant gridpoint are first determined. Next, $$(a_\times )_{_{\ell
                           m}}$$, $$(a_+)_{_{\ell
                           m}}$$, and $$(h_{_{\ell
                           m}})$$ for each $$(\ell
                           ,m)$$ mode are interpolated to the radial coordinate value of that point. The dependent multipole amplitudes $$(b_\times )_{_{\ell
                           m}}$$, $$(b_+)_{_{\ell
                           m}}$$, $$(c_+)_{_{\ell
                           m}}$$, and $$(d_+)_{_{\ell
                           m}}$$ are then computed using the constraint equations (240). Finally, the Regge–Wheeler tensor spherical harmonics $$(\hat{e}_1)_{i
                           j}$$–$$(\hat{f}_4)_{i
                           j}$$ are computed for the angular coordinates $$(\theta ,\phi
                           )$$ for each $$(\ell
                           ,m)$$ mode and the sum in Eq. (238) is performed. This leads to the reconstructed component of $$\kappa _{i
                           j}$$ (and therefore $$K_{i
                           j}$$). A completely analogous algorithm can be used to reconstruct $$\partial _t K_{i
                           j}$$ in formulations in which this information is needed.

It is important to emphasize that this procedure allows one to compute $$K_{i
                           j}$$ at any point of **N** which is covered by the perturbative region. As a result, the numerical module can reconstruct the values of $$K_{ij}$$ and $$\partial _t K_{i
                           j}$$ on a 2-surface of arbitrary shape, or any collection of points outside of $$\varGamma
                           $$.

## Gravitational waves in the characteristic approach

The formalism for expressing Einstein’s equations as an evolution system based on characteristic, or null-cone, coordinates is based on work originally due to Bondi (1960) and Bondi et al. (1962) for axisymmetry, and extended to the general case by Sachs (1962). The formalism is covered in the review by Winicour (2005), to which the reader is referred for an in-depth discussion of its development and the associated literature.

Most work on characteristic evolution uses, or is an adpatation of, a finite difference code that was originally developed at the University of Pittsburgh and has become known as the PITT null code. The early work that eventually led to the PITT code was for the case of axisymmetry (Isaacson et al. 1983; Bishop et al. 1990; Gómez et al. 1994), and a general vacuum code was developed in the mid-1990s (Bishop et al. 1996b, 1997b; Lehner 1998, 1999, 2001). Subsequently, the code was extended to the non-vacuum case (Bishop et al. 1999b, 2005), and code adaptations in terms of variables, coordinates and order of accuracy have been investigated (Gómez 2001; Gómez and Frittelli 2003; Reisswig et al. 2007, 2013a). Spectral, rather than finite difference, implementations have also been developed, for both the axially symmetric case (de Oliveira and Rodrigues 2009) and in general (Handmer and Szilágyi 2015). One potential difficulty, although in practice it has not been important in characteristic extraction, is the development of caustics during the evolution, and algorithms to handle the problem have been proposed (Stewart and Friedrich 1982; Corkill and Stewart 1983). There are also approaches that use outgoing null cones but for which the coordinates are not Bondi–Sachs (Bartnik 1997; Bartnik and Norton 2000).

Shortly after the publication of the Bondi and Bondi–Sachs metrics and formalism, the idea of conformal compactification was introduced. This led to the well-known asymptotic description of spacetime, and the definitions of asymptotic flatness, past, future and spacelike infinity ($$I^+,I^-,I^0$$), and of past and future null infinity ($${\mathcal {J}}^-,\mathcal
                     {J}^+$$) (Penrose 1963); see also Penrose (1964, 1965b) and Tamburino and Winicour (1966); and the reviews by Adamo et al. (2012) and Frauendiener (2004). The key result is that gravitational radiation can be defined unambiguously in an asymptotically flat spacetime only at null infinity. The waves may be expressed in terms of the Bondi news $${{\mathcal
                     {N}}}$$ (see Eq. (271) below), the Newman–Penrose quantity $$\psi
                     _4$$, or the wave strain $$(h_+,h_\times
                     )$$.

After a characteristic code has been run using a compactified radial coordinate as in Eq. (259), the metric is known at $$\mathcal
                     {J}^+$$, and so it would seem to be straightforward to calculate the emitted gravitational radiation. Unfortunately, this is not in general the case because of gauge, or coordinate freedom, issues. The formulas do take a very simple form when expressed in terms of coordinates that satisfy the Bondi gauge condition in which the asymptotic flatness property is obviously satisfied, and for which conditions set at $$\mathcal
                     {J}^+$$ are propagated inwards along radial null geodesics. However, in a numerical simulation that is not the case: coordinate conditions are fixed on an extraction worldtube (in the case of characteristic extraction), or perhaps on a worldline (Siebel et al. 2003) or ingoing null hypersurface, and then propagated outwards to $$\mathcal
                     {J}^+$$. The result is that the geometry at and near $$\mathcal
                     {J}^+$$ may appear very different to one that is foliated by spherical 2-surfaces of constant curvature. Of course, the Bondi gauge and the general gauge are related by a coordinate transformation, and formulas for $${{\mathcal
                     {N}}}$$ and $$\psi
                     _4$$ are obtained by constructing the transformation.

An explicit formula in the general gauge for the news was obtained in Bishop et al. (1997b) (“Appendix 2”); and a calculation of $$\psi
                     _4$$ was reported in Babiuc et al. (2009), but the formula produced was so lengthy that it was not published. These formulas have been used in the production of most waveforms calculated by characteristic codes. An alternative approach, in which the coordinate transformation is explicit, rather than partially implicit, was suggested (Bishop and Deshingkar 2003) but has not been further used or developed. Recently, a formula for the wave strain $$(h_+,h_\times
                     )$$, which is the quantity used in the construction of templates for gravitational-wave data analysis, was derived (Bishop and Reisswig 2014). An important special case is that of the linearized approximation, in which deviations from the Bondi gauge are small. The resulting formulas for $${{\mathcal
                     {N}}}$$, $$\psi
                     _4$$ and $$(h_+,h_\times
                     )$$, are much simpler and so much easier to interpret than in the general case. Further these formulas are widely used because the linearized approximation often applies to the results of a waveform computation in a realistic scenario.

We set the context for this section by summarizing the Einstein equations in characteristic coordinates, and outlining the characteristic evolution procedure. The focus of this section is formulas for gravitational waves, and we next present the formulas in the simplest case, when the coordinates satisfy the Bondi gauge conditions. Much of the remainder of the section will be devoted to formulas for gravitational waves in the general gauge, and will include a discussion of conformal compactification. This section makes extensive use of spin-weighted spherical harmonics and the eth formalism, which topics are discussed in “Appendix 2”.

### The Einstein equations in Bondi–Sachs coordinates

We start with coordinates based upon a family of outgoing null hypersurfaces. Let *u* label these hypersurfaces, $$\phi
                        ^{^{_A}}$$ $$(A=2,3)$$ be angular coordinates labelling the null rays, and *r* be a surface area coordinate. In the resulting $$x^\alpha =(u,r,\phi
                        ^{^{_A}})$$ coordinates, the metric takes the Bondi–Sachs form

250 $$\begin{aligned} ds^2=
                        & {} -\left( e^{2\beta }(1 + W_c r)
                        -r^2h_{_{AB}}U^{^{_A}}U^{^{_{_{B}}}}\right) \, du^2 \nonumber \\&-
                        2e^{2\beta } \, du \, dr -2r^2 h_{_{AB}}U^{^{_{_{B}}}} \, du \, d\phi
                        ^{^{_A}} + r^2h_{_{AB}} \, d\phi ^{^{_A}} \, d\phi ^{^{_{_{B}}}},
                        \end{aligned}$$

where $$h^{^{_{AB}}}h_{BC}=\delta
                        ^{^{_A}}_C$$ and $$\det (h_{_{AB}})=\det
                        (q_{_{AB}})$$, with $$q_{_{AB}}$$ a metric representing a unit 2-sphere; $$W_c$$ is a normalized variable used in the code, related to the usual Bondi–Sachs variable *V* by $$V=r+W_c
                        r^2$$. It should be noted here that different references use various notations for what is here denoted as $$W_c$$, and in particular (Bishop et al. 1997b) uses *W* with $$W
                        :=r^2W_c$$. As discussed in Sect. 1, we represent $$q_{_{AB}}$$ by means of a complex dyad $$q_{_{A}}$$, then $$h_{_{AB}}$$ can be represented by its dyad component $$J:=h_{_{AB}}q^{^{_A}}q^{^{_{_{B}}}}/2$$. We also introduce the fields $$K :=\sqrt{1+J
                        \bar{J}}$$ and $$U
                        :=U^{^{_A}}q_{_{A}}$$. The spin-weight *s* of a quantity is defined and discussed in section “Spin-weighted fields” in “Appendix 2”; for the quantities used in the Bondi–Sachs metric

251 $$\begin{aligned} s(W_c)=
                        & {} s(\beta )=0,\qquad s(J)=2,\qquad s(\bar{J})=-2, \nonumber \\
                        s(K)= & {} 0, \qquad s(U)=1,\qquad s(\bar{U})=-1.
                        \end{aligned}$$

We would like to emphasize two matters: (a) The metric Eq. (250) applies quite generally, and does not rely on the spacetime having any particular properties. (b) There are many different metrics of the form Eq. (250) that describe a given spacetime, and changing from one to another is known as a gauge transformation (about which more will be said later).

The form of the Einstein equations for the general Bondi–Sachs metric has been known for some time, but it was only in 1997 (Bishop et al. 1997b) that they were used for a numerical evolution. [see also Gómez and Frittelli 2003 for an alternative semi-first-order form that avoids second angular derivatives ($$\eth ^2, \bar{\eth }^2,
                        \bar{\eth }\eth $$)]. The equations are rather lengthy, and only the hypersurface and evolution equations are given in that paper, in an “Appendix”.^12^ See also section “Computer algebra” in “Appendix 3”. Here, in order to make the discussion of the Einstein equations precise but without being overwhelmed by detail, we give the equations in vacuum in the linearized case, that is when any second-order term in the quantities $$J, \beta , U,
                        W_c$$ can be ignored. The Einstein equations are categorized into three classes, hypersurface, evolution, and constraint. The hypersurface equations are

252 $$\begin{aligned}
                        R_{11}:&\quad \frac{4}{r}\partial _r \beta =0,
                        \end{aligned}$$

253 $$\begin{aligned}
                        q^{^{_A}} R_{1A}:&\quad \frac{1}{2r} \left( 4 \eth \beta - 2 r \eth
                        \partial _r\beta + r \bar{\eth } \partial _r J +r^3 \partial ^2_r U +4 r^2
                        \partial _r U \right) = 0, \end{aligned}$$

254 $$\begin{aligned}
                        h^{^{_{AB}}} R_{_{AB}}:&\quad (4-2\eth \bar{\eth }) \beta
                        +\frac{1}{2}(\bar{\eth }^2 J + \eth ^2\bar{J}) +\frac{1}{2r^2}\partial
                        _r(r^4\eth \bar{U}+r^4\bar{\eth }U)\nonumber \\&\quad -2 r^2
                        \partial _r W_{c} -4 r W_c =0. \end{aligned}$$

The evolution equation is

255 $$\begin{aligned}
                        q^{^{_A}} q^{^{_{_{B}}}} R_{_{AB}}: \;\; -2\eth ^2\beta + \partial _r(r^2
                        \eth U) - 2r \partial _r J - r^2 \partial ^2_r J +2 r \partial _r\partial _u
                        (rJ)= 0. \end{aligned}$$

The constraint equations are Reisswig et al. (2007)

256 $$\begin{aligned}
                        R_{00}:&\quad \frac{1}{2r^2} \bigg ( r^3 \partial ^2_r
                        W_{c}+4r^2\partial _r W_{c}+2rW_c+r\eth \bar{\eth } W_c +2 \eth \bar{\eth }
                        \beta \nonumber \\&\quad -4 r \partial _u\beta - r^2 \partial
                        _u(\eth \bar{U} + \bar{\eth }U)+2 r^2 \partial _u W_{c} \bigg ) = 0,
                        \end{aligned}$$

257 $$\begin{aligned}
                        R_{01}:&\quad \frac{1}{4r^2} \bigg (2r^3 \partial ^2_r
                        W_{c}+8r^2\partial _r W_{c}+4rW_c+4 \eth \bar{\eth }\beta -\partial
                        _r(r^2\eth \bar{U}+r^2\bar{\eth }U)\bigg )=0,
                        \end{aligned}$$

258 $$\begin{aligned}
                        q^{^{_A}} R_{0A}:&\quad \frac{1}{4} \bigg ( 2r \eth \partial _r
                        W_{c}+2 \eth W_c+ 2 r(4 \partial _r U + r \partial _r^2 U)+4 U +(\eth
                        \bar{\eth }U-\eth ^2\bar{U})\nonumber \\&\quad +2 \bar{\eth
                        }\partial _u J-2 r^2 \partial _r\partial _u U-4 \eth \partial _u\beta \bigg
                        )=0. \end{aligned}$$

**Fig. 9:**
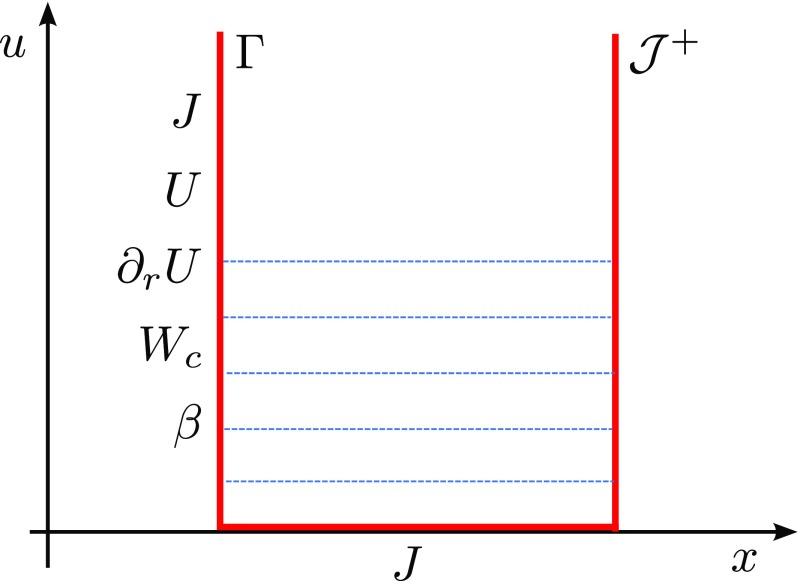
Schematic illustration of the boundary data required for the characteristic code. The data required is *J* at $$u=0$$ and $$\beta ,J, U, \partial _r U,
                                 W_c$$ on the worldtube $$\varGamma $$

An evolution problem is normally formulated in the region of spacetime between a timelike or null worldtube $$\varGamma
                        $$ and future null infinity ($${\mathcal
                        {J}}^+$$), with (free) initial data *J* given on $$u=0$$, and with boundary data for $$\beta ,U,\partial _r
                        U,W_c,J$$ satisfying the constraints given on $$\varGamma
                        $$ (Fig. 9). (In characteristic extraction, the data satisfies the Einstein equations inside $$\varGamma
                        $$, and so the issue of ensuring that the boundary data must satisfy the characteristic constraint equations does not arise). The hypersurface equations are solved to find $$\beta
                        ,U,W_c$$, and then the evolution equation gives $$\partial _u
                        J$$ and thence *J* on the “next” null cone. See Kreiss and Winicour (2011) and Babiuc et al. (2014) for a discussion of the well-posedness of the problem.

We extend the computational grid to $$\mathcal
                        {J}^+$$ by compactifying the radial coordinate *r* by means of a transformation $$r \rightarrow
                        x=f(r)$$ where $$\lim _{r \rightarrow
                        \infty } f(r)$$ is finite. In characteristic coordinates, the Einstein equations remain regular at $$\mathcal
                        {J}^+$$ under such a transformation. In practice, in numerical work the compactification is usually

259 $$\begin{aligned} r
                        \rightarrow x=\frac{r}{r+r_\varGamma }.
                        \end{aligned}$$

However, for the purpose of extracting gravitational waves, it is more convenient to express quantities as power series about $$\mathcal
                        {J}^+$$, and so we compactify using

260 $$\begin{aligned}
                        r\rightarrow \rho =1/r. \end{aligned}$$

(Common practice has been to use the notation $$\ell
                        $$ for 1 / *r*, but since we will have expressions involving the compactified radial coordinate and spherical harmonics such a notation would be confusing). Starting from the Bondi–Sachs metric Eq. (250), we make the coordinate transformation (260) to obtain

261 $$\begin{aligned} ds^2=
                        & {} \rho ^{-2}\left( -\left( e^{2\beta }(\rho ^2+\rho W_c)
                        -h_{_{AB}}U^{^{_A}}U^{^{_{_{B}}}}\right) \, du^2 +2e^{2\beta }\, du \, d\rho
                        -2 h_{_{AB}}U^{^{_{_{B}}}} \, du \, d\phi ^{^{_A}}\right. \nonumber
                        \\&\left. + h_{_{AB}} \, d\phi ^{^{_A}} \, d\phi
                        ^{^{_{_{B}}}}\right) . \end{aligned}$$

In contravariant form,

262 $$\begin{aligned}
                        {g}^{11}=e^{-2\beta }\rho ^3(\rho + W_c), \quad {g}^{1A}=\rho ^2e^{-2\beta }
                        U^{^{_A}}, {g}^{10}=\rho ^2e^{-2\beta }, \quad {g}^{^{_{AB}}}=\rho
                        ^2h^{^{_{AB}}}, \quad {g}^{0A}={g}^{00}=0.
                        \end{aligned}$$

Later, we will need to use the asymptotic Einstein equations, that is the Einstein equations keeping only the leading order terms when the limit $$r\rightarrow \infty
                        $$, or equivalently $$\rho \rightarrow
                        0$$, is taken. We write the metric variables as $$J=J_{(0)}+J_{(1)}\rho
                        $$, and similarly for $$\beta ,
                        U$$ and $$W_c$$. Each Einstein equation is expressed as a series in $$\rho
                        $$ and only leading order terms are considered. There is considerable redundancy, and instead of 10 independent relations we find (see section “Computer algebra” in “Appendix 3”)

263 $$\begin{aligned}&\displaystyle R_{11}=0 \rightarrow
                        \beta _{(1)}=0, \end{aligned}$$

264 $$\begin{aligned}&\displaystyle q^{^{_A}}
                        R_{1{\scriptscriptstyle A}}=0 \rightarrow -2\eth \beta _{(0)} +e^{-2\beta
                        _{(0)}} K_{(0)} U_{(1)}+e^{-2\beta _{(0)}} J_{(0)} \bar{U}_{(1)}=0,
                        \end{aligned}$$

265 $$\begin{aligned}&\displaystyle
                        h^{^{_{AB}}}R_{_{AB}}=0\rightarrow 2W_{c(0)} - \eth \bar{U}_{(0)} -
                        \bar{\eth }U_{(0)}=0, \end{aligned}$$

266 $$\begin{aligned}&\displaystyle q^{^{_A}}
                        q^{^{_{_{B}}}} R_{_{AB}}=0 \rightarrow 2K_{(0)}\eth U_{(0)} + 2\partial _u
                        J_{(0)} +\bar{U}_{(0)}\eth J_{(0)}+U_{(0)}\bar{\eth } J_{(0)}\nonumber
                        \\&\displaystyle +J_{(0)}\eth \bar{U}_{(0)}-J_{(0)}\bar{\eth }
                        U_{(0)}=0. \end{aligned}$$

The above are for the fully nonlinear case, with the linearized approximation obtained by setting $$K_{(0)}=e^{-2\beta
                        _{(0)}}=1$$, and ignoring products of *J* and *U* terms.

### The Bondi gauge

In the Bondi gauge, the form of the Bondi–Sachs metric is manifestly asymptotically flat since it tends to Minkowskian form as $$r\rightarrow \infty
                        $$. In order to see what conditions are thus imposed, the first step is to write the Minkowskian metric in compactified Bondi–Sachs coordinates. Starting from the Minkowski metric in spherical coordinates $$(t,r,\phi
                        ^{^{_A}})$$, we make the coordinate transformation $$(t,r)\rightarrow (u,\rho
                        )$$ where

267 $$\begin{aligned}
                        u=t-r,\;\;\rho =\frac{1}{r} \end{aligned}$$

to obtain

268 $$\begin{aligned}
                        ds^2=\rho ^{-2}\left( -\rho ^2 du^2+2 du\, d\rho +q_{_{AB}}d\phi
                        ^{^{_A}}\,d\phi ^{^{_{_{B}}}} \right) .
                        \end{aligned}$$

We use the notation $$\;\tilde{
                        }\;$$ to denote quantities in the Bondi gauge. The metric of Eqs. (261) and (262) still applies, with the additional properties as $${\tilde{\rho
                        }}\rightarrow 0$$,

269 $$\begin{aligned}
                        \tilde{J}&=0,\quad \tilde{K}=1,\quad \tilde{\beta }=0,\quad
                        \tilde{U}=0,\quad \tilde{W}_c=0, \nonumber \\ \partial _{\tilde{\rho
                        }}\tilde{K}&=0,\;\partial _{\tilde{\rho }}\tilde{\beta }=0,\;
                        \partial _{\tilde{\rho }}\tilde{U}=0,\;\partial _{\tilde{\rho
                        }}\tilde{W}_{c}=0. \end{aligned}$$

The undifferentiated conditions can be regarded as defining the Bondi gauge, being motivated by the geometric condition that the metric Eq. (261) should take the form Eq. (268) in the limit as $$\rho \rightarrow
                        0$$. The conditions on $$\partial _{\tilde{\rho
                        }}\tilde{\beta }$$, $$\partial _{\tilde{\rho
                        }}\tilde{U}$$ and $$\partial _{\tilde{\rho
                        }}\tilde{K}$$ follow from the asymptotic Eqs. (263), (264), and (423) respectively; and the condition on $$\partial _{\tilde{\rho
                        }}\tilde{W}_{c}$$ is obtained from the asymptotic Einstein equation $$h^{^{_{AB}}}R_{_{AB}}=0$$ to second order in $$\rho
                        $$ and applying the Bondi gauge conditions already obtained. The null tetrad in the Bondi gauge will be denoted by $$\tilde{\ell }^\alpha
                        ,\tilde{n}_{_{[NP]}}^\alpha , \tilde{m}^\alpha $$, with components to leading order in $$\tilde{\rho
                        }$$ [obtained by applying the coordinate transformation (267) to Eq. (96)]

270 $$\begin{aligned}
                        \tilde{\ell }^\alpha =\left( 0,- \frac{\tilde{\rho }^2}{\sqrt{2}},0,0\right)
                        ,\qquad \tilde{n}_{_{[NP]}}^\alpha =\left( \sqrt{2},\frac{\tilde{\rho
                        }^2}{\sqrt{2}},0,0\right) ,\qquad \tilde{m}^\alpha =\left(
                        0,0,\frac{\tilde{\rho } q^{^{_A}}}{\sqrt{2}}\right) .
                        \end{aligned}$$

The gravitational news was defined by Bondi et al. (1962) and is

271 $$\begin{aligned}
                        {{\mathcal {N}}}=\frac{1}{2} \partial _{\tilde{u}} \partial _{\tilde{\rho
                        }}\tilde{J}, \end{aligned}$$

evaluated in the limit $$\tilde{\rho }\rightarrow
                        0$$, and is related to the strain in the TT gauge by

272 $$\begin{aligned}
                        {{\mathcal {N}}}=\frac{1}{2} \partial _{\tilde{u}}\lim
                        _{\tilde{r}\rightarrow \infty } \tilde{r}\left( h_+ +i h_\times \right) =
                        \frac{1}{2}\partial _{\tilde{u}}H, \end{aligned}$$

where the rescaled strain *H* is

273 $$\begin{aligned} H:=\lim
                        _{\tilde{r}\rightarrow \infty } \tilde{r}\left( h_+ +i h_\times \right)
                        =\partial _{\tilde{\rho }} \tilde{J},
                        \end{aligned}$$

which result is a straightforward consequence of the relation $$\tilde{J}=h_++ih_\times
                        $$ discussed in section “Spin-weighted representation of deviations from spherical symmetry” in “Appendix 2”. When using the Newman–Penrose formalism to describe gravitational waves, it is convenient to introduce

274 $$\begin{aligned} \psi
                        ^0_4= \lim _{\tilde{r}\rightarrow \infty } \tilde{r}\psi _4 \;\;\left( =\lim
                        _{\tilde{\rho }\rightarrow 0} \frac{\psi _4}{\tilde{\rho }} \right) ,
                        \end{aligned}$$

since it will be important, when considering conformal compactification (Sect. 6.5), to have a quantity that is defined at $$\tilde{\rho
                        }=0$$. In the Bondi gauge, as shown in section “Computer algebra” in “Appendix 3”, $$\psi
                        ^0_4$$ simplifies to

275 $$\begin{aligned} \psi
                        ^0_4= \partial ^2_{\tilde{u}} \partial _{\tilde{\rho }}\bar{\tilde{J}},
                        \end{aligned}$$

evaluated in the limit $$\tilde{\rho }\rightarrow
                        0$$. Thus $$\psi
                        ^0_4$$, $${{\mathcal
                        {N}}}$$ and *H* are related by

276 $$\begin{aligned} \psi
                        ^0_4=2 \partial _{\tilde{u}} {\bar{\mathcal {N}}}= \partial ^2_{\tilde{u}}
                        \bar{H}. \end{aligned}$$

### General gauge

**Fig. 10:**
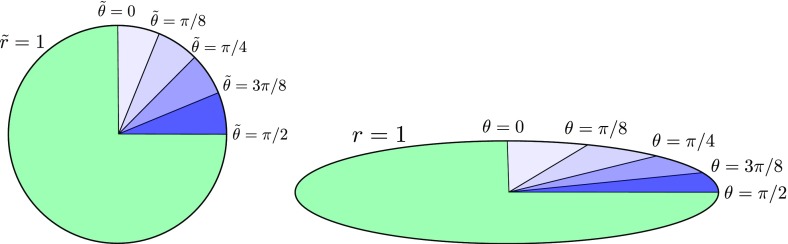
Illustration of the relation between the Bondi and general gauges in Minkowski spacetime. In the Bondi gauge the unit sphere $$\tilde{r}=1$$ has constant curvature (*left panel*). Now consider a coordinate transformation $$\tilde{\theta }\rightarrow \theta
                                 $$ with $$d\theta /d\tilde{\theta
                                 }>1$$ near where $$\tilde{\theta }$$ is 0 and $$\pi $$, and with $$d\theta /d\tilde{\theta
                                 }<1$$ near where $$\tilde{\theta }$$ is $$\pi /2$$. In these coordinates, the surface of constant surface area $$r=1$$ will be as shown in the *right panel*, and will not be a spherical surface of constant curvature

We construct quantities in the general gauge by means of a coordinate transformation to the Bondi gauge, although this transformation is largely implicit because it does not appear in many of the final formulas. The transformation is written as a series expansion in $$\rho
                        $$ with coefficients arbitrary functions of the other coordinates. Thus it is a general transformation, and the requirements that $$g^{\alpha \beta
                        }$$ must be of Bondi–Sachs form, and that $$\tilde{g}^{\alpha \beta
                        }$$ must be in the Bondi gauge, impose conditions on the transformation coefficients. The transformation is (Fig. 10)

277 $$\begin{aligned} u
                        \rightarrow {\tilde{u}}=u+u_0+\rho A^u, \qquad \rho \rightarrow {\tilde{\rho
                        }}=\rho \omega +\rho ^2 A^\rho , \qquad \phi ^{^{_A}} \rightarrow
                        {\tilde{\phi }}^{^{_A}}=\phi ^{^{_A}}+\phi ^{^{_A}}_0+\rho A^{^{_A}},
                        \end{aligned}$$

where the transformation coefficients $$u_0,A^u,\omega ,A^\rho
                        ,\phi ^{^{_A}}_0,A^{^{_A}}$$ are all functions of *u* and $$\phi
                        ^{^{_A}}$$ only. Conditions on the coefficients are found by applying the tensor transformation law

278 $$\begin{aligned}
                        {{{\tilde{g}}}}^{\alpha \beta }=\frac{\partial {\tilde{x}}^\alpha }{\partial
                        x^\mu } \frac{\partial {\tilde{x}}^\beta }{\partial x^\nu } { g}^{\mu \nu },
                        \qquad \hbox {and}\qquad { g}_{\alpha \beta }=\frac{\partial {\tilde{x}}^\mu
                        }{\partial x^\alpha } \frac{\partial {\tilde{x}}^\nu }{\partial x^\beta
                        }{{\tilde{g}}}_{\mu \nu }, \end{aligned}$$

for specific cases of $$\alpha ,\beta
                        $$, using the form of the metric in Eqs. (261) and (262) and also applying the conditions in Eq. (269) to $${{\tilde{g}}}^{\alpha
                        \beta }$$ and $${{\tilde{g}}}_{\mu \nu
                        }$$ (Bishop et al. 1997b; Bishop and Deshingkar 2003; Bishop and Reisswig 2014). The procedure is shown in some detail for one case, with the other cases being handled in a similar way. The actual calculations are performed by computer algebra as discussed in section “Computer algebra” in “Appendix 3”.

From Eqs. (262) and (269), $${{\tilde{g}}}^{01}=\tilde{\rho }^2+ \mathcal
                        {O}(\tilde{\rho }^4)$$. Then using the contravariant transformation in Eq. (278) with $$\alpha =0,\beta
                        =1$$, we have

279 $$\begin{aligned}
                        \tilde{\rho }^2+\mathcal {O}(\tilde{\rho }^4)=\rho ^2\omega ^2+\mathcal
                        {O}({\rho }^4)= \frac{\partial {\tilde{u}}}{\partial x^\mu } \frac{\partial
                        {\tilde{\rho }}}{\partial x^\nu } { g}^{\mu \nu }.
                        \end{aligned}$$

Evaluating the right hand side to $$\mathcal {O}({\rho
                        }^2)$$, the resulting equation simplifies to give

280 $$\begin{aligned}
                        (\partial _u+U^{^{_{_{B}}}}\partial _{_{B}})u_{0}=\omega e^{2\beta }-1.
                        \end{aligned}$$

The remaining conditions follow in a similar way

281 $$\begin{aligned}
                        0+\mathcal {O} (\tilde{\rho }^4)= & {} {{{\tilde{g}}}}^{A1}, \qquad
                        \hbox {so that to }\mathcal {O} ({\rho }^2),\qquad (\partial
                        _u+U^{^{_{_{B}}}}\partial _{_{B}})\phi ^{^{_A}}_0=-U^{^{_A}},
                        \end{aligned}$$

282 $$\begin{aligned}
                        0+\mathcal {O} (\tilde{\rho }^4)= & {} {{{\tilde{g}}}}^{11}, \qquad
                        \hbox {so that to }\mathcal {O} ({\rho }^3),\qquad (\partial
                        _u+U^{^{_{_{B}}}}\partial _{_{B}})\omega =-\omega W_c/2,
                        \end{aligned}$$

283 $$\begin{aligned} 0=
                        & {} {{\tilde{g}}}^{00},\qquad \hbox {so that to }\mathcal {O}({\rho
                        }^2),\quad \; 2\omega A^u=\frac{J\bar{\eth }^2u_0+\bar{J}\eth ^2
                        u_0}{2}-K\eth u_0 \bar{\eth }u_0.\nonumber \\
                        \end{aligned}$$

In the next equations, $$X_0=q_{_{A}} \phi
                        _0^{^{_A}}, A=q_{_{A}} A^{^{_A}}$$; the introduction of these quantities is a convenience to reduce the number of terms in the formulas, since $$\phi _0^{^{_A}},
                        A^{^{_A}}$$ do not transform as 2-vectors. As a result, the quantity $$\zeta
                        =q+ip$$ also appears, and the formulas are specific to stereographic coordinates. We find

284 $$\begin{aligned}
                        0=\tilde{q}_{_{A}} {{\tilde{g}}}^{0A},
                        \end{aligned}$$

so that to $$\mathcal {O}({\rho
                        }^2)$$

285 $$\begin{aligned}
                        0=&\, 2A\omega +2A_u X_0 U \bar{\zeta } e^{-2\beta } +K\eth u_0 (2
                        +\bar{\eth }X_0+2X_0\bar{\zeta })\nonumber \\&+K\bar{\eth }u_0\eth
                        X_0 \bar{\eth }u_0 (2+\bar{\eth }X_0+2 X_0\bar{\zeta })-\bar{J}\eth u_0 \eth
                        X_0, \end{aligned}$$

286 $$\begin{aligned} \det
                        (q_{_{AB}})=&\det (g_{_{AB}})\rho ^4,
                        \end{aligned}$$

so that at $$\rho
                        =0$$

287 $$\begin{aligned} \omega
                        =\frac{1+q^2+p^2}{1+\tilde{q}^2+\tilde{p}^2} \sqrt{1+\partial _q
                        q_{0}+\partial _p p_{0}+\partial _q q_{0}\partial _p p_{0} -\partial _p
                        q_{0}\partial _q p_{0}}, \end{aligned}$$

and

288 $$\begin{aligned}
                        J=\frac{q^{^{_A}} q^{^{_{_{B}}}} g_{_{AB}}}{2} \rho ^2,
                        \end{aligned}$$

so that at $$\rho
                        =0$$

289 $$\begin{aligned} \qquad
                        J= \frac{(1+q^2+p^2)^2}{2(1+\tilde{q}^2+\tilde{p}^2)^2\omega ^2} \eth X_0
                        (2+\eth \bar{X}_0+2\bar{X} \zeta ). \end{aligned}$$

Explicit expressions for the null tetrad vectors $$n_{_{[NP]}}^\alpha
                        $$ and $$m^\alpha
                        $$ (but not $$\ell ^\alpha
                        $$) will be needed. $$n_{_{[NP]}}^\alpha
                        $$ is found by applying the coordinate transformation Eq. (278) to $$\tilde{n}_{_{[NP]}\alpha
                        }$$ (Eq. (270)) and then raising the index, giving to leading order in $$\rho
                        $$

290 $$\begin{aligned}
                        {n}_{_{[NP]}}^\alpha =\left( \frac{e^{-2\beta }\sqrt{2}}{\omega }, \rho
                        \frac{e^{-2\beta }(2\partial _u\omega +\bar{U}\eth \omega +U\bar{\eth
                        }\omega +2W_c\omega )}{\sqrt{2}\omega ^2}, \frac{U^{^{_A}}e^{-2\beta
                        }\sqrt{2}}{\omega }\right) . \end{aligned}$$

The calculation of an expression for $$m^\alpha
                        $$ is indirect. Let $$F^{^{_A}}$$ be a dyad of the angular part of the general gauge metric, so it must satisfy Eq. (384), then Bishop et al. (1997b); Babiuc et al. (2009),

291 $$\begin{aligned}
                        F^{^{_A}}=\left( \frac{q^{^{_A}}\sqrt{K+1}}{2}-\frac{\bar{q}^{^{_A}}
                        J}{2\sqrt{(K+1)}}\right) , \end{aligned}$$

with $$F^{^{_A}}$$ undetermined up to an arbitrary phase factor $$e^{-i\delta
                        (u,x^{^{_A}})}$$. We then define $$m_{_{[G]}}^\alpha
                        $$

292 $$\begin{aligned}
                        m_{_{[G]}}^\alpha = e^{-i\delta }\rho (0,0,F^{^{_A}}).
                        \end{aligned}$$

The suffix $${}_{_{[G]}}$$ is used to distinguish the above form from that defined in Eq. (270) since $$m_{_{[G]}}^\alpha \ne
                        m^\alpha $$. However, it will be shown later (see Sects. 6.5.1, 6.5.2 and section “Computer algebra” in “Appendix 3”) that the value of gravitational-wave descriptors is unaffected by the use of $$m_{_{[G]}}^\alpha
                        $$ rather than $$m^\alpha
                        $$ in its evaluation; thus it is permissible, for our purposes, to approximate $$m^\alpha
                        $$ by $$m_{_{[G]}}^\alpha
                        $$. We now transform $$m_{_{[G]}}^\alpha
                        $$ in Eq. (292) to the Bondi gauge,

293 $$\begin{aligned}
                        \tilde{m}_{_{[G]}}^\alpha =\frac{\partial \tilde{x}^\alpha }{\partial
                        x^\beta } m_{_{[G]}}^\beta =\left( \partial _{_{B}}u_0 e^{-i\delta
                        }\frac{\tilde{\rho }}{\omega } F^{^{_{_{B}}}}, \partial _{_{B}}\omega
                        e^{-i\delta }\frac{\tilde{\rho ^2}}{\omega ^2} F^{^{_{_{B}}}}, \partial
                        _{_{B}} \phi ^{^{_A}}_0 e^{-i\delta }\frac{\tilde{\rho }}{\omega }
                        F^{^{_{_{B}}}}\right) . \end{aligned}$$

The component $$\tilde{m}^1_{_{[G]}}$$ is of the same order as $$\tilde{\ell
                        }^1$$, and so $$\tilde{m}_{_{[G]}}^\alpha $$ and $$\tilde{m}^\alpha
                        $$ are not equivalent. It can be checked (see section “Computer algebra” in “Appendix 3”) (Bishop and Reisswig 2014) that the angular part of $$\tilde{m}_{_{[G]}}^\alpha $$ is equivalent to $$\tilde{m}^\alpha
                        $$, since $$\tilde{m}_{_{[G]}}^\alpha \tilde{m}_\alpha
                        =0$$ and

294 $$\begin{aligned}
                        \tilde{m}_{_{[G]}}^\alpha \bar{\tilde{m}}_\alpha =\nu ,
                        \end{aligned}$$

where $$|\nu
                        |=1$$.

Since we actually require $$\nu
                        =1$$, Eq. (294) can be used to set the phase factor $$\delta
                        $$ explicitly. The result is

295 $$\begin{aligned}
                        e^{i\delta }\!=\!\frac{F^{^{_{_{B}}}} \bar{\tilde{q}}_{_{A}}}{\omega
                        \sqrt{2}} \partial _{_{B}} \phi ^{^{_A}}_0 \!=\!\frac{1+q^2+p^2}{4\omega
                        (1\!+\!\tilde{q}^2+\tilde{p}^2)}\sqrt{\frac{2}{K+1}} \left( (K+1)(2+\eth
                        \bar{X}_0+2\bar{X}_0\zeta )-J\bar{\eth }\bar{X}_0\right) .\nonumber \\
                        \end{aligned}$$

An alternative approach (Bishop et al. 1997b; Babiuc et al. 2009), to the phase factor $$\delta
                        $$ uses the condition that $$m_{_{[G]}}^\alpha
                        $$ is parallel propagated along $$\mathcal
                        {J}^+$$ in the direction $${n}_{_{[NP]}}^\alpha
                        $$, yielding the evolution equation

296 $$\begin{aligned}
                        2i(\partial _u +U^{_A}\partial _{_A})\delta = \nabla _{_A} U^{_A} +h_{_{AC}}
                        \bar{F}^{_C} ( (\partial _u +U^{_B} \partial _{_B}) F^{_A} - F^{_B} \partial
                        _{_B} U^{_A}) , \end{aligned}$$

where $$\nabla
                        _{_A}$$ is the covariant derivative with respect to the angular part of the metric $$h_{_{AB}}$$.

### The gravitational-wave strain

An expression for the contravariant metric $$\tilde{g}^{\alpha \beta
                        }$$ in the Bondi gauge is obtained from Eqs. (262) and (269), and each metric variable is expressed as a Taylor series about $$\tilde{\rho
                        }=0$$ (e.g., $$\tilde{J}=0+\tilde{\rho
                        } \partial _{\tilde{\rho }}\tilde{J}+{{\mathcal {O}}}(\tilde{\rho
                        }^2)$$). Applying the coordinate transformation (277) we find $$g^{\alpha \beta
                        }$$, then use $$\tilde{\rho }=\omega
                        \rho +A^\rho \rho ^2$$ to express each component as a series in $$\rho
                        $$; note that the coefficients are constructed from terms in the Bondi gauge, e.g., $$\partial _{\tilde{\rho
                        }}\tilde{J}$$. Then both sides of

297 $$\begin{aligned}
                        J=\frac{q_{_{A}} q_{_{B}} g^{^{_{AB}}}}{2\rho ^2},
                        \end{aligned}$$

with $$g^{^{_{AB}}}$$ given in Eq. (262), are expressed as series in $$\rho
                        $$, and the coefficients of $$\rho
                        ^1$$ are equated. This leads to an equation in which $$\partial _\rho
                        J$$ depends linearly on $$\partial _{\tilde{\rho
                        }} \tilde{J}$$ ($$=H=\lim
                        _{\tilde{r}\rightarrow \infty } \tilde{r}\left( h_+ +i h_\times \right)
                        $$, the rescaled strain defined in Eq. (273) (Bishop and Reisswig 2014),

298 $$\begin{aligned} C_1
                        \partial _\rho J= C_2 \partial _{\tilde{\rho }} \tilde{J} +C_3 \partial
                        _{\tilde{\rho }}\tilde{\bar{J}}+C_4,
                        \end{aligned}$$

which may be inverted to give

299 $$\begin{aligned}
                        H=\partial _{\tilde{\rho }} \tilde{J}=\frac{C_1\bar{C}_2\partial _\rho J
                        -C_3 \bar{C}_1 \partial _{\rho }\bar{J} +C_3 \bar{C}_4 -\bar{C}_2
                        C_4}{\bar{C}_2 C_2-\bar{C}_3 C_3}, \end{aligned}$$

where the coefficients are

300 $$\begin{aligned}
                        C_1&=\frac{4\omega ^2
                        (1+\tilde{q}^2+\tilde{p}^2)^2}{(1+q^2+p^2)^2},\qquad C_2=\omega (2+\eth
                        \bar{X}_0+2\bar{X}_0\zeta )^2, \end{aligned}$$

301 $$\begin{aligned}
                        C_3&=\omega (\eth X_0)^2,\qquad C_4=\eth A (4+2\eth \bar{X}_0+4
                        \bar{X}_0\zeta )+\eth X_0 (2\eth \bar{A}+4\bar{A}\zeta ) +4\eth \omega \eth
                        u_0. \end{aligned}$$

These results are obtained using computer algebra as discussed in section “Computer algebra” in “Appendix 3”. The above formula for the wave strain involves intermediate variables, and the procedure for calculating them is to solve equations for the variable indicated in the following order: Eq. (281) for $$x_0^{^{_A}}$$ and thus $$X_0$$, Eq. (287) for $$\omega
                        $$, Eq. (280) for $$u_0$$, Eq. (283) for $$A^u$$, and Eq. (285) for *A*.

### Conformal compactification

Here we give only a brief introduction to this topic, as these matters are discussed more fully in many standard texts and reviews, e.g., Wald (1984) and Frauendiener (2004). We have made a coordinate compactification, resulting in the metric and null tetrad being singular at $$\rho
                        =0$$, which is therefore not included in the manifold. Thus, quantities are not evaluated at $$\rho
                        =0$$, but in the limit as $$\rho \rightarrow
                        0$$. Introducing a conformal transformation has the advantage that this technical issue is avoided and $$\mathcal
                        {J}^+$$ at $$\rho
                        =0$$ is included in the manifold; but also that the resulting formulas for $${{\mathcal
                        {N}}}$$ and $$\psi
                        ^0_4$$ are simpler. (Of course, it should be possible to use the asymptotic Einstein equations to simplify expressions derived in physical space, but due to the complexity of the formulas this approach has not been adopted).

We use the notation $$\hat{
                        }$$ for quantities in conformal space. In the general gauge, the conformal metric $$\hat{g}_{\alpha \beta
                        }$$ is related to the metric Eq. (261) by $$g_{\alpha \beta }=\rho
                        ^{-2}\hat{g}_{\alpha \beta }$$ so that

302 $$\begin{aligned}
                        d\hat{s}^2= & {} -\left( e^{2\beta }(\rho ^2+\rho W_c)
                        -h_{_{AB}}U^{^{_A}}U^{^{_{_{B}}}}\right) \, du^2 +2e^{2\beta } \, du \,
                        d\rho -2 h_{_{AB}}U^{^{_{_{B}}}} \, du \, d\phi ^{^{_A}} \nonumber
                        \\&+ h_{_{AB}} \, d\phi ^{^{_A}} \, d\phi ^{^{_{_{B}}}}.
                        \end{aligned}$$

In a similar way, the Bondi gauge conformal metric $$\hat{\tilde{g}}_{\alpha
                        \beta }$$ is related to the Bondi gauge metric $$\tilde{g}_{\alpha \beta
                        }$$ by $$\tilde{g}_{\alpha \beta
                        }=\tilde{\rho }^{-2}\hat{\tilde{g}}_{\alpha \beta
                        }$$. Thus $$\hat{g}_{\alpha \beta
                        }$$ and $$\hat{\tilde{g}}_{\alpha
                        \beta }$$ are regular at $$\rho
                        =0$$ (or equivalently at $$\tilde{\rho
                        }=0$$) and so in the conformal picture $$\rho
                        =0$$ is included in the manifold. The conformal metrics $$\hat{g}_{\alpha \beta
                        }$$ and $$\hat{\tilde{g}}_{\alpha
                        \beta }$$ are related by

303 $$\begin{aligned}
                        \hat{g}_{\alpha \beta }=\rho ^2 g_{\alpha \beta }=\rho ^2 \frac{\partial
                        {\tilde{x}}^\gamma }{\partial x^\alpha } \frac{\partial {\tilde{x}}^\delta
                        }{\partial x^\beta }{{\tilde{g}}}_{\gamma \delta } =\frac{\rho
                        ^2}{\tilde{\rho }^2}\frac{\partial {\tilde{x}}^\gamma }{\partial x^\alpha }
                        \frac{\partial {\tilde{x}}^\delta }{\partial x^\beta
                        }{\hat{{\tilde{g}}}}_{\gamma \delta } =\frac{1}{\omega ^2}\frac{\partial
                        {\tilde{x}}^\gamma }{\partial x^\alpha } \frac{\partial {\tilde{x}}^\delta
                        }{\partial x^\beta }{\hat{{\tilde{g}}}}_{\gamma \delta } +{{\mathcal
                        {O}}}(\rho ), \end{aligned}$$

which at $$\rho
                        =0$$ is the usual tensor transformation law with an additional factor $$\omega
                        ^{-2}$$. A quantity that obeys this property is said to be *conformally invariant* with weight *n* where *n* is the power of $$\omega
                        $$ in the additional factor; thus the metric tensor is conformally invariant with weight $$-2$$. In practice, it is not necessary to establish a relation of the form Eq. (303) to prove conformal invariance. The key step is to be able to show that a tensor quantity $$T^{a\cdots }_{b\cdots
                        }$$ satisfies $$\hat{T}^{a\cdots
                        }_{b\cdots } =\rho ^{-n}T^{a\cdots }_{b\cdots }$$, then conformal invariance with weight *n* easily follows. In Eq. (303) the error term $${{\mathcal {O}}}(\rho
                        )$$ is shown explicitly, although it turns out to be irrelevant since the relation is evaluated at $$\rho
                        =0$$. This is generally the case, so from now on the error terms will not be taken into account; the one exception will be in the News calculation Sect. 6.5.1 which in places involves off-$$\mathcal
                        {J}^+$$ derivatives (since $$\partial _\rho
                        {{\mathcal {O}}}(\rho ) ={{\mathcal {O}}}(1)$$).

It is important to note that not all tensor quantities are conformally invariant, and in particular this applies to the metric connection and thus covariant derivatives

304 $$\begin{aligned}
                        \hat{\varGamma }^\gamma _{\alpha \beta }=\varGamma ^\gamma _{\alpha \beta }
                        +\frac{\delta ^\gamma _\alpha \partial _\beta \rho +\delta ^\gamma _\beta
                        \partial _\alpha \rho -\hat{g}_{\alpha \beta }\hat{g}^{\gamma \delta
                        }\partial _\delta \rho }{\rho }, \end{aligned}$$

and to the Ricci scalar in *n*-dimensions

305 $$\begin{aligned}
                        \hat{R}=\rho ^{-2}\left[ R-2(n-1)g^{ab}\nabla _a\nabla _b\ln \rho
                        -(n-1)(n-2)g^{ab}(\nabla _a\ln \rho )(\nabla _b\ln \rho )\right] .
                        \end{aligned}$$

The Weyl tensor, however, is conformally invariant,

306 $$\begin{aligned}
                        \hat{C}^\alpha _{\beta \gamma \delta }=C^\alpha _{\beta \gamma \delta },
                        \qquad \hbox {and}\qquad \hat{C}_{\alpha \beta \gamma \delta }=\rho ^2
                        C_{\alpha \beta \gamma \delta }, \end{aligned}$$

so that the forms $$C^\alpha _{\beta \gamma
                        \delta }$$ and $$C_{\alpha \beta \gamma
                        \delta }$$ are conformally invariant with weights 0 and $$-2$$ respectively. The construction of the conformal null tetrad vectors is not unique. It is necessary that orthonormality conditions analogous to Eq. (97) be satisfied, and it is desirable that the component of leading order in $$\rho
                        $$ should be finite but nonzero at $$\rho
                        =0$$. These conditions are achieved by defining

307 $$\begin{aligned}
                        \hat{n}_{_{[NP]}}^\alpha =n_{_{[NP]}}^\alpha , \quad \hat{m}_{_{[G]}}^\alpha
                        =\frac{m_{_{[G]}}^\alpha }{\rho }. \end{aligned}$$

Thus $$\hat{n}_{_{[NP]}}^\alpha
                        $$ and $$\hat{\tilde{n}}_{_{[NP]}}^a$$ are related by the usual tensor transformation law, and

308 $$\begin{aligned}
                        \hat{\tilde{m}}_{_{[G]}}^\alpha =\frac{\tilde{m}_{_{[G]}}^\alpha
                        }{\tilde{\rho }}= \frac{1}{\omega \rho }\frac{\partial \tilde{x}^\alpha
                        }{\partial x^\beta } m_{_{[G]}}^\beta =\frac{1}{\omega }\frac{\partial
                        \tilde{x}^\alpha }{\partial x^\beta } \hat{m}_{_{[G]}}^\beta .
                        \end{aligned}$$

With these definitions, $$n_{_{[NP]}}^\alpha
                        ,m_{_{[G]}}^\alpha $$ are conformally invariant with weights 0 and 1 respectively.

Considering the conformally compactified metric of the spherical 2-surface described by the angular coordinates ($$\tilde{\phi
                        }^{^{_A}}$$ or $$\phi
                        ^{^{_A}}$$) at $$\mathcal
                        {J}^+$$, we have

309 $$\begin{aligned}
                        ds^2=\tilde{\rho }^{-2} d\hat{\tilde{s}}^2= \tilde{\rho
                        }^{-2}q_{_{AB}}d\tilde{\phi }^{^{_A}}d\tilde{\phi }^{^{_{_{B}}}} =\rho
                        ^{-2}d\hat{s}^2=\rho ^{-2}h_{_{AB}}d\phi ^{^{_A}} d\phi ^{^{_{_{B}}}},
                        \end{aligned}$$

so that

310 $$\begin{aligned}
                        q_{_{AB}}d\tilde{\phi }^{^{_A}}d\tilde{\phi }^{^{_{_{B}}}} =\omega
                        ^2h_{_{AB}}d\phi ^{^{_A}}d\phi ^{^{_{_{B}}}},
                        \end{aligned}$$

since $$\omega =\tilde{\rho
                        }/\rho $$. The curvature of $$\mathcal
                        {J}^+$$ is evaluated in two different ways, and the results are equated. The metric on the LHS is that of a unit sphere, and therefore has Ricci scalar $${\mathcal
                        {R}}(\tilde{x}^{^{_A}})=2$$; and the metric on the RHS is evaluated using Eq. (305)with $$n=2$$. Thus,

311 $$\begin{aligned}
                        2=\frac{1}{\omega ^2}\left( {\mathcal {R}}(\phi
                        ^{^{_A}})-2h^{^{_{AB}}}\nabla _{_{A}}\nabla _{_{B}} \log (\omega )\right) ,
                        \end{aligned}$$

leading to

312 $$\begin{aligned} 2\omega
                        ^2+2h^{^{_{AB}}}\nabla _{_{A}}\nabla _{_{B}}\log (\omega )= & {}
                        2K-\bar{\eth }\eth K+\frac{1}{2} \left( \eth ^2\bar{J}+\bar{\eth }^2
                        J\right) \nonumber \\&+\frac{1}{4K}\left( (\bar{\eth }\bar{J})(\eth
                        J)-(\bar{\eth }{J})(\eth \bar{J})\right) ,
                        \end{aligned}$$

where the relationship between $${\mathcal {R}}(\phi
                        ^{^{_A}})$$ and *J*, *K* is derived in Gómez et al. (1997), and where $$h^{^{_{AB}}}\nabla
                        _{_{A}}\nabla _{_{B}}\log (\omega )$$ is given in terms of the $$\eth
                        $$ operator in Eq. (B1) of Bishop et al. (1997b). Eq. (312) is a nonlinear elliptic equation, and in practice is not actually solved. However, it will be used later, when considering the linearized approximation, since in that case it has a simple analytic solution.

#### The news $${{\mathcal
                           {N}}}$$

A difficulty with evaluating the gravitational radiation by means of Eq. (271) is that it is valid only in a specific coordinate system, so a more useful approach is to use the definition (Penrose 1963; Bishop et al. 1997b; Babiuc et al. 2009)

313 $$\begin{aligned}
                           {{\mathcal {N}}}= \lim _{\tilde{\rho }\rightarrow
                           0}\frac{\hat{\tilde{m}}^\alpha \hat{\tilde{m}}^\beta \hat{\tilde{\nabla
                           }}_\alpha \hat{\tilde{\nabla }}_\beta \tilde{\rho }}{\tilde{\rho }}.
                           \end{aligned}$$

At first sight Eqs. (271) and (313) do not appear to be equivalent, but the relationship follows by expanding out the covariant derivatives in Eq. (313)

314 $$\begin{aligned}
                           {{\mathcal {N}}}= \lim _{\tilde{\rho }\rightarrow
                           0}\frac{\hat{\tilde{m}}^\alpha \hat{\tilde{m}}^\beta (\partial _\alpha
                           \partial _\beta \tilde{\rho }-\hat{\tilde{\varGamma }}^\gamma _{\alpha
                           \beta } \partial _\gamma \tilde{\rho })}{\tilde{\rho }} =-\lim
                           _{\tilde{\rho }\rightarrow 0}\frac{q^{^{_A}} q^{^{_{_{B}}}}
                           \hat{\tilde{\varGamma }}^1_{_{AB}} }{2\tilde{\rho }},
                           \end{aligned}$$

then expressing the metric coefficients $$\tilde{J}$$ etc. as power series in $$\tilde{\rho
                           }$$ as introduced just before Eq. (263). Using the Bondi gauge conditions Eq. (269), it quickly follows that $$-q^{^{_A}}
                           q^{^{_{_{B}}}} \hat{\tilde{\varGamma }}^1_{(0)AB}/2 = \partial
                           _{\tilde{u}} \tilde{J}/2$$, which is zero, and the result follows since $$-q^{^{_A}}
                           q^{^{_{_{B}}}} \hat{\tilde{\varGamma }}^1_{(1)AB}/2 = \partial
                           _{\tilde{u}}\partial _{\tilde{\rho
                           }}\tilde{J}/2$$. Computer algebra has been used to check that replacing $$\hat{\tilde{m}}^\alpha $$ in Eq. (313) by $$\hat{\tilde{m}}_{_{[G]}}^\alpha
                           $$ (with $$\tilde{m}^\alpha
                           _{_{[G]}}$$ defined in Eq. (293)) has no effect.^13^

Because covariant derivatives are not conformally invariant, transforming Eq. (313) into the general gauge is a little tricky. We need to transform to physical space, where tensor quantities with no free indices are invariant across coordinate systems, and then to conformal space in the general gauge. From Eq. (304), and using $$\tilde{\rho }= \rho
                           \omega $$ and $$\tilde{\nabla
                           }_\gamma \tilde{\rho }=\delta ^1_\gamma $$,

315 $$\begin{aligned}
                           \hat{\nabla }_\alpha \hat{\nabla }_\beta \tilde{\rho }&= \nabla
                           _\alpha \nabla _\beta \tilde{\rho } +\frac{\hat{g}_{\alpha \beta
                           }\hat{g}^{\gamma 1}-\delta ^\gamma _\alpha \delta ^1_\beta -\delta
                           ^\gamma _\beta \delta ^1_\alpha }{\rho } \nabla _\gamma (\rho \omega ),
                           \end{aligned}$$

316 $$\begin{aligned}
                           \hat{\tilde{\nabla }}_\alpha \hat{\tilde{\nabla }}_\beta \tilde{\rho
                           }&= \tilde{\nabla }_\alpha \tilde{\nabla }_\beta \tilde{\rho }
                           +\frac{\hat{\tilde{g}}_{\alpha b}\hat{\tilde{g}}^{11}-2\delta ^1_\alpha
                           \delta ^1_\beta }{\tilde{\rho }}.
                           \end{aligned}$$

Now consider $$\tilde{m}_{_{[G]}}^\alpha \tilde{m}_{_{[G]}}^\beta
                           \times $$ Eq. (316) $$-\;m_{_{[G]}}^\alpha
                           m_{_{[G]}}^\beta \times $$ Eq. (315). Using the conditions that (a) scalar quantities are invariant in physical space so that $$\tilde{m}_{_{[G]}}^\alpha \tilde{m}_{_{[G]}}^\beta
                           \tilde{\nabla }_\alpha \tilde{\nabla }_\beta \tilde{\rho } -
                           m_{_{[G]}}^\alpha m_{_{[G]}}^\beta \nabla _\alpha \nabla _\beta
                           \tilde{\rho }=0$$, (b) $$\hat{\tilde{g}}^{11}$$ is zero to $${{\mathcal {O}}}
                           (\tilde{\rho }^2)$$, (c) $$ m_{_{[G]}}^\alpha
                           \delta ^1_\alpha =0$$, and (d) from Eq. (293)

317 $$\begin{aligned}
                           \tilde{m}_{_{[G]}}^\alpha \delta ^1_\alpha =e^{-i\delta }\rho ^2
                           F^A\partial _A\omega = \rho m_{_{[G]}}^\alpha \partial _\alpha \omega ,
                           \end{aligned}$$

it follows that

318 $$\begin{aligned}
                           \tilde{m}_{_{[G]}}^\alpha \tilde{m}_{_{[G]}}^\beta \hat{\tilde{\nabla
                           }}_\alpha \hat{\tilde{\nabla }}_\beta \tilde{\rho }
                           \!=\!m_{_{[G]}}^\alpha m_{_{[G]}}^\beta \left( \hat{\nabla }_\alpha
                           \hat{\nabla }_\beta (\rho \omega ) -\hat{g}_{\alpha \beta }\left(
                           \frac{\hat{g}^{11}\omega }{\rho } +\hat{g}^{1\gamma }\partial _\gamma
                           \omega \right) \!-\!\frac{2\rho \partial _\alpha \omega \partial _\beta
                           \omega }{\omega }\right) . \end{aligned}$$

Since $$m_{_{[G]}}^\alpha
                           $$ and $$\hat{\nabla }_\alpha
                           \rho $$ are orthogonal, we may write $$\hat{\nabla }_\alpha
                           \hat{\nabla }_\beta (\rho \omega ) = \omega \hat{\nabla }_\alpha
                           \hat{\nabla }_\beta \rho +\rho \hat{\nabla }_\alpha \hat{\nabla }_\beta
                           \omega $$, so that

319 $$\begin{aligned}
                           \tilde{m}_{_{[G]}}^\alpha \tilde{m}_{_{[G]}}^\beta \hat{\tilde{\nabla
                           }}_\alpha \hat{\tilde{\nabla }}_\beta \tilde{\rho } =m_{_{[G]}}^\alpha
                           m_{_{[G]}}^\beta \left( \omega \hat{\nabla }_\alpha \hat{\nabla }_\beta
                           \rho +\rho \hat{\nabla }_\alpha \hat{\nabla }_\beta \omega
                           -\hat{g}_{\alpha \beta }\left( \frac{\hat{g}^{11}\omega }{\rho }
                           +\hat{g}^{1\gamma }\partial _\gamma \omega \right) -\frac{2\rho \partial
                           _\alpha \omega \partial _\beta \omega }{\omega }\right) .
                           \end{aligned}$$

This expression is simplified by (a) expanding out the covariant derivatives, (b) expressing the metric as a power series in $$\rho
                           $$ and using $$m_{_{[G]}}^\alpha
                           m_{_{[G]}}^\beta \hat{g}_{(0)\alpha \beta }=0$$, and (c) using Eq. (282),

320 $$\begin{aligned}
                           \tilde{m}_{_{[G]}}^\alpha \tilde{m}_{_{[G]}}^\beta \hat{\tilde{\nabla
                           }}_\alpha \hat{\tilde{\nabla }}_\beta \tilde{\rho }\!=\!
                           m_{_{[G]}}^\alpha m_{_{[G]}}^\beta \left( - \omega \hat{\varGamma
                           }^1_{a\beta } +\rho \partial _\alpha \partial _\beta \omega -\rho
                           \partial _\gamma \omega \hat{\varGamma }^\gamma _{\alpha \beta }
                           -\frac{\rho \omega e^{-2\beta }W_c \partial _\rho \hat{g}_{\alpha \beta
                           }}{2} -\frac{2\rho \partial _\alpha \omega \partial _\beta \omega
                           }{\omega }\right) . \end{aligned}$$

Finally, Eq. (292) is used to replace $$m_{_{[G]}}^\alpha
                           $$ in terms of $$F^{^{_A}}$$, and the whole expression is divided by $$\tilde{\rho
                           }^3$$, yielding (Bishop et al. 1997b; Babiuc et al. 2009)

321 $$\begin{aligned}
                           {{\mathcal {N}}}&= \lim _{\tilde{\rho }\rightarrow 0}
                           \frac{\hat{\tilde{m}}^\alpha \hat{\tilde{m}}^\beta \hat{\tilde{\nabla
                           }}_\alpha \hat{\tilde{\nabla }}_\beta \tilde{\rho }}{\tilde{\rho }} =
                           \lim _{\tilde{\rho }\rightarrow 0} \frac{\hat{\tilde{m}}_{_{[G]}}^\alpha
                           \hat{\tilde{m}}_{_{[G]}}^\beta \hat{\tilde{\nabla }}_\alpha
                           \hat{\tilde{\nabla }}_\beta \tilde{\rho }}{\tilde{\rho }}\nonumber
                           \\&= \frac{e^{-2i\delta }}{\omega ^2}\bigg [ -\lim _{\rho
                           \rightarrow 0}\frac{F^{^{_A}}F^{^{_{_{B}}}} \hat{\varGamma
                           }^1_{_{AB}}}{\rho }\nonumber \\&\quad
                           +F^{^{_A}}F^{^{_{_{B}}}}\left( \frac{\partial _{_A}\partial _{_B}\omega
                           }{\omega } -\frac{\hat{\varGamma }^{\gamma }_{_{AB}}\partial _\gamma
                           \omega }{\omega } -\frac{\partial _\rho \hat{g}_{_{AB}}e^{-2\beta
                           }W_c}{2} -\frac{2\partial _{_A}\omega \partial _{_B}\omega }{\omega ^2}
                           \right) \bigg ]. \end{aligned}$$

The limit is evaluated by expressing each metric coefficient as a power series in $$\rho
                           $$, e.g., $$J=J_{(0)}+\rho
                           J_{(1)}$$, and then writing $$F^{^{_A}}F^{^{_{_{B}}}} \hat{\varGamma }^1_{_{AB}}=
                           F^{^{_A}}F^{^{_{_{B}}}} \hat{\varGamma }^1_{_{AB}(0)}+ \rho
                           F^{^{_A}}F^{^{_{_{B}}}} \hat{\varGamma
                           }^1_{_{AB}(1)}$$. Direct evaluation combined with use of the asymptotic Einstein Eq. (266) shows that $$F^{^{_A}}F^{^{_{_{B}}}} \hat{\varGamma
                           }^1_{_{AB}(0)}=0$$ (see section “Computer algebra” in “Appendix 3”), so that the limit evaluates to $$F^{^{_A}}F^{^{_{_{B}}}} \hat{\varGamma
                           }^1_{_{AB}(1)}$$. Further evaluation of Eq. (321) into computational $$\eth
                           $$ form is handled by computer algebra, as discussed in section “Computer algebra” in “Appendix 3”.

The attentive reader may have noticed that the derivation above used $$\tilde{\rho }=\rho
                           \omega $$ rather than $$\tilde{\rho }=\rho
                           (\omega +\rho A^\rho )$$, so that $$\partial _\rho \omega
                           $$ should not be taken as 0 but as $$A^\rho
                           $$. However, the corrections that would be introduced remain $${{\mathcal {O}}}(\rho
                           )$$ since (a) $$\hat{m}_{_{[G]}}^1=0$$, (b) in Eq. (319) the term $$\hat{g}^{11}A^\rho
                           $$ contained in $$\hat{g}^{1\gamma
                           }\partial _\gamma \omega $$ is $${{\mathcal {O}}}(\rho
                           ) A^\rho $$, and (c) in Eq. (321) the term $$F^A F^B
                           \hat{\varGamma }^1_{_{AB}}A^\rho $$ contained in $$F^A F^B
                           \hat{\varGamma }^\gamma _{_{AB}}\partial _\gamma \omega
                           $$ is also $${{\mathcal {O}}}(\rho
                           ) A^\rho $$.

#### The Newman–Penrose quantity $$\psi
                           ^0_4$$

The Newman–Penrose quantity $$\psi
                           _4$$, and its re-scaled version $$\psi
                           _4^0$$, were introduced in Sect. 3.3, and defined there for the case of physical space. Because the Weyl tensor is conformally invariant, it is straightforward to transform the earlier definition into one in the conformal gauge. Thus, in the conformal Bondi gauge,

322 $$\begin{aligned} \psi
                           ^0_4=\lim _{\tilde{\rho }=0} \frac{\hat{\tilde{C}}_{\alpha \beta \mu \nu
                           } \hat{\tilde{n}}_{_{[NP]}}^\alpha \bar{\hat{\tilde{m}}}^\beta
                           \hat{\tilde{n}}_{_{[NP]}}^\mu \bar{\hat{\tilde{m}}}^\nu }{\tilde{\rho }},
                           \end{aligned}$$

and again, as in the case of the news $${{\mathcal
                           {N}}}$$, the limiting process means that the metric variables need to be expressed as power series in $$\tilde{\rho
                           }$$. Calculating the Weyl tensor is discussed in section “Computer algebra” in “Appendix 3”, and the result is $$\psi ^0_4=\partial
                           ^2_{\tilde{u}}\partial _{\tilde{\rho
                           }}\bar{\tilde{J}}$$ as given in Eq. (275). The “Appendix” also checks that replacing $$\hat{\tilde{m}}^\alpha $$ in Eq. (322) by $$\hat{\tilde{m}}^\alpha
                           _{_{[G]}}$$ (with $$\tilde{m}^\alpha
                           _{_{[G]}}$$ defined in Eq. (293)) does not affect the result for $$\psi
                           ^0_4$$.

In this case, transforming Eq. (322) to the conformal general gauge is straightforward, since the tensor quantities are conformally invariant and the net weight is 0. The result is

323 $$\begin{aligned} \psi
                           ^0_4=\frac{1}{\omega }\lim _{\rho =0} \frac{\hat{C}_{\alpha \beta \mu \nu
                           }\hat{n}_{_{[NP]}}^\alpha \bar{\hat{m}}_{_{[G]}}^\beta
                           \hat{n}_{_{[NP]}}^\mu \bar{\hat{m}}_{_{[G]}}^\nu }{\rho },
                           \end{aligned}$$

where $$\hat{m}_{_{[G]}}^\alpha =e^{-i\delta
                           }(0,0,F^A)$$, and is further evaluated, by directly calculating the Weyl tensor, in section “Computer algebra” in “Appendix 3” (Babiuc et al. 2009) (but note that this reference uses a different approach to the evaluation of $$\psi
                           ^0_4$$).

### Linearized case

In the linearized case the Bondi–Sachs metric variables $$\beta
                        ,J,U,W_c$$ and the coordinate transformation variables $$u_0,A^u,(\omega
                        -1),A^\rho ,x^{^{_A}}_0,A^{^{_A}}$$ are regarded as small. Algebraically, the approximation is implemented by introducing a parameter $$\epsilon
                        =$$ max$$(|\beta
                        |,|J|,|U|,|W_c|)$$ in a neighbourhood of $$\mathcal
                        {J}^+$$. Then, the metric variables are re-written as $$\beta \rightarrow
                        \epsilon \beta $$ etc., and quantities such as $${{\mathcal {N}}},\psi
                        ^0_4$$ are expressed as Taylor series in $$\epsilon
                        $$ with terms $${{\mathcal
                        {O}}}(\epsilon ^2)$$ ignored, leading to considerable simplifications. It is common practice to assume that the error in the approximation is about $$\epsilon
                        ^2$$. While computational results do not contradict this assumption, a word of caution is needed: no work on establishing a formal error bound for this problem has been reported.

Equations (280)–(282) and Eq. (285), simplify to

324 $$\begin{aligned} \partial
                        _u u_0=(\omega -1)+2\beta ,\qquad \partial _u \phi
                        ^{^{_A}}_0=-U^{^{_A}},\qquad \partial _u \omega =-W_c/2, \;\; A=-\eth u_0.
                        \end{aligned}$$

It will also be useful to note the linearized form of Eq. (289),

325 $$\begin{aligned} J=\eth
                        X_0. \end{aligned}$$

In the linearized case, Eq. (312) takes the form (Bishop 2005)

326 $$\begin{aligned} 2+4
                        (\omega -1) +2\bar{\eth }\eth \omega =2+\frac{1}{2} \left( \eth
                        ^2\bar{J}+\bar{\eth }^2 J\right) , \end{aligned}$$

which is solved by decomposing $$\omega
                        $$ and *J* into spherical harmonic components

327 $$\begin{aligned} \omega
                        =1+\sum _{\ell \ge 2,|m|\le \ell }Y^{\ell \,m}\omega _{\ell \,m}, \;\;J=\sum
                        _{\ell \ge 2,|m|\le \ell }\,{}_2Y^{\ell \,m}J_{\ell \,m},
                        \end{aligned}$$

leading to

328 $$\begin{aligned} \omega
                        _{\ell \,m}(4-2\varLambda )=-\mathfrak {R}(J_{\ell \,m})\varLambda
                        (2-\varLambda ) \sqrt{\frac{1}{(\ell +2)\varLambda (\ell -1)}},
                        \end{aligned}$$

[recall that $$\varLambda =\ell (\ell
                        +1)$$] so that

329 $$\begin{aligned} \omega
                        _{\ell \,m}=-\frac{\varLambda }{2}\sqrt{\frac{1}{(\ell +2)\varLambda (\ell
                        -1)}}\mathfrak {R}(J_{\ell \,m}). \end{aligned}$$

Evaluating Eq. (321) for the news, and using Eq. (325), is discussed in section “Computer algebra” in “Appendix 3”. The result is Bishop (2005)

330 $$\begin{aligned}
                        {{\mathcal {N}}}=\frac{1}{2\rho }\left( \partial _u J+\eth U\right)
                        +\frac{1}{2} \left( \eth ^2 \omega +\partial _u\partial _\rho J+\partial
                        _\rho \eth U\right) . \end{aligned}$$

Now from the linearized asymptotic Einstein equations, $$\partial _u J+\eth
                        U=0$$ and $$\partial _\rho U-2\eth
                        \beta =0$$, so we get

331 $$\begin{aligned}
                        {{\mathcal {N}}}=\frac{1}{2} \left( \eth ^2 \omega +\partial _u\partial
                        _\rho J+2\eth ^2\beta \right) . \end{aligned}$$

The result is more convenient on decomposition into spherical harmonics, $${{\mathcal {N}}}=\sum
                        \,{}_2Y^{\ell \,m}{{\mathcal {N}}}_{\ell \,m}$$,

332 $$\begin{aligned}
                        {{\mathcal {N}}}_{\ell \,m}=-\frac{\varLambda \mathfrak {R}(J_{\ell
                        \,m})}{4} +\frac{\partial _u\partial _\rho J_{\ell \,m}}{2} +\sqrt{(\ell
                        +2)\varLambda (\ell -1)}\beta _{\ell \,m}.
                        \end{aligned}$$

In the linearized case, the evaluation of $$\psi
                        ^0_4$$ is straightforward, because the Weyl tensor is a first-order term so the null tetrad vectors need be correct only to zeroth order. As discussed in section “Computer algebra” in “Appendix 3”, we find (Babiuc et al. 2009)

333 $$\begin{aligned} \psi
                        ^0_4=\lim _{\tilde{\rho }\rightarrow 0}\left[ \frac{\partial _u\bar{\eth
                        }\bar{U}+\partial ^2_u\bar{J}}{\tilde{\rho }} +\frac{\rho }{\tilde{\rho }}
                        \left( -\bar{\eth }\bar{U} +\partial _u\partial _\rho \bar{\eth }\bar{U} -
                        \partial _u\bar{J} +\partial ^2_u\partial _\rho \bar{J} -\frac{1}{2}
                        \bar{\eth }^2W_c \right) \right] . \end{aligned}$$

It would appear that $$\psi
                        ^0_4$$ is singular, but applying the asymptotic Einstein equation Eq. (266) we see that these terms cancel; further, to linear order the deviation of $$\omega
                        $$ from unity is ignorable, so that

334 $$\begin{aligned} \psi
                        ^0_4=\left( - \bar{\eth }\bar{U} +\partial _u\partial _\rho \bar{\eth
                        }\bar{U} -\partial _u\bar{J} +\partial ^2_u\partial _\rho \bar{J}
                        -\frac{1}{2} \bar{\eth }^2W_c \right) .
                        \end{aligned}$$

Eq. (276) stated a relationship between $$\psi
                        ^0_4$$ and the news $${{\mathcal
                        {N}}}$$ which should remain true in this general linearized gauge. In order to see this, we modify Eq. (334) by applying Eq. (324), Eq. (264) and Eq. (266) to the terms $$W_c$$, $$\partial _\rho
                        \bar{U}$$ and $$\partial
                        _u\bar{J}$$, respectively, yielding

335 $$\begin{aligned} \psi
                        ^0_4=2\partial _u\bar{\eth }^2\beta + \bar{\eth }^2 \partial _u\omega
                        +\partial ^2_u\partial _\rho \bar{J},
                        \end{aligned}$$

from which it is clear that $$\psi ^0_4=2\partial
                        _u\bar{{\mathcal {N}}}$$.

In the linearized approximation, the wave strain Eq. (301) simplifies to Bishop and Reisswig (2014)

336 $$\begin{aligned}
                        H=\partial _\rho J-\eth A, \end{aligned}$$

and using Eq. (324) to replace *A*,

337 $$\begin{aligned}
                        H=\partial _\rho J+\eth ^2 u_0. \end{aligned}$$

An expression for $$u_0$$ is obtained using the first relationship in Eq. (324), $$\partial _u u_0=(\omega
                        -1)+2\beta $$, which is integrated to give $$u_0$$. It is clear that $$u_0$$ is subject to the gauge freedom $$u_0\rightarrow
                        u_0^\prime = u_0 + u_{_G}$$, provided that $$\partial _u
                        u_{_G}=0$$ so that $$u_{_G}=u_{_G}(x^{_A})$$. Thus the wave strain *H* is subject to the gauge freedom $$H\rightarrow H^\prime =
                        H + H_{_G}$$, where $$H_{_G}=\eth ^2 u_{_G}
                        (x^{_A})$$. Decomposing *H* into spherical harmonics, $$H=\sum \,{}_2Y^{\ell
                        \,m}H_{\ell \,m}$$, it follows that

338 $$\begin{aligned} H_{\ell
                        \,m}(u)=\partial _\rho J_{\ell \,m}(u)+\sqrt{(\ell +2)\varLambda (\ell -1)}
                        \int ^u \omega _{\ell \,m}(u^\prime )+2\beta _{\ell \,m}(u^\prime )
                        du^\prime , \end{aligned}$$

with $$\omega _{\ell
                        \,m}$$ given by Eq. (329). The gauge freedom now appears as a constant of integration for each spherical harmonic mode in Eq. (338). This freedom needs to be fixed by a gauge condition. Normally the spacetime is initially dynamic but tends to a final state that is static, for example the Kerr geometry. In such a case, we impose the condition $$H_{\ell
                        \,m}(u)\rightarrow 0$$ as $$u\rightarrow \infty
                        $$. The same gauge freedom would occur if the wave strain *H* were obtained by time integration of the news $${{\mathcal
                        {N}}}$$, in this case appearing as an arbitrary “constant” of integration $$f(\tilde{x}^{_A})$$.

## Numerical implementations of the characteristic approach

**Fig. 11:**
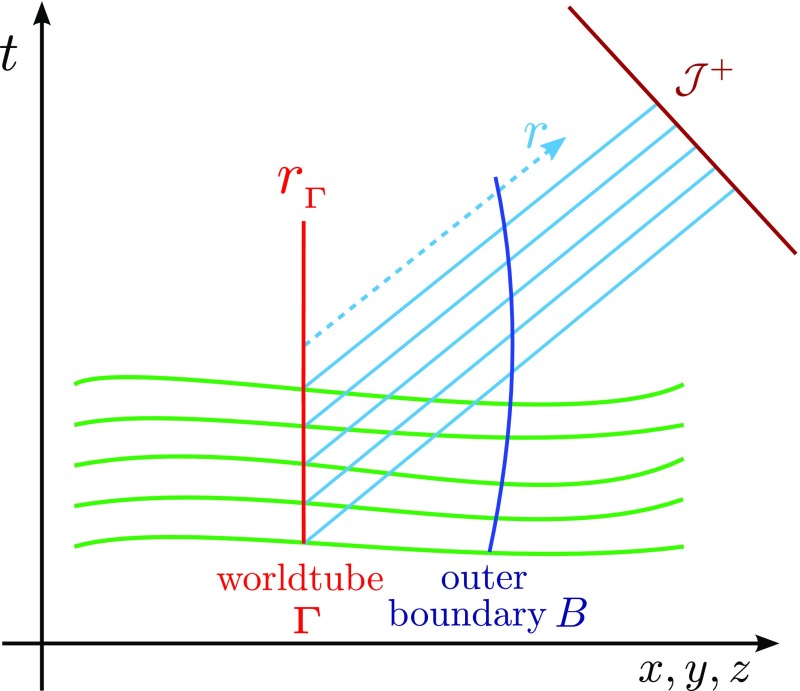
Schematic illustration of Cauchy-characteristic matching (CCM) and characteristic extraction(CE). In both cases there is a Cauchy evolution *green slices*, and a characteristic evolution *light blue slices* between $$r_\varGamma
                              $$ and $$\mathcal
                              {J}^+$$. The difference is that in CCM the outer boundary of the Cauchy evolution is at the worldtube $$r_\varGamma
                              $$ with boundary data supplied by the characteristic evolution; and in CE the outer boundary of the Cauchy evolution is as shown in *dark blue* and is subject to a boundary condition that excludes incoming gravitational waves

The idea of combining the “3+1” and characteristic approaches to extract the gravitational-wave signal from a numerical simulation was introduced in the 1990s (Bishop 1992, 1993). However, this early work focused on using the combination for the whole spacetime and was called Cauchy-characteristic matching (CCM). Subsequently, implementation difficulties with CCM led to the development of something less ambitious known as characteristic extraction (CE). Under certain conditions (which in practice are achievable), CE is just as accurate as CCM. The advantage of CCM, if it can be implemented, is that it has the potential to make a significant contribution to overall code efficiency (Bishop et al. 1996b). An outline of what is meant by CCM and CE, and the differences between the two approaches, is illustrated in Fig. 11 and described in the caption.

As first steps towards CCM in relativity, it was implemented for the model problem of a nonlinear scalar wave equation (Bishop et al. 1996a, 1997a) without any symmetries, and for the Einstein equations with a scalar field under the condition of spherical symmetry (Gómez et al. 1996; Lehner 2000). There has been a series of papers on CCM under axial symmetry (Clarke and d’Inverno 1994; Clarke et al. 1995; d’Inverno and Vickers 1997; d’Inverno et al. 2000; d’Inverno and Vickers 1996; Dubal et al. 1995, 1998). A detailed algorithm for CCM in relativity in the general case was presented in Bishop et al. (1999a). The stable implementation of matching is quite a challenge, and this goal has not yet been achieved (Szilágyi et al. 2000; Szilágyi 2000); although a stable implementation without symmetry has been reported with the Einstein equations linearized and using harmonic “3+1” coordinates (Szilágyi and Winicour 2003; Szilágyi et al. 2002). The issue of progress towards CCM is much more fully discussed in the review by Winicour (2005).

**Fig. 12:**
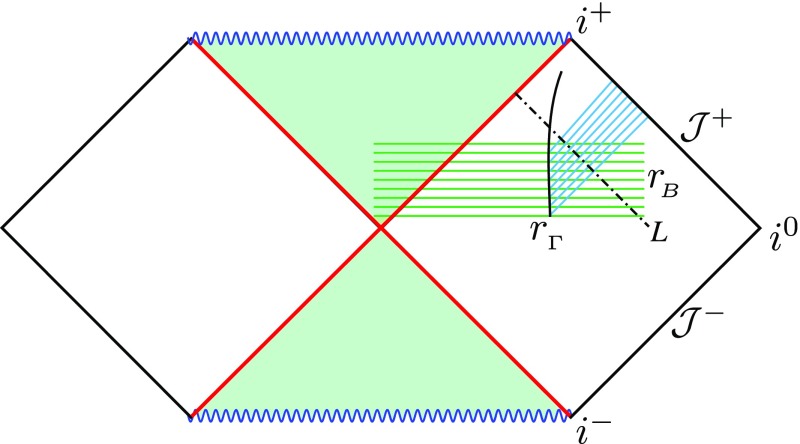
Portions of the Kruskal diagram that are determined numerically. The *green horizontal lines* indicate the region of spacetime that is determined by the Cauchy evolution and which has finite spatial extent with artificial outer boundary at $$r_{_B}$$. The *light blue diagonal lines* indicate the region that is determined by characteristic evolution, and which starts off from a worldtube $$\varGamma
                              $$ located at $$r_{_\varGamma
                              }$$ using boundary data from the Cauchy evolution. The future Cauchy horizon of the Cauchy initial data is indicated by the *dotted diagonal line* *L* parallel to $$\mathcal
                              {J}^+$$. As long as the worldtube $$\varGamma
                              $$ is located within the future Cauchy horizon, the numerically evolved subset of the spacetime is consistently determined

As a consequence of the difficulties with a stable implementation of CCM, in the 2000s attention shifted to the issue of developing CE, for which stability is not expected to be an issue. Further, although CCM has the advantage of high computational efficiency (Bishop et al. 1996b), it was realized that CE can be as accurate as CCM, provided the outer boundary of the Cauchy evolution is sufficiently far from the worldtube $$\varGamma
                     $$ that the two are not causally related, as indicated in Fig. 12. The implementation of CE for a test problem was described in 2005 (Babiuc et al. 2005). Subsequently, codes have been developed that yield useful results for the astrophysical problem of the inspiral and merger of two black holes (Reisswig et al. 2009; Reisswig 2010; Reisswig et al. 2010; Babiuc et al. 2011a, b). Work that uses, rather than develops, characteristic extraction includes (Ott 2011; Reisswig et al. 2011, 2013b). There is an alternative approach (Helfer 2010) that yields the emitted energy, momentum, and angular momentum, although it has not been implemented numerically.

The key feature of characteristic extraction is just a coordinate transformation, from Cauchy to characteristic coordinates in a neighbourhood of the worldtube $$\varGamma
                     $$. However, it is a more complicated procedure than it might appear, because the Bondi–Sachs radial coordinate *r* is a surface area coordinate, so it cannot be expressed explicitly in terms of the “3+1” coordinates. This complicates the matter in two ways

1. The coordinate transformation has to be made in two steps, firstly to a null coordinate system in which the radial coordinate is an affine parameter on the outgoing null radial geodesics, and secondly to Bondi–Sachs coordinates.
2. In general $$\varGamma
                              $$ is not a worldtube of constant *r*, so setting data at the innermost radial grid point of the Bondi–Sachs system requires special care.

Implementations of characteristic extraction mainly follow Bishop et al. (1999a), but do differ in certain aspects. Further, the field clearly needs to develop since most implementations are second-order accurate. This is a significant limitation since codes with higher order accuracy have existed for some time on the Cauchy side; and recently the first fourth order characterictic code has been reported (Reisswig et al. 2013a), as well as a spectral characteristic code (Handmer and Szilágyi 2015). An important recent development is the implementation of spectral characteristic extraction (Handmer et al. 2015, 2016), so that the whole computation is spectrally convergent. Below we outline the characteristic extraction procedure including some of the variations currently in use.

### Worldtube boundary data

Characteristic extraction is conceptually a post-processing procedure, as opposed to CCM in which the “3+1” and characteristic codes must run in step with each other. The transformation to Bondi–Sachs coordinates will require of the “3+1” data the full four-metric and all its first derivatives on the extraction worldtube $$\varGamma
                        $$. Thus the first issue is to consider precisely what data the “3+1” code should dump to file, for subsequent processing by the characteristic extraction code. The simplistic solution of just dumping everything is not practical, because the data set is too large, and this is even the case should the data dump be restricted to those grid-points that are close to $$\varGamma
                        $$. Some form of data compaction is required. On a given timeslice the extraction surface is spherical, so the natural compaction procedure is decomposition in terms of spherical harmonics. It turns out that this procedure also has some beneficial side-effects

1. It filters out high frequency noise.
2. It greatly simplifies the process of interpolation onto a regular angular grid.

Assuming that the “3+1” spacelike coordinates are approximately Cartesian $$x^i_{\scriptscriptstyle
                        [C]}=(x,y,z)$$, the extraction worldtube $$\varGamma
                        $$ is defined by

339 $$\begin{aligned}
                        R^2=x^2+y^2+z^2, \end{aligned}$$

for some fixed radius *R*. Of course the above is a simple coordinate-specific, rather than a geometric, definition; but in practice the definition has worked well and $$\varGamma
                        $$ has not exhibited any pathologies such as becoming non-convex. As indicated above, the extraction code will need the full four-metric and its first-derivatives, and it is a matter of choice as to whether conversion from the “3+1” variables (such as lapse, shift and three-metric) to four-metric is performed in the “3+1” code or in the extraction routine; for simplicity, this discussion will be on the basis that the conversion is performed in the “3+1” code. The conversion formulas are given in Eq. (75). The time derivatives of the four-metric could be found by finite differencing, but the results are likely to be less noisy if they can be expressed in terms of other variables in the “3+1” code—e.g., the “1+log” slicing condition and the hyperbolic $$\tilde{\varGamma
                        }$$-driver condition (Pollney et al. 2011), if being used, would mean that time derivatives of the lapse and shift are known directly, and the time derivative of the three-metric may be obtainable from the extrinsic curvature. For the spatial derivatives of the four-metric, it is sufficient to calculate and write to file only the radial derivative, since $$\partial
                        _x$$, $$\partial
                        _y$$, $$\partial
                        _z$$ can later be reconstructed from the radial derivative and angular derivatives of the spherical harmonics which are known analytically. The radial derivative in terms of the Cartesian derivatives is

340 $$\begin{aligned} \partial
                        _R = \frac{1}{R} \left( x\partial _x+y\partial _y+z\partial _z \right) .
                        \end{aligned}$$

Having calculated the above variables and derivatives at “3+1” grid points in a neighbourhood of $$\varGamma
                        $$, they must each be interpolated onto points on the coordinate sphere $$\varGamma
                        $$ using (at least) fourth-order interpolation. Then each quantity *A*, whether scalar, vector or tensor, is decomposed as

341 $$\begin{aligned} A_{\ell
                        \,m} = \int _{S^2} d\varOmega \, \bar{Y}^{\ell \,m} A(\varOmega ),
                        \end{aligned}$$

and the $$A_{\ell
                        \,m}$$ are written to file. The decomposition is performed for $$\ell \le \ell _{\max
                        }$$, and in practice $$\ell _{\max }\approx
                        8$$.

A variation of the above procedure was introduced by Babiuc et al. (2011a). Instead of calculating the radial derivative of a quantity *A*, the idea is to decompose *A* into a product of spherical harmonics and Chebyshev polynomials in *r*. More precisely, we consider a “thick” worldtube $$R_1<R<R_2$$ and the idea is to seek coefficients $$A_{k,\ell
                        ,m}$$ such that we may write

342 $$\begin{aligned} A=\sum
                        _{k,\ell ,m}A_{k\ell \, m}U^k(\tau (R))Y^{\ell \,m},
                        \end{aligned}$$

where $U^k$ is a Chebyshev polynomial of the second kind, and

343 $$\begin{aligned} \tau
                        (R)=\frac{2R-R_1-R_2}{R_2-R_1}, \end{aligned}$$

so that, within the thick worldtube, the argument of $$U^k$$ has the required range of −1 to 1. The coefficients $$A_{k,\ell
                        ,m}$$ are then determined by a least squares fit to the data at each “3+1” grid point within the thick worldtube. The decomposition is carried out for $$k\le k_{\max
                        }$$, and in practice (Babiuc et al. 2011a) takes $$k_{\max
                        }=6$$. Thus, this procedure involves writing three times as much data to file compared to that of calculating the radial derivative, but is probably more accurate and has the flexibility of being able to reconstruct data, including radial derivatives, at any point within the thick worldtube. Babiuc et al. (2011a) also introduced the option of calculating time derivatives via a Fourier transform process, so being able to filter out high frequency noise.

### Reconstruction from spectral modes

The variables are reconstructed via

344 $$\begin{aligned} A = \sum
                        _{\ell \, m} A_{\ell \,m} Y^{\ell \,m}\,
                        \end{aligned}$$

in the case of decomposition only into angular modes, or via Eq. (342) in the case of decomposition into both angular and radial modes. The radial derivatives at the extraction worldtube $$R=R_\varGamma
                        $$ are obtained either directly, or by analytic differentiation in the case that the reconstruction is in terms of Chebyshev polynomials. We then need to obtain the Cartesian derivatives in terms of radial and angular derivatives, and by the chain rule

345 $$\begin{aligned} \partial
                        _i A =\sum _{\ell \,m}\left( Y^{\ell \,m}\partial _i R\partial _{_R} A_{\ell
                        \,m} + A_{\ell \,m}\partial _i\phi ^{2}\partial _{\phi ^2} Y^{\ell \,m} +
                        A_{\ell \,m}\partial _i\phi ^{3}\partial _{\phi ^3} Y^{\ell \,m} \right) ,
                        \end{aligned}$$

where $$\phi ^{_A}=\phi ^2,\phi
                        ^3$$ are the angular coordinates. The angular derivatives of the $$Y^{\ell
                        \,m}$$ may be re-expresed in terms of spin-weighted spherical harmonics, and $$\partial _i \phi
                        ^{_A}$$ expressed explicitly in terms of the Cartesian coordinates. The details depend on the specific angular coordinates being used, and in the common case of stereographic coordinates the formulas are Reisswig et al. (2010)

346 $$\begin{aligned} \partial
                        _i A = \sum _{\ell \,m}\left( \frac{A_{\ell \,m}\sqrt{\ell (\ell
                        +1)}}{1+q^2+p^2} \left[ {}_1Y^{\ell \,m}(\partial _i q-i\partial _i p)-
                        {}_{-1}Y^{\ell \,m}(\partial _i q+i \partial _ip) \right] + \partial _i R
                        \partial _{_R} A_{\ell \,m}Y^{\ell \,m}\right) ,
                        \end{aligned}$$

where

347 $$\begin{aligned}&\displaystyle \partial _i R =
                        \partial _i\sqrt{x^2+y^2+z^2} = \frac{(x,y,z)}{R},
                        \end{aligned}$$

348 $$\begin{aligned}&\displaystyle \partial _i q =
                        \partial _i\left( \frac{x}{\sqrt{x^2+y^2+z^2}\pm z} \right) \nonumber
                        \\&\displaystyle = \frac{1}{(R\pm z)^2} \left( R\pm z-x^2/R,\,
                        -xy/R,\, -xz/R\mp x \right) , \end{aligned}$$

349 $$\begin{aligned}&\displaystyle \partial _i p\! =\!
                        \partial _i\left( \frac{\pm y}{\sqrt{x^2\!+\!y^2\!+\!z^2}\pm z} \right)
                        \!=\! \frac{1}{(R\pm z)^2}\left( \mp xy,\, \pm R+z\mp y^2/R,\, -y\mp yz/R
                        \right) ,\nonumber \\ \end{aligned}$$

where the upper sign is valid for the north patch and the lower sign is valid for the south patch.

### Transformation to null affine coordinates

**Fig. 13:**
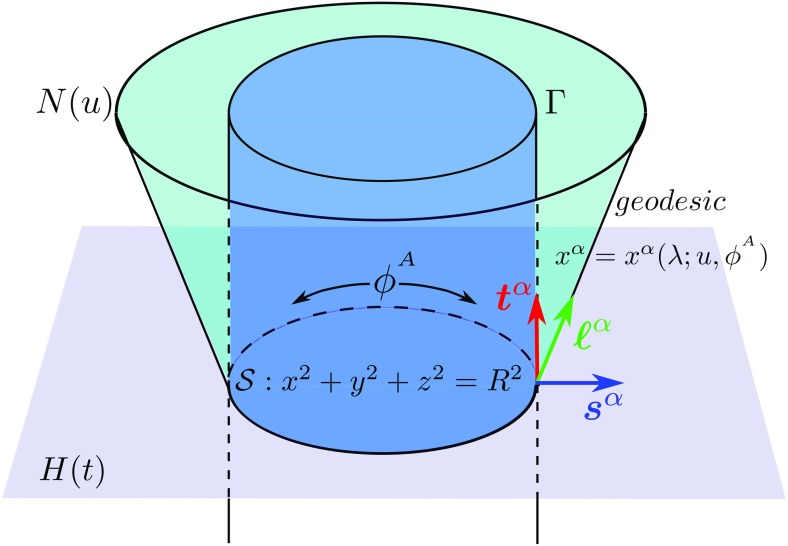
Schematic illustration of the (first stage) construction of characteristic coordinates and metric

In this section we construct the coordinate transformation from the Cartesian like “3+1” coordinates to a null coordinate system in which the radial coordinate is an affine parameter rather than the Bondi–Sachs surface area coordinate. As already mentioned, we need this intermediate step because the surface area coordinate cannot be expressed as a function of only the “3+1” coordinates, but would also need terms involving the three-metric $$\gamma
                        _{ij}$$. The procedure is illustrated schematically in Fig. 13.

On the extraction worldtube $$\varGamma
                        $$ we can simply define the coordinate transformation, but off $$\varGamma
                        $$ it will need to be calculated. The null affine coordinates are $$x_{\scriptscriptstyle
                        [N]}^\alpha =(u,\lambda ,q,p)$$, and the relation to the “3+1” coordinates $$x_{\scriptscriptstyle
                        [C]}^\alpha =(t,x,y,z)$$ on $$\varGamma
                        $$ is defined to be

350 $$\begin{aligned}
                        u=t,\;\lambda =0, \end{aligned}$$

with *q*, *p* given by Eq. (406) for $$r=R_\varGamma
                        $$.

Although the above is given in terms of stereographic angular coordinates (*q*, *p*), rather than general angular coordinates $$\phi
                        ^{^{_A}}$$, the formulas that follow will not be specific to stereographic coordinates.

The unit normal $$n^{\mu
                        }$$ to the hypersurface $$\varSigma
                        _{t}$$ is determined from the lapse and shift as stated in Eq. (71). Let $$s^{\alpha
                        }=(s^{i},0)$$ be the outward pointing unit normal to the section $$S_{t}$$ of the worldtube at time $$t^{n}$$. By construction, $$s^{i}$$ lies in the slice $$\varSigma
                        _{t}$$, and is given by Eq. (94). The generators $$\ell ^{\alpha
                        }$$ of the outgoing null cone through $$S_{t}$$ are given on the worldtube by

351 $$\begin{aligned} \ell
                        ^{\alpha } = \frac{n^{\alpha } + s^{\alpha }}{\alpha - \gamma _{ij} \beta
                        ^{i} s^{j}}, \end{aligned}$$

which is normalized so that $$\ell ^{\alpha }
                        t_{\alpha } = -1$$, where $$t^{\alpha } =
                        (1,0,0,0)$$ is the Cauchy evolution vector.

We may now build the coordinate transformation between the “3+1” Cartesian coordinates $$x^{\alpha
                        }$$ and the (null) affine coordinates $${y}^{\alpha
                        }$$. As already discussed, we need this in a neighborhood of the worldtube, not just on the worldtube. Along each outgoing null geodesic emerging from $$S_{t}$$, angular and time coordinates are defined by setting their values to be constant along the rays, and equal to their values on the worldtube. Geometrically, the idea is that we define (*u*, *q*, *p*) to be constant on each null geodesic generator. Algebraically, given $$(u,\lambda
                        ,q,p)$$, the “3+1” coordinates are given by

352 $$\begin{aligned}
                        x_{\scriptscriptstyle [C]}^{\alpha } = x_{\scriptscriptstyle
                        [C]}^{(0)}{}^{\alpha } + \ell ^{\alpha } \lambda + O(\lambda ^{2}),
                        \end{aligned}$$

where $$x_{\scriptscriptstyle
                        [C]}^{(0)}{}^{\alpha }$$ is given by Eq. (350), and where $$\ell ^{\alpha
                        }$$ is given by Eq. (351). This expression determines $$x_{\scriptscriptstyle
                        [C]}^{\alpha }(u,\lambda ,q,p)$$ to $$O(\lambda
                        ^{2})$$. Consequently, the calculation of any quantity off-$$\varGamma
                        $$ is restricted to be be second-order accurate. If higher order is required, we would need to take into account how the geodesic generators, i.e., the $$\ell ^{\alpha
                        }$$, vary off-$$\varGamma
                        $$, which would mean using information provided by the geodesic equation.

Then the metric $$g_{{\scriptscriptstyle
                        [N]}\alpha \beta }$$ in null affine coordinates $$x_{\scriptscriptstyle
                        [N]}^\alpha =(u,\lambda ,q,p)$$ is expressed in terms of the “3+1” metric as

353 $$\begin{aligned}
                        {g}_{{\scriptscriptstyle [N]}{\alpha }{\beta }}= \frac{\partial
                        x_{\scriptscriptstyle [C]}^{\mu }}{\partial x_{\scriptscriptstyle
                        [N]}^{\alpha }} \frac{\partial x_{\scriptscriptstyle [C]}^{\nu }}{\partial
                        x_{\scriptscriptstyle [N]}^{\beta }} g_{\mu \nu }.
                        \end{aligned}$$

The Jacobian of the coordinate transformation is now expressed as a series expansion in the parameter $$\lambda
                        $$. We do not need the $$\partial _\lambda
                        x_{\scriptscriptstyle [C]}^{\mu }$$ because the coordinate $$\lambda
                        $$ is an affine parameter of the null geodesics, which fixes the $$g_{{\scriptscriptstyle
                        [N]}\lambda {\mu }}$$:

354 $$\begin{aligned}
                        {g}_{{\scriptscriptstyle [N]}\lambda \lambda }= {g}_{{\scriptscriptstyle
                        [N]}\lambda {\scriptscriptstyle A}} = 0, \qquad {g}_{{\scriptscriptstyle
                        [N]}\lambda {u}} = -1, \end{aligned}$$

with the numerical value of the last condition a consequence of the normalization condition $$t^{\alpha }\ell _{\alpha
                        }=-1$$. The relevant part of the coordinate transformation is then

355 $$\begin{aligned} \partial
                        _\xi x_{\scriptscriptstyle [C]}^{\mu } \!:=\! \frac{\partial
                        x_{\scriptscriptstyle [C]}^{\mu }}{\partial x_{\scriptscriptstyle [N]}^{\xi
                        }} \!=\! \partial _\xi x_{\scriptscriptstyle [C]}^{(0)}{}^{\mu } \!+\!
                        \partial _\xi x_{\scriptscriptstyle [C]}^{(1)}{}^{\mu } \lambda \!+\!
                        O(\lambda ^{2}), \quad \partial _\xi x_{\scriptscriptstyle [C]}^{(1)}{}^{\mu
                        } \!=\!\partial _\xi \ell ^{(0)}{}^{\mu }, \quad \mathrm{for} \quad {\xi } =
                        (u,q,p). \end{aligned}$$

The order $${{\mathcal {O}}}(\lambda
                        ^0)$$ part of the Jacobian is evaluated by analytic differentation of Eq. (350). From Eq.(355), the $${{\mathcal {O}}}(\lambda
                        ^1)$$ part of the Jacobian is obtained from $$\partial _\xi \ell
                        ^{(0)}{}^{\mu }$$ with $${\xi } =
                        (u,q,p)$$; since $$\ell ^{(0)}{}^{\mu
                        }$$ is known on the worldtube analytically in the angular directions from the spherical harmonic decomposition, and analytically or on a regular (*q*, *p*, *u*) grid in the time direction, $$\partial _\xi \ell
                        ^{(0)}{}^{\mu }$$ can easily be found by analytic differentiation or finite differencing.

### Null affine metric

The $$\lambda
                        $$-derivative of the Cauchy 4-metric at the worldtube can be expressed as

356 $$\begin{aligned}
                        (\partial _\lambda g_{\alpha \beta })_{|\varGamma } = \partial _\mu
                        g^{(0)}_{\alpha \beta } \ell ^{(0)}{}^{\mu },
                        \end{aligned}$$

so that the null affine metric takes the form

357 $$\begin{aligned}
                        {g}_{{\scriptscriptstyle [N]}{\alpha }{\beta }} =
                        {g}^{(0)}_{{\scriptscriptstyle [N]}{\alpha }{\beta }} + \partial _\lambda
                        {g}_{{\scriptscriptstyle [N]}{\alpha }{\beta }} \lambda + O(\lambda ^{2}).
                        \end{aligned}$$

In the above Eq., the ${}^{(0)}$ coefficients are

358 $$\begin{aligned}
                        {g}^{(0)}_{{\scriptscriptstyle [N]}{u}{u}}&= g_{tt}{}_{|\varGamma },
                        \nonumber \\ {g}^{(0)}_{{\scriptscriptstyle [N]}{u}{\scriptscriptstyle
                        A}}&= \left( \partial _{_{A}}x_{\scriptscriptstyle [C]}^{i}
                        g_{it}{}\right) _{|\varGamma }, \nonumber \\ {g}^{(0)}_{\scriptscriptstyle
                        [N]AB}&= \left( \partial _{_{A}}x_{\scriptscriptstyle [C]}^{i}
                        \partial _{_{B}}x_{\scriptscriptstyle [C]}^{j} g_{ij}{}\right) _{|\varGamma
                        }, \end{aligned}$$

and the $$\lambda
                        $$ derivative coefficients are

359 $$\begin{aligned} \partial
                        _\lambda {g}_{{\scriptscriptstyle [N]}{u}{u}}&= \left[ \partial
                        _\lambda g_{tt} + 2\, \partial _u \ell ^{\mu } g_{\mu t}\right] _{|\varGamma
                        } + O(\lambda ), \nonumber \\ \partial _\lambda {g}_{{\scriptscriptstyle
                        [N]}{u} {\scriptscriptstyle A}}&= \left[ \partial _{_A}
                        x_{\scriptscriptstyle [C]}^{k} \left( \partial _u \ell ^{\mu } g_{k\mu } +
                        \partial _\lambda g_{kt} \right) +\partial _{_A} \ell ^{k} g_{kt} + \partial
                        _{_A}\ell ^{t} g_{tt} \right] _{|\varGamma } + O(\lambda ), \nonumber \\
                        \partial _\lambda {g}_{\scriptscriptstyle [N]AB}&= \left[ \partial
                        _{_A} x_{\scriptscriptstyle [C]}^{k} \partial _{_B}x_{\scriptscriptstyle
                        [C]}^{l} \partial _\lambda g_{kl} + \left( \partial _{_A} \ell ^{\mu }
                        \partial _{_B} x_{\scriptscriptstyle [C]}^{l} + \partial _{_B}\ell ^{\mu
                        }\partial _{_A} x_{\scriptscriptstyle [C]}^{l} \right) g_{\mu l} \right]
                        _{|\varGamma } + O(\lambda ), \end{aligned}$$

and where the $$\lambda
                        $$-derivatives of the Cauchy metric are evaluated as

360 $$\begin{aligned} \partial
                        _\lambda g_{\alpha \beta }=\ell ^\gamma \partial _\gamma g_{\alpha \beta }.
                        \end{aligned}$$

The contravariant null affine metric, $${g}_{\scriptscriptstyle
                        [N]}^{\alpha \beta }$$, is also expressed as an expansion in $$\lambda
                        $$,

361 $$\begin{aligned}
                        {g}_{\scriptscriptstyle [N]}^{{\mu }{\nu }} = g_{\scriptscriptstyle
                        [N]}^{(0)}{}^{{\mu }{\nu }} + \partial _\lambda {g}^{{\mu }{\nu
                        }}_{\scriptscriptstyle [N]} \lambda + O(\lambda ^{2}) .
                        \end{aligned}$$

The coefficients are obtained from the conditions

362 $$\begin{aligned}
                        {g}_{\scriptscriptstyle [N]}^{(0)}{}^{{\mu }{\alpha }}
                        {g}^{(0)}_{{\scriptscriptstyle [N]}{\alpha }{\nu }} = \delta ^{{\mu }}_{{\nu
                        }}, \qquad \partial _\lambda {g}^{{\mu }{\nu }}_{\scriptscriptstyle [N]} = -
                        {g}^{{\mu }{\alpha }} \, {g}_{\scriptscriptstyle [N]}^{{\beta }{\nu }} \,
                        \partial _\lambda {g}_{{\scriptscriptstyle [N]}{\alpha }{\beta }} ,
                        \end{aligned}$$

as well as the requirement that certain components are fixed (which follows from Eq. (354))

363 $$\begin{aligned}
                        {g}_{\scriptscriptstyle [N]}^{\lambda {u}} = -1, \qquad
                        {g}_{\scriptscriptstyle [N]}^{ u {\scriptscriptstyle A}} =
                        {g}_{\scriptscriptstyle [N]}^{{u}{u}} = 0 .
                        \end{aligned}$$

Thus the contravariant null affine metric and its $$\lambda
                        $$ derivative are

364 $$\begin{aligned}
                        {g}_{\scriptscriptstyle [N]}^{_{AB}} {g}_{\scriptscriptstyle [N]
                        BC}&= \delta ^{_{A}}_{\, \,_{C}}, \nonumber \\
                        {g}_{\scriptscriptstyle [N]}^{\lambda {\scriptscriptstyle A}}&=
                        {g}_{\scriptscriptstyle [N]}^{_{AB}} g_{{\scriptscriptstyle
                        [N]}u{\scriptscriptstyle B}}, \nonumber \\ {g}_{\scriptscriptstyle
                        [N]}^{\lambda \lambda }&= - g_{{\scriptscriptstyle [N]}{u}{u}} +
                        {g}_{\scriptscriptstyle [N]}^{\lambda {\scriptscriptstyle A}}
                        g_{{\scriptscriptstyle [N]}u{\scriptscriptstyle A}}, \nonumber \\ \partial
                        _\lambda g^{_{AB}}_{\scriptscriptstyle [N]}&= -
                        {g}_{\scriptscriptstyle [N]}^{_{AC}} {g}_{\scriptscriptstyle [N]}^{_{BD}}
                        \partial _\lambda g_{\scriptscriptstyle [N]CD}, \nonumber \\ \partial
                        _\lambda g^{\lambda {\scriptscriptstyle A}}_{\scriptscriptstyle
                        [N]}&= {g}_{\scriptscriptstyle [N]}^{_{AB}} \partial _\lambda \left(
                        g_{{\scriptscriptstyle [N]}{u}{\scriptscriptstyle B}} -
                        {g}_{\scriptscriptstyle [N]}^{\lambda {\scriptscriptstyle C}} \partial
                        _\lambda g_{\scriptscriptstyle [N]CB} \right) , \nonumber \\ \partial
                        _\lambda g^{\lambda \lambda }_{\scriptscriptstyle [N]}&= - \partial
                        _\lambda g_{{\scriptscriptstyle [N]}{u}{u}} + 2\, {g}_{\scriptscriptstyle
                        [N]}^{\lambda {A}}\partial _\lambda g_{{\scriptscriptstyle
                        [N]}{u}{\scriptscriptstyle A}} - {g}_{\scriptscriptstyle [N]}^{\lambda
                        {\scriptscriptstyle A}} {g}_{\scriptscriptstyle [N]}^{\lambda
                        {\scriptscriptstyle B}} \partial _\lambda g_{\scriptscriptstyle [N]AB} .
                        \end{aligned}$$

### Metric in Bondi–Sachs coordinates

We are now able to construct the surface area coordinate $$r(u,\lambda ,\phi
                        ^{_A})$$

365 $$\begin{aligned} r =
                        \left( \frac{\det ({g}_{\scriptscriptstyle [N]AB})}{\det (q_{_{AB}})}
                        \right) ^{{1}/{4}}. \end{aligned}$$

In order to make the coordinate transformation $$x_{\scriptscriptstyle
                        [N]}^{\alpha }=(u,\lambda ,\phi ^{_A}) \rightarrow x_{\scriptscriptstyle
                        [B]}^{\alpha }=(u,r,\phi ^{_A})$$, we need expressions for $$\partial _\lambda
                        r$$, $$\partial _{_A}
                        r$$ and $$\partial _u
                        r$$. From Eq. (365),

366 $$\begin{aligned} \partial
                        _\lambda r&= \frac{r}{4} {g}_{\scriptscriptstyle [N]}^{_{AB}}
                        \partial _\lambda g_{\scriptscriptstyle [N]AB},
                        \end{aligned}$$

367 $$\begin{aligned} \partial
                        _{_C} r&= \frac{r}{4} \left( {g}_{\scriptscriptstyle [N]}^{_{AB}}
                        \partial _{_C} g_{\scriptscriptstyle [N]AB} - q^{^{_{AB}}}\partial _{_C}
                        q_{_{AB}} \right) , \end{aligned}$$

where

368 $$\begin{aligned} \partial
                        _{_C} g_{\scriptscriptstyle [N]AB} = \left( \partial _{_A}\partial _{_C}
                        x_{\scriptscriptstyle [C]}^{i}\, \partial _{_B}x_{\scriptscriptstyle
                        [C]}^{j} + \partial _{_A}x_{\scriptscriptstyle [C]}^{i}\, \partial
                        _{_B}\partial _{_C}x_{\scriptscriptstyle [C]}^{j} \right) g_{ij} + \partial
                        _{_A}x_{\scriptscriptstyle [C]}^{i}\,\partial _{_B} x_{\scriptscriptstyle
                        [C]}^{j}\, \partial _{_C}x_{\scriptscriptstyle [C]}^{k}\, \partial _k g_{ij}
                        , \end{aligned}$$

in which the $$\partial _{_A}\partial
                        _{_C}x_{\scriptscriptstyle [C]}^{i}$$ are evaluated analytically in terms of $$\phi
                        ^{_A}$$. An expression for $$\partial _u
                        r$$ will be required later but only on the worldtube $$\varGamma
                        $$, so that Eq. (358) may be used when simplifying $$\partial
                        _u$$ applied to Eq. (365); further on $$\varGamma
                        $$, $$\partial _u=\partial
                        _t$$, and by construction $$\partial
                        _{_A}x_{\scriptscriptstyle [C]}^{i}$$ is independent of time. Thus,

369 $$\begin{aligned} \partial
                        _u r&=\, \frac{r}{4}\, {g}_{\scriptscriptstyle
                        [N]}^{\scriptscriptstyle AB} \partial _u {g}_{\scriptscriptstyle [N] AB}
                        \nonumber \\&=\,\frac{r}{4}\, {g}_{\scriptscriptstyle
                        [N]}^{\scriptscriptstyle AB} \partial _u\left( \partial
                        _{_A}x_{\scriptscriptstyle [C]}^{i}\, \partial _{_B}x_{\scriptscriptstyle
                        [C]}^{j}\, g_{ij} \right) \nonumber \\&= \, \frac{r}{4}\,
                        {g}_{\scriptscriptstyle [N]}^{\scriptscriptstyle AB} \partial
                        _{_A}x_{\scriptscriptstyle [C]}^{i}\, \partial _{_B}x_{\scriptscriptstyle
                        [C]}^{j}\,\partial _t g_{ij}. \end{aligned}$$

The metric $$g_{\scriptscriptstyle
                        [B]}^{\alpha \beta }$$ in Bondi–Sachs coordinates is obtained from the coordinate transformation

370 $$\begin{aligned}
                        g_{\scriptscriptstyle [B]}^{\alpha \beta } = \frac{\partial
                        x_{\scriptscriptstyle [B]}^{\alpha }}{\partial x_{\scriptscriptstyle
                        [N]}^{\mu }} \frac{\partial x_{\scriptscriptstyle [B]}^{\beta }}{\partial
                        x_{\scriptscriptstyle [N]}^{\nu }} g_{\scriptscriptstyle [N]}^{{\mu }{\nu
                        }}. \end{aligned}$$

Note that the spherical part of the metric is unchanged, i.e., $$g_{\scriptscriptstyle
                        [B]}^{\scriptscriptstyle AB}=g_{\scriptscriptstyle [N]}^{\scriptscriptstyle
                        AB}$$, and only the components $$g_{\scriptscriptstyle
                        [B]}^{11}$$, $$g_{\scriptscriptstyle
                        [B]}^{1{\scriptscriptstyle A}}$$ and $$g_{\scriptscriptstyle
                        [B]}^{01}$$ on $$\varGamma
                        $$ need to be determined. From Eq. (363),

371 $$\begin{aligned}&\displaystyle
                        g_{\scriptscriptstyle [B]}^{11} = \partial _\alpha \, \partial _\beta r\,
                        g_{\scriptscriptstyle [N]}^{{\alpha }{\beta }} = \left( \partial _\lambda
                        r\right) ^2 g_{\scriptscriptstyle [N]}^{11} + 2\, \partial _\lambda r\,
                        \left( \partial _{_A}r\,g_{\scriptscriptstyle [N]}^{1 {\scriptscriptstyle
                        A}} - \partial _u r \right) +\partial _{_A} r\,\partial _{_B}r\,
                        g_{\scriptscriptstyle [N]}^{_{AB}}, \nonumber \\&\displaystyle
                        g_{\scriptscriptstyle [B]}^{1{\scriptscriptstyle A}} = \partial _\alpha r\,
                        g_{\scriptscriptstyle [N]}^{{\alpha } {\scriptscriptstyle A}} =\partial
                        _\lambda r\, g_{\scriptscriptstyle [N]}^{1 {\scriptscriptstyle A}} +
                        \partial _{_{B}}r\, g_{\scriptscriptstyle [N]}^{_{AB}}, \nonumber
                        \\&\displaystyle g_{\scriptscriptstyle [B]}^{01} = \partial
                        _{{\alpha }}r\, g_{\scriptscriptstyle [N]}^{0{\alpha }} = - \partial
                        _{\lambda }r. \end{aligned}$$

As discussed in Sect. 6.1, the characteristic Einstein equations are not formulated directly in terms of the metric components, but in terms of quantities derived from the metric, specifically $$J,\beta
                        ,U$$ and $$W_c$$. Explicitly, the relations between these quantities and the contravariant metric components are

372 $$\begin{aligned}
                        J=-\frac{q_{_{A}} q_{_{B}} g_{\scriptscriptstyle [B]}^{^{_{AB}}}}{2 r^2},
                        \qquad \beta =-\frac{1}{2} \log (g_{\scriptscriptstyle [B]}^{01}), \qquad
                        U=\frac{g_{\scriptscriptstyle [B]}^{1A}}{g_{\scriptscriptstyle
                        [B]}^{01}}q_{_{A}}, \qquad W_c=-\frac{g_{\scriptscriptstyle
                        [B]}^{11}+g_{\scriptscriptstyle [B]}^{01}}{g_{\scriptscriptstyle
                        [B]}^{01}r}. \end{aligned}$$

### Starting up the null code at the worldtube

As already mentioned, a difficulty faced is that Eq. (372) gives metric quantities on the worldtube $$\varGamma
                        $$, which is not in general a hypersurface at a constant value of the *r*-coordinate. The original method for tackling the problem makes use of a Taylor series in $$\lambda
                        $$ (Bishop et al. 1999a), and has been implemented in Szilágyi et al. (2000), Szilágyi (2000), Babiuc et al. (2005), Reisswig et al. (2009) and Reisswig et al. (2010). Recently, a method that uses a special integration algorithm between the worldtube and the first characteristic grid-point, has been proposed and tested (Babiuc et al. 2011a, b). Both approaches are outlined below.

#### Taylor series method

The Taylor series method is based on writing, for some quantity *A*,

373 $$\begin{aligned}
                           A(u,\lambda ,q,p)=A(u,0,q,p)+\lambda \partial _{\lambda }A(u,0,q,p)
                           +{{\mathcal {O}}}(\lambda ^2), \end{aligned}$$

where *A* represents $$J,\beta
                           ,U$$ and $$W_c$$. *A* needs to be written as a function of null affine coordinates, so that $$\partial _{\lambda
                           }A$$ can be evaluated. Also, using Eqs. (365) and (367) evaluated on the worldtube, we need to find the value of $$\lambda
                           $$ at which $$r(\lambda
                           )=r_i$$, where $$r_i$$ is an *r*-grid-point near the worldtube; this needs to be done for each grid-point in both the angular and time domains. The derivation of the Taylor expansions is straightforward, with second $$\lambda
                           $$-derivatives eliminated using the Einstein equations (Bishop et al. 1999a). The results are

374 $$\begin{aligned}
                           \partial _\lambda J= & {} -\frac{1}{2\,r^{2}} q_{_A} q_{_B}
                           \partial _\lambda g^{^{_{AB}}}_{\scriptscriptstyle [N]} -
                           2\,\frac{\partial _\lambda r}{r} J,
                           \end{aligned}$$

375 $$\begin{aligned}
                           \partial _\lambda \beta= & {} \frac{r}{8}\, \partial _\lambda r
                           \left( \partial _\lambda J \partial _\lambda \bar{J}- \frac{1}{1 + J
                           \bar{J}} \left( \bar{J}\partial _\lambda J +\partial _\lambda \bar{J}
                           J\right) ^2 \right) . \end{aligned}$$

376 $$\begin{aligned}
                           \partial _\lambda U= & {} - \left( \partial _\lambda
                           g^{1{\scriptscriptstyle A}}_{\scriptscriptstyle [N]} + \frac{\partial
                           _\lambda \partial _{_B} r}{\partial _\lambda r} g_{\scriptscriptstyle
                           [N]}^{_{AB}} + \frac{\partial _{_B}r}{\partial _\lambda r} \partial
                           _\lambda g^{_{AB}}_{\scriptscriptstyle [N]} \right) q_{_{A}} + 2 \,
                           \partial _\lambda \beta \left( U + g_{\scriptscriptstyle
                           [N]}^{1{\scriptscriptstyle A}} q_{_{A}} \right) ,
                           \end{aligned}$$

377 $$\begin{aligned}
                           \partial _\lambda W_{c}= & {} - \frac{\partial _\lambda r}{r}
                           \left( \left( \frac{\partial _\lambda r}{r} + 2\, \partial _\lambda \beta
                           \right) g_{\scriptscriptstyle [N]}^{11} - \partial _\lambda
                           g^{11}_{\scriptscriptstyle [N]} - \frac{1}{r} \right) + \frac{2}{r}
                           \left( \frac{\partial _\lambda r \partial _u r}{r} - \partial _\lambda
                           \partial _u r \right) \nonumber \\&+ \frac{2}{r} \left( \partial
                           _\lambda \partial _{_A}r - \frac{\partial _\lambda r\partial _{_A}r}{r}
                           \right) g_{\scriptscriptstyle [N]}^{1{\scriptscriptstyle A}} +
                           2\frac{\partial _{_A}r}{r}\,\partial _\lambda g^{1{\scriptscriptstyle
                           A}}_{\scriptscriptstyle [N]} \nonumber \\&+ \frac{\partial
                           _{_B}r}{r\,\partial _\lambda r} \left( 2 \, \partial _\lambda \partial
                           _{_A}r g_{\scriptscriptstyle [N]}^{_{AB}} + 2 \, \partial _\lambda \beta
                           \partial _{_A}r + \partial _{_A}r \partial _\lambda
                           g^{_{AB}}_{\scriptscriptstyle [N]} \right) - \frac{\partial _{_A}r \,
                           \partial _{_B}r}{r^2}\, g_{\scriptscriptstyle [N]}^{_{AB}}.
                           \end{aligned}$$

#### Special evolution routine between the worldtube and the first radial grid-point

In this approach, on a null cone say $$u=u_n$$, we need only the values of the Bondi–Sachs metric variables at the angular grid-points on the worldtube. We also suppose that the value of *J* is known at all grid-points of the Bondi–Sachs coordinate system on the given null cone, either as initial data or from evolution from the previous null cone. A mask is set to identify those radial grid-points for which $$x_i-x_\varGamma
                           < \varDelta x$$, and these points will be called “B points”. The special algorithm is concerned with setting data at the points $$i=B+1$$, called “B+1 points”. The first hypersurface equation in the hierarchy is the one for $$\beta
                           $$, and is the simplest one to hadle. The algorithm is

378 $$\begin{aligned} \beta
                           _{{\scriptscriptstyle B}+1}=\beta _\varGamma +\varDelta _r
                           \frac{r_{{\scriptscriptstyle B}+1} +r_\varGamma }{16\varDelta _r^2}\left(
                           (J_{{\scriptscriptstyle B}+1}-J_\varGamma )(\bar{J}_{{\scriptscriptstyle
                           B}+1}-\bar{J}_\varGamma ) -(K_{{\scriptscriptstyle B}+1}-K_\varGamma )^2
                           \right) , \end{aligned}$$

where $$\varDelta
                           _r=r_{{\scriptscriptstyle B}+1}-r_{_\varGamma
                           }$$. The local truncation error associated with this algorithm is $${{\mathcal
                           {O}}}(\varDelta _r^3)$$. The remaining hypersurface equations involve angular derivatives, which cannot be evaluated on the worldtube because it is not, in general, a hypersurface of constant *r*. Consequently, the right hand sides of these equations are evaluated at the B+1 points rather than at the points mid-way between $$r_{{\scriptscriptstyle B}+1}$$ and $$r_{\scriptscriptstyle
                           \varGamma }$$. Schematically, the hypersurface equations are of the form $$(r^n
                           A)_{,r}=f$$, and the algorithm is

379 $$\begin{aligned}
                           A_{{\scriptscriptstyle B}+1}=\frac{r_{\scriptscriptstyle \varGamma }^n
                           A_{\scriptscriptstyle \varGamma } +\varDelta _r f_{{\scriptscriptstyle
                           B}+1}}{r_{{\scriptscriptstyle B}+1}^n}.
                           \end{aligned}$$

The result is that the local truncation error for these equations is reduced to $${{\mathcal
                           {O}}}(\varDelta _r^2)$$. Even so, one start-up step with error $${{\mathcal
                           {O}}}(\varDelta _r^2)$$ is consistent with the global error of $${{\mathcal
                           {O}}}(\varDelta _x^2)$$.

Since the value of *r* varies on the worldtube, it may happen that the angular neighbour of a B+1 point is a B point. Thus, the code must also set data for the metric variables at the B points, even though much of this data will not be needed.

### Initial data

The above discussion has shown how data should be set at, or on a neighbourhood of, the inner worldtube $$\varGamma
                        $$, but in order to run a characteristic code data for *J* is also required on an initial null cone $$u=$$ constant. Earlier work has adopted the simplistic but unphysical approach of just setting $$J=0$$, assuming that the error so introduced would quickly be eliminated from the system. Babiuc et al. (2011a) and Bishop et al. (2011) investigated the matter. It was found that the error due to simplistic initial data is usually small, but it can take a surprisingly long time, up to 800 M, until saturation by other effects occurs. In terms of observations by a gravitational-wave detector, the effect of the error in search templates is not relevant. However, if a signal is detected, the effect would be relevant for accurate parameter estimation at large SNR (signal to noise ratio), but no quantitative estimates have been given.

Two methods for setting physically realistic initial data for a characteristic evolution have been proposed and tested. In Bishop et al. (2011) the initial data is set by means of fitting the boundary data to a general form of a linearized solution to the vacuum Einstein equations. On the other hand, Babiuc et al. (2011a) sets the initial data by means of the simple condition

380 $$\begin{aligned}
                        J=J|_\varGamma \frac{r_\varGamma }{r},
                        \end{aligned}$$

as in this case there should be no incoming radiation since the Newman–Penrose quantity $$\psi
                        _0=0$$.

### Implementation summary

The issues summarized here are: (1) setting up a characteristic code that starts from the output of a “3+1” code; (2) estimating the gravitational waves from metric data in a compactified domain output by a characteristic code; (3) estimating quantities derived from the gravitational waves, i.e., the energy, momentum and angular momentum.

#### Setting worldtube boundary data for the characteristic code

The coding of characteristic extraction is a complex process, and is not simply a matter of implementing a few of the formulas derived earlier in this section. Below, we outline the key steps that are required. The reader is also referred to section “Numerical codes” in “Appendix 3” for information about computer code that implements characteristic extraction.

1. Within the “3+1” code, write a routine that uses Eq. (342) to perform a spectral decomposition of the three-metric, lapse and shift, and outputs the data to file.
2. In a front-end to the characteristic code, write a routine that reads the data from the file created in the previous step, and reconstructs the four-metric and its first derivatives at the angular grid-points of the extraction worldtube.
3. Construct the generators $$\ell ^\alpha $$ of the outgoing null cone using Eq. (351), and then the Jacobian $$\partial x_{\scriptscriptstyle [C]}^{\mu
                                    }/\partial x_{\scriptscriptstyle [N]}^{\alpha
                                    }$$ as a series expansion in the affine paramenter $$\lambda $$, for each angular grid-point on the worldtube.
4. As described in Sect. 7.4, construct the null affine metric $$g_{{\scriptscriptstyle [N]}\alpha \beta
                                    }$$ and its first $$\lambda $$-derivative at the angular grid-points of the extraction worldtube; then construct the contravariant forms $$g_{\scriptscriptstyle [N]}^{\alpha \beta
                                    }$$ and $$\partial _\lambda g_{\scriptscriptstyle
                                    [N]}^{\alpha \beta }$$.
5. From Eq. (365), determine the surface area coordinate *r* and its first derivatives at the angular grid-points of the extraction worldtube.
6. Construct the Jacobian $$\partial x_{\scriptscriptstyle [B]}^{\mu
                                    }/\partial x_{\scriptscriptstyle [N]}^{\alpha
                                    }$$, and thus the Bondi–Sachs metric $$g_{\scriptscriptstyle [N]}^{\alpha \beta
                                    }$$ and then the metric coefficients $$\beta ,J,U,W_c$$ at the angular grid-points of the extraction worldtube.
7. Implement either of the special start-up procedures described in Sect. 7.6.
8. The construction of a characteristic code is not described in this review, but see section “Numerical codes” in “Appendix 3” for information about the availability of such codes.

#### Estimation of gravitational waves

In Sect. 6, many formulas used $$\rho
                           $$ (=1 / *r*) as the radial coordinate, but that is unlikely to apply in practice. In the case that the radial code coordinate is *x* given by Eq. (259), the relation between $$\partial
                           _x$$ and $$\partial _\rho
                           $$ at $$\mathcal
                           {J}^+$$ is

381 $$\begin{aligned}
                           \partial _\rho = -\frac{ \partial _x}{r_\varGamma }.
                           \end{aligned}$$

If all the metric coefficients near $$\mathcal
                           {J}^+$$ are small (which, in practice, is often but not always the case), then the linearized formulas apply, and:

- $\psi ^0_4$ is evaluated using Eq. (334).
- The news ${{\mathcal {N}}}$ is evaluated, decomposed into spherical harmonics, using Eq. (332).
- The strain *H* is evaluated, decomposed into spherical harmonics, using Eq. (338).

In the general (nonlinear) case, it is first necessary to evaluate the coordinate transformation functions $$\phi
                           _0^{_A}(u,x^{_A})$$, $$\omega
                           (u,x^{_A})$$ and $$u_0(u,x^{_A})$$. The reason for doing so is that it is then possible to determine, at each $$(u,\phi
                           ^{_A})$$ grid point, the corresponding values of the Bondi gauge coordinates $$(\tilde{u},\tilde{\phi
                           }^{_A})$$. Thus the gravitational-wave quantities can be expressed as functions of the (physically meaningful) Bondi gauge coordinates, rather than as functions of the code coordinates. This issue did not arise in the linearized case because it is a second-order effect and thus ignorable. The procedure for evaluating these functions is:

- $$\phi
                                    _0^{_A}(u,x^{_A})$$. Solve the evolution problem Eq. (281) with initial data $$\phi
                                    _0^{_A}(0,x^{_A})=0$$. This initial condition assumes that the initial data for *J* has been set with $$J=0$$ at $$\mathcal {J}^+$$.
- $$\omega (u,x^{_A})$$. Either solve the evolution problem Eq. (282) with initial data $$\omega (0,x^{_A})=1$$, or evaluate the explicit formula Eq. (287).
- $$u_0(u,x^{_A})$$. Solve the evolution problem Eq. (280). In this case, there is a gauge freedom to set the initial data $$u_0(0,x^{_A})$$ arbitrarily.

In the cases of $${{\mathcal
                           {N}}}$$ and $$\psi
                           ^0_4$$, the phase factor $$\delta
                           (u,x^{_A})$$ also needs to be evaluated. This can be done either explicitly, Eq. (295), or by solving the evolution problem Eq. (296) with initial data $$\delta
                           (0,x^{_A})=0$$. Then:

- $\psi ^0_4$ is evaluated using Eq. (323).
- The news ${{\mathcal {N}}}$ is evaluated using Eq. (321).
- The strain *H* is evaluated using Eq. (301).

#### Energy, momentum and angular momentum in the waves

The formulas for the energy, momentum and angular momentum have already been given in terms of $$\psi
                           _4$$ in Sect. 3.3.3, and these formulas are directly applicable here on substituting $$\psi
                           _4$$ by $$\psi
                           ^0_4/r$$. The resulting formulas involve one or two time integrals of $$\psi
                           ^0_4$$, and it is useful to note that here all such integration can be avoided by using

382 $$\begin{aligned} \int
                           _{-\infty }^t \int _{-\infty }^{t^\prime }\psi ^0_4 \, dt^\prime \,
                           dt^{\prime \prime } =\bar{H},\quad \int _{-\infty }^t \psi ^0_4 \,
                           dt^\prime = 2\bar{{\mathcal {N}}}.
                           \end{aligned}$$

## A comparison among different methods

This review has described the following methods for extracting the gravitational-wave signal from a numerical simulation

- The quadrupole formula, including various modifications, leading to the wave strain $$(h_+,h_\times
                              )$$;
- $$\psi
                              _4$$ (fixed radius) and $$\psi
                              _4$$ (extrapolation), leading to the Newman–Penrose quantity $$\psi
                              _4$$;
- Gauge-invariant metric perturbations, leading to the wave strain $$(h_+,h_\times
                              )$$;
- Characteristic extraction, leading to the wave strain $$(h_+,h_\times
                              )$$, the gravitational news $${\mathcal
                              {N}}$$, or the Newman–Penrose quantity $$\psi
                              _4$$.

There are a number of factors that need to be taken into account in deciding the appropriate method for a particular simulation. In outline, these factors are:

- *Physical problem motivating the simulation*. The most appropriate method for extracting gravitational waves is affected by how the result is to be used. It may be that only moderate accuracy is required, as would be the case for waveform template construction for use in searches in detector data; on the other hand, high accuracy would be needed for parameter estimation of an event in detector data at large SNR. Further, the purpose of the simulation may be not to determine a waveform, but to find the emitted momentum of the radiation and thus the recoil velocity of the remnant.
- *Domain and accuracy of the simulation*. The domain of the simulation may restrict the extraction methods that can be used. All methods, except that using the quadrupole formula, require the existence of a worldtube, well removed from the domain boundary, on which the metric is Minkowskian (or Schwarzschild) plus a small correction. As discussed in Sect. 3.3.2, extrapolation methods need these worldtubes over an extended region. Further, the accuracy of the simulation in a neighbourhood of the extraction process clearly limits the accuracy that can be expected from any gravitational-wave extraction method.
- *Ease of implementation of the various extraction methods*. All the methods described in this review are well understood and have been applied in different contexts and by different groups. Nevertheless, the implementation of a new gravitational-wave extraction tool will always require some effort, depending on the method, for coding, testing and verification.
- *Accuracy of the various extraction methods*. Theoretical estimates of the expected accuracy of each method are known, but precise data on actual performance is more limited because suitable exact solutions are not available. In a simulation of a realistic astrophysical scenario, at least part of the evolution is highly nonlinear, and the emitted gravitational waves are oscillatory and of varying amplitude and frequency. On the other hand, exact solutions are known in the linearized case with constant amplitude and frequency, or in the general case under unphysical conditions (planar or cylindrical symmetry, or non-vacuum). One exception is the Robinson–Trautman solution (Robinson and Trautman 1962), but in that case the gravitational waves are not oscillatory and instead decay exponentially.
  Thus, in an astrophysical application, the accuracy of a computed waveform is estimated by repeating the simulation using a different method; then the difference between the two waveforms is an estimate of the error, provided that it is in line with the theoretical error estimates. In some work, the purpose of comparing results of different methods is not method testing, but rather to provide validation of the gravitational-wave signal prediction. The only method that is, in principle, free of any systematic error is characteristic extraction, but the method was not available for general purpose use until the early 2010s. It should also be noted that there remains some uncertainty about factors that could influence the reliability of a computed waveform (Boyle 2016).

### Comparisons of the accuracy of extraction methods

A number of computational tests have been reported, in which the accuracy of various extraction methods is compared. Such tests are, of course, specific to a particular physical scenario, and to the choice of “3+1” evolution code, initial data, gauge conditions, etc. Some of the tests reported are now outlined, together with the results that were obtained. While it is natural to want to generalize these results, a word of caution is needed since the testing that has been undertaken is quite limited. Thus any generalization should be regarded as providing only a guide to which there may well be exceptions.

- Nagar et al. (2005) investigates various modifications of the standard quadrupole formula in comparison to results obtained using gauge-invariant metric perturbations for the case of oscillating accretion tori. Good results are obtained when back-scattering is negligible, otherwise noticeable differences in amplitude occur.
- Balakrishna et al. (2006) computes $\psi _4$ (fixed radius) and gauge-invariant metric perturbations for gravitational waves from boson star perturbations, but detailed comparisons between the two methods were not made.
- Pollney et al. (2007) compares gauge-invariant metric perturbations to $\psi _4$ (fixed radius) extraction for the recoil resulting from a binary black hole merger. It was found that results for the recoil velocities are consistent between the two extraction methods.
- Shibata et al. (2003) and Baiotti et al. (2009) compare gauge-invariant metric perturbations, modified quadrupole formula and $\psi _4$ (fixed radius) extraction for a perturbed neutron star. While the results are generally consistent, each method experienced some drawback. The gauge-invariant method has a spurious initial junk component that gets larger as the worldtube radius is increased. In $\psi _4$ extraction, fixing the constants of integration that arise in obtaining the wave strain can be a delicate issue, although such problems did not arise in this case. The generalized quadrupole formula led to good predictions of the phase, but to noticeable error in the signal amplitude.
- Reisswig et al. (2009, 2010) compare $\psi ^0_4$ from characteristic extraction and from $\psi _4$-extrapolation for Binary Black Hole (BBH) inspiral and merger in spinning and non-spinning equal mass cases. The “3+1” evolution was performed using a finite difference BSSNOK code (Pollney et al. 2011). A comparison was also made in Babiuc et al. (2011a) for the equal mass, non-spinning case. Recently, a more detailed investigation of the same problem and covering a somewhat wider range of BBH parameter space, was undertaken (Taylor et al. 2013) using SpEC for “3+1” evolution (Szilágyi et al. 2009).
  These results lead to two main conclusions. (1) The improved accuracy of characteristic extraction is not necessary in the context of constructing waveform templates to be used for event searches in detector data. (2) Characteristic extraction does provide improved accuracy over methods that extract at only one radius. The $\psi _4$-extrapolation method performs better, and there are results for $\psi ^0_4$ that are equivalent to characteristic extraction in the sense that the difference between the two methods is less than an estimate of other errors. However, that does not apply to all modes, particularly the slowly varying $m=0$ “memory” modes.
- A study of gravitational-wave extraction methods in the case of stellar core collapse (Reisswig et al. 2011) compared characteristic extraction, $\psi _4$ extraction (fixed radius), gauge-invariant metric perturbations, and the quadrupole formula. In these scenarios, the quadrupole formula performed surprisingly well, and gave results for the phase equivalent to those obtained by characteristic extraction, with a small under-estimate of the amplitude. However, quadrupole formula methods fail if a black hole forms and the region inside the horizon is excised from the spacetime. The gauge-invariant metric perturbation method gave the poorest results, with spurious high frequency components introduced to the signal. In characteristic extraction and $\psi _4$ extraction the waveform was obtained via a double time integration, and the signal was cleaned up using Fourier methods to remove spurious low frequency components.
- It is only very recently (Bishop and Reisswig 2014) that a method was developed in characteristic extraction to obtain the wave strain directly instead of via integration of ${{\mathcal {N}}}$ or $\psi ^0_4$. That work also compared the accuracy of the waveform obtained to that found from integration of $\psi _4^0$ using $\psi _4$-extrapolation, in two cases—a binary black hole merger, and a stellar core collapse simulation. When comparing the wave strain from characteristic extraction to that found by time integration of $\psi ^0_4$, good agreement was found for the dominant (2,2) mode, but there were differences for $\ell \ge 4$.

### Electronic supplementary material

Below is the link to the electronic supplementary material.

Supplementary material 1 (zip 163 KB)

## Figures and Tables

**Fig. 14:**
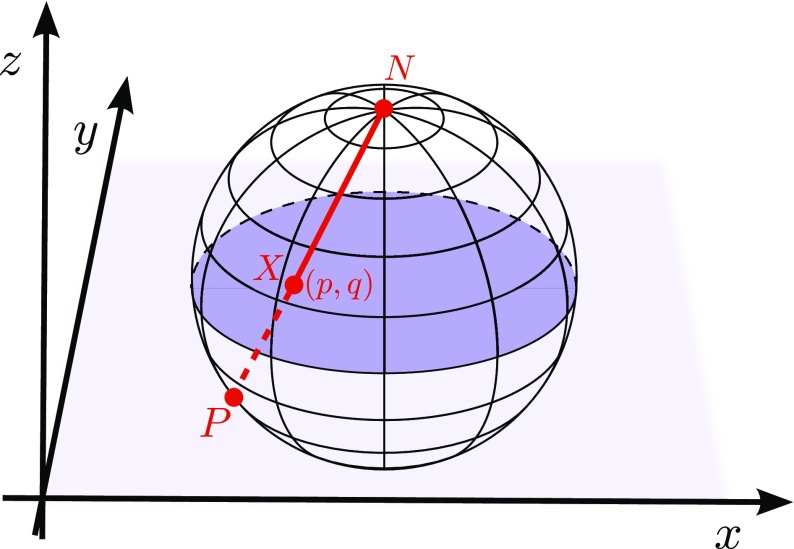
Illustration of stereographic coordinates. Consider a unit sphere with centre at the origin of Cartesian (*x*, *y*, *z*) coordinates. Construct a straight line from the north pole *N* of the sphere (at $x=y=0,z=1$) to a point *P* on the sphere, and let the *line* meet the plane $z=0$ at *X*. Let the *Cartesian* coordinates of *X* be $(x,y,z)=(q_{_{[S]}},p_{_{[S]}},0)$, then the southern patch *stereographic* coordinates of *P* are $(q_{_{[S]}},p_{_{[S]}})$. The northern patch stereographic coordinates are constructed in a similar way, with the *straight line* in this case starting from the south pole

## Footnotes

1 Note that the “bar” operator can in principle be applied also to the trace so that ${\bar{h}}=-h$.

2 Note that the orthogonality condition fixes three and not four components since one further constraint needs to be satisfied, i.e., $$\kappa ^{\mu } A_{\mu
                        \nu } u^{\nu } = 0$$.

3 Of course no gravitational waves are present in Newton’s theory of gravity and the formula merely estimates the time variations of the quadrupole moment of a given distribution of matter.

4 An alternative notation is to mark with an upper left index $^{(4)}$ the four-dimensional tensors and to leave unmarked the three-dimensional ones (Baumgarte and Shapiro 2010; Gourgoulhon 2012).

5 The values of $r_k$ may vary with $t_*$ since the extraction spheres are constructed to be of constant coordinate radius *s*.

6 In the case of non-oscillatory modes, usually with $$m=0$$, fitting to the real and imaginary parts of $$\psi _4^{\ell
                        m}$$ is preferred.

7 All of our discussion hereafter will deal with perturbative analyses in the time domain. However, a hybrid approach is also possible in which the perturbation equations are solved in the frequency domain. In this case, the source terms are given by time-dependent perturbations created, for instance, by the motion of matter and computed by fully nonlinear three-dimensional codes (Ferrari et al. 2006).

8 These results were originally obtained by Regge and Wheeler (1957) and by Zerilli (1970a, b) in a specific gauge (i.e., the Regge–Wheeler gauge). Subsequently, the work of Moncrief showed how to reformulate the problem in a gauge-invariant form by deriving the equations from a suitable variational principle (Moncrief 1974).

9 We recall that in the notation of Cunningham et al. (1978) $$\widetilde{\psi } =
                        \varLambda (\varLambda -2)\varPsi ^{(\mathrm o)}$$, and the multipoles in Cunningham et al. (1978) are related to ours as $$\widetilde{h}_2 = h_2 =
                        -2h$$, $$\widetilde{h}_{0} =
                        h^{(\mathrm o)}_{0}$$ and $$\widetilde{h}_{1} =
                        h^{(\mathrm o)}_{1}$$.

10 Note that in principle the gauge invariant quantities are independent of radius [cf. Eq. (219)]. In practice, however, their amplitudes may reach the correct asymptotic value only at sufficiently large distances. For this reason the extraction is in practice performed at different extraction radii and the amplitudes compared for convergence to an asymptotic value (Rupright et al. 1998; Rezzolla et al. 1999a).

11 This is a standard choice in modern numerical-relativity codes but there are no restrictions on the choice of the coordinate system.

12 There is a misprint in Eq. (A3) of the journal version of the reference, which has been corrected in the version on the arXiv, and also in Reisswig et al. (2013a).

13 This result applies only to conformal space, not to physical space.

## Appendix 1: Notation

### Latin symbols

$A_{\mu \nu }, A_+,A_\times$: Wave amplitude tensor and coefficients
$A,A^A,A^u,A^\rho$: Coefficients in transformation between Bondi and general gauges
*a*: Term in Schwarzschild metric
*b*: Term in Schwarzschild metric
$$C_{abcd}$$: Weyl tensor
$d\varOmega$: Surface area element of a sphere
*E*: Energy
$E^{\ell m}_{_A}$: Vector spherical harmonic
$$\varvec{e}_{+},\varvec{e}_{\times },\varvec{e}_{_\mathrm{L}},\varvec{e}_{_\mathrm{R}}$$: Polarization tensors
$\varvec{e}_x$: Unit vector in the direction of the coordinate *x*
$F^{_A}$: Dyad of general Bondi–Sachs gauge metric
${{\mathcal {F}}}$: Fourier transform operator
$$G_{ab}$$: Einstein tensor
*G*: Quantity in Cauchy-perturbative matching
$$g_{\alpha \beta }$$: four-metric
Background metric
*H*: Re-scaled wave strain, i.e., $\lim _{\tilde{r}\rightarrow \infty } r(h_++ih_\times )$
$H_0,H_1$: Quantity in Cauchy-perturbative matching
$h_{_{AB}}$: Angular part of metric
$$h_{\mu \nu }$$: Metric perturbation
$h^{^\mathrm{TT}}_{\mu \nu }$: Metric perturbation in TT gauge
$\bar{h}_{\mu \nu }$: Trace-free metric perturbation
$h^{(0)}_{_A}, h$: Quantity in Cauchy-perturbative matching
Trace-less mass quadrupole
$\mathcal {J}^+$: Future null infinity
*J*: Bondi–Sachs metric variable
$$J_i$$: Angular momentum
*K*: Bondi–Sachs metric variable
*K*: Quantity in Cauchy-perturbative matching
$$K_{ij}$$: Extrinsic curvature
$k_{_A}$: Quantity in Cauchy-perturbative matching
$\mathcal {L}$: Lie derivative operator
$L^{\ell m}, L^{\ell m}_{_A}$: Quantities in Cauchy-perturbative matching
$\ell ^\alpha$: Newman–Penrose tetrad vector, tangent to outgoing null geodesic
$m^\alpha$: Newman–Penrose tetrad vector
$$m_{_{[G]}}^\alpha $$: Approximation to $$m^\alpha $$
$${{\mathcal {N}}}$$: Gravitational news
$N^\mu _\nu$: Time projection operator
$n_{_{[NP]}}^\alpha$: Newman–Penrose tetrad vector
$n^\alpha$: Unit normal to $\varSigma _t$
$$P_i$$: three-momentum
*p*: Stereographic coordinate
$Q^{(0)}$: Quantity in Cauchy-perturbative matching
*q*: Stereographic coordinate
$q^{^{_A}}$: Complex dyad vector
$q_{_{AB}}$: Unit sphere metric
*R*: “3+1” radial coordinate, $$R=\sqrt{x^2+y^2+z^2}$$
$R, R_{ab}, R_{abcd}$: Ricci scalar, Ricci tensor, Riemann tensor
$${\mathcal {R}}$$: Intrinsic 2-curvature
*r*: Bondi–Sachs radial coordinate
*r*: Radial coordinate in Cauchy perturbative approach
$r_*$: “Tortoise” radial coordinate
$S^{(0)}$: Quantity in Cauchy-perturbative matching
$$S_t$$: $$\varGamma \cap \varSigma _t$$
$S^{\ell m}_c,S^{\ell m}_{cd}$: Vector spherical harmonic, tensor spherical harmonic
*s*: Radial like coordinate
$s^i$: Unit normal to $S_t$
$T_{ab}$: Stress-energy tensor
$T^{\ell m}, T^{\ell m}_{_A}$: Quantities in Cauchy-perturbative matching
$t_{ab}$: Stress-energy tensor of gravitational waves (averaged)
*t*: “3 + 1” time coordinate
*U*: Bondi–Sachs metric variable
$U^k(x)$: Chebyshev polynomial of the second kind
*u*: Bondi–Sachs time coordinate
$u^\mu$: Fluid four-velocity
$V_\ell ^{(0)}$: Quantity in Cauchy-perturbative matching
*W*: Lorentz factor
$W^{\ell m}$: Quantities in Cauchy-perturbative matching
$W_c$: Bondi–Sachs metric variable
$$x^\alpha , x^i$$: Coordinates
$x_{\scriptscriptstyle [B]}^\alpha$: Bondi–Sachs coordinates
$x_{\scriptscriptstyle [C]}^\alpha$: Minkowski-like coordinates
$x_{\scriptscriptstyle [N]}^\alpha$: Null affine coordinates
$X^{\ell m}$: Quantities in Cauchy-perturbative matching
$Y^{\ell \,m},{}_sY^{\ell \,m}$: Spherical harmonic, spin-weighted version
$Z^{\ell \,m}{}_sZ^{\ell \,m}$: “Real” spherical harmonic, spin-weighted version
$Z^{\ell \,m}_{_{CD}}$: Tensor spherical harmonic

### Greek symbols

$$\alpha $$: Lapse
$\beta$: Bondi–Sachs metric variable
$$\beta ^i$$: Shift
$$\varGamma $$: Extraction worldtube
$$\gamma _{ij}$$: three-metric
$$\delta $$: Phase factor
$\eta _{\mu \nu }$: Metric of special relativity
$\theta$: Spherical polar coordinate
$\kappa _\alpha$: Wave propagation vector
$\kappa _1,\kappa _2$: Quantities in Cauchy-perturbative matching
$$\varLambda $$: $$\ell (\ell + 1)$$
$$\lambda $$: Affine parameter
$\xi ^\alpha$: Vector defining gauge transformation
$$\xi $$: (*u*, *q*, *p*)
$\rho$: Compactified radial coordinate
$$\varSigma _t$$: Timelike slice
$\Phi ^{(o)},\Phi ^{(e)}$: Quantities in Cauchy-perturbative matching
$$\phi ^{^{_A}}$$: Angular coordinates
$\phi$: Spherical polar coordinate
$\Upsilon$: $- q^{\scriptscriptstyle A}\bar{q}^{\scriptscriptstyle B} \nabla _{\scriptscriptstyle A} q_{\scriptscriptstyle B}/2$, factor in definition of $\eth$
$\chi$: Quantity in Cauchy-perturbative matching
$\varPsi ^{(o)},\varPsi ^{(e)}$: Quantities in Cauchy-perturbative matching
$\psi _0 \cdots \psi _4$: Newman–Penrose quantities
$$\psi _4^0$$: Re-scaled $$\psi _4$$, i.e., $$\lim _{r\rightarrow \infty } r\psi _4$$
$$\varOmega _\mu $$: $$\nabla _\mu t$$
$\omega$: Relation between the radial coordinate in the general and Bondi gauges
$$\omega $$: Wave frequency

### Operators

$\nabla _\alpha$: Covariant derivative operator
$\partial _\alpha$: Partial derivative operator
$$\Box $$: Wave operator
$\eth$: Spin-weighted angular derivative operator
$\hat{ }$: Quantity in conformally compactified gauge
$\hat{ }$: Index in another coordinate system
$\tilde{ }$: Quantity in Bondi gauge
$\tilde{ }$: Fourier transformed quantity
$\bar{ }$: Complex conjugate (except see above for $\bar{h}_{\mu \nu }$)

### Indexing conventions

$${}_{\alpha ,\beta ,\ldots }$$: (0, 1, 2, 3) Spacetime indices
$${}_{i,j,\ldots }$$: (1, 2, 3) Spacelike indices
$${}_{\scriptscriptstyle A,B, \ldots }$$: (2, 3) Angular indices
${}_{a,b,\ldots }$: (0, 1) Non-angular indices
${}_{\ell m}$: Spherical harmonic indices

## Appendix 2: Spin-weighed spherical harmonics and the $\eth$ operator

A convenient way to represent vector and tensor quantities over the sphere, including their angular derivatives, is to use spin-weighted quantities and the $$\eth
                           $$ operator. The formalism was introduced by Newman and collaborators in the 1960s (Newman and Penrose 1966; Goldberg et al. 1967), and has been described in textbooks such as Penrose and Rindler (1984, 1986) and Stewart (1990). Even so, the theory is not well known and there are variations in notation and conventions (which topic is discussed further in Sect. 1), so the theory will be presented here in some detail based on the conventions of Gómez et al. (1997). We will describe the theory, both in general terms and with specific reference to the coordinates commonly used, i.e., spherical polar and stereographic. Further, the spin-weighted spherical harmonic functions will be introduced, as well as the vector and tensor spherical harmonics (Newman and Silva-Ortigoza 2006). All of these can be used as basis functions on the sphere.

### The complex dyad

Let $$\phi
                              ^{^{_A}}$$, $$q_{_{AB}}$$ and $$q^{^{_{AB}}}$$ ($$2\le A,B \le
                              3$$) be coordinates and the associated metric of a unit sphere. For example, in standard spherical polars, $$\phi
                              ^{^{_A}}=(\theta ,\phi )$$ and

383 $$\begin{aligned}
                              q_{_{AB}}=\left( \begin{array}{cc} 1 &{}\quad 0 \\ 0
                              &{}\quad \sin ^2\theta \end{array} \right) .
                              \end{aligned}$$

The first step is to introduce a complex dyad $$q^{^{_A}}$$. Geometrically, the dyad is a 2-vector that can be written as $$q^{^{_A}}=\mathfrak {R}(q^{^{_A}})+i\mathfrak
                              {I}(q^{^{_A}})$$ where $$\mathfrak
                              {R}(q^{^{_A}})$$, $$\mathfrak
                              {I}(q^{^{_A}})$$ are real and orthonormal. In other words, the real and imaginary parts of $$q^{^{_A}}$$ are unit vectors at right-angles to each other. From this definition, it is straightforward to verify the following properties

384 $$\begin{aligned}
                              q^{^{_A}} q_{_{A}}=0,\; q^{^{_A}} \bar{q}_{_{A}}=2,\;
                              q_{_{AB}}=\frac{1}{2}\left( q_{_{A}} \bar{q}_{_{B}}+\bar{q}_{_{A}}
                              q_{_{B}}\right) . \end{aligned}$$

Clearly, the dyad $$q^{^{_A}}$$ is not unique being arbitary up to a rotation and/or reflection, so that $$p^{^{_A}}=e^{i\gamma
                              }q^{^{_A}}$$ and $$p^{^{_A}}=\bar{q}^{^{_A}}$$ are also dyads. Even so, it is convenient to write the dyad in a way that is natural to the coordinates being used, which for a diagonal metric means that the real part of the dyad should be aligned to the $$\phi
                              ^2$$ direction, and the imaginary part to the $$\phi
                              ^3$$ direction. Thus, the dyad usually used in spherical polar coordinates is

385 $$\begin{aligned}
                              q^{^{_A}}=\left( 1,\frac{i}{\sin \theta }\right) , \hbox { and }
                              q_{_{A}}=(1,i\sin \theta ). \end{aligned}$$

The possibility of different parities, i.e., of a reflection, introduces an additional complication, which can be avoided by convention: All the coordinate systems used in this review are right-handed, and we will not consider dyads that are related by complex conjugation. This is equivalent to embedding the sphere into Euclidean space with coordinates $$(r,\phi ^2,\phi
                              ^3)$$ that are always *right-handed*, i.e., the vector product $$\mathfrak
                              {R}(\varvec{q}) \times \mathfrak
                              {I}(\varvec{q})$$ points in the positive *r*-direction.

The dyad $$q^{^{_A}}$$ resembles the angular components of the tetrad vector $$m^\alpha
                              $$ given in Eq. (96) when evaluated on a unit sphere, but there is a difference of a factor of $$\sqrt{2}$$. This is an example of different conventions used by different authors, which matter is discussed further in Sect. 1.

### Spin-weighted fields

Having defined the dyad $$q^{^{_A}}$$, we are now in a position to construct spin-weighted fields from vector and tensor fields. In the simplest case, suppose that $$\mu (\phi ^2,\phi
                              ^3)$$ is a scalar field. Then $$\mu
                              $$ is also a spin-weighted field with spin-weight $$s=0$$. Given a vector field $$v_{_{A}}(\phi
                              ^2,\phi ^3)$$, we define

386 $$\begin{aligned}
                              V=q^{^{_A}} v_{_{A}} \end{aligned}$$

to be a field with spin-weight $$s=1$$. Since *V* is a complex quantity, it contains two independent fields and thus uniquely represents the two components of $$v_{_{A}}$$. We can also define the quantity $$\bar{V}=\bar{q}^{^{_A}}
                              v_{_{A}}$$ with spin-weight $$s=-1$$, but of course it is not independent of *V*. From a second-rank tensor $$t_{_{AB}}$$ we can construct two independent fields

387 $$\begin{aligned}
                              T=q^{^{_A}} q^{^{_{_{B}}}} t_{_{AB}},\; \tau =q^{^{_A}}
                              \bar{q}^{^{_{_{B}}}} t_{_{AB}},
                              \end{aligned}$$

with spin-weights $$s=2$$ and $$s=0$$ respectively. Together *T* and $$\tau
                              $$ have 4 independent fields to represent the 4 components of $$t_{_{AB}}$$. In general, given a tensor field $$w_{A\cdots
                              D}$$, the quantity

388 $$\begin{aligned}
                              W=w_{_{A \cdots BC\cdots D}}q^{^{_A}} \cdots q^{^{_{_{B}}}}
                              \bar{q}^{^{_C}} \cdots \bar{q}^{^{_D}},
                              \end{aligned}$$

with *m* factors *q* and *n* factors $\bar{q}$ is defined to be a quantity with spin-weight $s=m-n$.

Spin-weighted quantities may also be defined in terms of their transformation properties. However, here, we use the definition above and later derive the transformation rule Eq. (398).

### Differentiation and the $\eth$ operator

We would now like to define derivative operators $$\eth
                              $$ and $$\bar{\eth
                              }$$ that act on spin-weighted fields, and that are consistent with covariant differentiation. This means that if *W* is defined as in Eq. (388), then we would like

389 $$\begin{aligned}
                              \eth W=\nabla _{_{_E}}w_{_{A \cdots BC\cdots D}}q^{^{_A}} \cdots
                              q^{^{_{_{B}}}} \bar{q}^{^{_C}} \cdots \bar{q}^{^{_D}} q^{^{_E}},\;
                              \bar{\eth } W =\nabla _{_{_E}}w_{_{A \cdots BC\cdots D}} \cdots
                              q^{^{_{_{B}}}} \bar{q}^{^{_C}} \cdots \bar{q}^{^{_D}} \bar{q}^{^{_E}}.
                              \end{aligned}$$

We see immediately that, if *W* has spin-weight *s*, then $$\eth
                              W$$ has spin-weight $$s+1$$ and $$\bar{\eth }
                              W$$ has spin-weight $$s-1$$. We achieve the desired effect by the definition

390 $$\begin{aligned}
                              \eth W=q^{^{_A}} \partial _{_{A}} W + s \Upsilon W,\;\; \bar{\eth }
                              W=\bar{q}^{^{_A}} \partial _{_{A}} W - s \bar{\Upsilon } W
                              \end{aligned}$$

where

391 $$\begin{aligned}
                              \Upsilon =-\frac{1}{2} q^{^{_A}}\bar{q}^{^{_{_{B}}}} \nabla
                              _{_{_A}}q_{B}. \end{aligned}$$

(Normally, the quantity denoted here by $$\Upsilon
                              $$ is given the notation $$\varGamma
                              $$; but we use the notation $$\Upsilon
                              $$ because $$\varGamma
                              $$ is used to represent the extraction worldtube). We demonstrate the above for a spin-weight 1 field $$V=q^{^{_A}}
                              v_{_{A}}$$. Starting from the definition Eq. (389) and the ansatz Eq. (390) with $$\Upsilon
                              $$ to be determined, we obtain

392 $$\begin{aligned}
                              q^{^{_A}}\partial _{_{_A}}(q^{^{_{_{B}}}} v_{_{B}}) +\Upsilon
                              q^{^{_C}} v_{_{C}} =q^{^{_A}} q^{^{_{_{B}}}} \partial _{_{_B}}v_{_{A}}
                              -q^{^{_A}} q^{^{_{_{B}}}} v_{_{C}} \varGamma ^{^{_C}}_{_{AB}},
                              \end{aligned}$$

and thus

393 $$\begin{aligned}
                              v_{_{C}}\left( q^{^{_C}}\Upsilon +q^{^{_A}}\left( \partial
                              _{_{_A}}q^{^{_C}} +q^{^{_{_{B}}}}\varGamma ^{^{_C}}_{_{AB}}\right)
                              \right) =0 \end{aligned}$$

after renaming some dummy indices. This must be true for all $v_{_{C}}$, and the bracketed term is just a covariant deriavtive, so we have

394 $$\begin{aligned}
                              q^{^{_C}}\Upsilon +q^{^{_A}} \nabla _{_{_A}}q^{^{_C}}=0.
                              \end{aligned}$$

We lower the free superscript ${}^{^{_C}}$ and then contract with $\bar{q}^{^{_C}}$ to obtain the desired result.

It should be noted that, in general, $$\eth
                              $$ and $$\bar{\eth
                              }$$ do not commute. The commutator is

395 $$\begin{aligned}
                              (\bar{\eth }\eth - \eth \bar{\eth })W= 2sW,
                              \end{aligned}$$

so that the operators commute only in the case of a quantity with spin-weight $s=0$.

### Coordinate transformation of spin-weighted quantities: Rotation factors $\exp (i\gamma )$

Spin-weighted quantities are defined in a way that they have no free tensorial indices so it would appear that they are scalars, but this is misleading because different dyads are used in the different coordinate systems. Suppose that we have two coordinate systems $$S_{(q)}$$ and $$S_{(p)}$$ with natural dyads $$\varvec{q}$$ and $$\varvec{p}$$ respectively. Each dyad has components in each of the coordinate systems, and so we define $$q^{^{_A}}_{(q)},
                              q^{^{_A}}_{(p)}$$ to mean the components of $$\varvec{q}$$ in $$S_{(q)},S_{(p)}$$ respectively; and similarly for $$p^{^{_A}}_{(q)}$$ and $$p^{^{_A}}_{(p)}$$. Assuming that $$S_{(q)}$$ and $$S_{(p)}$$ have the same parity, then their dyads are related by a rotation and, as discussed just after Eq. (384),

396 $$\begin{aligned}
                              p^{^{_A}}_{(p)}=\exp (i\gamma )q^{^{_A}}_{(p)}.
                              \end{aligned}$$

Suppose that $\varvec{v}$ is a vector and that $V_{(q)},V_{(p)}$ are the corresponding spin-weighted quantities with respect to the dyads $\varvec{q},\varvec{p}$ respectively. Thus

397 $$\begin{aligned}
                              V_{(p)}=p^{^{_A}}_{(p)} v_{(p){\scriptscriptstyle A}}=\exp (i\gamma
                              )q^{^{_A}}_{(p)}v_{(p){\scriptscriptstyle A}}= \exp (i\gamma
                              )q^{^{_A}}_{(q)}v_{(q){\scriptscriptstyle A}}=\exp (i\gamma
                              )V_{(q)}.\nonumber \\ \end{aligned}$$

Generalizing to the case where *V* is defined with *m* factors $q^A$ and $m-s$ factors $\bar{q}^A$ so that the spin-weight is *s*, we find

398 $$\begin{aligned}
                              V_{(p)}&= V_{(q)} \exp (i m \gamma )\exp (-i(m-s)\gamma
                              )\nonumber \\&=V_{(q)} \exp (i s\gamma ).
                              \end{aligned}$$

### Specific coordinate systems

It is very convenient to define coordinate systems on the unit sphere in terms of a coordinate transformation from a Cartesian system. The Cartesian coordinates are denoted by $$x^a_{_{[C]}}=(x,y,z)$$, and the spherical coordinates by $$x^a=(r,\phi
                              ^2,\phi ^3)$$. We use computer algebra to construct the Jacobian of the transformation bewteen spherical and Cartesian coordinates, and then to find the components of the dyad with respect to the Cartesian system. We use the notation $$Q^i$$ to denote the components of a dyad with respect to Cartesian coordinates. The computer algebra also evaluates the quantity $$\Upsilon
                              $$ used in the definition of the $$\eth
                              $$ operator Eq. (391), see section “Computer algebra” in “Appendix 3”.

#### Spherical polar coordinates

We use coordinates $(r,\theta ,\phi )$ for standard spherical polar coordinates. Only one coordinate patch is required, but the coordinate system is singular at the poles $\theta =0$ and $\theta =\pi$. The relation to Cartesian coordinates is

399 $$\begin{aligned} x=r\sin \theta \cos \phi ,\qquad
                                 y=r\sin \theta \sin \phi ,\qquad z=r\cos \theta
                                 \end{aligned}$$

and the inverse transformation is

400 $$\begin{aligned} r=\sqrt{x^2+y^2+z^2},\qquad
                                 \theta =\arccos \left( \frac{z}{\sqrt{x^2+y^2+z^2}}\right) ,\qquad
                                 \phi =\arctan \left( \frac{y}{x}\right) .
                                 \end{aligned}$$

Transforming the Cartesian metric to $(r,\theta ,\phi )$ coordinates, we find, as is well known,

401 $$\begin{aligned} ds^2=dr^2+r^2(d\theta ^2+\sin
                                 ^2\theta d\phi ^2), \end{aligned}$$

which is normally represented by the dyad

402 $$\begin{aligned} q^{^{_A}}=\left( 1,\frac{i}{\sin
                                 \theta }\right) , \qquad \hbox {or}\qquad q_{_{A}}=(1,i\sin \theta
                                 ). \end{aligned}$$

The components of the dyad with respect to the Cartesian coordinates are

403 $$\begin{aligned} Q^i=\left( \cos \theta \cos \phi
                                 - i\sin \phi , \cos \theta \sin \phi + i\cos \phi , -\sin \theta
                                 \right) , \end{aligned}$$

and the quantity $\Upsilon$ is

404 $$\begin{aligned} \Upsilon =- \cot \theta .
                                 \end{aligned}$$

#### Stereographic coordinates

In stereographic coordinates, the sphere is described by means of two patches, called North and South, with local coordinates $x^a_{_{[N]}}=(r,q,p)$ and $x^a_{_{[S]}}=(r,q,p)$ defined on each patch. Where it is necessary to distinguish between (*q*, *p*) on the North and South patches, we will use the suffix ${}_{_{[N]}}$ or ${}_{_{[S]}}$, but otherwise the suffix will be omitted. The relation to Cartesian coordinates is (Fig. 14)

405 $$\begin{aligned} \hbox {North:}\!\!\!\!\quad x=
                                 & {} \frac{2qr}{1+q^2+p^2},\quad
                                 y=\frac{2pr}{1+q^2+p^2},\quad z=\frac{r(1-q^2-p^2)}{1+q^2+p^2},
                                 \nonumber \\ \hbox {South:}\!\!\!\!\quad x\!= & {}
                                 \!\frac{2qr}{1+q^2+p^2},\quad y\!=\!\frac{-2pr}{1+q^2+p^2},\quad
                                 z\!=\!-\frac{r(1-q^2-p^2)}{1+q^2+p^2}.
                                 \end{aligned}$$

The inverse transformation is

406 $$\begin{aligned} \hbox {North:}\!\!\;\; r=
                                 & {} \sqrt{x^2+y^2+z^2},\quad
                                 q=\frac{x}{\sqrt{x^2+y^2+z^2}+z},\quad
                                 p=\frac{y}{\sqrt{x^2+y^2+z^2}+z}, \nonumber \\ \hbox
                                 {South:}\!\!\;\; r= & {} \sqrt{x^2+y^2+z^2},\quad
                                 q=\frac{x}{\sqrt{x^2+y^2+z^2}-z},\quad
                                 p=-\frac{y}{\sqrt{x^2+y^2+z^2}-z}.\nonumber \\
                                 \end{aligned}$$

It is then straightforward to construct the Jacobian and to transform the Cartesian metric into the metric in (*r*, *q*, *p*) coordinates. We find, on both patches,

407 $$\begin{aligned}
                                 ds^2=dr^2+\frac{4r^2}{(1+q^2+p^2)^2}\left( dq^2+dp^2\right) ,
                                 \end{aligned}$$

which is normally represented by the dyad

408 $$\begin{aligned}
                                 q^{^{_A}}=\frac{1+q^2+p^2}{2}(1,i), \qquad \hbox {or}\qquad
                                 q_{_{A}}=\frac{2}{1+q^2+p^2}(1,i).
                                 \end{aligned}$$

The relationship between $(r_{_{[N]}},q_{_{[N]}},p_{_{[N]}})$ and $(r_{_{[S]}},q_{_{[S]}},p_{_{[S]}})$ is found by going via the Cartesian coordinates. We apply Eq. (405) to find values for (*x*, *y*, *z*), and then apply Eq. (406) to find the corresponding values for $(r_{_{[S]}},q_{_{[S]}},p_{_{[S]}})$. The result is

409 $$\begin{aligned} r_{_{[S]}}=r_{_{[N]}},\qquad
                                 q_{_{[S]}}=\frac{q_{_{[N]}}}{q_{_{[N]}}^2+p_{_{[N]}}^2},\qquad
                                 p_{_{[S]}}=-\frac{p_{_{[N]}}}{q_{_{[N]}}^2+p_{_{[N]}}^2},
                                 \end{aligned}$$

which may be expressed more compactly and informatively as

410 $$\begin{aligned} q_{_{[S]}}+i
                                 p_{_{[S]}}=\frac{1}{q_{_{[N]}}+i p_{_{[N]}}}.
                                 \end{aligned}$$

The components of the dyads with respect to the Cartesian coordinates are

411 $$\begin{aligned} Q^i_{_{[N]}}=\left( \frac{1 - q^2
                                 + p^2 - 2 i q p}{1+q^2+p^2},\; \frac{i + i q^2 -i p^2 - 2 q
                                 p}{1+q^2+p^2},\; -\frac{2(q + ip)}{1+q^2+p^2}\right) ,
                                 \end{aligned}$$

412 $$\begin{aligned} Q^i_{_{[S]}}=\left( \frac{1 - q^2
                                 + p^2 - 2 i q p}{1+q^2+p^2},\; -\frac{i + i q^2 -i p^2 - 2 q
                                 p}{1+q^2+p^2}\,\; \frac{2(q + ip)}{1+q^2+p^2}\right) .
                                 \end{aligned}$$

The rotation factor between the dyads on the North and South patches, as defined in Eq. (396), is

413 $$\begin{aligned} \exp (i\gamma
                                 )=\frac{Q^i_{_{[S]}}\bar{Q}^j_{_{[N]}}\delta _{ij}}{2}
                                 =-\frac{q_{_{[N]}}-ip_{_{[N]}}}{q_{_{[N]}}+ip_{_{[N]}}},
                                 \end{aligned}$$

where Eq (410) has been used. The quantity $\Upsilon$ is

414 $$\begin{aligned} \Upsilon =q + i p.
                                 \end{aligned}$$

### The spin-weighted spherical harmonics ${}_s Y^{\ell \,m}$

The standard, i.e., spin-weight zero, spherical harmonics are given in Cartesian coordinates by

415 $$\begin{aligned}
                              Y^{\ell \, 0}=\sqrt{\frac{2\ell +1}{4\pi }}2^{-\ell } \sum
                              _{k=0}^{\left\lfloor \ell /2\right\rfloor } (-1)^k \frac{(2\ell
                              -2k)!}{k!(\ell -k)!(\ell -2k)!} \left( \frac{z}{r}\right) ^{\ell -2k},
                              \end{aligned}$$

and

416 $$\begin{aligned}
                              \left( \begin{array}{c} Y^{\ell \,m} \\ Y^{\ell \, -m} \end{array}
                              \right)= & {} \left( \begin{array}{c} (-1)^m(A_m+iB_m) \\
                              (A_m-iB_m) \end{array} \right) \sqrt{\frac{2\ell +1}{4\pi }}2^{-\ell
                              }\sqrt{\frac{(\ell -m)!}{(\ell +m)!}} \nonumber \\&\times \sum
                              _{k=0}^{\left\lfloor (\ell -m)/2\right\rfloor } \frac{(-1)^k(2\ell
                              -2k)!}{k!(\ell -k)!(\ell -2k-m)!} \frac{z^{\ell -2k-m}}{r^{\ell -2k}},
                              \end{aligned}$$

where in the above formula $m>0$, and where

417 $$\begin{aligned}
                              A_m(x,y)= & {} \sum _{k=0}^m\frac{m!}{k!(m-k)!}x^ky^{m-k}\cos
                              \left( \frac{\pi (m-k)}{2}\right) ,\nonumber \\ B_m(x,y)= & {}
                              \sum _{k=0}^m\frac{m!}{k!(m-k)!}x^ky^{m-k}\sin \left( \frac{\pi
                              (m-k)}{2}\right) . \end{aligned}$$

The symbol $$\lfloor \ \rfloor
                              $$ means truncation to an integer; for example, $$\lfloor 4/2
                              \rfloor =\lfloor 5/2 \rfloor =2$$. The (spin-weight zero) spherical harmonics in angular coordinates are then obtained by simply substituting the appropriate coordinate transformation $$x^a_{_{[C]}}=x^a_{_{[C]}}(x^a_{_{[S]}})$$ into the above formulas for $$Y^{\ell
                              \,m}$$, and it will be found that *r* cancels out of the result, leaving a formula in terms only of the angular coordinates. The constant factors in the definitions are chosen so that the $$Y^{\ell
                              \,m}$$ satisfy the orthonormality condition

418 $$\begin{aligned}
                              \int _{S^2}Y^{\ell \,m} \bar{Y}^{\ell ^\prime m^\prime }d\varOmega =
                              \delta ^{\ell \ell ^\prime } \delta ^{m m^\prime },
                              \end{aligned}$$

where integration is over the unit sphere, and where $$d\varOmega
                              =\sqrt{\det (q_{_{AB}})} d\phi ^2 d\phi ^3$$—e.g., in spherical polars $$d\varOmega =\sin
                              \theta d\theta d\phi $$.

The spin-weighted spherical harmonics are found by repeated application of the operators $$\eth
                              $$ or $$\bar{\eth
                              }$$:

419 $$\begin{aligned}&\displaystyle {}_sY^{\ell
                              \,m} = \sqrt{\frac{(\ell -s)!}{(\ell +s)!}}\eth ^s Y^{\ell \,m}, \quad
                              s>0, \nonumber \\&\displaystyle {}_{s}Y^{\ell \,m} =
                              (-1)^s \sqrt{\frac{(\ell +s)!}{(\ell -s)!}} \bar{\eth }^{-s} Y^{\ell
                              \,m}, \quad s<0. \end{aligned}$$

The $${}_sY^{\ell
                              \,m}$$ are defined only in the cases that $$|s|\le \ell
                              $$ and $$|m|\le \ell
                              $$, and in the spin-weight zero case it is usual to omit the $${}_s$$, i.e., $${}_0Y^{\ell
                              \,m}=Y^{\ell \,m}$$. The $${}_sY^{\ell
                              \,m}$$ satisfy the same orthonormality condition as the $$Y^{\ell
                              \,m}$$ in Eq. (418) and this is the origin of the square root factors in Eq. (419). However orthonormality does not fix the phase, and this raises issues that are pursued in Sect. 1. The $${}_sY^{\ell
                              \,m}$$ constitute a large number of different cases, and are obtained from computer algebra scripts, see section “Computer algebra” in Appendix 3.

The $$Y^{\ell
                              m}$$ satisfy the property

420 $$\begin{aligned}
                              \eth \bar{\eth }Y^{\ell m}=\bar{\eth }\eth Y^{\ell m}=-\varLambda
                              Y^{\ell m},\qquad \hbox {where }\varLambda =\ell (\ell + 1),
                              \end{aligned}$$

so that $$\eth \bar{\eth
                              }$$ is the Laplacian operator on the sphere. Using Eqs. (420) and (395), it follows that

421 $$\begin{aligned}
                              \eth ^2\bar{\eth }^2Y^{\ell m}=\bar{\eth }^2\eth ^2 Y^{\ell
                              m}=(\varLambda ^2-2\varLambda ) Y^{\ell m}.
                              \end{aligned}$$

### Spin-weighted representation of deviations from spherical symmetry

As already discussed, any quantity that can be regarded as a vector or tensor on the 2-sphere can be given a spin-weighted representation, and this includes quantities that describe the angular part of the metric in a curved spacetime. Suppose that a general metric has angular part $$ds^2=r^2
                              h_{_{AB}}dx^{^{_A}} \, dx^{^{_{_{B}}}}$$ (Bishop et al. 1996b); for the applications considered here we restrict attention to the case that *r* is a surface area coordinate (see Gómez et al. 1997 for the general case), so that $$\det
                              (h_{_{AB}})=\det (q_{_{AB}})$$ for some unit sphere metric $$q_{_{AB}}$$. Then we define

422 $$\begin{aligned}
                              J=\frac{1}{2} q^{^{_A}} q^{^{_{_{B}}}} h_{_{AB}}=-\frac{1}{2} q_{_{A}}
                              q_{_{B}} h^{^{_{AB}}}, \qquad \hbox { and } \qquad K=\frac{1}{2}
                              q^{^{_A}} \bar{q}^{^{_{_{B}}}} h_{_{AB}}=\frac{1}{2} q_{_{A}}
                              \bar{q}_{_{B}} h^{^{_{AB}}}, \end{aligned}$$

with the spherically symmetric case characterized by $J=0$. From the determinant condition, it follows that

423 $$\begin{aligned}
                              K^2=1+J\bar{J}. \end{aligned}$$

The metric can be written interms of *J* and *K*. We find in spherical polars

424 $$\begin{aligned}
                              h_{_{AB}}= & {} \left( \begin{array}{cc} \displaystyle
                              \frac{1}{2} (J+\bar{J}+2K) &{} \displaystyle \frac{i}{2}
                              (\bar{J}-J)\sin \theta \\ \\ \displaystyle \frac{i}{2} (\bar{J}-J)\sin
                              \theta &{} \displaystyle \frac{1}{2} (2K-J-\bar{J})\sin
                              ^2\theta \end{array} \right) ,\nonumber \\ h^{^{_{AB}}}= & {}
                              \left( \begin{array}{cc} \displaystyle \frac{1}{2} (2K-J-\bar{J})
                              &{} \displaystyle \frac{i}{2\sin \theta }(J-\bar{J}) \\ \\
                              \displaystyle \frac{i}{2\sin \theta }(J-\bar{J}) &{}
                              \displaystyle \frac{1}{2 \sin ^2\theta }( 2K+J+\bar{J}) \end{array}
                              \right) , \end{aligned}$$

and in stereographic coordinates (Bishop et al. 1997b)

425 $$\begin{aligned}
                              h_{_{AB}}= & {} \frac{2}{(1+q^2+p^2)^2}\left(
                              \begin{array}{cc} J+\bar{J}+2K &{} i(\bar{J}-J) \\ \\
                              i(\bar{J}-J) &{} 2K-J-\bar{J} \end{array} \right) ,\nonumber
                              \\ h^{^{_{AB}}}= & {} \frac{(1+q^2+p^2)^2}{8}\left(
                              \begin{array}{cc} 2K-J-\bar{J} &{} i(J-\bar{J}) \\ \\
                              i(J-\bar{J}) &{} 2K+J+\bar{J} \end{array} \right) .
                              \end{aligned}$$

The quantity *J* is simply related to the strain in planar coordinates, $$J=h_++ih_\times
                              $$. To see this, suppose that a plane with Cartesian-like coordinates (*x*, *y*) is tangent to the unit sphere at a given point $$(\theta _0,\phi
                              _0)$$ (The argument is simpler when using specific, rather than general, angular coordinates). In a neighbourhood of $$(\theta _0,\phi
                              _0)$$, the coordinate transformation is $$x=\theta -\theta
                              _0,y=(\phi -\phi _0)/\sin \theta _0$$. Now, $$h_+,h_\times
                              $$ are weak field quantities, and in this case $$|J|\ll
                              1$$. Further, $$J+\bar{J}=2\mathfrak
                              {R}(J)$$ and $$i(\bar{J}-J)=2\mathfrak
                              {I}(J)$$, and thus Eq. (424) may be simplified to

426 $$\begin{aligned}
                              h_{_{AB}}= & {} \left( \begin{array}{cc} \displaystyle
                              1+\mathfrak {R}{J} &{} \displaystyle \mathfrak {I}(J)\sin
                              \theta \\ \\ \displaystyle \mathfrak {I}(J)\sin \theta &{}
                              \displaystyle 1-\mathfrak {R}(J)\sin ^2\theta \end{array} \right) .
                              \end{aligned}$$

Then transforming from $$(\theta ,\phi
                              )$$ to (*x*, *y*) coordinates, and in a neighbourhood of $$(x,y)=(0,0)$$, gives

427 $$\begin{aligned}
                              h_{_{AB}}= & {} \left( \begin{array}{cc} \displaystyle
                              1+\mathfrak {R}{J} &{} \displaystyle \mathfrak {I}(J) \\ \\
                              \displaystyle \mathfrak {I}(J) &{} \displaystyle 1-\mathfrak
                              {R}(J) \end{array} \right) , \end{aligned}$$

which is the expected form in the TT gauge.

### $Z^{\ell \,m}$, the “real” $Y^{\ell \,m}$

Since metric quantities are real, a decomposition in terms of the $$Y^{\ell
                              \,m}$$ may introduce mode mixing between $$\pm
                              m$$ modes. This can be avoided by making use of the formalism described in Zlochower et al. (2003) and Bishop (2005), and using basis functions which, in the spin-weight 0 case, are purely real; following Bishop (2005), these are denoted as $${}_sZ^{\ell
                              \,m}$$.

428 $$\begin{aligned}
                              {}_s Z^{\ell \,m}= & {} \frac{1}{\sqrt{2}} \left[ {}_s Y^{\ell
                              \,m} +(-1)^m {}_s Y^{\ell \, -m}\right] \quad \hbox { for }
                              m>0, \nonumber \\ {}_s Z^{\ell \,m}= & {}
                              \frac{i}{\sqrt{2}} \left[ (-1)^m{}_s Y^{\ell \,m} -{}_s Y^{\ell \, -m}
                              \right] \quad \hbox { for } m<0, \nonumber \\ {}_s Z^{\ell \,
                              0}= & {} {}_s Y^{\ell \, 0}.
                              \end{aligned}$$

The $${}_sZ^{\ell
                              \,m}$$ obey orthonormal properties similar to those of the $$Y^{\ell
                              \,m}$$ in Eq. (418), and have a relationship with $$\eth ^s Z^{\ell
                              \,m}$$ similar to that for the $${}_sY^{\ell
                              \,m}$$ in Eq. (419).

### Vector and tensor spherical harmonics

Particularly within the context of gauge invariant perturbation theory, it is common practice to use vector and tensor spherical harmonics rather than spin-weighted spherical harmonics (Nagar and Rezzolla 2006). The vector spherical harmonics are defined, in the even parity case

429 $$\begin{aligned}
                              E^{\ell \,m}_{_{A}}= \nabla _{_{A}}Y^{\ell \,m},
                              \end{aligned}$$

and in the odd parity case

430 $$\begin{aligned}
                              S^{\ell \,m}_{_{C}}=\epsilon _{_{CD}} q^{^{_{DE}}} \nabla
                              _{_{E}}Y^{\ell \,m}, \end{aligned}$$

where $$\epsilon
                              _{_{CD}}$$ is the Levi-Civita completely antisymmetric tensor on the 2-sphere; for example, in spherical polars $$\epsilon _{\theta
                              \theta }=\epsilon _{\phi \phi }=0, \epsilon _{\theta \phi }=-\epsilon
                              _{\phi \theta }=\sin \theta $$. The tensor spherical harmonics are defined, in the even parity case

431 $$\begin{aligned}
                              Z^{\ell \,m}_{_{CD}}= \nabla _{_{C}}\nabla _{_{D}} Y^{\ell
                              \,m}+\frac{1}{2} \ell (\ell +1) q_{_{CD}}Y^{\ell \,m},
                              \end{aligned}$$

and in the odd parity case

432 $$\begin{aligned}
                              S^{\ell \,m}_{_{CD}}=\frac{1}{2} \left( \nabla _{_{D}} S^{\ell
                              \,m}_{_{C}} +\nabla _{_{C}}S^{\ell \,m}_{_{D}}\right) .
                              \end{aligned}$$

The vector and tensor spherical harmonics are related to the $$\eth
                              $$ operator and thereby to the spin-weighted spherical harmonics

433 $$\begin{aligned}&\displaystyle q^{^{_A}}
                              E^{\ell \,m}_{_{A}}=\eth Y^{\ell \,m}=\sqrt{(\ell +1)\ell }\,{}_1
                              Y^{\ell \,m}, \end{aligned}$$

434 $$\begin{aligned}&\displaystyle q^{^{_C}}
                              S^{\ell \,m}_{_{C}} =-i\eth Y^{\ell \,m}=-i\sqrt{\ell (\ell +1)}\,{}_1
                              Y^{\ell \,m}, \end{aligned}$$

435 $$\begin{aligned}&\displaystyle q^{^{_C}}
                              q^{^{_D}} Z^{\ell \,m}_{_{CD}}= \eth ^2 Y^{\ell \,m} = \sqrt{(\ell
                              +2)(\ell +1)\ell (\ell -1)} \,{}_2 Y^{\ell \,m},
                              \end{aligned}$$

436 $$\begin{aligned}&\displaystyle S^{\ell
                              \,m}_{_{CD}} q^{^{_C}} q^{^{_D}}=-i\eth ^2 Y^{\ell \,m} =-i\sqrt{(\ell
                              +2)(\ell +1)\ell (\ell -1)}\,{}_2 Y^{\ell \,m}.
                              \end{aligned}$$

### Regge–Wheeler harmonics

We report below the explicit expressions of the Regge–Wheeler harmonics, $$(\hat{e}_1)_{i
                              j},\ldots ,(\hat{f}_4)_{i j}$$, which have been introduced in Sect. 5.6.2 when discussing the numerical implementation of the Cauchy-perturbative method. In particular, the tensor spherical harmonics $$(\hat{e}_{1})_{ij}$$ and $$(\hat{e}_{2})_{ij}$$ in have the rather lengthy but otherwise straightforward expressions

437 $$\begin{aligned}
                              \left( \hat{e}_{1}\right) _{ij}=\left( \begin{array}{ccc} 0
                              &{} \displaystyle -\frac{1}{\sin \theta }\partial _{\phi
                              }Y_{\ell m} &{} \sin \theta \partial _{\theta }Y_{\ell m} \\
                              \\ \displaystyle -\frac{1}{\sin \theta }\partial _{\phi }Y_{\ell m}
                              &{} 0 &{} 0 \\ \\ \sin \theta \partial _{\theta
                              }Y_{\ell m} &{} 0 &{} 0 \end{array} \right) ,
                              \end{aligned}$$

and

438 $$\begin{aligned}
                              \left( \hat{e}_{2}\right) _{ij}\!=\!\left( \begin{array}{ccc} 0
                              &{} 0 &{} 0 \\ \\ 0 &{} \displaystyle
                              \frac{1}{\sin \theta } \left( \partial _{\theta \phi }^{2}-\cot \theta
                              \partial _{\phi }\right) Y_{\ell m} &{} \displaystyle
                              \frac{1}{2}\left( \displaystyle \frac{1}{\sin ^{2}\theta } \partial
                              _{\phi }^{2}-\cos \theta \partial _{\theta }- \sin \theta \partial
                              _{\theta }^{2}\right) Y_{\ell m} \\ \\ 0 &{} \displaystyle
                              \frac{1}{2}\left( \displaystyle \frac{1}{\sin ^{2}\theta }\partial
                              _{\phi }^{2} - \cos \theta \partial _{\theta }-\sin \theta \partial
                              _{\theta }^{2} \right) Y_{\ell m} &{} -\left( \sin \theta
                              \partial _{\theta \phi }^{2}- \cos \theta \partial _{\phi }\right)
                              Y_{\ell m} \end{array} \right) .
                              \end{aligned}$$

Similarly, the tensor spherical harmonics $$(\hat{f}_{1})_{ij}
                              - (\hat{f}_{4})_{ij}$$ which enter in the decomposition of even-parity perturbations have the form

439 $$\begin{aligned}
                              \left( \hat{f}_{1}\right) _{ij}= & {} \left(
                              \begin{array}{ccc} 0 &{} \partial _{\theta }Y_{\ell m}
                              &{} \partial _{\phi }Y_{\ell m} \\ \\ \partial _{\theta
                              }Y_{\ell m} &{} 0 &{} 0 \\ \\ \partial _{\phi }Y_{\ell
                              m} &{} 0 &{} 0 \end{array} \right) ,
                              \end{aligned}$$

440 $$\begin{aligned}
                              \left( \hat{f}_{2}\right) _{ij}= & {} \left(
                              \begin{array}{ccc} Y_{\ell m} &{}\quad 0 &{}\quad 0 \\
                              \\ 0 &{}\quad 0 &{}\quad 0 \\ \\ 0 &{}\quad 0
                              &{}\quad 0 \end{array} \right) ,
                              \end{aligned}$$

441 $$\begin{aligned}
                              \left( \hat{f}_{3}\right) _{ij}= & {} \left(
                              \begin{array}{ccc} 0 &{} 0 &{} 0 \\ \\ 0 &{}
                              Y_{\ell m} &{} 0 \\ \\ 0 &{} 0 &{} \sin
                              ^{2}\theta Y_{\ell m} \end{array} \right) ,
                              \end{aligned}$$

and

442 $$\begin{aligned}
                              \left( \hat{f}_{4}\right) _{ij}=\left( \begin{array}{ccc} 0
                              &{} 0 &{} 0 \\ \\ 0 &{} \partial _{\theta
                              }^{2}Y_{\ell m} &{} \left( \partial _{\theta \phi }^{2}-\cot
                              \theta \partial _{\phi }\right) Y_{\ell m} \\ \\ 0 &{} \left(
                              \partial _{\theta \phi }^{2}-\cot \theta \partial _{\phi }\right)
                              Y_{\ell m} &{} \left( \partial _{\phi }^{2} -\sin \theta \cos
                              \theta \partial _{\theta }\right) Y_{\ell m} \end{array} \right) .
                              \end{aligned}$$

### Issues of convention in the definitions of spin-weighted quantities

The reader needs to be aware that different authors use different conventions in the definitions of quantities discussed in this section, and that it is therefore inadvisable to use expressions from different sources without first carefully checking the conventions used.

Fortunately, the definitions of the $$Y^{\ell
                              \,m}$$ do seem to be standard throughout the mathematical-physics community. However, there are differences in the definition of the complex dyad, here denoted by $$q^{^{_A}}$$ and normalized so that $$q^{^{_A}}
                              \bar{q}_{_{A}}=2$$; much other work uses $$m^{^{_A}}$$ with normalization $$m^{^{_A}}
                              \bar{m}_{_{A}}=1$$ so that $$q^{^{_A}}=\sqrt{2}m^{^{_A}}$$. Clearly the dyad definition—$$q^{^{_A}}$$ or $$m^{^{_A}}$$—affects the definition of spin-weighted quantities. The $$\eth
                              $$ operator is defined so that for a spin-weight 0 scalar *V*, $$\eth \bar{\eth
                              }V=\nabla ^2 V$$ where $$\nabla
                              ^2$$ is the Laplacian operator on the unit sphere, and the various definitions of $$\eth
                              $$ all have the same magnitude. However, there can be a variation in sign. For example, the definition used in Alcubierre (2008) is $$-1$$ times that used here. This implies that the definition of $${}_sY^{\ell
                              \,m}$$ is $$-1$$ times that used here for any *odd* *s*, positive or negative. However, for *even* *s* definitions of $${}_sY^{\ell
                              \,m}$$ are consistent.

The definitions of the *even* vector and tensor spherical harmonics seem to be consistent, although the notation can vary. However, there are sign differences in the definition of the *odd* vector and tensor spherical harmonics, for example Alcubierre (2008) and Baumgarte and Shapiro (2010).

## Appendix 3: Computer codes and scripts

### Computer algebra

This appendix describes the computer algebra (Maple) files used to derive a number of equations in the main text. The files are available in the online version at doi:10.1007/s41114-016-0001-9. The maple script files are named name.map with output in name.out. In some cases the main script files call auxilliary scripts as detailed below.

The files gamma.map, gamma.out, R.map and contraint.map are used to derive the vacuum nonlinear Einstein equations for the Bondi–Sachs metric in Sect. 6.1. The linearized Einstein equations were given as Eqs. (253) to (258). The file gamma.map calculates the Christoffel symbols, and then the script R.map reads gamma.out and calculates the hypersurface and evolution equations, confirming the formulas given in Bishop et al. (1997b). The files J.map, k.map, U.map and W.map are auxilliary scripts used by R.map. The script constraint.map uses gamma.out and k.map, and evaluates $$R_{00},R_{01}$$ and $$q^AR_{0A}$$. The asymptotic Einstein Eqs. (263) to (266), as well as the condition $$\partial
                              _{\tilde{\rho }}\tilde{W}_c=0$$ in Eq. (269), are derived in the script asympt.map, and using the auxilliary files gamma-asympt.map, gamma-asympt.out.

The script C_trans.map uses compactified.map and derives Eqs. (280) to (285) and (290). The script J_om_Jrho_delta.map uses compactified.map and derives Eqs. (295), (301) and (336). The script JK.map also uses compactified.map and derives Eqs. (287) and (289), and checks that $$|\nu
                              |=1$$ in Eq. (294) and that $$\tilde{m}_0^\alpha
                              \tilde{m}_\alpha = 0$$. The script NewsBondi.map uses conformal.map and evaluates the news $${{\mathcal
                              {N}}}$$ in the Bondi gauge, confirming that Eq. (313) reduces to $$\partial
                              _{\tilde{u}}\partial _{\tilde{\rho
                              }}\tilde{J}/2$$ independently of whether $$\hat{\tilde{m}}^\alpha
                              _{(0)}$$ or $$\hat{\tilde{m}}^\alpha $$ is used in Eq. (313). The script checkFA_FB_GamAB1_0.map uses gamma-asympt.map and shows that $$F^{^{_A}}F^{^{_{_{B}}}} \hat{\varGamma
                              }^1_{_{AB}(0)}=0$$, as discussed just after Eq. (321). The script NewsGen.map uses conformal.map for the further evaluation of Eq. (321) to obtain an expression for $${{\mathcal
                              {N}}}$$ in the general case. The script psi4Bondi.map uses weyl_asympt.map to evaluate Eq. (322) confirming that, in the Bondi gauge, $$\psi ^0_4=\partial
                              ^2_{\tilde{u}}\partial _{\tilde{\rho
                              }}\bar{\tilde{J}}$$, independently of whether $$\hat{\tilde{m}}^\alpha
                              _{(0)}$$ or $$\hat{\tilde{m}}^\alpha $$ is used. The script psi4Gen.map again uses weyl_asympt.map to reduce, in the general gauge, Eq. (323) to computational $$\eth
                              $$ form. In the linearized case, NewsLin.map uses news.map and conformal.map to derive Eq. (330) for $${{\mathcal
                              {N}}}$$, and psi4_lin.map uses weyl_asympt.map to derive Eq. (333) for $$\psi
                              ^0_4$$.

The files polars.map and stereo.map are used in Appendix 2. They specify the coordinate transformation between spherical and Cartesian coordinates, as well as the metric, dyad and $$\Upsilon
                              $$ (used in the evaluation of the operator $$\eth
                              $$) in the spherical coordinates. Each file passes these quantities to the procedure “C2P” in the file procs.map, which checks that all the relations given in Eqs. (384) and (391) are satisfied; this procedure also calculates and outputs the dyad transformed into Cartesian coordinates, i.e., $$Q^a$$. The procedure “sYlm” in procs.map requires as input the various quantities defined in the coordinate-specific files, together with values for $$s,\ell
                              $$ and *m*; it then calculates $${}_sY^{\ell
                              \,m}$$ in the appropriate coordinate patch using the equations given in Appendix 2. The file sYlm.map reads each of the driver files in turn, and for each coordinate patch calculates all the $${}_sY^{\ell
                              \,m}$$ for $$\ell \le \ell
                              _{\mathrm{max}}$$, $$|s|\le \min (\ell
                              _{\mathrm{max}},s_{\mathrm{max}})$$, with default values $$\ell
                              _{\mathrm{max}}=3,s_{\mathrm{max}}=2$$. The output is written to polars-sYlm.out, stereoNorth-sYlm.out and stereoSouth-sYlm.out.

### Numerical codes

The Einstein toolkit (http://einsteintoolkit.org) contains a wide range of numerical relativity codes using the Cactus code framework (http://cactuscode.org). In particular, it contains the following thorns relevant to gravitational-wave extraction: “Extract” which implements the Cauchy-perturbative method, “WeylScal4” which implements $$\psi
                              _4$$ extraction. The process of characteristic extraction is started in “NullSHRExtract” which constructs the worldtube boundary data, and then the various thorns listed under “PITTNullCode” are used for the characteristic evolution and the determination of gravitational-wave descriptors at $$\mathcal
                              {J}^+$$.

## References

[CR1] Abbott BP (2016). Observation of gravitational waves from a binary black hole
merger. Phys Rev Lett.

[CR2] Abrahams A, Cook G (1994). Collisions of boosted black holes: perturbation theory prediction of
gravitational radiation. Phys Rev D.

[CR3] Abrahams A, Evans C (1988). Reading off gravitational radiation waveforms in numerical relativity
calculations: matching to linearised gravity. Phys Rev D.

[CR4] Abrahams A, Evans C (1990). Gauge invariant treatment of gravitational radiation near the source:
analysis and numerical simulations. Phys Rev D.

[CR5] Abrahams A, Price RH (1996a) Black-hole collisions from Brill–Lindquist initial data: predictions of perturbation theory. Phys Rev D 53:1972–1976. doi:10.1103/PhysRevD.53.1972 10.1103/physrevd.53.1972 10020189

[CR6] Abrahams AM, Price RH (1996b) Applying black hole perturbation theory to numerically generated spacetimes. Phys Rev D 53:1963. doi:10.1103/PhysRevD.53.1963 10.1103/physrevd.53.1963 10020188

[CR7] Abrahams A, Bernstein D, Hobill D, Seidel E, Smarr LL (1992). Numerically generated black hole spacetimes: interaction with
gravitational waves. Phys Rev D.

[CR8] Abrahams AM, Shapiro SL, Teukolsky SA (1995). Calculation of gravitational wave forms from black hole collisions and
disk collapse: applying perturbation theory to numerical
space-times. Phys Rev D.

[CR9] Abrahams AM, Rezzolla L, Rupright ME (1998). Gravitational wave extraction and outer boundary conditions by
perturbative matching. Phys Rev Lett.

[CR10] Adamo TM, Newman ET, Kozameh CN (2012) Null geodesic congruences, asymptotically-flat spacetimes and their physical interpretation. Living Rev Relativ 15:lrr-2012-1. doi:10.12942/lrr-2012-1. http://www.livingreviews.org/lrr-2012-1, arXiv:0906.2155 10.12942/lrr-2012-1 PMC5660887 29142499

[CR11] Alcubierre M (2008). Introduction to 3+1 numerical relativity, international series of monographs
on physics.

[CR12] Alic D, Bona-Casas C, Bona C, Rezzolla L, Palenzuela C (2012). Conformal and covariant formulation of the Z4 system with
constraint-violation damping. Phys Rev D.

[CR13] Alic D, Kastaun W, Rezzolla L (2013). Constraint damping of the conformal and covariant formulation of the
Z4 system in simulations of binary neutron stars. Phys Rev D.

[CR14] Allen G, Camarda K, Seidel E (1998) 3D black hole spectroscopy: determining waveforms from 3D excited black holes. ArXiv e-prints arXiv:gr-qc/9806036

[CR15] Allen G, Goodale T, Seidel E (1999) The cactus computational collaboratory: Enabling technologies for relativistic astrophysics, and a toolkit for solving pdes by communities in science and engineering. In: The seventh symposium on the frontiers of massively parallel computation (frontiers’99). IEEE, Los Alamitos, pp 36–41

[CR16] Andrade Z, Price RH (1999). Excitation of the odd parity quasinormal modes of compact
objects. Phys Rev D.

[CR17] Anninos P, Hobill D, Seidel E, Smarr LL, Suen WM (1993). The collision of two black holes. Phys Rev Lett.

[CR18] Anninos P, Hobill D, Seidel E, Smarr LL, Suen WM (1995). The head-on collision of two equal mass black holes. Phys Rev D.

[CR19] Anninos P, Price RH, Pullin J, Seidel E, Suen WM (1995). Head-on collision of two black holes: comparison of different
approaches. Phys Rev D.

[CR20] Arnowitt R, Deser S, Misner CW (2008). Republication of: the dynamics of general relativity. Gen Relativ Gravit.

[CR21] Aylott B (2009). Testing gravitational-wave searches with numerical relativity
waveforms: results from the first Numerical INJection Analysis (NINJA)
project. Class Quantum Gravity.

[CR22] Babiuc MC, Szilágyi B, Hawke I, Zlochower Y (2005). Gravitational wave extraction based on Cauchy-characteristic
extraction and characteristic evolution. Class Quantum Gravity.

[CR23] Babiuc MC, Bishop NT, Szilágyi B, Winicour J (2009). Strategies for the characteristic extraction of gravitational
waveforms. Phys Rev D.

[CR24] Babiuc MC, Szilágyi B, Winicour J, Zlochower Y (2011a) Characteristic extraction tool for gravitational waveforms. Phys Rev D 84:044057. doi:10.1103/PhysRevD.84.044057. arXiv:1011.4223

[CR25] Babiuc MC, Winicour J, Zlochower Y (2011b) Binary black hole waveform extraction at null infinity. Class Quantum Gravity 28:134006. doi:10.1088/0264-9381/28/13/134006. arXiv:1106.4841

[CR26] Babiuc MC, Kreiss HO, Winicour J (2014). Testing the well-posedness of characteristic evolution of scalar
waves. Class Quantum Gravity.

[CR27] Baiotti L, Hawke I, Rezzolla L, Schnetter E (2005). Gravitational-wave emission from rotating gravitational collapse in
three dimensions. Phys Rev Lett.

[CR28] Baiotti L, De Pietri R, Manca GM, Rezzolla L (2007). Accurate simulations of the dynamical bar-mode instability in full
general relativity. Phys Rev D.

[CR29] Baiotti L, Bernuzzi S, Corvino G, De Pietri R, Nagar A (2009). Gravitational-wave extraction from neutron stars oscillations:
comparing linear and nonlinear techniques. Phys Rev D.

[CR30] Baker J, Campanelli M (2000). Making use of geometrical invariants in black hole
collisions. Phys Rev D.

[CR31] Baker J, Brandt SR, Campanelli M, Lousto CO, Seidel E, Takahashi R (2000). Nonlinear and perturbative evolution of distorted black holes:
odd-parity modes. Phys Rev D.

[CR32] Baker J, Brügmann B, Campanelli M, Lousto CO (2000). Gravitational waves from black hole collisions via an eclectic
approach. Class Quantum Gravity.

[CR33] Baker J, Brügmann B, Campanelli M, Lousto CO, Takahashi R (2001). Plunge waveforms from inspiralling binary black holes. Phys Rev Lett.

[CR34] Baker J, Campanelli M, Lousto CO (2002). The Lazarus project: a pragmatic approach to binary black hole
evolutions. Phys Rev D.

[CR35] Baker J, Campanelli M, Lousto CO, Takahashi R (2002). Modeling gravitational radiation from coalescing binary black
holes. Phys Rev D.

[CR36] Baker JG, Centrella J, Choi DI, Koppitz M, van Meter J (2006). Gravitational wave extraction from an inspiraling configuration of
merging black holes. Phys Rev Lett.

[CR37] Balakrishna J, Bondarescu R, Daues G, Siddhartha Guzman F, Seidel E (2006). Evolution of 3d boson stars with waveform extraction. Class Quantum Gravity.

[CR38] Bardeen JM, Press WH (1973). Radiation fields in the Schwarzschild background. J Math Phys.

[CR39] Bartnik R (1997). Einstein equations in the null quasispherical gauge. Class Quantum Gravity.

[CR40] Bartnik R, Norton AH (2000). Numerical methods for the Einstein equations in null quasi-spherical
coordinates. SIAM J Sci Comput.

[CR41] Baumgarte TW, Shapiro SL (1999). Numerical integration of Einstein’s field
equations. Phys Rev D.

[CR42] Baumgarte TW, Shapiro SL (2010). Numerical relativity: solving Einstein’s equations on the
computer.

[CR43] Beetle C, Bruni M, Burko LM, Nerozzi A (2005). Towards wave extraction in numerical relativity: foundations and
initial-value formulation. Phys Rev D.

[CR44] Bernuzzi S, Hilditch D (2010). Constraint violation in free evolution schemes: comparing bssnok with
a conformal decomposition of z4. Phys Rev D.

[CR45] Bishop NT, d’Inverno RA (1992). Some aspects of the characteristic initial value problem in numerical
relativity. Approaches to numerical relativity.

[CR46] Bishop NT (1993). Numerical relativity: combining the Cauchy and characteristic initial
value problem. Class Quantum Gravity.

[CR47] Bishop NT (2005). Linearized solutions of the Einstein equations within a
Bondi–Sachs framework, and implications for boundary conditions in
numerical simulations. Class Quantum Gravity.

[CR48] Bishop N, Deshingkar S (2003). New approach to calculating the news. Phys Rev D.

[CR49] Bishop NT, Reisswig C (2014). The gravitational wave strain in the characteristic formalism of
numerical relativity. Gen Rel Gravit.

[CR50] Bishop NT, Clarke C, d’Inverno R (1990). Numerical relativity on a transputer array. Class Quantum Gravity.

[CR51] Bishop NT, Gómez R, Holvorcem PR, Matzner RA, Papadopoulos P, Winicour J (1996). Cauchy-characteristic matching: a new approach to radiation boundary
conditions. Phys Rev Lett.

[CR52] Bishop NT, Gómez R, Lehner L, Winicour J (1996). Cauchy-characteristic extraction in numerical
relativity. Phys Rev D.

[CR53] Bishop NT, Gómez R, Holvorcem PR, Matzner RA, Papadopoulos P, Winicour J (1997a) Cauchy-characteristic evolution and waveforms. J Comput Phys 136:140–167. doi:10.1006/jcph.1997.5754

[CR54] Bishop NT, Gómez R, Lehner L, Maharaj M, Winicour J (1997b) High-powered gravitational news. Phys Rev D 56:6298–6309. doi:10.1103/PhysRevD.56.6298. arXiv:gr-qc/9708065

[CR55] Bishop NT, Gómez R, Isaacson RA, Lehner L, Szilágyi B, Winicour J, Bhawal B, Iyer BR (1999). Cauchy-characteristic matching. Black holes, gravitational radiation and the universe: essays in honour of
c.v. Vishveshwara, fundamental theories of physics.

[CR56] Bishop NT, Gómez R, Lehner L, Maharaj M, Winicour J (1999). The incorporation of matter into characteristic numerical
relativity. Phys Rev D.

[CR57] Bishop NT, Gómez R, Lehner L, Maharaj M, Winicour J (2005). Characteristic initial data for a star orbiting a black
hole. Phys Rev D.

[CR58] Bishop NT, Pollney D, Reisswig C (2011). Initial data transients in binary black hole
evolutions. Class Quantum Gravity.

[CR59] Blanchet L (2014) Gravitational radiation from post-Newtonian sources and inspiralling compact binaries. Living Rev Relativ 17:lrr-2014-2. doi:10.12942/lrr-2014-2. http://www.livingreviews.org/lrr-2014-2, arXiv:1310.1528 10.12942/lrr-2014-2 PMC5256563 28179846

[CR60] Blanchet L, Damour T, Schäfer G (1990). Post-Newtonian hydrodynamics and post-Newtonian gravitational wave
generation for numerical relativity. Mon Not R Astron Soc.

[CR61] Bona C, Palenzuela-Luque C (2009) Elements of numerical relativity and relativistic hydrodynamics: from Einstein’s equations to black hole simulations, vol 673, lecture notes in physics. Springer, Berlin. doi:10.1007/b135928

[CR62] Bona C, Ledvinka T, Palenzuela C, Žáček M (2003). General-covariant evolution formalism for numerical
relativity. Phys Rev D.

[CR63] Bona C, Ledvinka T, Palenzuela C, Žáček M (2004). Symmetry-breaking mechanism for the Z4 general-covariant evolution
system. Phys Rev D.

[CR64] Bona C, Palenzuela-Luque C, Bona-Casas C (2009) Elements of numerical relativity and relativistic hydrodynamics: from Einstein’s equations to astrophysical simulations, vol 783, 2nd edn, lecture notes in physics. Springer, Berlin

[CR65] Bondi H (1960). Gravitational waves in general relativity. Nature.

[CR66] Bondi H, van der Burg MGJ, Metzner AWK (1962). Gravitational waves in general relativity VII. Waves from
axi-symmetric isolated systems. Proc R Soc Lond Ser A.

[CR67] Boyle M (2016) Transformations of asymptotic gravitational-wave data. Phys Rev D 93:084031. doi:10.1103/PhysRevD.93.084031. arXiv:1509.00862

[CR68] Boyle M, Mroué AH (2009). Extrapolating gravitational-wave data from numerical
simulations. Phys Rev D.

[CR69] Cactus (2016) The Cactus code. URL http://www.cactuscode.org/, project homepage

[CR70] Camarda K, Seidel E (1999). Three-dimensional simulations of distorted black holes: comparison
with axisymmetric results. Phys Rev D.

[CR71] Campanelli M, Lousto CO (1998). The imposition of Cauchy data to the Teukolsky equation I: the
nonrotating case. Phys Rev D.

[CR72] Campanelli M, Lousto CO (1999). Second order gauge invariant gravitational perturbations of a Kerr
black hole. Phys Rev D.

[CR73] Campanelli M, Krivan W, Lousto CO (1998). The imposition of Cauchy data to the Teukolsky equation II: numerical
comparison with the Zerilli–Moncrief approach to black hole
perturbations. Phys Rev D.

[CR74] Campanelli M, Lousto CO, Baker J, Khanna G, Pullin J (2000). The imposition of Cauchy data to the Teukolsky equation III: the
rotating case. Phys Rev D.

[CR75] Campanelli M, Kelly BJ, Lousto CO (2006). The Lazarus project II: space-like extraction with the
quasi-Kinnersley tetrad. Phys Rev D.

[CR76] Campanelli M, Lousto CO, Nakano H, Zlochower Y (2009). Comparison of numerical and post-Newtonian waveforms for generic
precessing black-hole binaries. Phys Rev D.

[CR77] Cerdá-Durán P, Faye G, Dimmelmeier H, Font JA, Ibáñez JM, Müller E, Schäfer G (2005). CFC+: improved dynamics and gravitational waveforms from relativistic
core collapse simulations. Astron Astrophys.

[CR78] Chandrasekhar S (1978). The gravitational perturbations of the kerr black hole. I. The
perturbations in the quantities which vanish in the stationary
state. Proc R Soc Lond.

[CR79] Chandrasekhar S (1983). The mathematical theory of black holes, the international series of
monographs on physics.

[CR80] Chandrasekhar S, Detweiler S (1975). The quasi-normal modes of the Schwarzschild black hole. Proc R Soc Lond.

[CR81] Clarke CJS, d’Inverno RA (1994). Combining Cauchy and characteristic numerical evolutions in curved
coordinates. Class Quantum Gravity.

[CR82] Clarke CJS, d’Inverno RA, Vickers JA (1995). Combining Cauchy and characteristic codes. i. The vacuum cylindrically
symmetric problem. Phys Rev D.

[CR83] Cook GB, Huq MF, Klasky SA (1998). Boosted three-dimensional black-hole evolutions with singularity
excision. Phys Rev Lett.

[CR84] Corkill RW, Stewart JM (1983). Numerical relativity. II. Numerical methods for the characteristic
initial value problem and the evolution of the vacuum field equations for
space-times with two killing vectors. Proc R Soc Lond Ser A.

[CR85] Corvino G, Rezzolla L, Bernuzzi S, De Pietri R, Giacomazzo B (2010). On the shear instability in relativistic neutron stars. Class Quantum Gravity.

[CR86] Cunningham CT, Price RH, Moncrief V (1978). Radiation from collapsing relativistic stars I. Linearized odd-parity
radiation. Astrophys J.

[CR87] Cunningham CT, Price RH, Moncrief V (1979). Radiation from collapsing relativistic stars II. Linearized
even-parity radiation. Astrophys J.

[CR88] d’Inverno RA, Vickers JA (1996). Combining Cauchy and characteristic codes. III. The interface problem
in axial symmetry. Phys Rev D.

[CR89] d’Inverno RA, Vickers JA (1997). Combining Cauchy and characteristic codes. IV. The characteristic
field equations in axial symmetry. Phys Rev D.

[CR90] d’Inverno RA, Dubal MR, Sarkies EA (2000). Cauchy-characteristic matching for a family of cylindrical solutions
possessing both gravitational degrees of freedom. Class Quantum Gravity.

[CR91] Damour T, Gopakumar A (2006). Gravitational recoil during binary black hole coalescence using the
effective one body approach. Phys Rev D.

[CR92] Damour T, Nagar A (2016) Astrophysical black holes. In: Haardt F, Gorini V, Moschella U, Treves A, Colpi M (eds) The effective-one-body approach to the general relativistic two body problem, vol 905, lecture notes in physics. Springer, Berlin, pp 273–312. doi:10.1007/978-3-319-19416-5_7

[CR93] de Felice F, Clarke CJS (1990). Relativity on curved manifolds. Cambridge monographs on mathematical
physics.

[CR94] de Oliveira HP, Rodrigues EL (2009). A dynamical system approach for the Bondi problem. Int J Mod Phys A.

[CR95] Dimmelmeier H, Ott CD, Janka H, Marek A, Müller E (2007) Generic gravitational-wave signals from the collapse of rotating stellar cores. Phys Rev Lett 98:251101. doi:10.1103/PhysRevLett.98.251101. arXiv:astro-ph/0702305 10.1103/PhysRevLett.98.251101 17678008

[CR96] Dubal MR, d’Inverno RA, Clarke CJS (1995). Combining Cauchy and characteristic codes. II. The interface problem
for vacuum cylindrical symmetry. Phys Rev D.

[CR97] Dubal MR, d’Inverno RA, Vickers JA (1998). Combining Cauchy and characteristic codes. V. Cauchy-characteristic
matching for a spherical spacetime containing a perfect fluid. Phys Rev D.

[CR98] Einstein A (1916). Näherungsweise integration der feldgleichungen der
gravitation. Sitzungsber K Preuss Akad Wiss and Phys-Math Kl.

[CR99] Einstein A (1918). Über gravitationswellen. Sitzungsber K Preuss Akad Wiss.

[CR100] Favata M, Hughes SA, Holz DE (2004). How black holes get their kicks: gravitational radiation recoil
revisited. Astrophys J.

[CR101] Ferrari V, Kokkotas KD (2000). Scattering of particles by neutron stars: time evolutions for axial
perturbations. Phys Rev D.

[CR102] Ferrari V, Gualtieri L, Rezzolla L (2006). A hybrid approach to black hole perturbations from extended matter
sources. Phys Rev D.

[CR103] Finn LS, Evans CR (1990). Determining gravitational radiation from Newtonian self-gravitating
systems. Astrophys J.

[CR104] Fiske DR, Baker JG, van Meter JR, Choi DI, Centrella JM (2005). Wave zone extraction of gravitational radiation in three-dimensional
numerical relativity. Phys Rev D.

[CR105] Font JA, Goodale T, Iyer S, Miller M, Rezzolla L, Seidel E, Stergioulas N, Suen WM, Tobias M (2002). Three-dimensional numerical general relativistic hydrodynamics. II.
Long-term dynamics of single relativistic stars. Phys Rev D.

[CR106] Frauendiener J (2004) Conformal infinity. Living Rev Relativ 7:lrr-2004-1. doi:10.12942/lrr-2004-1. http://www.livingreviews.org/lrr-2004-1 10.12942/lrr-2004-1 PMC5256109 28179863

[CR107] Frittelli S (1997). Note on the propagation of the constraints in standard 3+1 general
relativity. Phys Rev D.

[CR108] Frittelli S, Gómez R (2000). Ill-posedness in the Einstein equations. J Math Phys.

[CR109] Gerlach UH, Sengupta UK (1979). Even parity junction conditions for perturbations on most general
spherically symmetric space-times. J Math Phys.

[CR110] Gerlach UH, Sengupta UK (1979). Gauge-invariant perturbations on most general spherically symmetric
space-times. Phys Rev D.

[CR111] Gerlach UH, Sengupta UK (1980). Gauge-invariant coupled gravitational, acoustical, and electromagnetic
modes on most general spherical space-times. Phys Rev D.

[CR112] Geroch R, Esposito FP, Witten L (1977). Asymptotic structure of space-time. Asymptotic structure of spacetime.

[CR113] Geroch R, Winicour J (1981). Linkages in general relativity. J Math Phys.

[CR114] Goldberg JN, MacFarlane AJ, Newman ET, Rohrlich F, Sudarshan ECG (1967). Spin-$$s$$ spherical harmonics and $$\eth
                           $$. J Math Phys.

[CR115] Gómez R (2001). Gravitational waveforms with controlled accuracy. Phys Rev D.

[CR116] Gómez R, Frittelli S (2003). First-order quasilinear canonical representation of the characteristic
formulation of the einstein equations. Phys Rev D.

[CR117] Gómez R, Papadopoulos P, Winicour J (1994). Null cone evolution of axisymmetric vacuum spacetimes. J Math Phys.

[CR118] Gómez R, Laguna P, Papadopoulos P, Winicour J (1996). Cauchy-characteristic evolution of
Einstein–Klein–Gordon systems. Phys Rev D.

[CR119] Gómez R, Lehner L, Papadopoulos P, Winicour J (1997). The eth formalism in numerical relativity. Class Quantum Gravity.

[CR120] Gourgoulhon E (2012) 3+1 Formalism in general relativity: bases of numerical relativity, vol 846, lecture notes in physics. Springer, Berlin. doi:10.1007/978-3-642-24525-1. arXiv:gr-qc/0703035

[CR121] Gundlach C, Martín-García JM (2000). Gauge-invariant and coordinate-independent perturbations of stellar
collapse I: the interior. Phys Rev D.

[CR122] Gundlach C, Martín-García JM (2001). Gauge-invariant and coordinate-independent perturbations of stellar
collapse II: matching to the exterior. Phys Rev D.

[CR123] Gunnarsen L, Shinkai H, Maeda K (1995). A ‘3+1’ method for finding principal null
directions. Class Quantum Gravity.

[CR124] Handmer CJ, Szilágyi B (2015). Spectral characteristic evolution: a new algorithm for gravitational
wave propagation. Classical and Quantum Gravity.

[CR125] Handmer CJ, Szilagyi B, Winicour J (2015). Gauge invariant spectral Cauchy characteristic
extraction. Class Quantum Gravity.

[CR126] Handmer CJ, Szilágyi B, Winicour J (2016) Spectral Cauchy characteristic extraction of strain, news and gravitational radiation flux. ArXiv e-prints ArXiv:1605.04332

[CR127] Harada T, Iguchi H, Shibata M (2003) Computing gravitational waves from slightly nonspherical stellar collapse to black hole: odd-parity perturbation. Phys Rev D 68:024002. doi:10.1103/PhysRevD.68.024002. arXiv:gr-qc/0305058

[CR128] Helfer AD (2010). Estimating energy-momentum and angular momentum near null
infinity. Phys Rev D.

[CR129] Henry RC (2000). Kretschmann scalar for a Kerr–Newman black
hole. Astrophys J.

[CR130] Hinder I, Wardell B, Bentivegna E (2011). Falloff of the Weyl scalars in binary black hole
spacetimes. Phys Rev D.

[CR131] Isaacson R (1968). Gravitational radiation in the limit of high frequency. II. Nonlinear
terms and the effective stress tensor. Phys Rev.

[CR132] Isaacson R, Welling J, Winicour J (1983). Null cone computation of gravitational radiation. J Math Phys.

[CR133] Ishibashi A, Kodama H (2003). Stability of higher-dimensional Schwarzschild black
holes. Prog Theor Phys.

[CR134] Karlovini M (2002). Axial perturbations of general spherically symmetric
spacetimes. Class Quantum Gravity.

[CR135] Kawamura M, Oohara K (2004). Gauge-invariant gravitational wave extraction from coalescing binary
neutron stars. Prog Theor Phys.

[CR136] Kawamura M, Oohara Ki, Nakamura T (2003) General relativistic numerical simulation on coalescing binary neutron stars and gauge-invariant gravitational wave extraction. ArXiv e-prints arXiv:astro-ph/0306481

[CR137] Kinnersley W (1969). Type d vacuum metrics. J Math Phys.

[CR138] Kodama H, Ishibashi A (2003). A master equation for gravitational perturbations of maximally
symmetric black holes in higher dimensions. Prog Theor Phys.

[CR139] Kodama H, Ishibashi A (2004). Master equations for perturbations of generalized static black holes
with charge in higher dimensions. Prog Theor Phys.

[CR140] Kodama H, Ishibashi A, Seto O (2000). Brane world cosmology: gauge-invariant formalism for
perturbation. Phys Rev D.

[CR141] Kreiss HO, Winicour J (2011). The well-posedness of the null-timelike boundary problem for
quasilinear waves. Class Quantum Gravity.

[CR142] Landau LD, Lifshitz EM (1975). The classical theory of fields, course of theoretical physics.

[CR143] Lehner L (1998) Gravitational radiation from black hole spacetimes. PhD thesis, University of Pittsburgh, Pittsburgh

[CR144] Lehner L (1999). A dissipative algorithm for wave-like equations in the characteristic
formulation. J Comput Phys.

[CR145] Lehner L (2000). Matching characteristic codes: exploiting two
directions. Int J Mod Phys D.

[CR146] Lehner L (2001). Numerical relativity: a review. Class Quantum Gravity.

[CR147] Lousto CO, Price RH (1997). Headon collisions of black holes: the particle limit. Phys Rev D.

[CR148] Lousto CO, Zlochower Y (2007). A practical formula for the radiated angular momentum. Phys Rev D.

[CR149] Lousto CO, Nakano H, Zlochower Y, Campanelli M (2010). Intermediate-mass-ratio black hole binaries: intertwining numerical
and perturbative techniques. Phys Rev D.

[CR150] Martel K (2004). Gravitational waveforms from a point particle orbiting a Schwarzschild
black hole. Phys Rev D.

[CR151] Martel K, Poisson E (2002). A one-parameter family of time-symmetric initial data for the radial
infall of a particle into a Schwarzschild black hole. Phys Rev D.

[CR152] Martel K, Poisson E (2005). Gravitational perturbations of the Schwarzschild spacetime: a
practical covariant and gauge-invariant formalism. Phys Rev D.

[CR153] Martín-García JM, Gundlach C (1999). All nonspherical perturbations of the choptuik spacetime
decay. Phys Rev D.

[CR154] Mathews J (1962). Gravitational multipole radiation. J Soc Ind Appl Math.

[CR155] McKechan DJA, Robinson C, Sathyaprakash BS (2010). A tapering window for time-domain templates and simulated signals in
the detection of gravitational waves from coalescing compact
binaries. Class Quantum Gravity.

[CR156] Misner CW, Thorne KS, Wheeler JA (1973). Gravitation.

[CR157] Moncrief V (1974). Gravitational perturbations of spherically symmetric systems. I. The
exterior problem. Ann Phys.

[CR158] Nagar A, Rezzolla L (2006). Gauge-invariant non-spherical metric perturbations of Schwarzschild
black-hole spacetimes. Class Quantum Gravity.

[CR159] Nagar A, Díaz G, Pons JA, Font JA (2004). Accretion-driven gravitational radiation from nonrotating compact
objects: infalling quadrupolar shells. Phys Rev D.

[CR160] Nagar A, Font JA, Zanotti O, de Pietri R (2005). Gravitational waves from oscillating accretion tori: comparison
between different approaches. Phys Rev D.

[CR161] Nakamura T, Oohara K, Kojima Y (1987). General relativistic collapse to black holes and gravitational waves
from black holes. Prog Theor Phys Suppl.

[CR162] Nakano H, Healy J, Lousto CO, Zlochower Y (2015). Perturbative extraction of gravitational waveforms generated with
numerical relativity. Phys Rev D.

[CR163] Nerozzi A (2007). Scalar functions for wave extraction in numerical
relativity. Phys Rev D.

[CR164] Nerozzi A, Beetle C, Bruni M, Burko LM, Pollney D (2005). Towards wave extraction in numerical relativity: the quasi-Kinnersley
frame. Phys Rev D.

[CR165] Nerozzi A, Bruni M, Re V, Burko LM (2006). Towards a wave-extraction method for numerical relativity. IV: testing
the quasi-Kinnersley method in the Bondi–Sachs framework. Phys Rev D.

[CR166] Newman ET, Penrose R (1963). An approach to gravitational radiation by a method of spin
coefficients. J Math Phys.

[CR167] Newman ET, Penrose R (1966). Note on the Bondi–Metzner–Sachs group. J Math Phys.

[CR168] Newman ET, Silva-Ortigoza G (2006). Tensorial spin-s harmonics. Class Quantum Gravity.

[CR169] Oechslin R, Rosswog S, Thielemann FK (2002). Conformally flat smoothed particle hydrodynamics application to
neutron star mergers. Phys Rev D.

[CR170] Ott CD (2011). Dynamics and gravitational wave signature of collapsar
formation. Phys Rev Lett.

[CR171] Pazos E, Dorband EN, Nagar A, Palenzuela C, Schnetter E, Tiglio M (2007). How far away is far enough for extracting numerical waveforms, and how
much do they depend on the extraction method?. Class Quantum Gravity.

[CR172] Penrose R (1963). Asymptotic properties of fields and space-times. Phys Rev Lett.

[CR173] Penrose R, Infeld L (1964). The light cone at infinity. Relativistic Theories of gravitation.

[CR174] Penrose R (1965a) Gravitational collapse and space-time singularities. Phys Rev Lett 14:57. doi:10.1103/PhysRevLett.14.57

[CR175] Penrose R (1965b) Zero rest-mass fields including gravitation: asymptotic behaviour. Proc R Soc Lond Ser A 284:159–203. doi:10.1098/rspa.1965.0058

[CR176] Penrose R, Rindler W (1984). Spinors and spacetime, vol. 1: two-spinor calculus and relativistic
fields.

[CR177] Penrose R, Rindler W (1986). Spinors and spacetime, vol. 2: spinor and twistor methods in space-time
geometry.

[CR178] Pfeiffer HP (2007). Reducing orbital eccentricity in binary black hole
simulations. Class Quantum Gravity.

[CR179] Poisson E (2004). Absorption of mass and angular momentum by a black hole: time-domain
formalisms for gravitational perturbations, and the small-hole or slow-motion
approximation. Phys Rev D.

[CR180] Poisson E, Pound A, Vega I (2011) The motion of point particles in curved spacetime. Living Rev Relativ 14:lrr-2011-7. doi:10.12942/lrr-2011-7. http://www.livingreviews.org/lrr-2011-7, arXiv:1102.0529 10.12942/lrr-2011-7 PMC5255936 28179832

[CR181] Pollney D (2007). Recoil velocities from equal-mass binary black-hole mergers: a
systematic investigation of spin-orbit aligned configurations. Phys Rev D.

[CR182] Pollney D, Reisswig C, Dorband N, Schnetter E, Diener P (2009). The asymptotic falloff of local waveform measurements in numerical
relativity. Phys Rev D.

[CR183] Pollney D, Reisswig C, Schnetter E, Dorband N, Diener P (2011). High accuracy binary black hole simulations with an extended wave
zone. Phys Rev D.

[CR184] Press WH (1971). Long wave trains of gravitational waves from a vibrating black
hole. Astrophys J.

[CR185] Pretorius F (2005). Numerical relativity using a generalized harmonic
decomposition. Class Quantum Gravity.

[CR186] Price RH (1972). Nonspherical perturbations of relativistic gravitational collapse. I.
Scalar and gravitational perturbations. Phys Rev D.

[CR187] Price RH (1972). Nonspherical perturbations of relativistic gravitational collapse. II.
Integer-spin, zero-rest-mass fields. Phys Rev D.

[CR188] Price RH, Pullin J (1994). Colliding black holes: the close limit. Phys Rev Lett.

[CR189] Regge T, Wheeler J (1957). Stability of a Schwarzschild singularity. Phys Rev.

[CR190] Reisswig C (2010) Binary black hole mergers and novel approaches to gravitational wave extraction in numerical relativity. PhD thesis, Universität Hannover, Hannover

[CR191] Reisswig C, Pollney D (2011). Notes on the integration of numerical relativity
waveforms. Class Quantum Gravity.

[CR192] Reisswig C, Bishop NT, Lai CW, Thornburg J, Szilagyi B (2007). Characteristic evolutions in numerical relativity using six angular
patches. Class Quantum Gravity.

[CR193] Reisswig C, Bishop NT, Pollney D, Szilágyi B (2009). Unambiguous determination of gravitational waveforms from binary black
hole mergers. Phys Rev Lett.

[CR194] Reisswig C, Bishop NT, Pollney D, Szilagyi B (2010). Characteristic extraction in numerical relativity: binary black hole
merger waveforms at null infinity. Class Quantum Gravity.

[CR195] Reisswig C, Ott CD, Sperhake U, Schnetter E (2011). Gravitational wave extraction in simulations of rotating stellar core
collapse. Phys Rev D.

[CR196] Reisswig C, Bishop NT, Pollney D (2013a) General relativistic null-cone evolutions with a high-order scheme. Gen Rel Gravit 45:1069–1094. doi:10.1007/s10714-013-1513-1. arXiv:1208.3891

[CR197] Reisswig C, Haas R, Ott CD, Abdikamalov E, Mösta P, Pollney D, Schnetter E (2013b) Three-dimensional general-relativistic hydrodynamic simulations of binary neutron star coalescence and stellar collapse with multipatch grids. Phys Rev D 87:064023. doi:10.1103/PhysRevD.87.064023. arXiv:1212.1191

[CR198] Reula OA (1998) Hyperbolic methods for Einstein’s equations. Living Rev Relativ 1:lrr-1998-3. doi:10.12942/lrr-1998-3. http://www.livingreviews.org/lrr-1998-3 10.12942/lrr-1998-3 PMC5253804 28191833

[CR199] Rezzolla L, Zanotti O (2013) Relativistic hydrodynamics. Oxford University Press, Oxford. doi:10.1093/acprof:oso/9780198528906.001.0001

[CR200] Rezzolla L, Abrahams AM, Matzner RA, Rupright ME, Shapiro SL (1999a) Cauchy-perturbative matching and outer boundary conditions: computational studies. Phys Rev D 59:064001. doi:10.1103/PhysRevD.59.064001. arXiv:gr-qc/9807047

[CR201] Rezzolla L, Shibata M, Asada H, Baumgarte TW, Shapiro SL (1999b) Constructing a mass-current radiation-reaction force for numerical simulations. Astrophys J 525:935–949. doi:10.1086/307942. arXiv:gr-qc/9905027

[CR202] Robinson I, Trautman A (1962). Some spherical gravitational waves in general
relativity. Proc R Soc Lond Ser A.

[CR203] Ruiz M, Alcubierre M, Núñez D, Takahashi R (2007). Multiple expansions for energy and momenta carried by gravitational
waves. Gen Relativ Gravit.

[CR204] Ruiz M, Alcubierre M, Núñez D, Takahashi R (2008). Multipole expansions for energy and momenta carried by gravitational
waves. Gen Relativ Gravit.

[CR205] Ruoff J (2001). New approach to the evolution of neutron star
oscillations. Phys Rev D.

[CR206] Ruoff J, Laguna P, Pullin J (2001). Excitation of neutron star oscillations by an orbiting
particle. Phys Rev D.

[CR207] Rupright ME, Abrahams AM, Rezzolla L (1998). Cauchy-perturbative matching and outer boundary conditions I: methods
and tests. Phys Rev D.

[CR208] Sachs RK (1962). Gravitational waves in general relativity VIII. Waves in
asymptotically flat space-time. Proc R Soc Lond Ser A.

[CR209] Santamaría L, Ohme F, Ajith P, Brügmann B, Dorband N, Hannam M, Husa S, Mösta P, Pollney D, Reisswig C, Robinson EL, Seiler J, Krishnan B (2010). Matching post-Newtonian and numerical relativity waveforms: systematic
errors and a new phenomenological model for non-precessing black hole
binaries. Phys Rev D.

[CR210] Sarbach O, Tiglio M (2001). Gauge invariant perturbations of Schwarzschild black holes in
horizon-penetrating coordinates. Phys Rev D.

[CR211] Scheel MA, Boyle M, Chu T, Kidder LE, Matthews KD, Pfeiffer HP (2009). High-accuracy waveforms for binary black hole inspiral, merger, and
ringdown. Phys Rev D.

[CR212] Seidel E (1990). Gravitational radiation from even-parity perturbations of stellar
collapse: mathematical formalism and numerical methods. Phys Rev D.

[CR213] Seidel E (1991). Normal mode excitation from stellar collapse to a black hole:
odd-parity perturbations. Phys Rev D.

[CR214] Seidel E, Da Costa GS, Demarque P (1987). Intermediate-age core helium burning stars and the distance to the
magellanic clouds. Astrophys J.

[CR215] Seidel E, Myra ES, Moore T (1988). Gravitational radiation from type-ii supernovae: the effect of the
high-density equation of state. Phys Rev D.

[CR216] Shibata M, Nakamura T (1995). Evolution of three-dimensional gravitational waves: harmonic slicing
case. Phys Rev D.

[CR217] Shibata M, Sekiguchi Y (2004). Gravitational waves from axisymmetric rotating stellar core collapse
to a neutron star in full general relativity. Phys Rev D.

[CR218] Shibata M, Sekiguchi Y (2005). Three-dimensional simulations of stellar core collapse in full general
relativity: nonaxisymmetric dynamical instabilities. Phys Rev D.

[CR219] Shibata M, Sekiguchi YI (2003). Gravitational waves from axisymmetrically oscillating neutron stars in
general relativistic simulations. Phys Rev D.

[CR220] Shibata M, Taniguchi K, Uryū K (2003). Merger of binary neutron stars of unequal mass in full general
relativity. Phys Rev D.

[CR221] Siebel F, Font JA, Müller E, Papadopoulos P (2003). Axisymmetric core collapse simulations using characteristic numerical
relativity. Phys Rev D.

[CR222] Smarr LL (1977). Spacetimes generated by computers: black holes with gravitational
radiation. Ann NY Acad Sci.

[CR223] Sopuerta CF, Yunes N, Laguna P (2006). Gravitational recoil from binary black hole mergers: the close-limit
approximation. Phys Rev D.

[CR224] Stewart JM (1990). Advanced general relativity, Cambridge monographs on mathematical
physics.

[CR225] Stewart JM, Friedrich H (1982). Numerical relativity. I. The characteristic initial value
problem. Proc R Soc Lond Ser A.

[CR226] Szilágyi B (2000) Cauchy-characteristic matching in general relativity. PhD thesis, University of Pittsburgh, Pittsburgh. arXiv:gr-qc/0006091

[CR227] Szilágyi B, Winicour J (2003). Well-posed initial-boundary evolution in general
relativity. Phys Rev D.

[CR228] Szilágyi B, Gómez R, Bishop NT, Winicour J (2000). Cauchy boundaries in linearized gravitational theory. Phys Rev D.

[CR229] Szilágyi B, Schmidt B, Winicour J (2002). Boundary conditions in linearized harmonic gravity. Phys Rev D.

[CR230] Szilágyi B, Lindblom L, Scheel MA (2009). Simulations of binary black hole mergers using spectral
methods. Phys Rev D.

[CR231] Tamburino LA, Winicour J (1966). Gravitational fields in finite and conformal Bondi
frames. Phys Rev.

[CR232] Taylor NW, Boyle M, Reisswig C, Scheel MA, Chu T, Kidder LE, Szilágyi B (2013). Comparing gravitational waveform extrapolation to
Cauchy-characteristic extraction in binary black hole simulations. Phys Rev D.

[CR233] Teukolsky SA (1972). Rotating black holes: separable wave equations for gravitational and
electromagnetic perturbations. Phys Rev Lett.

[CR234] Teukolsky SA (1973). Perturbations of a rotating black hole. I. Fundamental equations for
gravitational, electromagnetic, and neutrino-field perturbations. Astrophys J.

[CR235] Thorne K (1980). Gravitational-wave research: current status and future
prospects. Rev Mod Phys.

[CR236] Thorne K (1980). Multipole expansions of gravitational radiation. Rev Mod Phys.

[CR237] Tominaga K, Saijo M, Maeda KI (1999). Gravitational waves from a test particle scattered by a neutron star:
axial mode case. Phys Rev D.

[CR238] Vishveshwara CV (1970). Scattering of gravitational radiation by a Schwarzschild
black-hole. Nature.

[CR239] Vishveshwara CV (1970). Stability of the Schwarzschild metric. Phys Rev D.

[CR240] Wald RM (1984). General relativity.

[CR241] Winicour J (1968). Some total invariants of asymptotically flat
space-times. J Math Phys.

[CR242] Winicour J, Held A (1980). Angular momentum in general relativity. General relativity and gravitation: one hundred years after the birth of
Albert Einstein.

[CR243] Winicour J (2005) Characteristic evolution and matching. Living Rev Relativ 8:lrr-2005-10. doi:10.12942/lrr-2005-10. http://www.livingreviews.org/lrr-2005-10, arXiv:gr-qc/0508097 10.12942/lrr-2005-10 PMC5256076 28179870

[CR244] York JW, Smarr LL (1979). Kinematics and dynamics of general relativity. Sources of gravitational radiation.

[CR245] Zanotti O, Rezzolla L, Font JA (2003). Quasi-periodic accretion and gravitational waves from oscillating
“toroidal neutron stars” around a Schwarzschild black
hole. Mon Not R Astron Soc.

[CR246] Zerilli FJ (1970). Effective potential for even-parity Regge–Wheeler
gravitational perturbation equations. Phys Rev Lett.

[CR247] Zerilli FJ (1970). Gravitational field of a particle falling in a Schwarzschild geometry
analyzed in tensor harmonics. Phys Rev D.

[CR248] Zerilli FJ (1970). Tensor harmonics in canonical form for gravitational radiation and
other applications. J Math Phys.

[CR249] Zlochower Y, Gómez R, Husa S, Lehner L, Winicour J (2003). Mode coupling in the nonlinear response of black holes. Phys Rev D.

[CR250] Zwerger T, Müller E (1997). Dynamics and gravitational wave signature of axisymmetric rotational
core collapse. Astron Astrophys.

